# Who is pregnant? Defining real-world data-based pregnancy episodes in the National COVID Cohort Collaborative (N3C)

**DOI:** 10.1093/jamiaopen/ooad067

**Published:** 2023-08-16

**Authors:** Sara E Jones, Katie R Bradwell, Lauren E Chan, Julie A McMurry, Courtney Olson-Chen, Jessica Tarleton, Kenneth J Wilkins, Victoria Ly, Saad Ljazouli, Qiuyuan Qin, Emily Groene Faherty, Yan Kwan Lau, Catherine Xie, Yu-Han Kao, Michael N Liebman, Federico Mariona, Anup P Challa, Li Li, Sarah J Ratcliffe, Melissa A Haendel, Rena C Patel, Elaine L Hill, N3C Consortium, Adam B Wilcox, Adam M Lee, Alexis Graves, Alfred (Jerrod) Anzalone, Amin Manna, Amit Saha, Amy Olex, Andrea Zhou, Andrew E Williams, Andrew Southerland, Andrew T Girvin, Anita Walden, Anjali A Sharathkumar, Benjamin Amor, Benjamin Bates, Brian Hendricks, Brijesh Patel, Caleb Alexander, Carolyn Bramante, Cavin Ward-Caviness, Charisse Madlock-Brown, Christine Suver, Christopher Chute, Christopher Dillon, Chunlei Wu, Clare Schmitt, Cliff Takemoto, Dan Housman, Davera Gabriel, David A Eichmann, Diego Mazzotti, Don Brown, Eilis Boudreau, Elizabeth Zampino, Emily Carlson Marti, Emily R Pfaff, Evan French, Farrukh M Koraishy, Federico Mariona, Fred Prior, George Sokos, Greg Martin, Harold Lehmann, Heidi Spratt, Hemalkumar Mehta, Hongfang Liu, Hythem Sidky, J W Awori Hayanga, Jami Pincavitch, Jaylyn Clark, Jeremy Richard Harper, Jessica Islam, Jin Ge, Joel Gagnier, Joel H Saltz, Johanna Loomba, John Buse, Jomol Mathew, Joni L Rutter, Justin Starren, Karen Crowley, Katie Rebecca Bradwell, Kellie M Walters, Ken Wilkins, Kenneth R Gersing, Kenrick Dwain Cato, Kimberly Murray, Kristin Kostka, Lavance Northington, Lee Allan Pyles, Leonie Misquitta, Lesley Cottrell, Lili Portilla, Mariam Deacy, Mark M Bissell, Marshall Clark, Mary Emmett, Mary Morrison Saltz, Matvey B Palchuk, Meredith Adams, Meredith Temple-O'Connor, Michael G Kurilla, Michele Morris, Nabeel Qureshi, Nasia Safdar, Nicole Garbarini, Noha Sharafeldin, Ofer Sadan, Patricia A Francis, Penny Wung Burgoon, Peter Robinson, Philip R O Payne, Rafael Fuentes, Randeep Jawa, Rebecca Erwin-Cohen, Rena Patel, Richard A Moffitt, Richard L Zhu, Rishi Kamaleswaran, Robert Hurley, Robert T Miller, Saiju Pyarajan, Sam G Michael, Samuel Bozzette, Sandeep Mallipattu, Satyanarayana Vedula, Scott Chapman, Shawn T O'Neil, Soko Setoguchi, Stephanie S Hong, Steve Johnson, Tellen D Bennett, Tiffany Callahan, Umit Topaloglu, Usman Sheikh, Valery Gordon, Vignesh Subbian, Warren A Kibbe, Wenndy Hernandez, Will Beasley, Will Cooper, William Hillegass, Xiaohan Tanner Zhang

**Affiliations:** Office of Data Science and Emerging Technologies, National Institute of Allergy and Infectious Diseases, National Institutes of Health, Rockville, MD 20852, United States; Palantir Technologies, Denver, CO 80202, United States; College of Public Health and Human Sciences, Oregon State University, Corvallis, OR 97331, United States; Department of Biomedical Informatics, University of Colorado, Anschutz Medical Campus, Aurora, CO 80045, United States; Department of Obstetrics and Gynecology, University of Rochester Medical Center, Rochester, NY 14620, United States; Department of Obstetrics and Gynecology, Medical University of South Carolina, Charleston, SC 29425, United States; Biostatistics Program, Office of the Director, National Institute of Diabetes and Digestive and Kidney Diseases, National Institutes of Health, Bethesda, MD 20892, United States; Department of Obstetrics and Gynecology, University of Rochester Medical Center, Rochester, NY 14620, United States; Palantir Technologies, Denver, CO 80202, United States; Department of Public Health Sciences, University of Rochester Medical Center, Rochester, NY 14618, United States; School of Public Health, University of Minnesota, Minneapolis, MN 55455, United States; Sema4, Stamford, CT 06902, United States; Department of Public Health Sciences, University of Rochester Medical Center, Rochester, NY 14618, United States; Sema4, Stamford, CT 06902, United States; IPQ Analytics, LLC, Kennett Square, PA 19348, United States; Beaumont Hospital, Dearborn, MI 48124, United States; Wayne State University, Detroit, MI 48202, United States; Department of Chemical and Biomolecular Engineering, Vanderbilt University, Nashville, TN 37212, United States; Sema4, Stamford, CT 06902, United States; Department of Public Health Sciences, University of Virginia, Charlottesville, VA 22903, United States; College of Public Health and Human Sciences, Oregon State University, Corvallis, OR 97331, United States; Department of Medicine and Global Health, University of Washington, Seattle, WA 98105, United States; Department of Obstetrics and Gynecology, University of Rochester Medical Center, Rochester, NY 14620, United States; Department of Public Health Sciences, University of Rochester Medical Center, Rochester, NY 14618, United States

**Keywords:** electronic health records, pregnancy, algorithms, COVID-19, gestational age

## Abstract

**Objectives:**

To define pregnancy episodes and estimate gestational age within electronic health record (EHR) data from the National COVID Cohort Collaborative (N3C).

**Materials and Methods:**

We developed a comprehensive approach, named Hierarchy and rule-based pregnancy episode Inference integrated with Pregnancy Progression Signatures (HIPPS), and applied it to EHR data in the N3C (January 1, 2018–April 7, 2022). HIPPS combines: (1) an extension of a previously published pregnancy episode algorithm, (2) a novel algorithm to detect gestational age-specific signatures of a progressing pregnancy for further episode support, and (3) pregnancy start date inference. Clinicians performed validation of HIPPS on a subset of episodes. We then generated pregnancy cohorts based on gestational age precision and pregnancy outcomes for assessment of accuracy and comparison of COVID-19 and other characteristics.

**Results:**

We identified 628 165 pregnant persons with 816 471 pregnancy episodes, of which 52.3% were live births, 24.4% were other outcomes (stillbirth, ectopic pregnancy, abortions), and 23.3% had unknown outcomes. Clinician validation agreed 98.8% with HIPPS-identified episodes. We were able to estimate start dates within 1 week of precision for 475 433 (58.2%) episodes. 62 540 (7.7%) episodes had incident COVID-19 during pregnancy.

**Discussion:**

HIPPS provides measures of support for pregnancy-related variables such as gestational age and pregnancy outcomes based on N3C data. Gestational age precision allows researchers to find time to events with reasonable confidence.

**Conclusion:**

We have developed a novel and robust approach for inferring pregnancy episodes and gestational age that addresses data inconsistency and missingness in EHR data.

## Objectives

Pregnancy episodes in electronic health record (EHR) data are often inconsistently coded across patients. Our objective was to leverage pregnancy concepts recorded in the nationally pooled EHR data of the National COVID Cohort Collaborative (N3C) to comprehensively infer pregnancy start and end dates using novel algorithms and to show the utility of these pregnancy episodes as a resource for COVID-19 research.

## Background and significance

The COVID-19 pandemic has substantially impacted daily life and is especially concerning for vulnerable populations. Pregnant persons appear to be at higher risk for incident and severe COVID-19 infections than non-pregnant persons.^1–4^ Increased rates of cesarean section deliveries, lower gestational age at delivery, and preterm birth have been observed among pregnant persons with COVID-19.^5^ Pregnant persons with COVID-19 may also present more frequently with acute respiratory distress syndrome and hemolysis, elevated liver enzymes, and low platelets syndrome.^6^^,^^7^ Moreover, access to and utilization of healthcare services markedly changed during the pandemic,^1^^,^^2^^,^^8^^,^^9^ which may have impacted antenatal care. Numerous knowledge gaps related to pregnancy and COVID-19 persist, including the impact of vaccination and the mechanisms underlying elevated risk for various poor outcomes.

**Figure 1.**
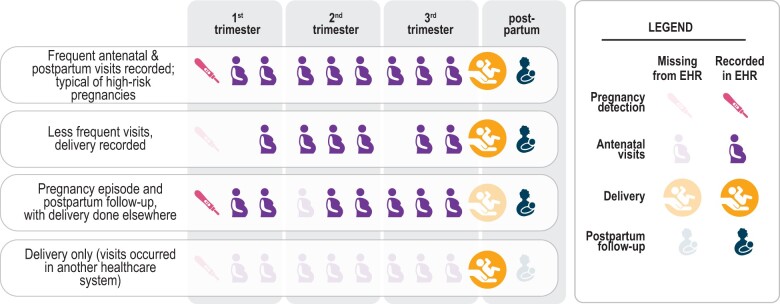
Common scenarios of pregnancy episodes in N3C illustrate how the EHR provides an incomplete picture of care. Some visits were not recorded (occurred in another healthcare system), and yet others were likely recorded inconsistently or inaccurately. Some routine visits may have occurred in another healthcare system or may not have occurred at all, potentially due to healthcare disruption caused by the pandemic.

EHR data can inform these knowledge gaps. The N3C^10^ offers COVID-19 researchers access to harmonized EHR data from more than 12 million individuals from 72 data partner healthcare systems (hereafter, sites; as of data release on April 7, 2022). This is the first and largest publicly available EHR repository with national sampling in the United States. However, data fields for start and end of pregnancies and gestational age at birth do not currently exist in a consistent form within EHRs, making it challenging to ascertain pregnancy episodes and gestational age. Further, sites differ widely on how accurately and consistently this information is collected in their respective EHRs (Figure 1); moreover, the “gold-standard” of birth certificate-based data are usually unavailable in EHRs. Use of the N3C provides an opportunity to investigate and standardize pregnancy episodes and gestational age at a national scale.

To overcome these limitations, EHR-based investigations commonly utilize algorithms for determining gestational age and pregnancy (or delivery) episodes using diagnosis codes and delivery dates.^11–22^ In N3C, we started with an existing algorithm using Observational Medical Outcomes Partnership (OMOP) concepts defined in Matcho et al.^12^ However, this and similar algorithms rely heavily on anchoring the pregnancy with a specifically recorded pregnancy outcome (eg, live birth), and do not leverage available pregnancy data for which an outcome is missing or may not have occurred yet, with the exception of the algorithm by Bertoia et al.^20^ They also rely on approximations based on the pregnancy outcome or available pregnancy markers to infer pregnancy start, which may not always be recorded accurately in the data or may be absent from the EHR. Therefore, we adapted Matcho et al^12^ and layered on our own data-driven algorithms to robustly and precisely infer pregnancy start or last menstrual period (LMP) in a similar spirit to work done by others (eg, Bertoia et al who used ICD-10-CM Z3A codes in claims data^20^). This more comprehensive and inclusive approach provides a robust foundation for public health research using harmonized data on pregnant persons beyond COVID-19. New exposures, such as medications, vaccines, or emerging pathogens, among pregnant persons will always require real-world data, including from EHRs, to address timely questions as this group continues to be largely excluded from trials or efficacy studies.^23^^,^^24^

## Materials and methods

### N3C cohort preparation

Detailed information on overall N3C organization can be found in previously published reports (Text S1A).^10^ Briefly, the N3C, which is overseen by the National Center for Advancing Translational Science (NCATS) of the National Institutes of Health (NIH), collects and harmonizes information from single- and multi-hospital health systems across the United States and stores data in a central location, the N3C Data Enclave. For this study, we used a limited dataset, which contains deidentified data, 5-digit patient ZIP codes, and exact dates of COVID-19 diagnoses and service use. The N3C Data Enclave’s Palantir Foundry platform, a secure analytics platform, was used for data access and analysis. Each N3C data partner site provides demographic, visit, vital status, medication, laboratory, and diagnosis data; data are harmonized to the OMOP Common Data Model (CDM) version 5.3.1.^25^ The N3C cohort is composed of COVID-19-positive patients as well as COVID-19-negative controls matched on as many of the 4 sociodemographic variables (ie, age, sex, race, and ethnicity) as possible. Any COVID-19-positive patient from an N3C study site is included in the database if the following criteria are met: an encounter after January 1, 2020 that includes (1) one of a set of a priori-defined SARS-CoV-2 laboratory tests, (2) a “strong positive” diagnosis code, or (3) 2 “weak positive” diagnosis codes during one encounter or on the same date prior to May 1, 2020. The cohort definition and the diagnosis codes used are publicly available on GitHub.^26^ For patients included in N3C, encounters in the same data partner site on or after January 1, 2018 are also included (ie, “lookback data”). We utilized N3C dataset release version 73, April 7, 2022.

### Overview of our HIPPS approach to inferring pregnancy episodes, gestational age, and pregnancy start dates

**Figure 2.**
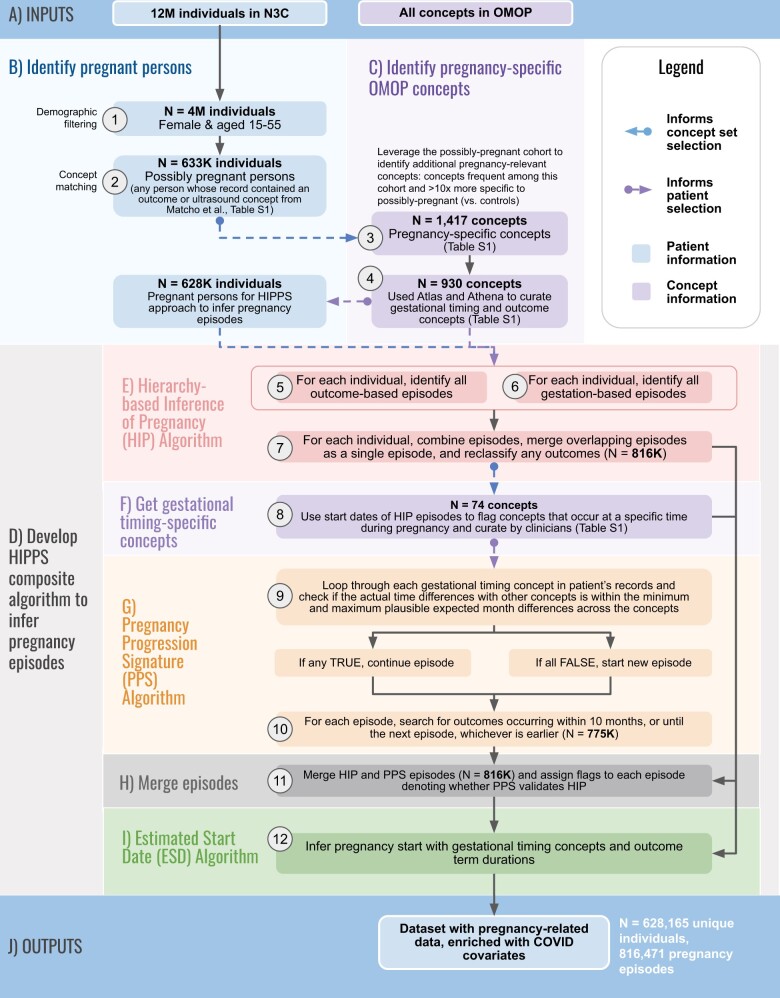
Overview of HIPPS. The inputs (A) of our composite algorithm are all individuals and the full set of OMOP concepts in N3C. From these, we identify pregnant persons (B) and identify pregnancy-specific concepts (C). Our HIPPS algorithm (D) is comprised of the Hierarchy-based Inference of Pregnancy (HIP) algorithm (E), Get gestational timing concepts (F), Pregnancy Progression Signature (PPS) Algorithm (G), Merge episodes (H), and Estimated Start Date (ESD) Algorithm (I). The output is a dataset with pregnancy-related data and enriched with COVID covariates. The following are the individual steps within each panel. (1) From the 12 million (M) patients in N3C, we identified 4M that were both female and of reproductive age (15–55 years). (2) Of these, we identified 633K possibly pregnant persons who matched at least one concept in an initial set of ultrasound and pregnancy outcome concepts from Matcho et al.^12^ (3) To develop an enriched set of concepts specific for pregnancy, we then assessed concept frequency among the initial cohort of possibly pregnant persons and chose 1417 concepts that were present in at least 1000 individuals and were 10X (determined empirically via distribution analysis) more frequent among possibly pregnant persons relative to controls (all other patients in N3C). (4) We utilized Athena and Atlas to expand and curate the concepts from Matcho et al related to pregnancy outcomes and gestational age. (5–7) For each of the 628K pregnant persons selected for the HIPPS approach based on the presence of at least one of the 930 concepts in their records, we inferred pregnancy episodes with the Hierarchy-based Inference of Pregnancy (HIP) Algorithm. Gestation-based and outcome-based episodes were first defined before combining them for each pregnant person. Any overlapping episodes were merged as a single episode and any outcomes were reclassified if the gestational age info did not align with the outcome. (8) We then used the start dates of the HIP episodes to discover concepts that occur during a specific time during pregnancy from the 1417 pregnancy-specific concepts derived from Step 3. Concepts with a standard deviation of <1.5 months were kept for clinicians to vet and assign minimum and maximum months of when these concepts most likely occur during pregnancy. (9) The Progressing Pregnancy Signature (PPS) Algorithm was applied to both (A) validate HIP algorithm predictions and (B) provide further evidence of pregnancy in the data using a separate set of concepts and logic: iterations of comparisons of each concept with other gestational timing concepts for each person were performed, followed by checks of whether the actual difference in dates was within the minimum and maximum plausible expected months for the concepts. If at least one comparison between concepts evaluated to “TRUE”, the algorithm continued building the pregnancy episode. If all evaluated to “FALSE”, then a new pregnancy episode was begun if a minimum permissible retry period of 60 days was also met. (10) For each PPS episode, outcomes were added if they occurred within 10 months or until the next episode, whichever was earlier. (11) We then combined HIP and PPS episodes and checked which HIP episodes and outcomes were supported by PPS. (12) Lastly, pregnancy start dates were calculated using the Estimated Start Date (ESD) Algorithm using the gestational timing concepts from Step 8. The result of the HIPPS approach is a dataset that can be enriched to include other variables of interest related to pregnancy and in our case, COVID-19.

Our composite algorithm, named Hierarchy and rule-based pregnancy episode Inference integrated with Pregnancy Progression Signatures (HIPPS), uses existing and data-driven analytic methods to identify pregnancies (Figure 2); HIPPS classifies each as a “pregnancy episode” recording all pregnancies per person (ie, allowing an individual to be pregnant more than once during our observation period). Briefly, we first identified all females of reproductive age (15–55 years, inclusive) within N3C and applied pregnancy-specific concepts to identify potential pregnancies (Figure 2.1–4). Next, we applied 2 distinct algorithms to infer and validate unique pregnancy episodes (Figure 2.5–11). Then, we used gestational timing concepts to rigorously determine pregnancy episode start dates and their precision level (Figure 2.12). Finally, we merged the pregnancy episodes data with other pregnancy-related information as well as metrics important to COVID-19 research (Figure 2.J).

**Figure 3.**
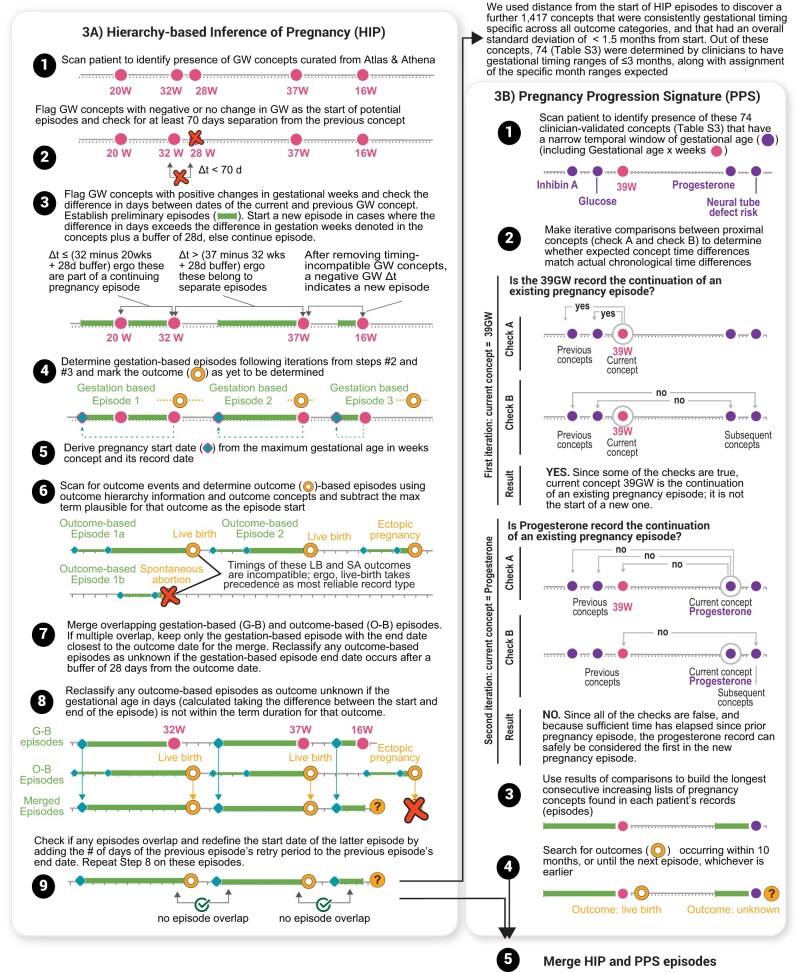
Inference of pregnancy episodes. (A) Definition of Hierarchy-based Inference of Pregnancy (HIP) algorithm: episodes were inferred by both an initial set of gestational timing markers (gestation-based episodes) and outcomes (outcome-based episodes), shown in steps 1–6. Both types of episodes were then merged and quality checked (steps 7–9). (B) Definition of Pregnancy Progression Signature (PPS) algorithm: we leveraged further, empirically derived, gestational timing markers based on low standard deviation distance from HIP algorithm start dates across any pregnancy outcome category, followed by clinician curation of expected gestational timing ranges (*N* = 74 concepts). The patient records were first scanned for this new set of gestational timing concepts (step 1), followed by detailed iterations across the patient data making comparisons between pairs of concepts to determine whether to extend or start a new episode (steps 2–3). To provide further clarity, equations representing these iterations and detailed application to an example patient can be found in Figure S1. Where present, outcomes were appended to the end of the progression signatures to derive the full recorded episode (step 4). Finally, we first check for any overlap of HIP and PPS episodes. Then we have additional steps to remove episodes that overlap with more than 2 episodes—prioritizing the episodes with the closest end dates. The resulting pairs of overlapping episodes are merged using a union of the 2 source episodes (eg, taking the earliest date from both, and the latest date from both) (step 5). Note that for both algorithms these panels provide the full algorithm definition, examples are used purely to provide clarity.

### Selecting relevant concepts for computable pregnancy phenotype and gestational age

As an initial step to define a computable phenotype for pregnancy in N3C, we started from a previously published concept set^12^ and enriched it with additional concepts related to outcomes and gestational age using a combination of concept curation and concept frequency analysis. This resulted in an expanded list of OMOP concepts that were relevant to either known pregnancy outcomes or gestational age (Table S1, Text S1B). The following steps of the HIPPS approach describe the logic we implemented to complete the full computable phenotype definition of pregnancy.

### Hierarchy-based inference of pregnancy (HIP) algorithm: rule-based algorithm

To capture pregnancies without an outcome recorded in N3C, we developed the HIP Algorithm (Figure 3A, Text S1C and D). Firstly, we used the “Outcome assessment and classification” step^12^ to define episodes with outcomes using concepts listed in Table S1. We applied the minimum required duration between consecutive outcomes to determine distinct pregnancy episodes per patient. If a patient has more than one outcome type, the outcomes were assessed in this order: live birth, stillbirth, ectopic pregnancy, abortion (separately for spontaneous and induced), and delivery record only. For example, if a patient has both live birth and stillbirth outcomes, we first checked that there was at least 182 days or 26 weeks between live birth outcomes and 168 days or 24 weeks between stillbirth outcomes. Of the outcomes that met these requirements, we then checked that any stillbirth outcome followed at least 24 weeks or preceded at least 26 weeks a live birth outcome. Secondly, we used gestational age concepts that were prevalent in N3C data and had week-level resolution (GW) for estimating gestational age such as “Gestation period, X weeks” to identify gestation-based episodes without outcomes. We marked any GW record as a potential start of a new episode if the number corresponding to the week is less than or the same as the previous GW record’s week. We performed additional checks to ensure that the projected start date of any starting GW record of a new episode does not overlap with a previous episode. Once the initial gestation-based episodes were delineated, we then calculated the start date of a gestation-based episode by tracking backwards the maximum gestational age in weeks from its corresponding record date. Lastly, gestation-based episodes were combined with outcome-based episodes if they overlapped. If the maximum gestational age of an overlapping gestation-based episode does not align with the expected term duration of the outcome, then the outcome is removed. This step eliminates misclassified outcomes. If an outcome-based episode does not overlap with a gestation-based episode, then the start date is calculated using the maximum possible outcome-specific estimate (ie, 301 days or 43 weeks for live birth).

### Pregnancy progression signature (PPS) algorithm: novel temporal sequence analysis for detecting pregnancy episodes

We developed the PPS algorithm to validate plausible episodes identified by the HIP algorithm (Figure 3B, Figure S1, Text S1E). PPS searches for signatures of progressing pregnancy concepts across each patient record using an adaptation of longest increasing consecutive subsequence (LICS) analysis^27^ to find the longest subsequence of a given set of concepts in which all concepts of the subsequence appear in increasing order based on when they occur during pregnancy to define a pregnancy episode. To create a set of progressing pregnancy concepts, we assessed the frequently recorded pregnancy-related concepts from our concept frequency analysis and determined the mean and standard deviation of when these concepts occur relative to the start of each HIP episode with a pregnancy outcome. After filtering for concepts with a standard deviation <1.5 months, our clinicians reviewed these concepts and provided expected time ranges of occurrence during pregnancy for each concept. Any concepts where the gestational time range spanned >3 months were removed. This resulted in 74 progressing pregnancy concepts (eg, “Glucose; tolerance test (GTT), 3 specimens (includes glucose)”). Time intervals across these concepts in the pregnant person’s data were compared to their expected gestational time ranges. For each concept, (1) comparisons were made with all previous concepts and the current concept and (2) between each successive pair of concepts surrounding the current concept. If any comparison yielded discordance between the actual time elapsed between concepts and the expected gestational time ranges, this may indicate the start of a new episode. We then checked if the minimum permissible time between the end of a pregnancy episode and the start of a subsequent pregnancy episode of 2 months (retry period) was met. If there was at least a 2-month separation between the current and previous concept, a new pregnancy episode was assigned. These multiple comparisons and checks per concept address the influence of any historical records or recording of concepts outside the expected gestational time range (eg, gestational diabetes screening occurring before 6 months or 24 weeks) from intervening with finding the true progressing sequence of gestational timing concepts. Further cleanup removes any episodes with a length greater than 12 months or patients with more than 5 episodes occurring in a year. To assign a measure of support for outcomes identified in HIP episodes, we inferred pregnancy outcomes in the PPS by checking for any outcome within a window of 14 days before the episode end to up to the earliest date from either (1) the next episode start date, or (2) up to 10 months minus the last record’s expected minimum month after the start of the episode. We then selected the outcome based on Matcho et al’s outcome hierarchy assessment.^12^

### Estimated start date (ESD) algorithm—estimating precise pregnancy start dates

**Figure 4.**
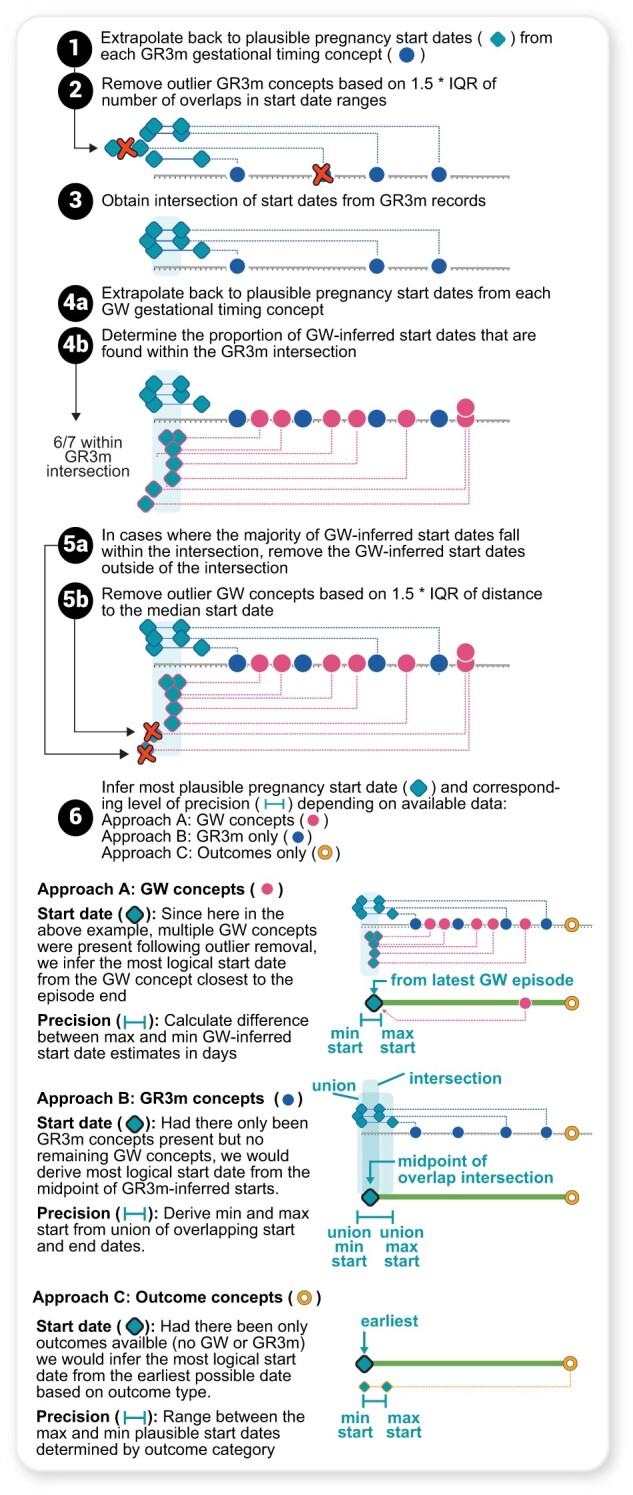
Inferring pregnancy start dates using the Estimated Start Date (ESD) algorithm. The 74 concepts determined to be gestational timing specific were split into 2 types: Gestational Range 3 months (GR3m) and Gestational Week (GW). GR3m indicated concepts that have a possible gestational timing span of >1 week but <3 months at the point they occur during pregnancy (eg, estriol can be tested between months 3.75–5.5). GW indicated concepts of type “Gestation period, X weeks,” denoted as point estimates since week-level was the smallest unit of precision for gestational timing in this study. Start dates or plausible start date ranges from these concepts were determined by extrapolating backwards using gestational timing information, and outliers were removed using the 1.5*IQR on each concept type and the overlap of intersecting start date ranges and point estimates (steps 1–5b). The most logical start dates were then assigned based on concept types present, and levels of precision of start date estimate were added (step 6).

To estimate the start date (LMP) for identified pregnancies (Figure 4, Text S1F and G) we leveraged the 74 clinician-validated concepts, which included concepts with week-level resolution (GW) and resolution between 1-week and 3 months (GR3m) (eg, “Trisomy 18 risk in Fetus,” which is generally assessed between 12 and 24 weeks). We first identified all GW and GR3m concepts within each pregnancy episode, kept only the max GW concept if there was more than one type of GW concept sharing the same date, and inferred the pregnancy start dates for each recorded concept. Next, we filtered out outlier GW concepts that did not fall within the intersection of the inferred pregnancy start dates of the GR3m concepts and did not meet the 1.5*interquartile range (IQR) threshold of the GW concept start dates. These steps help to remove any concept that may occur much earlier or later than the concept’s expected gestational time range. For example, concepts related to gestational diabetes screening (eg, “Glucose; tolerance test (GTT), 3 specimens (includes glucose)”) may take place earlier in some pregnancies than expected (even if the data are clinically accurate). Instead, we rely on the other concepts within the episode to estimate LMP. GW concepts that occurred latest in pregnancy were selected as the inferred pregnancy start date because the dates of these GW concepts appeared to be more precise based on empirical analysis (Figures S6 and S7).

### Precision categories and outcome concordance scores

We developed 2 types of precision metrics: (1) the precision of the estimated LMP and (2) the agreement of the outcomes assigned by HIP and PPS algorithms. For (1), we assigned precision categories based on the difference in days of the first and last possible start dates: “week_poor-support”, precision ≤ 7 days but a single GW concept was used to extrapolate start date; “week”, precision ≤ 7 days; “two-week”, 7<precision ≤ 14 days; “three-week”, 14<precision ≤ 21 days; “month”, 21<precision ≤ 28 days; “two-months”, 28<precision ≤ 56 days; “three-months”, 56<precision ≤ 84 days; “non-specific”, no timing information. For comparison, we used a baseline method that obtains the start dates using only the GW concepts within an episode without removal of outliers and assigned precision based on the maximum start date difference between GW concepts. To assign outcome concordance scores (2), we merged episodes of the above algorithms, HIP and PPS, and added flags for whether the episode was detected by either algorithm. We then checked if PPS also detected the same outcome as HIP within ±14 days as a measure of support for the inferred pregnancy outcome from HIP. If the outcomes do not match, the outcome that occurs later is kept as the final outcome for that episode. If one of the episodes does not have an outcome in one of the algorithms, then the outcome from the other algorithm is designated as the final outcome. Using this information, we created an outcome concordance score from 0 to 2 where 2 indicates an episode with both of the following features: (1) outcome matched and outcome dates are within 14 days of each other, and (2) estimated gestational age at the outcome date is within the expected term duration of that outcome; 1 indicates an episode with an outcome within the expected term duration but no outcome match; 0 indicates an episode with an outcome not within the expected term duration even with an outcome match.

### Addition of pregnancy-related information to each pregnancy episode

For each pregnant person and pregnancy episode, we ascertained sociodemographic information (eg, age, self-reported race/ethnicity) and other pregnancy-related information (eg, preterm status). “Unknown” is deterministically imputed and other privacy measures enforced for American Indian/Alaska Native participants in accordance with NIH/NCATS data governance agreements with American Indian/Alaska Native sovereign tribal nations. We created OMOP concept sets (Table S1) for pregnancy-related variables (Table S2) from all possible concepts available in various OMOP concept databases (eg, Athena^28^ and Atlas,^29^Figure 2.4); all concept sets were vetted by clinicians. For each pregnancy episode, we captured the recorded pregnancy lengths (time duration between start and end dates of records for pregnancy that occur within the EHR data) and inferred pregnancy lengths (time duration between LMP and pregnancy outcome dates estimated using HIPPS).

### Clinician validation

Manual chart review is not possible in N3C due to regulations that minimize reidentification risk; thus, we utilized clinician annotation to validate key inferences using N3C data (see Text S1H for details). We conducted this work in 2 stages: (1) an internal pilot stage (*n* = 70 records across sampling categories, randomly sampling 2:1 ratio of live birth to non-live-birth categories) to elicit a realistic range of validation-metric values; and (2) full validation substudy size based on the pilot estimates (*n* = 280 records; Figure S2A), motivated by a set of feasible sizes that are multiples of the internal pilot (Figure S2B). We provided 3 obstetrician-gynecologists with an N3C dataset containing all records from OMOP clinical data tables for measurements, observations, conditions, drugs, and procedures for 240 randomly selected and stratified pregnancy episodes greater than one day from 166 pregnant persons, of whom 72 had more than one recorded pregnancy, and 40 non-pregnant persons for a total of *N* = 280 (Figure S2B). Each clinician assessed the presence, number, dates, and outcomes of each pregnancy episode as well as the gestational age in weeks at outcome and estimated LMP. Additionally, the clinicians rated their level of confidence in annotating the above information with categories of not confident (−1), neutral (0), or confident (1). This information was collected using Fusion, a Palantir spreadsheet application, within the N3C enclave to minimize reidentification risk. Lastly, we had an additional clinician “double-review” a random 10% subset of the records to estimate (and thus ensure satisfactory) interrater agreement. We computed the percent agreement for pregnancy outcome category, gestational age in weeks, date-related metrics, and the total number of episodes to quantify concordance of HIPPS with clinicians’ validations (Text S1H).

### Assessment of pregnancy episode detection and gestational timing validation using alternative pregnancy-specific gestational timing concepts (external to HIPPS)

To provide further validation of HIPPS in detecting pregnancy episodes within N3C and inferring the start and end of pregnancy episodes, we specifically checked the overlap of 25 pregnancy-specific gestational timing concepts not used in HIPPS with the pregnancy episodes (Table S3). These concepts, which were identified as part of the PPS algorithm and had a standard deviation <1.5 months, were validated by clinicians to occur exclusively during pregnancy over a range wider than was suitable for episode definition (4–10 months). An example of a concept from this set is “Polyhydramnios”, a condition of excess amniotic fluid buildup during pregnancy, which occurs between 6 to 10 months (inclusive) during pregnancy and has a time range of 5 months (Table S3). For each concept, we calculated the percentage of occurrences by dividing the number of occurrences that overlap with a pregnancy episode within the expected gestational time range for that concept with the total number of occurrences.

### COVID-19 analysis

We used descriptive statistics to compare demographic characteristics across 3 types of pregnancy cohorts: (1) all episodes regardless of outcome category (**cohort 1: all episodes**), (2) episodes with outcomes (excluding delivery record only) and start dates with up to 1 month resolution for estimated start date (includes week level poor support) (**cohort 2: month-level)**, and (3) episodes with outcomes (excluding delivery record only) with high concordance (score of 2) between HIP and PPS with week-level resolution for estimated start date (**cohort 3: week-level)**. For each cohort, we stratified episodes into: (1) all episodes, (2) episodes ending before March 1, 2020, (3) episodes with indication of COVID-19-positivity after March 1, 2020, and (4) episodes without indication of COVID-19-positivity after March 1, 2020. We took the first occurrence of either a positive COVID-19 PCR or antigen (Ag) lab result or COVID-19 diagnosis code (U07.1) to assess COVID-19 positivity during pregnancy. COVID-19 screening was indicated if a COVID-19 PCR or Ag lab test, regardless of the result, occurred during pregnancy. We defined a COVID-19 reinfection as any infection that occurred at least 60 days after the index infection date.

## Results

**Figure 5.**
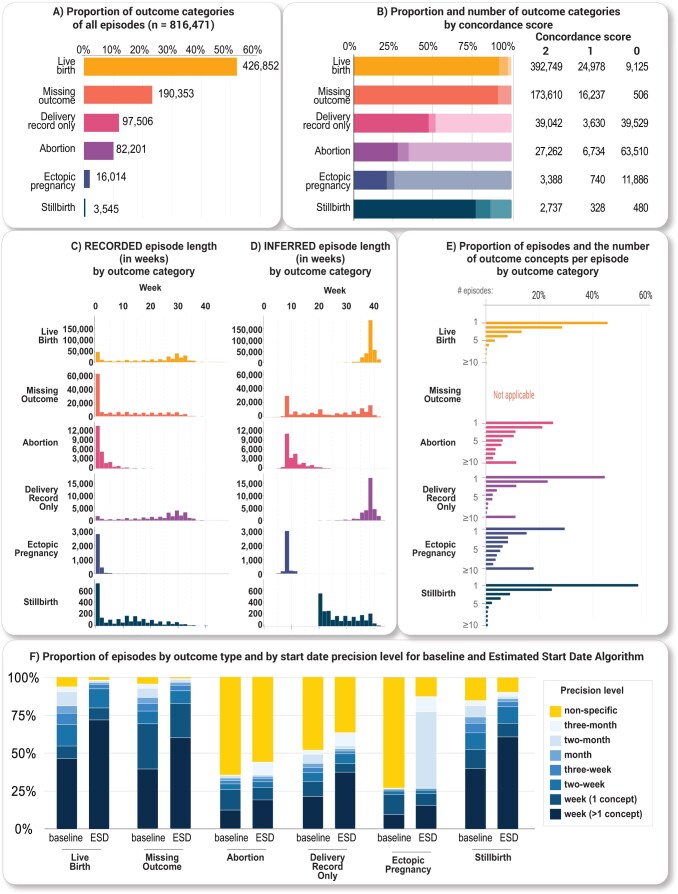
HIPPS results. (A) Histogram of the number of outcome concepts per episode by outcome category. (B) Outcome concordance scores by outcome category. An outcome concordance score of 2 has an outcome within the expected term duration and is supported by both HIP and PPS. An outcome concordance score of 1 has an outcome within the expected term duration. An outcome concordance score of 0 does not have an outcome within the expected term duration. (C) Histogram of episodes with week-level resolution only (*N* = 563 471) by outcome category of recorded pregnancy lengths (start and end dates of records for pregnancy that occur within the EHR data) in weeks and (D) inferred pregnancy lengths (pregnancy start and end estimated using HIPPS) in weeks. (E) Histogram of episodes with week-level resolution only (*N* = 563 471) by outcome category. Number of outcome concepts were determined from the outcome date to 28 days after. (F) Proportion of episodes by outcome category and by start date precision level for baseline and Estimated Start Date Algorithm. The baseline method obtained the start dates using only the week-level or GW concepts within an episode without any removal of outliers and assigned precision based on the maximum start date difference between GW concepts.

**Figure 6.**
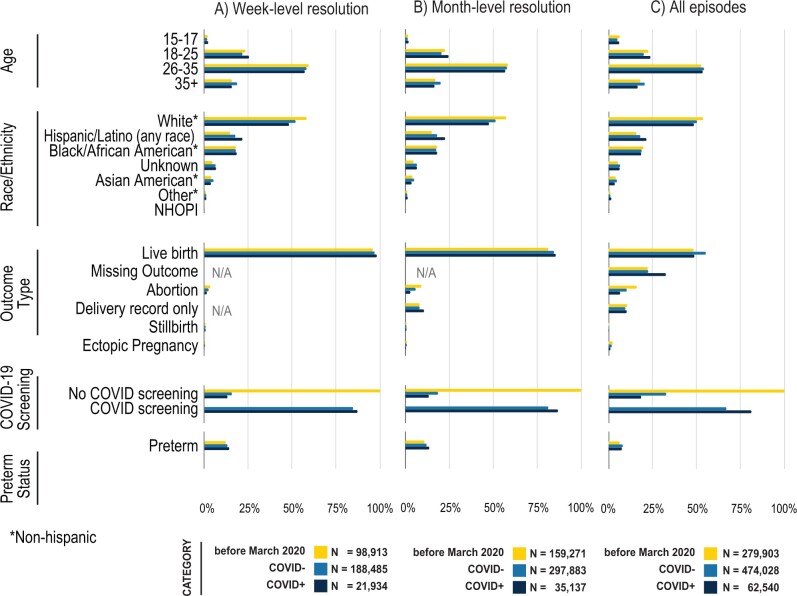
Demographics and outcomes of pregnant persons before and during COVID-19 pandemic, stratified by week-level resolution (A), month-level resolution (B), and all patients (C). See Table S7 for source data. Note that COVID negative (COVID-) includes pregnant persons without any results in their records.

To identify pregnancy episodes, we used 930 OMOP concepts including 74 concepts for inferring pregnancy start dates (Figure 2A). As of data extraction date (April 7, 2022), 72 unique data partner sites contributed data for >12 million total patients in N3C. Among these patients, HIPPS identified 4 million females of reproductive age (15–55 years), of whom a subset of 628K had at least one pregnancy episode recorded (Figure 2B). We identified 816 471 episodes of which 426 852 (52.3%) had live births, 3545 (0.4%) stillbirths, 97 506 (11.9%) abortions (both spontaneous and induced), 16 014 (2.0%) ectopic pregnancies, 82 201 (10.1%) with delivery record only, and 190 353 (23.3%) missing outcome (Figure 5A). Of patients with pregnancy episodes, 51.4% are White non-Hispanic, 17.7% are Hispanic or Latino any race, 19.1% Black/African American non-Hispanic, 4.3% Asian American non-Hispanic, 0.2% Native Hawaiian or Pacific Islander non-Hispanic, 6.2% unknown, and 1.1% other non-Hispanic (Figure 6).

In the sections below, we describe the main results of our analysis. For ease of presentation, we combined induced and spontaneous abortions under “Abortion.” In the Supplementary Materials, we provide additional details and data analysis that support each step of the HIPPS algorithm.

### Characterization of full set of episodes from HIPPS approach

Pregnancy episodes identified by our HIPPS approach found a significant proportion of likely ongoing pregnancies, had appropriate pregnancy lengths for respective outcome categories where the vast majority of episodes identified by HIP algorithm were also supported by PPS, and common outcomes had a high concordance score. Of the 190 353 (23.3%) of the episodes missing an outcome, 41 629 (21.9%) of these likely represent an ongoing pregnancy as these episodes had not reached the time range of plausible delivery outcomes, for example, for live birth or stillbirth (Figure 5A). Of the episodes first inferred by HIP, 724 251 (88.7%) episodes were supported by PPS. Of those not supported by PPS (92 220 episodes, 11.3%), 74 493 (80.8%) HIP episodes did not have any gestational information, and 81 151 (88.0%) episodes only had a recorded length of 1 day. When looking at the outcome concordance score, a metric of support for the identified outcomes, both live birth and stillbirth tended to have a higher percentage of episodes with the highest score of 2 (Figure 5B). Abortions and ectopic pregnancies on the other hand tended to have a higher percentage of episodes with the lowest score of 0 (Figure 5B) and a recorded episode length of 1 day (Figure 5C). Our algorithmically inferred pregnancy length corresponded well with the expected time intervals by outcome category (Figure 5D). Over 40.0% of episodes across all outcomes that occur after 20 weeks of gestation (live birth, stillbirth, delivery record only) only have one outcome concept within 28 days after the pregnancy outcome date (Figure 5E). Conversely, less than 30% of episodes for abortion and ectopic pregnancy contained only one outcome concept (Figure 5E).

### Pregnancy start date estimation and evaluation of precision

Using the GW and GR3m concepts for ESD, our algorithm yielded 475 433 (58.2%) episodes with week-level (>1 concept) resolution. Compared with a baseline method, our algorithm achieved a 55.5% increase in episodes with week-level precision and reduced the number of episodes for non-specific precision by 64.9% (Figure 5F, Table S4). Across all episodes with pregnancy outcomes, the proportion of episodes with week-level resolution increased (Figure 5F, Table S5).

### Clinician validation

Clinician validation agreed 98.8% with HIPPS-identified episodes (Cohen’s kappa coefficient estimated as 0.872 with asymptotic 95% CI, 0.828 to 0.915, exact *P*-value of test for rejecting the null of chance agreement: 1.38 × 10^−43^) (Table 1, Text S1M). The clinicians identified 7 extra episodes (4 with no outcome and 3 with abortions). Conversely, there were 3 episodes that were not classified as episodes by the clinicians (1 with no outcome, 1 ectopic pregnancy, and 1 abortion) and one non-pregnant person marked as having a pregnancy episode (no outcome) by the clinicians. Thus, agreement was assessed between 245 episodes considered by clinicians (as “gold standard”) with the 240 episodes identified by HIPPS and between 42 non-episodes from the clinicians with the randomly sampled 40 persons without episodes by our algorithm (Table 1).

**Table 1.** Clinician validation of episodes and outcome categories

	Number identified by algorithm	Number identified by clinician	Percent completeness of algorithm compared to clinician review^a^	Percent agreement between algorithm and clinician^b^	**Clinicians’ average confidence score** **(**−**1, 0, 1)**	Standard deviation of clinicians’ confidence scores
**Number of non-episodes** **(among 40 females)**	40	42	92.9	97.5	N/A	N/A
**Number of episodes** **(among 166 females)**	240	245	88.6	90.4	N/A	N/A
**Outcome**						
Live birth	80	89	89.9	100.0	0.96	0.19
Stillbirth	40	38	100.0	95.0	0.97	0.16
Abortion	40	47	78.7	92.5	1	0
Ectopic pregnancy	40	33	100.0	82.5	0.85	0.44
No outcome	40	38	76.3	72.5	N/A	N/A
**Gestational age at outcome (±1 week)**						
Live birth	N/A	N/A		98.8	0.71	0.46
Stillbirth	N/A	N/A		89.5	0.57	0.55
Abortion	N/A	N/A		67.6	−0.34	0.61
Ectopic pregnancy	N/A	N/A		63.6	−0.58	0.50

As these cases were randomly selected for clinician validation, they do not reveal information about the subpopulation and are not subject to the same policy regarding masking small numbers of patients.

a Percent completeness of algorithm compared to clinician review is the number of matching algorithmically defined episodes out of all possible clinician-defined episodes for these categories.

b Percent agreement between algorithm and clinician is the number of matching clinician-defined episodes out of all possible algorithmically defined episodes for these categories.

The percent agreement by outcome category, including no outcome, was over 70.0%, and the average confidence score ranged from 0.85 to 1.00. Within 1 week, HIPPS had high agreement with clinicians’ validations: 85.2% to 96.4% for inferred start dates, 90.9% to 100.0% for inferred end dates, and 63.6% to 98.8% for gestational age (Tables 1 and 2). These average confidence scores tended to be higher for live birth versus other outcomes (Tables 1 and 2). Lastly, interrater agreement ranged from 88.2% to 100% per metric (see Text S1M for more details).

**Table 2.** Clinician validation of start and end dates of both inferred and recorded pregnancy episodes

		Algorithmic concordance (% episodes) with clinician-assigned date ± buffer			**Clinicians’ average confidence score (**−**1, 0, 1)**	Standard deviation of clinicians’ confidence scores
		±7 days	±14 days	±21 days		
**Inferred start date of inferred pregnancy episode^a^**						
**Outcome**	Live birth	93.8	98.8	100.0	0.56	0.52
	Stillbirth	86.8	92.1	94.7	0.42	0.60
	Abortion	85.2	92.6	96.3	0.04	0.84
	Ectopic pregnancy	87.5	91.7	91.7	−0.04	0.89
**No outcome**		96.4	96.4	96.4	0.61	0.57
**Inferred end date of inferred pregnancy episode (outcome only)**						
**Outcome**	Live birth	97.5	98.8	100.0	0.80	0.40
	Stillbirth	100.0	100.0	100.0	0.61	0.55
	Abortion	91.9	94.6	94.6	0.42	0.50
	Ectopic pregnancy	90.9	90.9	90.9	0.48	0.62
**Start date of recorded pregnancy episode**						
**Outcome**	Live birth	68.8	71.3	77.5	0.84	0.40
	Stillbirth	68.4	78.9	84.2	0.79	0.41
	Abortion	73.0	78.4	81.1	0.78	0.48
	Ectopic pregnancy	97.0	100.0	100.0	0.88	0.33
**No outcome**		65.5	69.0	72.4	0.83	0.385
**End date of recorded pregnancy episode**						
**Outcome**	Live birth	91.3	93.8	97.5	0.79	0.41
	Stillbirth	84.2	86.8	89.5	0.68	0.57
	Abortion	94.6	97.3	97.3	0.54	0.56
	Ectopic pregnancy	78.8	81.8	93.9	0.52	0.62
**No outcome**		75.9	82.8	82.8	0.72	0.45

As these cases were randomly selected for clinician validation, they do not reveal information about the subpopulation and are not subject to the same policy regarding masking small numbers of patients.

a Not all episodes have ascertainable start dates.

### Assessment of pregnancy episode detection and gestational timing validation using alternative pregnancy-specific gestational timing concepts (external to HIPPS)

Similarly, our additional validation analysis to check the robustness of HIPPS to detect pregnancy episodes using 25 clinician-curated concepts independent to our algorithm definition and known to occur specifically *within* pregnancy resulted in a mean of 90.2 (SD = 5.4)% overlap with HIPPS-identified episodes (Table S6).

### Characterization of the various pregnancy cohorts stratified by COVID-19 status

Among the approximate 816 471 pregnancy episodes (cohort 1: all episodes), 492 291 (60.3%) had month-level granularity for gestational start date (cohort 2: month-level) and 309 332 (37.9%) had week-level granularity for gestational start date (cohort 3: week-level) (Figure 6, Table S7). Comparing across cohorts, patient characteristics were fairly similar with a few exceptions: patients in cohort 3 (week-level) were less likely to be 15–17 years old, were more likely to have a defined outcome of live birth, and more likely to have COVID-19 screening during pregnancy (Figure 6). There were minimal differences in patient characteristics before versus after March 2020 (Figure 6). Demographics of our cohort were similar to national pregnancy statistics (Tables S9 and S10).

For episodes post-March 2020, we further stratified by COVID-19 positivity during pregnancy; COVID-19-positive pregnant persons were more likely to be Hispanic (21.3% vs 18.2%), more likely to be 18–25 years old and less likely to be 35+ years old. Those with a positive test or diagnosis were more likely to be screened for COVID-19 than those without (81.3% vs 63.3%, Table S7). These trends were similar for cohorts 2 and 3, however, missing data were less frequent among these cohorts compared to cohort 1, possibly indicating a more complete EHR record.

## Discussion

We have created the largest US cohort to date of COVID-19 positive pregnant persons. In this geographically diverse and historically unprecedented dataset, we identified 816 471 pregnancy episodes, with the majority of episodes having week-level resolution for pregnancy start. The completeness of race/ethnicity data in our cohort (6% missingness) is a strength of this data source. Our comprehensive, novel resource enables precise inference of pregnancy episodes in N3C and is a strong foundation for researchers looking to address knowledge gaps regarding pregnancy.

Our composite algorithm, HIPPS, combines 2 independent algorithms, each developed to address the disjointed or noncontinuous EHR records of a pregnant person: one algorithm, HIP, is an extension of a previously-developed rule-based algorithm,^12^ and the other is PPS, an entirely novel algorithm based on the longest increasing consecutive subsequence analysis. We used PPS to validate episodes from HIP. HIPPS offers unique elements not currently covered by existing, published algorithms. First, we followed a prior algorithm that only considered pregnancies with an outcome (eg, live birth), omitting pregnancies without outcomes.^12^ As such pregnancies still offer important data, including for COVID-19, our HIP and PPS algorithms were designed to capture pregnancies with and without outcomes, the former by employing “Gestation period, X weeks” concepts as attempted by Naleway et al^15^ and analogous to the ICD-10-CM Z3A codes used in Bertoia et al.^20^ Second, PPS checks if the gestational timing data is progressing, even without outcome anchoring, and finds 1.7-fold longer episodes compared to HIP, highlighting the value of adding this approach to existing episode length inferences. Third, our HIPPS approach is consistent with how clinicians would annotate EHR information, as demonstrated by our clinician validation. Fourth, high concordance between HIP and PPS provides high confidence in our pregnancy episode and outcome inference. Fifth, combining empirical analysis and clinician validation to derive gestational timing concepts for PPS and ESD allows episode inference to be optimized to the EHR dataset available. This is an advantage over using an inflexible set of concepts of previous algorithms^12^^,^^15^ for inferring pregnancy episode timing that may not be generalizable to other EHR datasets.

Our final novel sub-algorithm applied to the episodes, ESD, rigorously estimates pregnancy start date or LMP; this is ever more important in ascertaining risks related to exposures in pregnancy and outcomes. By including a validation metric, precision in days, for ESD, we provide a high level of confidence for the estimated LMP, with over 58.2% and 83.2% episodes with week- and month-level resolution, respectively. Such precision in gestational age allows researchers to determine associations with an unprecedented level of confidence.

Finally, as proof-of-concept of application of HIPPS to N3C data, we generated 3 types of cohorts of pregnant persons, ranging from all episodes regardless of precision in gestational age (cohort 1) to a cohort with week-level precision for gestational age and defined outcome (cohort 3), and compared sociodemographic, clinical, and COVID-19 variables across the cohorts. Our cohorts were similar (age, race/ethnicity) to national statistics on live births (2019–2021) and CDC surveillance data on COVID-19 infection during pregnancy,^30^^,^^31^ demonstrating our results are consistent with national demographics. Furthermore, the distribution of gestational ages for live birth are similarly asymmetrical to the national distribution with a mode of 39 weeks.^32^ While we generally did not observe many significant differences in the proportion of pregnant persons across the 3 cohorts, some interesting differences arose. For example, those in cohort 3 are less likely to be teenaged, more likely to have live birth outcomes, and more likely to have been screened for COVID-19 than those in our other 2 cohorts. That this cohort type is less likely to have abortion or ectopic pregnancy is only natural given these outcomes have less gestational info. Thus, for example, questions anchoring on vaccination timing during pregnancy on risk of breakthrough or incident COVID-19 infection may require week-level granularity for estimated start date. Evaluating abortions as the outcome would be a likely mismatch. On the other hand, certain research questions may not require exact gestational age, as is common with claims data when researchers assign 40 weeks gestation to classify exposure where gestational info is missing, in which case selecting cohort 1 may be most appropriate. Our presentation of 3 possible cohorts, to elucidate the possible sizes, demographics, precision, and tradeoffs for using exact definition types to define pregnancy cohorts in N3C, should aid future researchers for various research efforts both within and outside of N3C.

While our work has several unique strengths, limitations also exist. First, we are unable to conduct source validation. Due to the nature of N3C not allowing reidentification, we cannot conduct a “gold standard” validation of our findings against source data using birth certificates or medical charts; thus, our work, in principle, cannot ascertain accuracy. Though we attempted a clinician validation approach that mimicked a medical chart review, it is possible that both the clinician annotators and HIPPS misidentified a pregnancy episode or misclassified a pregnancy outcome. Regardless, previous literature supports our approach for defining pregnancy episodes even without source data validation,^12^ and our proportions of abortion, stillbirth, and ectopic pregnancy were roughly similar to the national proportions described in the literature.^33–35^ Second, as with any EHR dataset, the likelihood of missingness and misclassification is high. It is possible that we may have missed potential pregnancy episodes or outcomes with our current concept sets, although we found inferred episode overlap of 90.2% on average between the 25 gestational age-specific concepts independent of those used for algorithm definition. This suggests that concept bias, either in our overall algorithms or in the small numbers included in our clinician validation, is unlikely. Also, because we did not filter out any episodes with a single occurrence of an outcome concept, as Matcho et al^12^ had done, it is possible that our dataset overestimates the presence of some outcomes. Relatedly, misclassification may be high, especially for uncommon pregnancy outcomes, which could create sources of bias. Nonetheless, since we do provide the number of outcome concepts and confidence score for each pregnancy episode, users of the data and our approach can individualize criteria for excluding episodes depending on their research question. Third, while our approach has high certainty using the outcome concordance score as a metric of support for identifying common pregnancy outcomes, such as live birth, we observed less certainty in inferences related to less common outcomes (ie, ectopic pregnancies) or underreported outcomes (ie, spontaneous abortions), which are recurrent issues among all EHR-based phenotyping of such outcomes.

To make HIPPS generalizable to other datasets beyond N3C, we provide code (written in PySpark) and instructions in our GitHub repository, https://github.com/jonessarae/n3c_pregnancy_cohort, for interested users to adapt and apply our approach. Our algorithm can be applied to any observational data source which is either already in OMOP or has been converted to OMOP. Additionally, the N3C has scripts to convert other CDMs (ie, PCORnet) to OMOP,^36^ and the Simple Standard for Sharing Ontological Mappings (SSSOM) method^37^^,^^38^ can be applied to convert other CDMs not processed by N3C to OMOP.

## Conclusion

Using HIPPS to identify pregnancy episodes and outcomes, we have not only developed the largest cohort to date of COVID-19 positive pregnant persons from the United States but have enabled gestational age with high precision—crucial to research attempting to ascertain associations between certain pregnancy-related exposures and outcomes, elements that are ever-more important in COVID-19-related research in pregnancy. Our novel approach to gestational age has enduring implications for others to precisely infer pregnancy episodes in EHR data.

## Ethics statement

Data partner sites transfer their N3C-eligible data to NCATS/NIH under a Johns Hopkins University Reliance Protocol (IRB00249128) or via individual site agreements with NCATS (see below). Managed under the NIH authority, the N3C Data Enclave can be accessed as previously described^10^ and at ncats.nih.gov/n3c/resources, https://covid.cd2h.org/for-researchers.

**Table:** 

Site	IRB name	Exempted vs approved	Protocol number
Medical University of South Carolina	Health Sciences South Carolina Institutional Review Board	Exempt	Pro00111335
National Institutes of Health	NIH Office of IRB Operations	Exempt	N/A
University of Minnesota	University of Minnesota Institutional Review Board	Approved	STUDY00012706
University of Rochester	University of Rochester Research Subjects Review Board	Exempt	STUDY00005366
University of Washington	Human Subjects Division	Approved	STUDY00013147

## Code availability

All analyses were conducted in the N3C Enclave using R (v.3.5.1), Python (v.3.6.10), and PySpark (v.3.0.2). To support reproducibility, the code for implementing HIPPS, and full package list with versions, is available at https://github.com/jonessarae/n3c_pregnancy_cohort; it is also available to users with valid login credentials in the N3C enclave.

## Disclaimer

The content is solely the responsibility of the authors and does not necessarily represent the official views of the NIH.

Individual authors were supported by the following funding sources: NIMH R01131542 (RCP). The funding sources or study sponsors had no role in study design; in the collection, analysis, and interpretation of data; in the writing of the report; and in the decision to submit the paper for publication. S.E.J. was supported by an appointment to the National Institute of Allergy and Infectious Diseases (NIAID) Emerging Leaders in Data Science Research Participation Program. This program is administered by the Oak Ridge Institute for Science and Education through an interagency agreement between the US Department of Energy (DOE) and NIAID. ORISE is managed by ORAU under DOE contract number DE-SC0014664. All opinions expressed in this paper are the authors’ and do not necessarily reflect the policies and views of NIAID, DOE, or ORAU/ORISE.

## Supplementary Material

ooad067_Supplementary_Data

## Data Availability

The N3C data transfer to NCATS is performed under a Johns Hopkins University Reliance Protocol # IRB00249128 or individual site agreements with NIH. The N3C Data Enclave is managed under the authority of the NIH; information can be found at ncats.nih.gov/n3c/resources. Enclave data are protected, and can be accessed for COVID-related research with an NIH-approved approved (1) IRB protocol and (2) institutional Data Use Request (DUR). A detailed accounting of data protections and access tiers is found at https://ncats.nih.gov/n3c/resources/data-access. Enclave and data access instructions can be found at https://covid.cd2h.org/for-researchers; all code used to produce the analyses in this manuscript is available within the N3C Enclave to users with valid login credentials to support reproducibility.

## Notes

**Author Contributions:** Sara E. Jones and Dr Katie R. Bradwell contributed equally and are considered as first authors of this work. Drs Rena C. Patel and Elaine L. Hill contributed equally and are considered as senior authors of this work.
