# Supplementary material for: Who is pregnant? Defining real-world data-based pregnancy episodes in the National COVID Cohort Collaborative (N3C)
Source: JAMIA Open. 2023 Aug 16;6(3):ooad067. doi: 10.1093/jamiaopen/ooad067 (PMC10432357; doi:10.1093/jamiaopen/ooad067)
Supplement: ooad067_Supplementary_Data [file ooad067_supplementary_data.pdf]

Supplementary Material for “Who is pregnant? defining real-world data-based pregnancy episodes in the National COVID Cohort Collaborative (N3C)”

Table of Contents

Supplementary Methods **1**

A. Detailed information on National COVID Cohort Collaboration (N3C) 1

B. Data quality assessment and identifying pregnancy-specific concepts 2

C. Modifications to the Pregnancy Episode Algorithm from Matcho et al. 2

D. Detailed steps for Hierarchy-based Inference of Pregnancy (HIP) Algorithm 3

E. Detailed steps for Pregnancy Progression Signature (PPS) Algorithm 5

F. Detailed explanation of Estimated Start Date (ESD) Algorithm 6

G. Pseudocode for Estimated Start Date (ESD) Algorithm 7

H. Detailed methods for clinician validation 10

Supplementary Results **12**

I. Data availability analysis 12

J. Gestational timing concept curation and exploration 12

K. Performance of HIPPS and HIPPS component algorithms to baseline 12

L. Analysis of PPS contribution to HIPPS 12

M. Clinician validation substudy - additional details for interrater agreement findings 13

N. Comparison to national sample of live births and pregnancies with COVID-status 13

Supplementary Tables **15**

Supplementary Figures **117**

References **125**

Supplementary Methods

A. Detailed information on National COVID Cohort Collaboration (N3C)

The N3C, which is overseen by NCATS, systematically and regularly collects data derived from the electronic health records of people who were tested for COVID-19 or who had related symptoms, as well as data from individuals infected with pathogens that can support comparative studies, such as SARS 1, MERS and H1N1. This data source includes such information as demographics, symptoms, lab test results, procedures, medications, medical conditions, physical measurements and more. The N3C utilizes the OMOP Common Data Model for standardization of clinical structure and content across all databases. All content (conditions, procedures, measurements, drugs and observations) in the OMOP Common Data Model are referred to as concepts. Native source codes are mapped to the dictionary that is considered standard for that domain (conditions, procedures, etc.) in the Standard Vocabularies. For example, the International Classification of Diseases, Tenth Revision, Clinical Modification (ICD-10-CM) codes are mapped to Systematized Nomenclature of Medicine—Clinical Terms (SNOMED-CT)

concepts. Institutions contributing data work with NCATS to transfer a limited data set relevant to COVID-19 in the institution's preferred Common Data Model (derived from electronic health records) to the N3C Data Enclave on a recurring basis. This limited dataset leaves out 16 types of direct identifying information about the patient and their relatives, employers, or household members — such as names, account numbers, telephone numbers, email addresses and social security numbers. ZIP codes and dates of service are included in the limited dataset because these are critical for tracking the progress of the pandemic over time and place. The N3C data harmonization team ingests the limited data set, runs quality checks and transforms different data models into a harmonized OMOP (OMOP version 5.3.1) analytics data set. Currently, the four common data models utilized by the participating sites include ACT, OMOP, PCORnet, and TriNetX. The data, which are represented at the individual patient level, are hosted within the N3C Enclave is Palantir Foundry, a data science platform enabling complex and reproducible analysis using standard open-source, analytical packages in languages such as Python, R, SQL, and Java, as well as point-and-click and dashboard-style analytical tools. Additional information on N3C can be found at https://ncats.nih.gov/n3c and https://covid.cd2h.org/dashboard/.

B. Data quality assessment and identifying pregnancy-specific concepts

We assessed the availability and data quality of concepts related to gestational age and other pregnancy-related information in N3C. In addition to "Gestation period, X weeks", we explored other concepts such as "Last menstrual period start date" and "Delivery date Estimated" that could be utilized for gestational age (see variable "Gestational age, other" for complete list of concepts in Table S1). We evaluated the missingness of these concepts as well as their values in N3C and by data partner. To find potentially pregnant persons in N3C, we applied concepts pertaining to pregnancy outcomes and radiography (e.g., ultrasonography) from Matcho et al.'s algorithm [1]. All concepts from Matcho et al.'s algorithm were updated to reflect the most recent OMOP vocabulary within N3C. Concepts used for females with pregnancy-related concepts in their record ("pregnant cases") were searched for in other patients ("non-pregnant controls") in order to find concepts specific to pregnant persons. We included concepts related to measurements, observations, conditions, drugs, and procedures. Concept frequency within the pregnant reference group was determined in order to help rank concepts common to pregnant persons. A minimum frequency of usage in at least 1000 distinct profiles within the pregnant reference set was used to filter out concepts with low frequency. Of these concepts, we kept those with 10 times greater frequency among possibly pregnant persons vs. controls (all other patients in N3C).

C. Modifications to the Pregnancy Episode Algorithm from Matcho et al.

We updated and expanded the pregnancy outcomes from Matcho et al. using Athena [2] and Atlas [3] as Matcho et al. only categorized concepts with 100 patients (1,702 concepts) out of 3,081 concepts in their S1 Table [1]. We removed any concepts that were not specific to a pregnancy outcome (i.e. molar AND/OR ectopic pregnancy). For each outcome category, we started with the first record for each patient and assessed if any subsequent record occurred after the necessary time interval in the S3 Table from Matcho et al. [1] to be considered a new episode or pregnancy. The process of checking if each record was outside the time window for that

particular outcome category was repeated for all consecutive records following any record indicated as a distinct pregnancy. We kept episodes with only one instance of an outcome concept (as we did not want to miss any potential pregnancies given the EHR nature of our data source commonly having only a delivery visit, for example) vs. at least two in Matcho et al. for all outcomes other than live birth [1].

For abortion and ectopic pregnancy outcomes, outcome dates were not reassigned to the last date in a two week period following either outcome category as done in Matcho et al. [1]. The purpose of keeping the first occurrence of outcome dates was for determining gestational age in weeks as accurately as possible. In addition, we did not apply the requirement that an ectopic pregnancy outcome must have a methotrexate exposure or an ectopic pregnancy-related procedure during the 14 day period after the ectopic pregnancy record date since we wanted to capture all possible ectopic pregnancies. For every outcome other than live birth, we did not assess for any antenatal visit or pregnancy confirmation record found during the 42 day period following the outcome date as the concepts may not appear in N3C. Instead we used the gestation-based episodes to help identify any invalid outcomes.

Following the hierarchy as described in Matcho et al. [1], each outcome category was then combined in this order: live birth, stillbirth, ectopic pregnancy, abortion, and delivery record only. We also grouped spontaneous and induced abortions under just one category, abortions like Matcho et al. [1]. With every outcome category added in the order listed above, any outcome record of that category was compared to all records of the previous outcome categories and was kept if it occurred outside the required windows of time between outcome categories.

Since many of the pregnancy start markers from Matcho et al. were not represented in N3C and would require extensive concept vetting within the N3C vocabulary to ensure the concept sets in N3C were as representative and comprehensive for each pregnancy start marker category defined in Matcho et al., we only updated the "gestational age indicated" concepts [1]. These concepts were the most prevalent within our data (See Supplementary Materials, Section B. "Data availability analysis and identifying pregnancy-specific concepts") and could provide gestational age with week-level resolution unlike the other pregnancy start marker categories.

After merging gestation-based episodes with outcome-based episodes, we checked if the estimated gestational age in weeks for each episode defined by an outcome was clinically possible using the S4 table from Matcho et al. [1]. We modified the minimum pregnancy timeline of stillbirths to 20 weeks or 140 days to align with the CDC definition of stillbirths [4]. If the estimated gestational age in weeks for the outcome was not found to be clinically possible, the outcome was removed.

D. Detailed steps for Hierarchy-based Inference of Pregnancy (HIP) Algorithm

PREG - pregnancy without outcome
DELIV - delivery record only
LB - live births
SB - stillbirths

ECT - ectopic pregnancies
AB/SA - spontaneous and induced abortions

Determining outcome-based episodes

1. Get all outcome-based records in order of occurrence (DELIV, LB, SB, ECT, AB/SA).
2. For records of the same outcome category, calculate the number of days between each record and keep the earliest record and any other record if it occurred outside the required window of time between records.
3. Construct independent pregnancy episodes following the hierarchy: LB, SB, ECT, AB/SA, and DELIV. Starting with LB, every outcome further down the hierarchy is assessed and kept as an independent episode if it meets the minimum allowable time with every other outcome already determined to be an independent episode.
4. Calculate the minimum and maximum start dates for each pregnancy episode based on the outcome's minimum and maximum term length.

Determining gestation-based episodes

1. Get all gestation-based records in order of occurrence and copy any values from the measurement domain table and set as gestational age in weeks. Keep only any records with a gestational age in weeks > 0 and <= 44 to remove any strange values.
2. If there are two or more gestational records with the same date, the record with the max gestational age in weeks is kept.
3. Calculate the difference in gestational age in weeks between records as well as the difference in days between record dates.
4. Any record with a negative change or no change in the gestational age in weeks from the previous record is flagged as the start of a potential episode. This record is then checked if there is at least a separation of 70 days from the previous record. The number of days, 70, was determined by taking the minimum outcome limit in days, 56, and adding a buffer of 14 days. If the record is not at least 70 days from the previous record, it is no longer flagged as the start of an episode.
5. For all records with a positive change in the gestational age in weeks from the previous record is then checked if the date difference in days between records is greater than the difference in days between the record's gestational age in weeks and the previous record's gestational age in weeks with a buffer of 28 days. The buffer of 28 days was determined by taking the minimum retry period in days, 14, and adding 14 days as a buffer. If the date difference in days is greater than the difference in days between the record's gestational age in weeks and the previous record's gestational age in weeks with the buffer, then this record is flagged as a start of a new episode.
6. Get the minimum and maximum gestational age in weeks and the corresponding visit dates per pregnancy episode. Also get the first and last visits and their corresponding gestational age in weeks per pregnancy episode.

7. All gestation-based episodes are classified as PREG.

Combine gestation-based and outcome-based episodes and clean up episodes

1. Calculate the start date by subtracting the maximum gestational age in weeks from the date that the maximum gestational age in weeks occurs in the gestation-based episode. The date for maximum gestational age in weeks serves as the end date for the episode.
2. If any gestation-based episode overlaps with an outcome-based episode using the maximum start date and the outcome date as the start and end of the episode respectively, it is removed as a distinct episode. All information related to the gestation-based episode is incorporated into the outcome-based episode.
3. Check for any gestation-based episodes that overlap with more than one outcome-based episode and keep only those episodes where the gestation-based end date is closest to the outcome date.
4. Estimate gestational age in days by calculating the difference in days between the outcome date and the start date from the gestation-based episode.
5. Reclassify any outcome-based episodes as PREG if the gestational age in days is either under the minimum term length or over the maximum term length.
6. Reclassify any outcome-based episodes as PREG if the gestation-based episode end date occurs after a buffer of 28 days from the outcome date.
7. Recalculate the gestational age in days using the gestation-based episode end date for any reclassified outcome-based episodes.
8. Identify any episodes that overlap by checking if the difference in days between the start date and the previous episode's end date is negative. If negative, then the previous episode overlaps the following episode. Only remove the previous episode if the category is PREG.
9. Calculate the new estimated start date if the latter episode has overlap with the previous episode by adding the number of days of the previous episode's retry period to the previous episode's end date.
10. If there are any remaining episodes with gestational age in weeks at outcome date not within the term durations, reclassify as PREG.

E. Detailed steps for Pregnancy Progression Signature (PPS) Algorithm

Identifying gestational age concepts

1. Obtain an initial cohort of women with evidence of pregnancy.
2. Use the women from step 1 vs all other patients to determine which concepts to obtain a specificity metric per concept, and apply a cut-off of 10X to filter to the most "pregnancy specific" concepts.
3. Using the pregnancy specific concepts from step 2, determine the mean and standard deviation of when the concepts occur relative to pregnancy start across episodes of each

pregnancy outcome category from HIP algorithm. Filter out any concept that has a standard deviation of > 2 for any outcome category. Also take the standard deviation across all mean values from pregnancy start date for all outcome categories, and filter out any concept where the standard deviation of the mean > 1.5 (i.e. removing concepts with dissimilar gestational timing across different pregnancy outcomes)

4. Provide the concepts resulting from step 3 to clinicians for manual curation and addition of expected time ranges for occurrence during pregnancy. Filter out any concepts where the gestational timing of a concept spanned > 3 months
5. Save the concepts resulting from step 4 to a lookup table of concepts and their corresponding minimum and maximum month.

Determining episodes with clinician-curated gestational age concepts

1. Identify all the patients that have these concepts and the dates of when these concepts occur in their EHR data, for each concept appending the min and max expected months from the concept lookup. Within each patient, sort the list of {concepts, actual dates and expected min, max months} elements by actual date.
2. Iterate through each patient, and within each patient loop through the gestational timing concept record dates in chronological order, comparing whether the actual difference in dates is within minimum and maximum plausible (based on min, max months) differences in dates across the two concepts. Make these comparisons both with each current concept, i, and all previous concepts, and between each successive pair of concepts surrounding and outward from i.
3. The result of the comparisons from step 2 will be either i) at least one comparison returns True (that the actual time elapsed between the patient's concepts matches what we'd expect given the expected maximum and minimum months for the concepts under comparison), or ii) all comparisons yield False. For i) we presume that this is due to a continuing pregnancy, and thus continue the same episode number as the previous patient record. In the case of ii) we perform an additional check to see if the difference in actual dates between i and i-1 is > 2 i.e. greater than the minimum permissible retry period [1], and if so, we assume that a new pregnancy has started and we begin a new episode number.
4. The episodes from step 3 then undergo further filtering to remove any that are of implausible length (> 12 months), followed by renumbering of all episode numbers in cases where episodes are removed.
5. Patients with episodes are then filtered out under the following circumstances: i) male gender (most likely newborns or miscoding in the EHR of either gender or concepts) or ii) the patient was not of child-bearing age (designated as 15-55 years old in this study), or iii) had > 5 episodes in any one year

F. Detailed explanation of Estimated Start Date (ESD) Algorithm

First, for all gestational week-level resolution (GW) concepts we calculated the pregnancy start date by subtracting the plausible gestational age in weeks from the date the concept occurred. For concepts with resolution between one-week and three months (GR3m), the range for the earliest and latest possible start date was obtained. Outlier GR3m concepts were removed based on 1.5*IQR of the number of overlaps to other GR3m concepts. If the majority of GW concepts within an episode were contained within the intersection of the GR3m start date ranges, we filtered to those GW concepts within the GR3m intersection. Next, out of those remaining GW concepts, we removed additional outliers using 1.5*IQR of the GW concept start dates. We then took the GW concept that occurred latest in the pregnancy episode as the pregnancy start date, as the start dates of GW concepts occurring later in pregnancy have greater precision. If an episode did not contain any GW concepts, then the midpoint of the intersection of the GR3m concepts was set as the estimated start date. Where GW concepts lacked any GR3m range overlap or where GR3m concepts were missing, we removed outliers from the full set of GW concepts using 1.5*IQR and took the GW concept that occurs latest in pregnancy to extrapolate the pregnancy start date.

In addition to estimating the pregnancy start date, we assigned a level of precision to further adjudicate the use of the calculated gestational age in weeks against the estimated pregnancy start date. In cases where GW concepts were used to estimate the start date, this was the difference between the maximum and minimum start date, in days, out of concepts following the 1.5*IQR filtering step. Otherwise, we calculated precision using the union in days of the GR3m concepts that overlap after removal of any GR3m outliers. If GW or GR3m concepts are not available, we calculated the maximum plausible outcome-specific gestational age in days to assign a start date and the duration of the minimum and maximum outcome-specific gestational age in days to compute the precision. We then assigned precision categories based on precision in days: “week_poor-support”, precision ≤ 7 days but a single GW concept was used to extrapolate back to a pregnancy start date; “week”, precision ≤ 7 days; “two-week”, 7 days < precision ≤ 14 days; “three-week”, 14 days < precision ≤ 21 days; “month”, 21 days < precision ≤ 28 days; “two-months”, 28 days < precision ≤ 56 days; “three-months”, 56 days < precision ≤ 84 days; “non-specific”, no timing information.

G. Pseudocode for Estimated Start Date (ESD) Algorithm

<u>Definitions</u>

GW: Gestational week concept (Gestational age estimated, Gestational age, Gestational age in weeks, and Gestation period X weeks)
GR3m: Gestational timing concepts of up to three months and > one week (clinician curated)
START_DATE: start date of pregnancy working back from gestational timing concepts
PRECISION: precision in days (0 means non-specific gestational timing) based on the max discrepancy between relevant concepts
Xd = precision in terms of difference in days predicted from the concepts used to estimate start date of pregnancy

Filter definitions

- FILTER#1: use the 1.5*IQR rule to remove outlier GR3m concepts in terms of number of overlaps to other GR3m concept ranges, then obtain the intersection (overlap) in pregnancy start date for the remaining GR3m concepts as a range
- FILTER#2: if more than one GW concept is found, use the 1.5*IQR rule to remove outlier concepts then keep the max GW (the GW concept that occurs latest in pregnancy)

Pseudocode

Start by performing the following two steps:
(1) in cases where distinct GW concepts occur on the same date, keep the maximal GW value (since previous values are likely to be historical references)
(2) extrapolate the start date from the GW concepts and the potential min and max start dates from each GR3m concept.

Next, process gestational timing information to derive the pregnancy start date, precision in days, and precision category via the logic below.

```
If the episode contains GW concepts:
    if the episode also contains valid GR3m concepts:
        Apply FILTER#1
        check the percent of GW concepts that agree with the GR3m overlap
        if >= 50.0% agreement:
            START_DATE = use the GW concepts that agree to apply FILTER#2
            PRECISION = the max difference between those concepts that agree and are not outliers
            return (START_DATE, PRECISION=Xd)
        if < 50.0% agreement:
            if the episode has > 1 GW concepts:
                START_DATE = apply FILTER#2 for the GW concepts
                PRECISION = the max difference in days between those non-outlier concepts
                return (START_DATE, PRECISION=Xd)
            else:
                START_DATE = derived from the single GW concept
                return (START_DATE, PRECISION=-1)
    if the episode does not contain GR3m concepts:
        if the episode has > 1 GW concepts:
            START_DATE = apply FILTER#2 for the GW concepts
            PRECISION = the max difference in days between those non-outlier concepts
            return (START_DATE, PRECISION=Xd)
        else:
            START_DATE = the start date from single GW concept
            return (START_DATE, PRECISION=-1)

if the episode contains 0 GW concepts and >= 1 GR3m concept:
    Apply FILTER#1
    START_DATE = midpoint of the above range
    PRECISION= union (overlap, where applicable) in pregnancy start date for the GR3m concept(s) in days
    return (START_DATE, PRECISION=Xd)

if the episode contains 0 GW concepts and 0 GR3m concepts (episodes containing outcomes without timing info):
    START_DATE = subtract the max term duration in days plausible for outcome from the pregnancy end date
    PRECISION = difference in max and min term duration in days plausible for outcome
```

```
        return (START_DATE, PRECISION=Xd)


Add in an PRECISION_CATEGORY column:
        If PRECISION == -1:
                PRECISION_CATEGORY = 'week_poor-support'
        if 0 =< PRECISION <= 7:
                PRECISION_CATEGORY = 'week'
        if 7 < PRECISION <= 14:
                PRECISION_CATEGORY = 'two-week'
        if 14 < PRECISION <= 21:
                PRECISION_CATEGORY = 'three-week'
        if 21 < PRECISION <= 28:
                PRECISION_CATEGORY = 'month'
        if 28 < PRECISION <= 56:
                PRECISION_CATEGORY = 'two-month'
        if 56 < PRECISION <= 84:
                PRECISION_CATEGORY = 'three-month'
        else:
                PRECISION_CATEGORY = 'non-specific'
```

H. Detailed methods for clinician validation

As mentioned in the main manuscript, manual chart review is not possible in N3C due to regulations that minimize reidentification risk; thus, we utilized clinician annotation of algorithm-identified records to validate the processes by which we obtain key inferences. A two-staged sample of EHR-based records (event sequences with affiliated dates) were randomly selected from among those assessed by HIPPS and then reviewed independently by three clinicians trained in obstetrics and gynecology. Presence of date-anchored pregnancy episodes, including attributes such as gestational timing and any accompanying outcomes, were determined by these clinicians. The initial stage (an internal pilot study) was conducted to yield a realistic range for our chosen set of metrics, namely percent agreement and chance-corrected measures of agreement, for evaluating classifications between our HIPPS algorithm and clinician annotation among the validation substudy sample. Preliminary estimates of near-complete agreement from the internal pilot, considered in tandem with comparable substudy validation designs in the literature (clinicians-alongside-algorithmic determinations, e.g., in determining mode of delivery from EHRs), helped to set lower bounds for minimally-detectable measures. For example, the internal pilot indicated Cohen's kappa values of 0.76 to guide as either an alternative value relative to comparable values from the literature (horizontal pink line in Figure S2A, relative to 0.45 to 0.7 range reported in relevant literature [5,6] or as a referent null value to "rule out" via having its 95% confidence interval's lower bound exceed such a referent (null) value (i.e., improving upon the internal pilot, as shown by the vertical cyan line segment in

Figure S2A, intersecting the minimally-detectable alternative kappa value ‘κ1’ of 0.85 for the referent null value ‘κ0’ of 0.76 at the curve estimating minimally-detectable κ values). This motivated the overall target sample size of N=280 for the clinician validation substudy.

We provided three obstetrician-gynecologists with an N3C dataset containing all records from OMOP clinical data tables for measurements, observations, conditions, drugs, and procedures for 240 randomly-selected and stratified pregnancy episodes greater than one day from 166 pregnant persons, of whom 72 had more than one recorded pregnancy, and 40 non-pregnant persons, hence total N=280 (Figure S2B). The sample included records identified by the algorithm as falling into one of the following stratified categories: (1) 200 pregnancy episodes consisting of four pregnancy outcomes to yield 40 episodes per each of 3 non-live-birth-outcome groups and 80 for the live birth group (noting that we purposefully sampled 2:1 for live-birth to non-live birth categories given the overall predominance and importance of this outcome for many future analyses), (2) 40 pregnancy episodes without a known pregnancy outcome, and (3) a group of records with no pregnancy episodes identified. Within the group with no pregnancy episodes identified, we randomly selected 40 females of reproductive age with a pregnancy-related concept yet no evidence of pregnancy progression. We calculated the percent agreement for date-related metrics stratified by the number of days (7, 14, 21, and 28 days) between HIPPS' dates and the clinicians' dates [7]. For classification of episodes by outcome status, we used both large-sample (asymptotic) and "exact" versions of conventional methods for assessing classification schemes, wherever possible [8,9]. As an example of using both versions, we employed Cohen's kappa coefficient for quantifying uncertainty in this estimate using an "exact" conditional approach appropriate for modestly-sized samples [8]. This overall set of records was supplemented by seven additional episodes identified by reviewing clinicians yet not by HIPPS from among the sampled records for included patients. In addition we estimated Gwet's AC1 as another chance-corrected measure of agreement to complement Cohen's kappa coefficient [10] as Cohen's kappa coefficient has been found to be affected by prevalence and marginal probability [11]. For evaluating interrater agreement, we had an additional clinician "double-review" a random 10% subset of the validation records to compare annotations between the two sets of clinicians. This subset included 17 pregnancy episodes (from 11 patients) and three patients without any pregnancy episodes. For date-related metrics, any difference in less than or equal to 14 days was considered a match between the two clinicians. We report 95% confidence intervals for each chance-corrected agreement measure to quantify uncertainty in the feasibility-constrained validation substudy comparing HIPPS and clinicians' determinations. Lastly, noting that individual patients could contribute to more than one pregnancy episode and, thus, be randomly sampled again for a separate pregnancy episodes, inducing small amounts of violation of typical statistics' independent observations assumptions (perhaps leading to anticonservative inferences), we also note that a series of parallel analyses accounting for within-patient dependencies, though less well-known in informatics communities [9], indicate similar conclusions are obtained. Measures of agreement estimates and inferential statistics were obtained using the irrCAC package in R (version 3.6+) and Cytel's StatXact® v.8 [12,13].

Supplementary Results

I. Data availability analysis

"Gestation period, X weeks" shows up in 78.8% of pregnant persons from 70 out of 72 sites (Figure S3). We further looked at the distribution of values for four concepts with less than 60.0% missingness and present in at least 15 sites (Figure S4). We chose to focus on "Gestational age", "Gestational age Estimated", and "Gestational age in weeks" for HIP as these concepts seem to give the most information over the span of an episode in addition to the "Gestation period, X weeks" concepts. Lastly, we checked the availability of concepts related to parity, gravidity, single/multiple pregnancy as well as gestational age by site to check if this data can be inferred in N3C (Figure S5). Several such concepts, including gravidity and parity, are not routinely available in N3C.

J. Gestational timing concept curation and exploration

For each of the 1,417 pregnancy-specific concepts we determined the mean month and standard deviation of when the concept occurs relative to the pregnancy start of HIP-inferred episodes with outcomes. We retained a total of 145 concepts after filtering out concepts with a standard deviation >1.5 months (Table S3). Clinicians assigned expected gestational time ranges to each of the 145 concepts (Table S3), and we kept 74 concepts with time ranges ≤ 3 months. The vast majority of records of the 43 clinician-curated GR3m gestational timing concepts occurred during the expected time ranges using HIP's episodes (Figure S7). Similarly, the records of the GW concepts aligned with their corresponding clinician gestational time range.

K. Performance of HIPPS and HIPPS component algorithms to baseline

To assess completeness of our approach for inferring pregnancy episodes, we compared our HIPPS overall and HIP/PPS components to a baseline that is in the spirit of the existing literature using outcomes to define episodes. In Figure S8A, we show that compared to an outcome-based baseline, our gestation-based HIP, HIP combined, PPS, and HIPPS all have more inferred episodes. We found 1.3X more episodes for HIP, and 1.4X more episodes for PPS, compared to baseline due to these additional episodes without outcomes. We stratify in Figure S8A and S8B by whether the episode has an outcome to highlight how our approach provides inference for episodes without outcomes.

L. Analysis of PPS contribution to HIPPS

PPS supported 724,251 (88.7%) episodes and matched on outcomes, including those without outcomes, for 680,396 episodes (83.3%) out of 816,471 HIP episodes. Both abortion and ectopic pregnancy had over 50% of episodes with discordant outcomes between the two algorithms (Table S8A). Of the 119,755 episodes with discordant outcomes between HIP and PPS, 10,514 episodes (8.8%) utilized the outcomes inferred by PPS due to their later occurrence in the pregnancy episodes compared to HIP's outcomes (Table S8B). We also found that PPS offers the advantage of extending the timespan of evidence we find recorded in the data for a progressing

pregnancy. Notably, PPS contained 1.6-fold more recorded episodes of length > 6 months compared to HIP.

For presentation purposes, we exclude PPS only from the main results of the manuscript because upon investigation, the episodes were less likely to have an outcome (S8C) and more likely to be nonspecific with respect to pregnancy start (S8D). For the end user, the code provides flags for these episodes and the researcher can choose to keep them in for their respective analyses. Future refreshes of N3C data or use in another EHR dataset may not warrant their exclusion.

M. Clinician validation substudy - additional details for interrater agreement findings

The interrater agreement was high; percent agreement between the assigned pair of two clinicians for the overlap of 17 records (including 3 deemed non-episodes by clinicians) ranged from 88.2% to 100.0% for each attribute, with Cohen's kappa coefficient (**κ**; adding additionally-identified episodes, by type, *not* identified by HIPPS) estimated as 1 across the two distinct pairs of clinicians with overlapping records reviewed (subsets of size 14 & 6), for all attributes except for inferred end date and gestational age, which each had a **κ** estimated as 0.714 with asymptotic 95% CI, 0.214 to 1.0 (exact p-value of test for chance agreement of 0.06667). This is in keeping with notably ample levels of agreement yet lack of full chance-corrected agreement between consensus of clinicians' reviews relative to HIPPS across 287 records; Cohen's kappa coefficient estimated as 0.872 with asymptotic 95% CI, 0.828 to 0.915, exact p-value of test for chance agreement: 1.38×10^{-43}, consistent conclusion-wise with a more readily-reproducible Monte-Carlo-sampling-obtained [via 10,000 samples] upper bound of 95% confidence interval, 4.46×10^{-4}. We also estimated Gwet's AC1 as 0.875 (95% CI: 0.833 to 0.918, $p < 0.0001$; as an alternative measure of agreement less prone to issues faced by **κ**) in comparing algorithm-clinician classification agreement overall for the records reviewed (n=287) and 0.857 (95% CI: 0.808 to 0.905, $p < 0.0001$) among the subset of records with episodes (n=248; Gwet's AC1 indicates its appeal for robustness to differing categories' marginal proportions by noting that, in this case, Cohen's **κ** estimates are 0.937, 95%CI: 0.916 – 0.958).

N. Comparison to national sample of live births and pregnancies with COVID-status

In Table S9, we compare maternal characteristics for our HIPPS-inferred pregnancies in N3C to national summary statistics of live births 2019-2021 reported in a recent preprint [14]. Pre-pandemic and during-pandemic, our sample is very similar to national birth statistics for maternal age, although our sample is slightly older across both timeframes. For race, our sample pre-pandemic is very similar for Hispanic mothers, but among non-Hispanic mothers our samples differ in the following ways: less white, more Asian, more missing/unknown, more Black. During the pandemic, our sample is more Hispanic and continues to be less white, more Asian, more missing/unknown, and more Black. All of these differences, except for white, however, are relatively small (2 to 4 percentage points).

We also compared our sample of COVID-positive pregnant persons to CDC estimates sampled from 14 states [15]. In Table S10, we present maternal characteristics for HIPPS-inferred pregnancies in N3C during the pandemic who are COVID positive and negative compared to the

CDC surveillance estimates. Our sample is more similar to the national sample of live births presented in Table S9 whereas the CDC sample appears to be more Hispanic than the national sample, possibly explained by the 14 states included in the CDC data. For maternal age, our data is very similar to the CDC data. Here, our sample of pregnant persons is similarly white non-Hispanic, however, we have more Black persons, fewer Hispanic persons, more other/unknown, and less Asian. Across COVID status, the patterns are directionally similar but different in magnitude.

Supplementary Tables

Table S1. Concept sets used for pregnancy and COVID-related variables.

			30	3,051	903	176	1,417	930	74	88
**Concept ID**	**Concept Name**	**Category (Per Matcho et al)**	**Variable**	**Matcho et al**	**Matcho et al in N3C (at least 100 patients)**	**HIP concepts in N3C (at least 100 patients)**	**Specific to initial possibly-pregnant cohort (633K)**	**Used for HIP**	**Used for PPS**	**Used for ESD**
444098	Gestation period, 40 weeks		Gestation period, X weeks (GW)	X	X	X	X	X	X	X
441678	Gestation period, 33 weeks	GEST	Gestation period, X weeks (GW)	X	X	X	X	X	X	X
444417	Gestation period, 29 weeks	GEST	Gestation period, X weeks (GW)	X	X	X	X	X	X	X
435640	Gestation period, 25 weeks	GEST	Gestation period, X weeks (GW)	X	X	X	X	X	X	X
444267	Gestation period, 35 weeks	GEST	Gestation period, X weeks (GW)	X	X	X	X	X	X	X
432430	Gestation period, 27 weeks	GEST	Gestation period, X weeks (GW)	X	X	X	X	X	X	X
433864	Gestation period, 31 weeks	GEST	Gestation period, X weeks (GW)	X	X	X	X	X	X	X
3009306	Alpha-1-Fetoprotein [Mass/volume] in Serum or Plasma	AFP	Gestational age, one week to three months (GR3m)	X	X		X		X	X
3024370	Alpha-1-Fetoprotein [Multiple of the median] in Serum or Plasma	AFP	Gestational age, one week to three months (GR3m)	X	X		X		X	X
2212198	Alpha-fetoprotein (AFP); serum	AFP	Gestational age, one week to three months (GR3m)	X	X		X		X	X
2110279	Amniocentesis; diagnostic	AMNIO	Gestational age, one week to three months (GR3m)	X	X		X		X	X
2211763	Doppler echocardiography, fetal, pulsed wave and/or continuous wave with spectral display; complete	OTEST	Gestational age, one week to three months (GR3m)	X	X		X		X	X
2722250	Echocardiography, fetal, cardiovascular system, real time with image documentation (2D), with or without M-mode recording	OTEST	Gestational age, one week to three months (GR3m)	X	X		X		X	X
3025455	Estriol (E3).unconjugated [Mass/volume] in Serum or Plasma	OTEST	Gestational age, one week to three months (GR3m)	X	X		X		X	X
4062558	False labor at or after 37 completed weeks of gestation		Gestational age, one week to three months (GR3m)	X	X		X		X	X
3014716	Glucose [Mass/volume] in Serum or Plasma --1 hour post 100 g glucose PO	DIAB	Gestational age, one week to three months (GR3m)	X	X		X		X	X
3010300	Glucose [Mass/volume] in Serum or Plasma --1 hour post dose glucose	DIAB	Gestational age, one week to three months (GR3m)	X	X		X		X	X
3006717	Glucose [Mass/volume] in Serum or Plasma --2 hours post 100 g glucose PO	DIAB	Gestational age, one week to three months (GR3m)	X	X		X		X	X
3027457	Glucose [Mass/volume] in Serum or Plasma --3 hours post 100 g glucose PO	DIAB	Gestational age, one week to three months (GR3m)	X	X		X		X	X
3027198	Glucose [Mass/volume] in Serum or Plasma --3 hours post dose glucose	DIAB	Gestational age, one week to three months (GR3m)	X	X		X		X	X
432695	Post-term pregnancy	POSTT	Gestational age, one week to three months (GR3m)	X	X		X		X	X
4094910	Pregnancy test positive	PCONF	Gestational age, one week to three months (GR3m)	X	X		X		X	X
2211785	Ultrasonic guidance for amniocentesis, imaging supervision and interpretation	AMNIO	Gestational age, one week to three months (GR3m)	X	X		X		X	X
2211751	Ultrasound, pregnant uterus, real time with image documentation, fetal and maternal evaluation plus detailed fetal anatomic examination, transabdominal approach; single or first gestation	ULS	Gestational age, one week to three months (GR3m)	X	X		X		X	X
2211747	Ultrasound, pregnant uterus, real time with image documentation, fetal and maternal evaluation, first trimester (< 14 weeks 0 days), transabdominal approach; single or first gestation	ULS	Gestational age, one week to three months (GR3m)	X	X		X		X	X
2211753	Ultrasound, pregnant uterus, real time with image documentation, first trimester fetal nuchal translucency measurement, transabdominal or transvaginal approach; single or first gestation	NULS	Gestational age, one week to three months (GR3m)	X	X		X		X	X
4175637	Premature pregnancy delivered	PREM	Delivery record only (DELIV)	X	X	X	X	X		
4065737	Delivery by combination of forceps and vacuum extractor		Delivery record only (DELIV)	X	X	X	X	X		
4014720	Normal birth	DELIV	Delivery record only (DELIV)	X	X	X	X	X		
2110321	Vaginal delivery only, after previous cesarean delivery (with or without episiotomy and/or forceps); including postpartum care	POST	Delivery record only (DELIV)	X	X	X	X	X		
2110319	Routine obstetric care including antepartum care, vaginal delivery (with or without episiotomy, and/or forceps) and postpartum care, after previous cesarean delivery	POST	Delivery record only (DELIV)	X	X	X	X	X		
2110309	Vaginal delivery only (with or without episiotomy and/or forceps); including postpartum care	POST	Delivery record only (DELIV)	X	X	X	X	X		
2110308	Vaginal delivery only (with or without episiotomy and/or forceps)	DELIV	Delivery record only (DELIV)	X	X	X	X	X		
2110307	Routine obstetric care including antepartum care, vaginal delivery (with or without episiotomy, and/or forceps) and postpartum care	POST	Delivery record only (DELIV)	X	X	X	X	X		
441641	Delivery normal	DELIV	Delivery record only (DELIV)	X	X	X	X	X		
435022	Forceps delivery - delivered	DELIV	Delivery record only (DELIV)	X	X	X	X	X		
433260	Mother delivered	DELIV	Delivery record only (DELIV)	X	X	X	X	X		
4015701	Cesarean section	DELIV	Delivery record only (DELIV)	X	X	X	X	X		
2110323	Cesarean delivery only, following attempted vaginal delivery after previous cesarean delivery	DELIV	Delivery record only (DELIV)	X	X	X	X	X		
2110317	Cesarean delivery only; including postpartum care	POST	Delivery record only (DELIV)	X	X	X	X	X		
2110316	Cesarean delivery only	DELIV	Delivery record only (DELIV)	X	X	X	X	X		
2101814	Anesthesia for cesarean delivery following neuraxial labor analgesia/anesthesia (List separately in addition to code for primary procedure performed)	DELIV	Delivery record only (DELIV)	X	X	X	X	X		
2101807	Anesthesia for cesarean delivery only	LDEL	Delivery record only (DELIV)	X	X	X	X	X		
437942	Cesarean delivery - delivered	DELIV	Delivery record only (DELIV)	X	X	X	X	X		
193277	Deliveries by cesarean	DELIV	Delivery record only (DELIV)	X	X	X	X	X		
4060558	Uterine scar from previous surgery in pregnancy, childbirth and the puerperium - delivered		Delivery record only (DELIV)	X	X	X	X	X		
192978	First degree perineal tear during delivery - delivered	DELIV	Delivery record only (DELIV)	X	X	X	X	X		
193525	Abdominal pregnancy	PCOMP	Ectopic pregnancy (ECT)	X	X	X	X	X		
437611	Ectopic pregnancy	ECT	Ectopic pregnancy (ECT)	X	X	X	X	X		
2110300	Laparoscopic treatment of ectopic pregnancy; with salpingectomy and/or oophorectomy	ECT_SURG1	Ectopic pregnancy (ECT)	X	X	X	X	X		

199076	Tubal pregnancy	ECT	Ectopic pregnancy (ECT)	X	X	X	X	X		
439922	Gestation period, 24 weeks	GEST	Gestation period, X weeks (GW)	X	X	X	X	X		X
3048230	Gestational age in weeks	GEST	Gestational age, other	X	X	X	X	X		X
436747	Legal termination of pregnancy without complication	SA	Induced abortion (AB)	X	X	X	X	X		
2101812	Anesthesia for induced abortion procedures	AB	Induced abortion (AB)	X	X	X	X	X		
2110329	Induced abortion, by dilation and curettage	AB	Induced abortion (AB)	X	X	X	X	X		
2110330	Induced abortion, by dilation and evacuation	AB	Induced abortion (AB)	X	X	X	X	X		
4081422	Elective termination of pregnancy		Induced abortion (AB)	X	X	X	X	X		
4014295	Single live birth	LB	Livebirth (LB)	X	X	X	X	X		
4014296	Twins - both live born	LB	Livebirth (LB)	X	X	X	X	X		
76482	Missed miscarriage	SA	Spontaneous abortion (SA)	X	X	X	X	X		
192678	Miscarriage complicated by damage to pelvic organs and/or tissues	SA	Spontaneous abortion (SA)	X				X		
193820	Miscarriage complicated by renal failure		Spontaneous abortion (SA)	X				X		
194694	Failed attempted abortion with complication	SA	Spontaneous abortion (SA)	X	X					
194704	Miscarriage complicated by embolism		Spontaneous abortion (SA)	X				X		
195317	Incomplete miscarriage with genital tract or pelvic infection	SA	Spontaneous abortion (SA)	X	X	X		X		
195319	Abortion complicated by renal failure	SA	Spontaneous abortion (SA)	X						
195594	Spontaneous abortion complicated by delayed AND/OR excessive hemorrhage	SA	Spontaneous abortion (SA)	X						
196746	Miscarriage complicated by shock		Spontaneous abortion (SA)	X				X		
197612	Abortion complicated by delayed AND/OR excessive hemorrhage	SA	Spontaneous abortion (SA)	X						
197617	Legal abortion complicated by delayed AND/OR excessive hemorrhage	SA	Spontaneous abortion (SA)	X						
201076	Abortion complicated by damage to pelvic organs AND/OR tissues	SA	Spontaneous abortion (SA)	X						
432682	Complete miscarriage with delayed or excessive hemorrhage	SA	Spontaneous abortion (SA)	X				X		
432689	Incomplete miscarriage with metabolic disorder		Spontaneous abortion (SA)	X				X		
434094	Miscarriage with complication	SA	Spontaneous abortion (SA)	X	X	X	X	X		
4014454	Single stillbirth	SB	Stillbirth (SB)	X	X	X	X	X		
4060687	Intrauterine death - delivered	SB	Stillbirth (SB)	X	X	X	X	X		
4015701	Cesarean section	DELIV	Cesarean section	X	X	X	X			
2110323	Cesarean delivery only, following attempted vaginal delivery after previous cesarean delivery	DELIV	Cesarean section	X	X	X	X			
2110317	Cesarean delivery only; including postpartum care	POST	Cesarean section	X	X	X	X			
2110316	Cesarean delivery only	DELIV	Cesarean section	X	X	X	X			
2101814	Anesthesia for cesarean delivery following neuraxial labor analgesia/anesthesia (List separately in addition to code for primary procedure performed)	DELIV	Cesarean section	X	X	X	X			
2101807	Anesthesia for cesarean delivery only	LDEL	Cesarean section	X	X	X	X			
437942	Cesarean delivery - delivered	DELIV	Cesarean section	X	X	X	X			
193277	Deliveries by cesarean	DELIV	Cesarean section	X	X	X	X			
2211754	Ultrasound, pregnant uterus, real time with image documentation, first trimester fetal nuchal translucency measurement, transabdominal or transvaginal approach; each additional gestation (List separately in addition to code for primary procedure)	NULS	First trimester	X	X		X			
2211753	Ultrasound, pregnant uterus, real time with image documentation, first trimester fetal nuchal translucency measurement, transabdominal or transvaginal approach; single or first gestation	NULS	First trimester	X	X		X			
2211748	Ultrasound, pregnant uterus, real time with image documentation, fetal and maternal evaluation, first trimester (< 14 weeks 0 days), transabdominal approach; each additional gestation (List separately in addition to code for primary procedure)	ULS	First trimester	X	X		X			
2211747	Ultrasound, pregnant uterus, real time with image documentation, fetal and maternal evaluation, first trimester (< 14 weeks 0 days), transabdominal approach; single or first gestation	ULS	First trimester	X	X		X			
3011536	Delivery date Estimated	PCONF	Gestational age, other	X	X		X			
4059478	Estimated date of delivery	AGP	Gestational age, other	X	X		X			
3036844	Gestational age US composite estimate	GEST	Gestational age, other	X	X		X			
4260747	Length of gestation at birth		Gestational age, other	X	X		X			
442442	Elderly primigravida with antenatal problem	PCOMP	Gravidity	X	X		X			
4133029	Primigravida	PCONF	Gravidity	X	X		X			
441085	Elderly primigravida	PCONF	Gravidity	X	X		X			
433826	Continuing pregnancy after abortion of one fetus or more		Multiple pregnancy	X	X		X			
432373	Continuing pregnancy after intrauterine death one fetus or more	PCOMP	Multiple pregnancy	X	X		X			
432969	Multiple pregnancy	PCONF	Multiple pregnancy	X	X		X			
3045823	Multiple pregnancy	PCONF	Multiple pregnancy	X	X		X			
441919	Twin pregnancy	PCONF	Multiple pregnancy	X	X		X			
434097	Twin pregnancy with antenatal problem	PCOMP	Multiple pregnancy	X	X		X			
4014296	Twins - both live born	LB	Multiple pregnancy	X	X	X	X			
2211750	Ultrasound, pregnant uterus, B-scan and/or real time with image documentation; complete (complete fetal and maternal evaluation), multiple gestation, after the first trimester	ULS	Multiple pregnancy	X	X		X			
3011465	Choriogonadotropin.beta subunit free [Units/volume] in Serum or Plasma	HCG	Other - Specific to initial possibly-pregnant cohort (633K)	X	X		X			
4173323	Extremely low birth weight infant		Other - Specific to initial possibly-pregnant cohort (633K)	X	X		X			
3038136	Choriogonadotropin.beta subunit [Units/volume] in Serum or Plasma	HCG	Other - Specific to initial possibly-pregnant cohort (633K)	X	X		X			
3008615	Choriogonadotropin.beta subunit [Units/volume] in Serum or Plasma by Immunoassay (EIA) 3rd IS		Other - Specific to initial possibly-pregnant cohort (633K)	X	X		X			
4312727	Secondary physiologic amenorrhea	AMEN	Other - Specific to initial possibly-pregnant cohort (633K)	X	X		X			
3025994	Estriol (E3) [Mass/volume] in Serum or Plasma	OTEST	Other - Specific to initial possibly-pregnant cohort (633K)	X	X		X			

443800	Amenorrhea	AMEN	Other - Specific to initial possibly-pregnant cohort (633K)	X	X		X			
2212631	Gonadotropin, chorionic (hCG); quantitative	HCG	Other - Specific to initial possibly-pregnant cohort (633K)	X	X		X			
440532	Heavy-for-dates at birth regardless of gestation period	POST	Other - Specific to initial possibly-pregnant cohort (633K)	X	X		X			
3018171	Choriogonadotropin [Units/volume] in Serum or Plasma	HCG	Other - Specific to initial possibly-pregnant cohort (633K)	X	X		X			
2110274	Follicle puncture for oocyte retrieval, any method	OVUL	Other - Specific to initial possibly-pregnant cohort (633K)	X	X		X			
3007332	Glucose [Mass/volume] in Serum or Plasma --1 hour post 75 g glucose PO	DIAB	Other - Specific to initial possibly-pregnant cohort (633K)	X	X		X			
3025673	Glucose [Mass/volume] in Serum or Plasma --2 hours post 75 g glucose PO	DIAB	Other - Specific to initial possibly-pregnant cohort (633K)	X	X		X			
4094911	Pregnancy test equivocal	Not categorized	Other - Specific to initial possibly-pregnant cohort (633K)	X	X		X			
2213351	Oocyte identification from follicular fluid	OVUL	Other - Specific to initial possibly-pregnant cohort (633K)	X	X		X			
440203	Fetal or neonatal effect of condition of umbilical cord	POST	Other - Specific to initial possibly-pregnant cohort (633K)	X	X		X			
2213362	Assisted oocyte fertilization, microtechnique; less than or equal to 10 oocytes	OVUL	Other - Specific to initial possibly-pregnant cohort (633K)	X	X		X			
2514560	Delivery/birthing room resuscitation, provision of positive pressure ventilation and/or chest compressions in the presence of acute inadequate ventilation and/or cardiac output	POST	Other - Specific to initial possibly-pregnant cohort (633K)	X	X		X			
2213364	Biopsy, oocyte polar body or embryo blastomere, microtechnique (for pre-implantation genetic diagnosis); less than or equal to 5 embryos	OVUL	Other - Specific to initial possibly-pregnant cohort (633K)	X	X		X			
2110197	Artificial insemination; intra-uterine	OVUL	Other - Specific to initial possibly-pregnant cohort (633K)	X	X		X			
2213347	Culture of oocyte(s)/embryo(s), less than 4 days	OVUL	Other - Specific to initial possibly-pregnant cohort (633K)	X	X		X			
80204	Fetal growth restriction	PCOMP	Other - Specific to initial possibly-pregnant cohort (633K)	X	X		X			
2213350	Assisted embryo hatching, microtechniques (any method)	OVUL2	Other - Specific to initial possibly-pregnant cohort (633K)	X	X		X			
2213361	Extended culture of oocyte(s)/embryo(s), 4-7 days	OVUL2	Other - Specific to initial possibly-pregnant cohort (633K)	X	X		X			
2110247	Salpingectomy, complete or partial, unilateral or bilateral (separate procedure)	ECT_SURG1	Other - Specific to initial possibly-pregnant cohort (633K)	X	X		X			
4012558	Possible pregnancy	Not categorized	Other - Specific to initial possibly-pregnant cohort (633K)	X	X		X			
2213363	Assisted oocyte fertilization, microtechnique; greater than 10 oocytes	OVUL	Other - Specific to initial possibly-pregnant cohort (633K)	X	X		X			
4057246	Postpartum psychosis	POST	Other - Specific to initial possibly-pregnant cohort (633K)	X	X		X			
3026300	Glucose [Mass/volume] in Serum or Plasma --2 hours post dose glucose	DIAB	Other - Specific to initial possibly-pregnant cohort (633K)	X	X		X			
3005625	Estriol (E3).unconjugated [Multiple of the median] adjusted in Serum or Plasma	OTEST	Other - Specific to initial possibly-pregnant cohort (633K)	X	X		X			
2212094	Obstetric panel This panel must include the following: Blood count, complete (CBC), automated and automated differential WBC count (85025 or 85027 and 85004) OR Blood count, complete (CBC), automated (85027) and appropriate manual differential WBC count (85007 or 85009) Hepatitis B surface antigen (HBsAg) (87340) Antibody, rubella (86762) Syphilis test, non-treponemal antibody; qualitative (eg, VDRL, RPR, ART) (86592) Antibody screen, RBC, each serum technique (86850) Blood typing, ABO (86900) AND Blood typing, Rh (D) (86901)	OTEST	Other - Specific to initial possibly-pregnant cohort (633K)	X	X		X			
3016704	1 minute Apgar Score	POST	Other - Specific to initial possibly-pregnant cohort (633K)	X	X		X			
4147414	Varicose veins of legs in pregnancy		Other - Specific to initial possibly-pregnant cohort (633K)	X	X		X			
2110275	Embryo transfer, intrauterine	OVUL2	Other - Specific to initial possibly-pregnant cohort (633K)	X	X		X			
4024659	Gestational diabetes mellitus	PCOMP	Other - Specific to initial possibly-pregnant cohort (633K)	X	X		X			
441959	Amniotic fluid -meconium stain	PCOMP	Other - Specific to initial possibly-pregnant cohort (633K)	X	X		X			
194439	Obstetric perineal wound disruption	POST	Other - Specific to initial possibly-pregnant cohort (633K)	X	X		X			
3000845	Glucose [Mass/volume] in Serum or Plasma --2 hours post XXX challenge	DIAB	Other - Specific to initial possibly-pregnant cohort (633K)	X	X		X			
3030177	Glucose tolerance [Interpretation] in Serum or Plasma Narrative	DIAB	Other - Specific to initial possibly-pregnant cohort (633K)	X	X		X			

2213352	Preparation of embryo for transfer (any method)	OVUL2	Other - Specific to initial possibly-pregnant cohort (633K)	X	X		X			
434758	Exceptionally large at birth	POST	Other - Specific to initial possibly-pregnant cohort (633K)	X	X		X			
435028	Puerperal pyrexia of unknown origin	POST	Other - Specific to initial possibly-pregnant cohort (633K)	X	X		X			
4299535	Pregnant	PCONF	Other - Specific to initial possibly-pregnant cohort (633K)	X	X		X			
3010164	Choriogonadotropin.beta subunit [Multiple of the median] adjusted in Serum or Plasma	HCG	Other - Specific to initial possibly-pregnant cohort (633K)	X	X		X			
80471	Disorder of lactation	POST	Other - Specific to initial possibly-pregnant cohort (633K)	X	X		X			
4061848	Retained intrauterine contraceptive device in pregnancy		Other - Specific to initial possibly-pregnant cohort (633K)	X	X		X			
4047564	Routine antenatal care	AGP	Other - Specific to initial possibly-pregnant cohort (633K)	X	X		X			
436166	Mild hyperemesis gravidarum	PCOMP	Other - Specific to initial possibly-pregnant cohort (633K)	X	X		X			
435604	Viral disease in mother complicating pregnancy, childbirth AND/OR puerperium	PCOMP	Other - Specific to initial possibly-pregnant cohort (633K)	X	X		X			
3002666	Glucose [Mass/volume] in Serum or Plasma --baseline	DIAB	Other - Specific to initial possibly-pregnant cohort (633K)	X	X		X			
439658	Disorder of pregnancy		Other - Specific to initial possibly-pregnant cohort (633K)	X	X		X			
2212633	Gonadotropin, chorionic (hCG); free beta chain	HCG	Other - Specific to initial possibly-pregnant cohort (633K)	X	X		X			
436485	Hyperemesis gravidarum with metabolic disturbance	PCOMP	Other - Specific to initial possibly-pregnant cohort (633K)	X	X		X			
440785	Vomiting of pregnancy	PCOMP	Other - Specific to initial possibly-pregnant cohort (633K)	X	X		X			
3032989	Alpha-1-Fetoprotein multiple of the median cutoff [Multiple of the median] in Serum or Plasma		Other - Specific to initial possibly-pregnant cohort (633K)	X	X		X			
76771	Obstetric non-purulent mastitis with postnatal complication	POST	Other - Specific to initial possibly-pregnant cohort (633K)	X	X		X			
432441	Finding of length of gestation	POST	Other - Specific to initial possibly-pregnant cohort (633K)	X	X		X			
441364	Complication of the puerperium	POST	Other - Specific to initial possibly-pregnant cohort (633K)	X	X		X			
198495	Fourth degree perineal laceration	POST	Other - Specific to initial possibly-pregnant cohort (633K)	X	X		X			
3004376	Choriogonadotropin [Multiple of the median] in Serum or Plasma		Other - Specific to initial possibly-pregnant cohort (633K)	X	X		X			
436740	Cervical incompetence	PCOMP	Other - Specific to initial possibly-pregnant cohort (633K)	X	X		X			
4063038	Infections of bladder in pregnancy		Other - Specific to initial possibly-pregnant cohort (633K)	X	X		X			
4058243	Diabetes mellitus during pregnancy, childbirth and the puerperium	POST	Other - Specific to initial possibly-pregnant cohort (633K)	X	X		X			
40758989	Choriogonadotropin.beta subunit [Multiple of the median] in Serum or Plasma	HCG	Other - Specific to initial possibly-pregnant cohort (633K)	X	X		X			
4146482	Urinary tract infection in pregnancy	PCOMP	Other - Specific to initial possibly-pregnant cohort (633K)	X	X		X			
438205	Maternal syphilis during pregnancy - baby not yet delivered	PCOMP	Other - Specific to initial possibly-pregnant cohort (633K)	X	X		X			
4060295	Infections of kidney in pregnancy		Other - Specific to initial possibly-pregnant cohort (633K)	X	X		X			
438815	Disorder of amniotic cavity AND/OR membrane	PCOMP	Other - Specific to initial possibly-pregnant cohort (633K)	X	X		X			
4307820	Unplanned pregnancy	PCONF	Other - Specific to initial possibly-pregnant cohort (633K)	X	X		X			
439393	Pre-eclampsia	PCOMP	Other - Specific to initial possibly-pregnant cohort (633K)	X	X		X			
4061847	Abnormal hematologic finding on antenatal screening of mother		Other - Specific to initial possibly-pregnant cohort (633K)	X	X		X			
4217975	Normal pregnancy	PCONF	Other - Specific to initial possibly-pregnant cohort (633K)	X	X		X			
4062133	Infection of obstetric surgical wound	POST	Other - Specific to initial possibly-pregnant cohort (633K)	X	X		X			
3025232	Glucose [Mass/volume] in Serum or Plasma --1 hour post XXX challenge	DIAB	Other - Specific to initial possibly-pregnant cohort (633K)	X	X		X			
4060424	Mental disorders during pregnancy, childbirth and the puerperium	PCOMP	Other - Specific to initial possibly-pregnant cohort (633K)	X	X		X			

4129834	Pregnancy with isoimmunization		Other - Specific to initial possibly-pregnant cohort (633K)	X	X		X			
4142037	Gestational proteinuria		Other - Specific to initial possibly-pregnant cohort (633K)	X	X		X			
4167493	Pregnancy-induced hypertension	PCOMP	Other - Specific to initial possibly-pregnant cohort (633K)	X	X		X			
2212199	Alpha-fetoprotein (AFP); amniotic fluid	AFP	Other - Specific to initial possibly-pregnant cohort (633K)	X	X		X			
434089	Antepartum hemorrhage	PCOMP	Other - Specific to initial possibly-pregnant cohort (633K)	X	X		X			
440787	Drug dependence in mother complicating pregnancy, childbirth AND/OR puerperium	PCOMP	Other - Specific to initial possibly-pregnant cohort (633K)	X	X		X			
4230221	Fatigue during pregnancy		Other - Specific to initial possibly-pregnant cohort (633K)	X	X		X			
321074	Pre-existing hypertension complicating pregnancy, childbirth and puerperium	PCOMP	Other - Specific to initial possibly-pregnant cohort (633K)	X	X		X			
4030872	Hyperemesis gravidarum	PCOMP	Other - Specific to initial possibly-pregnant cohort (633K)	X	X		X			
139895	Thyroid dysfunction during pregnancy, childbirth and the puerperium	PCOMP	Other - Specific to initial possibly-pregnant cohort (633K)	X	X		X			
2004844	Repair of other current obstetric laceration	POST	Other - Specific to initial possibly-pregnant cohort (633K)	X	X		X			
435875	Complication of pregnancy, childbirth and/or the puerperium	PCOMP	Other - Specific to initial possibly-pregnant cohort (633K)	X	X		X			
441081	Maternal gonorrhea during pregnancy - baby not yet delivered	PCOMP	Other - Specific to initial possibly-pregnant cohort (633K)	X	X		X			
4062569	Urinary tract infection following delivery	POST	Other - Specific to initial possibly-pregnant cohort (633K)	X	X		X			
437688	Abnormal findings on antenatal screening of mother	PCOMP	Other - Specific to initial possibly-pregnant cohort (633K)	X	X		X			
4060556	Uterine scar from previous surgery in pregnancy, childbirth and the puerperium		Other - Specific to initial possibly-pregnant cohort (633K)	X	X		X			
194700	Diabetes mellitus in mother complicating pregnancy, childbirth AND/OR puerperium	PCOMP	Other - Specific to initial possibly-pregnant cohort (633K)	X	X		X			
3014064	Pregnancy associated plasma protein A [Multiple of the median] in Serum or Plasma	OTEST	Other - Specific to initial possibly-pregnant cohort (633K)	X	X		X			
4060429	Disease of the respiratory system complicating pregnancy, childbirth and/or the puerperium		Other - Specific to initial possibly-pregnant cohort (633K)	X	X		X			
194710	Placenta previa without hemorrhage	PCOMP	Other - Specific to initial possibly-pregnant cohort (633K)	X	X		X			
3049837	Alpha-1-Fetoprotein interpretation in Serum or Plasma Narrative		Other - Specific to initial possibly-pregnant cohort (633K)	X	X		X			
433823	Infectious disease in mother complicating pregnancy, childbirth AND/OR puerperium	PCOMP	Other - Specific to initial possibly-pregnant cohort (633K)	X	X		X			
436489	Complication of obstetrical surgery AND/OR procedure	POST	Other - Specific to initial possibly-pregnant cohort (633K)	X	X		X			
42872398	Maternal obesity complicating pregnancy, childbirth and the puerperium, antepartum	PCOMP	Other - Specific to initial possibly-pregnant cohort (633K)	X	X		X			
4062791	Endocrine, nutritional and metabolic disease complicating pregnancy, childbirth and puerperium		Other - Specific to initial possibly-pregnant cohort (633K)	X	X		X			
441092	Advanced maternal age gravida	PCONF	Other - Specific to initial possibly-pregnant cohort (633K)	X	X		X			
440457	Threatened miscarriage	TA	Other - Specific to initial possibly-pregnant cohort (633K)	X	X		X			
4064724	Abnormal chromosomal and genetic finding on antenatal screening of mother		Other - Specific to initial possibly-pregnant cohort (633K)	X	X		X			
198212	Spotting per vagina in pregnancy	PCONF	Other - Specific to initial possibly-pregnant cohort (633K)	X	X		X			
4143296	Hemorrhoids in pregnancy		Other - Specific to initial possibly-pregnant cohort (633K)	X	X		X			
77340	Genitourinary tract infection in pregnancy	PCOMP	Other - Specific to initial possibly-pregnant cohort (633K)	X	X		X			
72693	Poor fetal growth affecting management	PCOMP	Other - Specific to initial possibly-pregnant cohort (633K)	X	X		X			
81636	Excessive fetal growth affecting management of mother	PCOMP	Other - Specific to initial possibly-pregnant cohort (633K)	X	X		X			
4065761	Hemorrhoids in the puerperium		Other - Specific to initial possibly-pregnant cohort (633K)	X	X		X			
4060296	Infections of the genital tract in pregnancy		Other - Specific to initial possibly-pregnant cohort (633K)	X	X		X			
314423	Benign essential hypertension complicating pregnancy, childbirth and the puerperium - not delivered	PCOMP	Other - Specific to initial possibly-pregnant cohort (633K)	X	X		X			

436483	Postpartum coagulation defects	POST	Other - Specific to initial possibly-pregnant cohort (633K)	X	X		X			
4334808	Fetal echocardiography	OTEST	Other - Specific to initial possibly-pregnant cohort (633K)	X	X		X			
4062790	Disease of the digestive system complicating pregnancy, childbirth and/or the puerperium		Other - Specific to initial possibly-pregnant cohort (633K)	X	X		X			
4239301	Unwanted pregnancy	PCONF	Other - Specific to initial possibly-pregnant cohort (633K)	X	X		X			
197050	Abnormality of organs AND/OR soft tissues of pelvis affecting pregnancy	PCOMP	Other - Specific to initial possibly-pregnant cohort (633K)	X	X		X			
438542	Gestation less than 24 weeks	GEST	Other - Specific to initial possibly-pregnant cohort (633K)	X	X		X			
194101	Uterine size for dates discrepancy	PCOMP	Other - Specific to initial possibly-pregnant cohort (633K)	X	X		X			
314432	Maternal hypotension syndrome	PCOMP	Other - Specific to initial possibly-pregnant cohort (633K)	X	X		X			
4161205	Hematoma of obstetric wound	POST	Other - Specific to initial possibly-pregnant cohort (633K)	X	X		X			
196758	Tumor of body of uterus affecting pregnancy	PCOMP	Other - Specific to initial possibly-pregnant cohort (633K)	X	X		X			
443214	Gestational edema	PCOMP	Other - Specific to initial possibly-pregnant cohort (633K)	X	X		X			
321080	Hypertension complicating pregnancy, childbirth and the puerperium	PCOMP	Other - Specific to initial possibly-pregnant cohort (633K)	X	X		X			
4058375	Disease of the skin and subcutaneous tissue complicating pregnancy, childbirth and/or the puerperium		Other - Specific to initial possibly-pregnant cohort (633K)	X	X		X			
4063155	Disease of nervous system complicating pregnancy, childbirth and puerperium		Other - Specific to initial possibly-pregnant cohort (633K)	X	X		X			
199891	Rhesus isoimmunization affecting pregnancy	PCOMP	Other - Specific to initial possibly-pregnant cohort (633K)	X	X		X			
437623	Polyhydramnios	PCOMP	Other - Specific to initial possibly-pregnant cohort (633K)	X	X		X			
4047392	Postpartum care	POST	Other - Specific to initial possibly-pregnant cohort (633K)	X	X		X			
77619	Reduced fetal movement	AGP	Other - Specific to initial possibly-pregnant cohort (633K)	X	X		X			
4289303	Placenta accreta		Other - Specific to initial possibly-pregnant cohort (633K)	X	X		X			
4145939	Congenital abnormality of uterus in pregnancy, childbirth and the puerperium		Other - Specific to initial possibly-pregnant cohort (633K)	X	X		X			
3008714	Choriogonadotropin [Multiple of the median] adjusted in Serum or Plasma	HCG	Other - Specific to initial possibly-pregnant cohort (633K)	X	X		X			
194699	Liver disorder in pregnancy	PCOMP	Other - Specific to initial possibly-pregnant cohort (633K)	X	X		X			
434697	Maternal tobacco abuse	PCOMP	Other - Specific to initial possibly-pregnant cohort (633K)	X	X		X			
2212713	Hemoglobin or RBCs, fetal, for fetomaternal hemorrhage; differential lysis (Kleihauer-Betke)	PCOMP	Other - Specific to initial possibly-pregnant cohort (633K)	X	X		X			
3026071	Glucose [Mass/volume] in Serum or Plasma --1 hour post meal	DIAB	Other - Specific to initial possibly-pregnant cohort (633K)	X	X		X			
4311246	Pre-existing hypertension in obstetric context		Other - Specific to initial possibly-pregnant cohort (633K)	X	X		X			
194109	Delayed AND/OR secondary postpartum hemorrhage	POST	Other - Specific to initial possibly-pregnant cohort (633K)	X	X		X			
433536	Severe pre-eclampsia	PCOMP	Other - Specific to initial possibly-pregnant cohort (633K)	X	X		X			
192679	Renal disease in pregnancy AND/OR puerperium without hypertension	PCOMP	Other - Specific to initial possibly-pregnant cohort (633K)	X	X		X			
444114	Maternal AND/OR fetal condition affecting labor AND/OR delivery		Other - Specific to initial possibly-pregnant cohort (633K)	X	X		X			
4323285	Fetal disorder		Other - Specific to initial possibly-pregnant cohort (633K)	X	X		X			
440158	Venereal disease in mother complicating pregnancy, childbirth AND/OR puerperium	PCOMP	Other - Specific to initial possibly-pregnant cohort (633K)	X	X		X			
4065625	Spinal and epidural anesthesia-induced headache during the puerperium		Other - Specific to initial possibly-pregnant cohort (633K)	X	X		X			
441922	Transient hypertension of pregnancy	PCOMP	Other - Specific to initial possibly-pregnant cohort (633K)	X	X		X			
198221	Pelvic hematoma during delivery	POST	Other - Specific to initial possibly-pregnant cohort (633K)	X	X		X			
440465	Postpartum state	POST	Other - Specific to initial possibly-pregnant cohort (633K)	X	X		X			

437334	Cervical incompetence with antenatal problem	PCOMP	Other - Specific to initial possibly-pregnant cohort (633K)	X	X		X			
321638	Benign essential hypertension complicating pregnancy, childbirth and the puerperium	PCOMP	Other - Specific to initial possibly-pregnant cohort (633K)	X	X		X			
198488	Placental abruption	PCOMP	Other - Specific to initial possibly-pregnant cohort (633K)	X	X		X			
375016	Central nervous system malformation in fetus affecting obstetrical care	PCOMP	Other - Specific to initial possibly-pregnant cohort (633K)	X	X		X			
4149783	Abdominal pain in pregnancy	PCOMP	Other - Specific to initial possibly-pregnant cohort (633K)	X	X		X			
4063043	Pre-existing type 2 diabetes mellitus		Other - Specific to initial possibly-pregnant cohort (633K)	X	X		X			
195877	Finding of gravid uterus	PCONF	Other - Specific to initial possibly-pregnant cohort (633K)	X	X		X			
434111	Oligohydramnios	PCOMP	Other - Specific to initial possibly-pregnant cohort (633K)	X	X		X			
4297232	Postnatal maternal examination	POST	Other - Specific to initial possibly-pregnant cohort (633K)	X	X		X			
141084	Pre-eclampsia or eclampsia with pre-existing hypertension	PCOMP	Other - Specific to initial possibly-pregnant cohort (633K)	X	X		X			
2110304	Cerclage of cervix, during pregnancy; vaginal	PCOMP	Other - Specific to initial possibly-pregnant cohort (633K)	X	X		X			
132685	Severe pre-eclampsia - not delivered	PCOMP	Other - Specific to initial possibly-pregnant cohort (633K)	X	X		X			
196751	Placenta previa with hemorrhage	PCOMP	Other - Specific to initial possibly-pregnant cohort (633K)	X	X		X			
4079835	Antenatal risk factors	PCOMP	Other - Specific to initial possibly-pregnant cohort (633K)	X	X		X			
4087235	Antenatal screening	OTEST	Other - Specific to initial possibly-pregnant cohort (633K)	X	X		X			
313543	Venous complication of pregnancy and/or the puerperium	PCOMP	Other - Specific to initial possibly-pregnant cohort (633K)	X	X		X			
4261510	Normal labor		Other - Specific to initial possibly-pregnant cohort (633K)	X	X		X			
74415	Genitourinary tract infection in pregnancy - not delivered	PCOMP	Other - Specific to initial possibly-pregnant cohort (633K)	X	X		X			
440795	Complication occurring during labor and delivery	LDEL	Other - Specific to initial possibly-pregnant cohort (633K)	X	X		X			
2211756	Ultrasound, pregnant uterus, real time with image documentation, follow-up (eg, re-evaluation of fetal size by measuring standard growth parameters and amniotic fluid volume, re-evaluation of organ system(s) suspected or confirmed to be abnormal on a previous scan), transabdominal approach, per fetus	ULS	Other - Specific to initial possibly-pregnant cohort (633K)	X	X		X			
2211757	Ultrasound, pregnant uterus, real time with image documentation, transvaginal	ULS	Other - Specific to initial possibly-pregnant cohort (633K)	X	X		X			
2211749	Ultrasound, pregnant uterus, real time with image documentation, fetal and maternal evaluation, after first trimester (> or = 14 weeks 0 days), transabdominal approach; single or first gestation	ULS	Other - Specific to initial possibly-pregnant cohort (633K)	X	X		X			
2211755	Ultrasound, pregnant uterus, real time with image documentation, limited (eg, fetal heart beat, placental location, fetal position and/or qualitative amniotic fluid volume), 1 or more fetuses	ULS	Other - Specific to initial possibly-pregnant cohort (633K)	X	X		X			
433270	Cord entanglement without compression	DELIV	Other - Specific to initial possibly-pregnant cohort (633K)	X	X		X			
2101813	Neuraxial labor analgesia/anesthesia for planned vaginal delivery (this includes any repeat subarachnoid needle placement and drug injection and/or any necessary replacement of an epidural catheter during labor)	LDEL	Other - Specific to initial possibly-pregnant cohort (633K)	X	X		X			
74698	Breech presentation	LDEL	Other - Specific to initial possibly-pregnant cohort (633K)	X	X		X			
40756968	Ultrasound, limited, joint or other nonvascular extremity structure(s) (eg, joint space, peri-articular tendon[s], muscle[s], nerve[s], other soft-tissue structure[s], or soft-tissue mass[es]), real-time with image documentation	ULS	Other - Specific to initial possibly-pregnant cohort (633K)	X	X		X			
4061157	Antenatal ultrasound scan abnormal	ULS	Other - Specific to initial possibly-pregnant cohort (633K)	X	X		X			
194702	Premature rupture of membranes	LDEL	Other - Specific to initial possibly-pregnant cohort (633K)	X	X		X			
197938	Uterine inertia	LDEL	Other - Specific to initial possibly-pregnant cohort (633K)	X	X		X			
432975	Trauma to perineum and/or vulva during delivery	DELIV	Other - Specific to initial possibly-pregnant cohort (633K)	X	X		X			
4062557	False labor	LDEL	Other - Specific to initial possibly-pregnant cohort (633K)	X	X		X			
437342	Abnormality of forces of labor	LDEL	Other - Specific to initial possibly-pregnant cohort (633K)	X	X		X			

443435	Primary uterine inertia	LDEL	Other - Specific to initial possibly-pregnant cohort (633K)	X	X		X			
192974	Secondary uterine inertia	LDEL	Other - Specific to initial possibly-pregnant cohort (633K)	X	X		X			
439893	Maternal obesity syndrome	PCOMP	Other - Specific to initial possibly-pregnant cohort (633K)	X	X		X			
2110342	Unlisted procedure, maternity care and delivery	DELIV	Other - Specific to initial possibly-pregnant cohort (633K)	X	X		X			
4152021	Ultrasound scan - obstetric	ULS	Other - Specific to initial possibly-pregnant cohort (633K)	X	X		X			
435325	Umbilical cord complication	LDEL	Other - Specific to initial possibly-pregnant cohort (633K)	X	X		X			
433542	Precipitate labor	LDEL	Other - Specific to initial possibly-pregnant cohort (633K)	X	X		X			
2110286	Fetal monitoring during labor by consulting physician (ie, non-attending physician) with written report; supervision and interpretation	LDEL	Other - Specific to initial possibly-pregnant cohort (633K)	X	X		X			
4273560	Premature labor	LDEL	Other - Specific to initial possibly-pregnant cohort (633K)	X	X		X			
75605	Fetal death, affecting management of mother	SA	Other - Specific to initial possibly-pregnant cohort (633K)	X	X		X			
440161	Indication for care AND/OR intervention in labor AND/OR delivery	LDEL	Other - Specific to initial possibly-pregnant cohort (633K)	X	X		X			
436477	Abnormal products of conception	SA	Other - Specific to initial possibly-pregnant cohort (633K)	X	X		X			
2211752	Ultrasound, pregnant uterus, real time with image documentation, fetal and maternal evaluation plus detailed fetal anatomic examination, transabdominal approach; each additional gestation (List separately in addition to code for primary procedure)	ULS	Other - Specific to initial possibly-pregnant cohort (633K)	X	X		X			
201642	Retained portions of placenta AND/OR membranes without hemorrhage	LDEL	Other - Specific to initial possibly-pregnant cohort (633K)	X	X		X			
438491	Failed medical induction of labor	LDEL	Other - Specific to initial possibly-pregnant cohort (633K)	X	X		X			
197625	Third stage hemorrhage	LDEL	Other - Specific to initial possibly-pregnant cohort (633K)	X	X		X			
439390	Perineal laceration during delivery	LDEL	Other - Specific to initial possibly-pregnant cohort (633K)	X	X		X			
439093	Traumatic lesion during delivery	DELIV	Other - Specific to initial possibly-pregnant cohort (633K)	X	X		X			
139888	Threatened premature labor - not delivered	LDEL	Other - Specific to initial possibly-pregnant cohort (633K)	X	X		X			
4065745	Exhaustion during labor	LDEL	Other - Specific to initial possibly-pregnant cohort (633K)	X	X		X			
4028644	Biochemical pregnancy	SA	Other - Specific to initial possibly-pregnant cohort (633K)	X	X		X			
2004765	Other manually assisted delivery	DELIV	Other - Specific to initial possibly-pregnant cohort (633K)	X	X		X			
441646	Umbilical cord around neck		Other - Specific to initial possibly-pregnant cohort (633K)	X	X		X			
4059697	US scan - fetal maturity	ULS	Other - Specific to initial possibly-pregnant cohort (633K)	X	X		X			
2101806	Anesthesia for vaginal delivery only	LDEL	Other - Specific to initial possibly-pregnant cohort (633K)	X	X		X			
434714	Prolonged second stage of labor	LDEL	Other - Specific to initial possibly-pregnant cohort (633K)	X	X		X			
2110320	Vaginal delivery only, after previous cesarean delivery (with or without episiotomy and/or forceps)	DELIV	Other - Specific to initial possibly-pregnant cohort (633K)	X	X		X			
441362	Prolonged first stage of labor	LDEL	Other - Specific to initial possibly-pregnant cohort (633K)	X	X		X			
2004746	Other artificial rupture of membranes	LDEL	Other - Specific to initial possibly-pregnant cohort (633K)	X	X		X			
434436	Failed trial of labor	LDEL	Other - Specific to initial possibly-pregnant cohort (633K)	X	X		X			
194100	Obstructed labor due to fetal malposition	LDEL	Other - Specific to initial possibly-pregnant cohort (633K)	X	X		X			
440476	Prolapse of cord	DELIV	Other - Specific to initial possibly-pregnant cohort (633K)	X	X		X			
4150970	Medical induction of labor		Other - Specific to initial possibly-pregnant cohort (633K)	X	X		X			
437622	Failed mechanical induction	LDEL	Other - Specific to initial possibly-pregnant cohort (633K)	X	X		X			
440167	Prolonged labor	LDEL	Other - Specific to initial possibly-pregnant cohort (633K)	X	X		X			

435927	Fetal or neonatal effect of complication of labor and/or delivery	LDEL	Other - Specific to initial possibly-pregnant cohort (633K)	X	X		X			
197043	Cervical observation during pregnancy and labor	LDEL	Other - Specific to initial possibly-pregnant cohort (633K)	X	X		X			
2110311	Delivery of placenta (separate procedure)	LDEL	Other - Specific to initial possibly-pregnant cohort (633K)	X	X		X			
4038495	Delivery finding	DELIV	Other - Specific to initial possibly-pregnant cohort (633K)	X	X		X			
4015143	Spontaneous rupture of fetal membranes	LDEL	Other - Specific to initial possibly-pregnant cohort (633K)	X	X		X			
434708	Cord around neck with compression	POST	Other - Specific to initial possibly-pregnant cohort (633K)	X	X		X			
4063306	Labor and delivery complication by meconium in amniotic fluid		Other - Specific to initial possibly-pregnant cohort (633K)	X	X		X			
197343	First degree perineal laceration	POST	Other - Specific to initial possibly-pregnant cohort (633K)	X	X		X			
198492	Second degree perineal laceration	POST	Other - Specific to initial possibly-pregnant cohort (633K)	X	X		X			
2110315	Routine obstetric care including antepartum care, cesarean delivery, and postpartum care	POST	Other - Specific to initial possibly-pregnant cohort (633K)	X	X		X			
433828	Cord tangled or knotted with compression	PCOMP	Other - Specific to initial possibly-pregnant cohort (633K)	X	X		X			
4052737	Compound presentation		Other - Specific to initial possibly-pregnant cohort (633K)	X	X		X			
2211761	Doppler velocimetry, fetal; middle cerebral artery	OTEST	Other - Specific to initial possibly-pregnant cohort (633K)	X	X		X			
2211759	Fetal biophysical profile; without non-stress testing	OTEST	Other - Specific to initial possibly-pregnant cohort (633K)	X	X		X			
2110310	External cephalic version, with or without tocolysis	Not categorized	Other - Specific to initial possibly-pregnant cohort (633K)	X	X		X			
2212714	Hemoglobin or RBCs, fetal, for fetomaternal hemorrhage; rosette	OTEST	Other - Specific to initial possibly-pregnant cohort (633K)	X	X		X			
2211758	Fetal biophysical profile; with non-stress testing	OTEST	Other - Specific to initial possibly-pregnant cohort (633K)	X	X		X			
4129016	Uterine fibroids in pregnancy, childbirth and the puerperium		Other - Specific to initial possibly-pregnant cohort (633K)	X	X		X			
2110283	Fetal contraction stress test	OTEST	Other - Specific to initial possibly-pregnant cohort (633K)	X	X		X			
2110282	Chorionic villus sampling, any method	OTEST	Other - Specific to initial possibly-pregnant cohort (633K)	X	X		X			
3006511	Appearance of Amniotic fluid	AMNIO	Other - Specific to initial possibly-pregnant cohort (633K)	X	X		X			
314099	Abnormal fetal heart rate	PCOMP	Other - Specific to initial possibly-pregnant cohort (633K)	X	X		X			
3022269	Alpha-1-Fetoprotein [Multiple of the median] adjusted in Amniotic fluid	AFP	Other - Specific to initial possibly-pregnant cohort (633K)	X	X		X			
443247	Labor and delivery complicated by fetal heart rate anomaly		Other - Specific to initial possibly-pregnant cohort (633K)	X	X		X			
2211784	Ultrasonic guidance for chorionic villus sampling, imaging supervision and interpretation	OTEST	Other - Specific to initial possibly-pregnant cohort (633K)	X	X		X			
192979	Obstetric high vaginal laceration	POST	Other - Specific to initial possibly-pregnant cohort (633K)	X	X		X			
2101829	Initial prenatal care visit (report at first prenatal encounter with health care professional providing obstetrical care. Report also date of visit and, in a separate field, the date of the last menstrual period [LMP]) (Prenatal)		Other - Specific to initial possibly-pregnant cohort (633K)	X	X		X			
2110236	Ligation or transection of fallopian tube(s), abdominal or vaginal approach, postpartum, unilateral or bilateral, during same hospitalization (separate procedure)	POST	Other - Specific to initial possibly-pregnant cohort (633K)	X	X		X			
436180	Infection of amniotic cavity	PCOMP	Other - Specific to initial possibly-pregnant cohort (633K)	X	X		X			
2101831	Subsequent prenatal care visit (Prenatal) [Excludes: patients who are seen for a condition unrelated to pregnancy or prenatal care (eg, an upper respiratory infection; patients seen for consultation only, not for continuing care)]	AGP	Other - Specific to initial possibly-pregnant cohort (633K)	X	X		X			
2211760	Doppler velocimetry, fetal; umbilical artery	OTEST	Other - Specific to initial possibly-pregnant cohort (633K)	X	X		X			
2101830	Prenatal flow sheet documented in medical record by first prenatal visit (documentation includes at minimum blood pressure, weight, urine protein, uterine size, fetal heart tones, and estimated date of delivery). Report also: date of visit and, in a separate field, the date of the last menstrual period [LMP] (Note: If reporting 0501F Prenatal flow sheet, it is not necessary to report 0500F Initial prenatal care visit) (Prenatal)		Other - Specific to initial possibly-pregnant cohort (633K)	X	X		X			
4021397	Chorioamnionitis		Other - Specific to initial possibly-pregnant cohort (633K)	X	X		X			

3023791	Uterus Fundal height Tape measure	PCONF	Other - Specific to initial possibly-pregnant cohort (633K)	X	X		X				
315881	Vascular lesion of cord		Other - Specific to initial possibly-pregnant cohort (633K)	X	X		X				
2212548	Pregnancy-associated plasma protein-A (PAPP-A)	OTEST	Other - Specific to initial possibly-pregnant cohort (633K)	X	X		X				
4145335	Placental infarct		Other - Specific to initial possibly-pregnant cohort (633K)	X	X		X				
199089	Laceration of cervix - obstetric	POST	Other - Specific to initial possibly-pregnant cohort (633K)	X	X		X				
3019145	Choriogonadotropin.beta subunit free [Mass/volume] in Serum or Plasma	HCG	Other - Specific to initial possibly-pregnant cohort (633K)	X	X		X				
2110301	Curettage, postpartum	POST	Other - Specific to initial possibly-pregnant cohort (633K)	X	X		X				
4198040	Malpresentation of fetus		Other - Specific to initial possibly-pregnant cohort (633K)	X	X		X				
2101832	Postpartum care visit (Prenatal)		Other - Specific to initial possibly-pregnant cohort (633K)	X	X		X				
43530886	Suspected fetal damage from disease in the mother	PCOMP	Other - Specific to initial possibly-pregnant cohort (633K)	X	X		X				
72973	Chromosomal abnormality in fetus affecting obstetrical care	PCOMP	Other - Specific to initial possibly-pregnant cohort (633K)	X	X		X				
200149	Placenta previa with hemorrhage - not delivered	PCOMP	Other - Specific to initial possibly-pregnant cohort (633K)	X	X		X				
4061426	Antenatal care: obstetric risk	AGP	Other - Specific to initial possibly-pregnant cohort (633K)	X	X		X				
40756825	Evaluation of cervicovaginal fluid for specific amniotic fluid protein(s) (eg, placental alpha microglobulin-1 [PAMG-1], placental protein 12 [PP12], alpha-fetoprotein), qualitative, each specimen	OTEST	Other - Specific to initial possibly-pregnant cohort (633K)	X	X		X				
192372	Placenta previa without hemorrhage - not delivered	PCOMP	Other - Specific to initial possibly-pregnant cohort (633K)	X	X		X				
3051741	Pregnancy associated plasma protein A [Mass/volume] in Serum or Plasma	OTEST	Other - Specific to initial possibly-pregnant cohort (633K)	X	X		X				
2110303	Episiotomy or vaginal repair, by other than attending	POST	Other - Specific to initial possibly-pregnant cohort (633K)	X	X		X				
2212334	Fetal fibronectin, cervicovaginal secretions, semi-quantitative	OTEST	Other - Specific to initial possibly-pregnant cohort (633K)	X	X		X				
4053583	Disorder of placenta	PCOMP	Other - Specific to initial possibly-pregnant cohort (633K)	X	X		X				
3004943	Alpha-1-Fetoprotein [Multiple of the median] in Amniotic fluid	POST	Other - Specific to initial possibly-pregnant cohort (633K)	X	X		X				
4060097	Antenatal care: history of infertility	AGP	Other - Specific to initial possibly-pregnant cohort (633K)	X	X		X				
4060239	Antenatal care: multip	AGP	Other - Specific to initial possibly-pregnant cohort (633K)	X	X		X				
200784	Placental condition affecting management of mother	PCOMP	Other - Specific to initial possibly-pregnant cohort (633K)	X	X		X				
2211764	Doppler echocardiography, fetal, pulsed wave and/or continuous wave with spectral display; follow-up or repeat study	OTEST	Other - Specific to initial possibly-pregnant cohort (633K)	X	X		X				
2213255	Tissue culture for non-neoplastic disorders; amniotic fluid or chorionic villus cells	AMNIO	Other - Specific to initial possibly-pregnant cohort (633K)	X	X		X				
4059988	Antenatal care: poor obstetric history	AGP	Other - Specific to initial possibly-pregnant cohort (633K)	X	X		X				
80463	Fetus with chromosomal abnormality with antenatal problem	PCOMP	Other - Specific to initial possibly-pregnant cohort (633K)	X	X		X				
3010296	Alpha-1-Fetoprotein [Mass/volume] in Amniotic fluid	AFP	Other - Specific to initial possibly-pregnant cohort (633K)	X	X		X				
2110284	Fetal non-stress test	OTEST	Other - Specific to initial possibly-pregnant cohort (633K)	X	X		X				
2110313	Antepartum care only; 7 or more visits	AGP	Other - Specific to initial possibly-pregnant cohort (633K)	X	X		X				
200789	Retained placenta, without hemorrhage	POST	Other - Specific to initial possibly-pregnant cohort (633K)	X	X		X				
4173786	Antenatal care	AGP	Other - Specific to initial possibly-pregnant cohort (633K)	X	X		X				
3003262	Estriol (E3).unconjugated [Multiple of the median] in Serum or Plasma	OTEST	Other - Specific to initial possibly-pregnant cohort (633K)	X	X		X				
433540	Polyhydramnios with antenatal problem	PCOMP	Other - Specific to initial possibly-pregnant cohort (633K)	X	X		X				
4062552	Pre-existing hypertensive heart disease complicating pregnancy, childbirth and the puerperium		Other - Specific to initial possibly-pregnant cohort (633K)	X	X		X				

2211762	Echocardiography, fetal, cardiovascular system, real time with image documentation (2D), with or without M-mode recording; follow-up or repeat study	OTEST	Other - Specific to initial possibly-pregnant cohort (633K)	X	X		X			
432967	Anemia during pregnancy - baby not yet delivered	PCOMP	Other - Specific to initial possibly-pregnant cohort (633K)	X	X		X			
4302252	High risk pregnancy care	PCOMP	Other - Specific to initial possibly-pregnant cohort (633K)	X	X		X			
4300078	Velamentous insertion of umbilical cord	PCOMP	Other - Specific to initial possibly-pregnant cohort (633K)	X	X		X			
443929	Postpartum hemorrhage	POST	Other - Specific to initial possibly-pregnant cohort (633K)	X	X		X			
4063307	Labor and delivery complicated by biochemical evidence of fetal stress		Other - Specific to initial possibly-pregnant cohort (633K)	X	X		X			
3021584	Pregnancy associated plasma protein A [Units/volume] in Serum or Plasma	OTEST	Other - Specific to initial possibly-pregnant cohort (633K)	X	X		X			
4034100	Placental insufficiency	PCOMP	Other - Specific to initial possibly-pregnant cohort (633K)	X	X		X			
434701	Anemia in mother complicating pregnancy, childbirth AND/OR puerperium	PCOMP	Other - Specific to initial possibly-pregnant cohort (633K)	X	X		X			
432386	Oligohydramnios with antenatal problem	PCOMP	Other - Specific to initial possibly-pregnant cohort (633K)	X	X		X			
196488	Atonic postpartum hemorrhage	POST	Other - Specific to initial possibly-pregnant cohort (633K)	X	X		X			
80165	Fetal disproportion	PCOMP	Other - Specific to initial possibly-pregnant cohort (633K)	X	X		X			
2110312	Antepartum care only; 4-6 visits	AGP	Other - Specific to initial possibly-pregnant cohort (633K)	X	X		X			
193830	Third degree perineal laceration	POST	Other - Specific to initial possibly-pregnant cohort (633K)	X	X		X			
439403	Abnormal glucose tolerance test during pregnancy - baby not yet delivered	PCOMP	Other - Specific to initial possibly-pregnant cohort (633K)	X	X		X			
192376	Rhesus isoimmunization with antenatal problem	PCOMP	Other - Specific to initial possibly-pregnant cohort (633K)	X	X		X			
4028479	Vaginal abnormality in pregnancy, childbirth and the puerperium		Other - Specific to initial possibly-pregnant cohort (633K)	X	X		X			
4014716	Placental finding	PCOMP	Other - Specific to initial possibly-pregnant cohort (633K)	X	X		X			
2110314	Postpartum care only (separate procedure)	POST	Other - Specific to initial possibly-pregnant cohort (633K)	X	X		X			
4112701	Prenatal examination and care of mother	AGP	Other - Specific to initial possibly-pregnant cohort (633K)	X	X		X			
4060034	Abnormal biochemical finding on antenatal screening of mother		Other - Specific to initial possibly-pregnant cohort (633K)	X	X		X			
4061433	A/N care: multiparous, older than 35 years	AGP	Parity	X	X		X			
4102166	Multiparous	Not categorized	Parity	X	X		X			
437937	Grand multipara	PCONF	Parity	X	X		X			
432695	Post-term pregnancy	POSTT	Post-term	X	X		X			
4086393	Premature delivery	PREM	Preterm pregnancy	X	X		X			
4175637	Premature pregnancy delivered	PREM	Preterm pregnancy	X	X	X	X			
4014295	Single live birth	LB	Singleton pregnancy	X	X	X	X			
4014454	Single stillbirth	SB	Singleton pregnancy	X	X	X	X			
4218813	Third trimester pregnancy	PCONF	Third trimester	X	X		X			
4114637	Forceps delivery	DELIV	Delivery record only (DELIV)	X				X		
4088084	Low forceps delivery	DELIV	Delivery record only (DELIV)	X				X		
4075187	Forceps cephalic delivery	DELIV	Delivery record only (DELIV)	X				X		
4075168	Assisted breech delivery	DELIV	Delivery record only (DELIV)	X				X		
4032762	Forceps application to aftercoming head		Delivery record only (DELIV)	X				X		
2004731	Other specified instrumental delivery		Delivery record only (DELIV)	X				X		
2004730	Other vacuum extraction	LDEL	Delivery record only (DELIV)	X	X	X		X		
2004726	Other total breech extraction	LDEL	Delivery record only (DELIV)	X				X		
2004724	Other partial breech extraction	LDEL	Delivery record only (DELIV)	X				X		
2004707	Other mid forceps operation	POST	Delivery record only (DELIV)	X				X		
442069	Vacuum extractor delivery - delivered	DELIV	Delivery record only (DELIV)	X				X		
440794	Elderly primigravida - delivered	DELIV	Delivery record only (DELIV)	X	X	X		X		
440793	Deliveries by breech extraction	DELIV	Delivery record only (DELIV)	X				X		
440462	Triplet pregnancy - delivered	DELIV	Delivery record only (DELIV)	X				X		
436767	Breech extraction - delivered	DELIV	Delivery record only (DELIV)	X				X		
435018	Twin pregnancy - delivered	DELIV	Delivery record only (DELIV)	X	X	X		X		
434110	Grand multiparity - delivered	DELIV	Delivery record only (DELIV)	X				X		
73537	Breech presentation - delivered	DELIV	Delivery record only (DELIV)	X	X	X		X		
4063163	Multiple delivery, all by cesarean section	DELIV	Delivery record only (DELIV)	X				X		
442082	Multiple pregnancy with malpresentation - delivered	DELIV	Delivery record only (DELIV)	X				X		
4223536	Classical cesarean section		Delivery record only (DELIV)	X				X		
4192676	Born by cesarean section	DELIV	Delivery record only (DELIV)	X	X	X		X		
4167089	Emergency cesarean section	DELIV	Delivery record only (DELIV)	X				X		
4075182	Elective cesarean section	DELIV	Delivery record only (DELIV)	X				X		
4066112	Delivery by emergency cesarean section	DELIV	Delivery record only (DELIV)	X				X		
4066111	Cesarean section - pregnancy at term	FT	Delivery record only (DELIV)	X				X		
4065739	Cesarean section following previous cesarean section		Delivery record only (DELIV)	X				X		
4061457	Delivery by elective cesarean section	DELIV	Delivery record only (DELIV)	X				X		
2110324	Cesarean delivery only, following attempted vaginal delivery after previous cesarean delivery; including postpartum care	POST	Delivery record only (DELIV)	X	X	X		X		
2004802	Other cesarean section of unspecified type	DELIV	Delivery record only (DELIV)	X				X		

2004789	Cesarean section of other specified type	DELIV	Delivery record only (DELIV)	X				X		
4065749	Puerperal endometritis - delivered with postnatal complication	DELIV	Delivery record only (DELIV)	X	X	X		X		
4065618	Secondary postpartum hemorrhage - delivered with postnatal problem	DELIV	Delivery record only (DELIV)	X				X		
4064171	Fetus with hereditary disease - delivered	DELIV	Delivery record only (DELIV)	X				X		
4062571	Fatigue during pregnancy - delivered		Delivery record only (DELIV)	X				X		
4060157	Umbilical cord tight around neck - delivered	DELIV	Delivery record only (DELIV)	X				X		
4058536	Eclampsia with postnatal complication		Delivery record only (DELIV)	X				X		
443521	Failed trial of labor - delivered	DELIV	Delivery record only (DELIV)	X				X		
443294	Obstetric non-purulent mastitis - delivered		Delivery record only (DELIV)	X				X		
443133	Antepartum hemorrhage with coagulation defect - delivered	DELIV	Delivery record only (DELIV)	X				X		
442419	Puerperal cerebrovascular disorder - delivered	DELIV	Delivery record only (DELIV)	X				X		
442418	Puerperal cerebrovascular disorder - delivered with postnatal complication		Delivery record only (DELIV)	X				X		
442090	Placental polyp - delivered with postnatal complication		Delivery record only (DELIV)	X				X		
442089	Obstetric nipple infection - delivered		Delivery record only (DELIV)	X				X		
442080	Obstetric laceration of cervix - delivered	DELIV	Delivery record only (DELIV)	X				X		
442059	Varicose veins of legs in pregnancy and the puerperium - delivered	DELIV	Delivery record only (DELIV)	X				X		
442054	Amniotic fluid pulmonary embolism - delivered		Delivery record only (DELIV)	X				X		
442053	Obstetric blood-clot pulmonary embolism - delivered	DELIV	Delivery record only (DELIV)	X				X		
442049	Obstetric air pulmonary embolism - delivered		Delivery record only (DELIV)	X				X		
441649	Antenatal deep vein thrombosis - delivered	DELIV	Delivery record only (DELIV)	X				X		
441645	Prolapse of cord - delivered	DELIV	Delivery record only (DELIV)	X				X		
441630	History of recurrent miscarriage - delivered	DELIV	Delivery record only (DELIV)	X				X		
441369	Cervical incompetence - delivered	DELIV	Delivery record only (DELIV)	X				X		
441363	Postpartum coagulation defects - delivered with postnatal problem	DELIV	Delivery record only (DELIV)	X				X		
440481	Cracked nipple in pregnancy, the puerperium or lactation - delivered with postnatal complication	POST	Delivery record only (DELIV)	X				X		
439894	Post-term pregnancy - delivered	POSTT	Delivery record only (DELIV)	X				X		
439094	Superficial thrombophlebitis in pregnancy and the puerperium - delivered	DELIV	Delivery record only (DELIV)	X				X		
439092	Cord tangled with compression - delivered	DELIV	Delivery record only (DELIV)	X				X		
439077	Severe pre-eclampsia - delivered with postnatal complication	DELIV	Delivery record only (DELIV)	X	X	X		X		
438490	Severe pre-eclampsia - delivered	DELIV	Delivery record only (DELIV)	X	X	X		X		
438220	Precipitate labor - delivered	DELIV	Delivery record only (DELIV)	X	X	X		X		
437936	Amniotic cavity infection - delivered	DELIV	Delivery record only (DELIV)	X	X	X		X		
437098	Fetal intrauterine distress first noted during labor AND/OR delivery in liveborn infant	POST	Delivery record only (DELIV)	X				X		
437055	Obstetric nipple infection - delivered with postnatal complication		Delivery record only (DELIV)	X	X	X		X		
436766	Varicose veins of perineum and vulva in pregnancy and the puerperium - delivered with postnatal complication		Delivery record only (DELIV)	X				X		
436173	Hyperemesis gravidarum with metabolic disturbance - delivered	DELIV	Delivery record only (DELIV)	X				X		
435610	Varicose veins of perineum and vulva in pregnancy and the puerperium - delivered	DELIV	Delivery record only (DELIV)	X				X		
435609	Vasa previa - delivered	DELIV	Delivery record only (DELIV)	X	X	X		X		
435031	Postnatal deep vein thrombosis - delivered with postnatal complication	DELIV	Delivery record only (DELIV)	X				X		
435020	Short cord - delivered	DELIV	Delivery record only (DELIV)	X	X	X		X		
434715	Cracked nipple in pregnancy, the puerperium or lactation - delivered		Delivery record only (DELIV)	X				X		
434713	Puerperal pyrexia of unknown origin - delivered with postnatal complication	DELIV	Delivery record only (DELIV)	X				X		
434431	Prolonged second stage - delivered	DELIV	Delivery record only (DELIV)	X				X		
434427	Polyhydramnios - delivered	DELIV	Delivery record only (DELIV)	X				X		
433812	Tuberculosis in pregnancy, childbirth and the puerperium - delivered		Delivery record only (DELIV)	X				X		
433274	Oligohydramnios - delivered	DELIV	Delivery record only (DELIV)	X				X		
321367	Vascular lesions of cord - delivered	DELIV	Delivery record only (DELIV)	X				X		
320456	Benign essential hypertension complicating pregnancy, childbirth and the puerperium - delivered with postnatal complication	DELIV	Delivery record only (DELIV)	X				X		
314103	Benign essential hypertension complicating pregnancy, childbirth and the puerperium - delivered	DELIV	Delivery record only (DELIV)	X	X	X		X		
313829	Maternal hypotension syndrome - delivered with postnatal problem	DELIV	Delivery record only (DELIV)	X				X		
201912	Renal hypertension complicating pregnancy, childbirth and the puerperium - delivered with postnatal complication		Delivery record only (DELIV)	X				X		
201368	Obstetric pelvic hematoma - delivered	DELIV	Delivery record only (DELIV)	X				X		
201359	Placenta previa without hemorrhage - delivered	DELIV	Delivery record only (DELIV)	X	X	X		X		
200160	Premature rupture of membranes - delivered	DELIV	Delivery record only (DELIV)	X	X	X		X		
199087	Obstructed labor caused by bony pelvis - delivered	DELIV	Delivery record only (DELIV)	X				X		
198816	Rupture of uterus before labor - delivered	DELIV	Delivery record only (DELIV)	X				X		
198499	Second degree perineal tear during delivery - delivered	DELIV	Delivery record only (DELIV)	X	X	X		X		
198216	Retroverted incarcerated gravid uterus - delivered	DELIV	Delivery record only (DELIV)	X				X		
197048	Obstetric inversion of uterus - delivered with postnatal problem	DELIV	Delivery record only (DELIV)	X	X	X		X		
196762	Primary uterine inertia - delivered	DELIV	Delivery record only (DELIV)	X				X		
196182	Obstructed labor caused by pelvic soft tissues - delivered	DELIV	Delivery record only (DELIV)	X				X		
196170	Liver disorder in pregnancy - delivered	DELIV	Delivery record only (DELIV)	X				X		
195878	Rhesus isoimmunization - delivered	DELIV	Delivery record only (DELIV)	X				X		
194711	Obstructed labor due to fetal malposition - delivered	DELIV	Delivery record only (DELIV)	X				X		
194429	Fourth degree perineal tear during delivery - delivered	DELIV	Delivery record only (DELIV)	X				X		
193539	Placental abruption - delivered	DELIV	Delivery record only (DELIV)	X	X	X		X		
193535	Fetal-maternal hemorrhage - delivered	DELIV	Delivery record only (DELIV)	X				X		
193275	Third degree perineal tear during delivery - delivered	DELIV	Delivery record only (DELIV)	X	X	X		X		
193271	Obstetric pelvic hematoma - delivered with postnatal problem	DELIV	Delivery record only (DELIV)	X				X		
193269	Obstetric high vaginal laceration - delivered	DELIV	Delivery record only (DELIV)	X				X		
193264	Placenta previa with hemorrhage - delivered	DELIV	Delivery record only (DELIV)	X				X		
192971	Pelvic soft tissue abnormality in pregnancy, childbirth and the puerperium - delivered with postnatal complication		Delivery record only (DELIV)	X				X		
192699	Rupture of uterus during and after labor - delivered	DELIV	Delivery record only (DELIV)	X				X		

192387	Obstetric perineal wound disruption - delivered with postnatal complication	POST	Delivery record only (DELIV)	X				X		
192384	Secondary uterine inertia - delivered	DELIV	Delivery record only (DELIV)	X	X	X		X		
141639	Transient hypertension of pregnancy - delivered with postnatal complication	POST	Delivery record only (DELIV)	X				X		
138811	Eclampsia - delivered with postnatal complication	POST	Delivery record only (DELIV)	X				X		
137940	Transient hypertension of pregnancy - delivered	DELIV	Delivery record only (DELIV)	X	X	X		X		
135601	Pre-eclampsia or eclampsia with pre-existing hypertension - delivered	POST	Delivery record only (DELIV)	X				X		
134414	Pre-eclampsia or eclampsia with pre-existing hypertension - delivered with postnatal complication	POST	Delivery record only (DELIV)	X				X		
133816	Eclampsia - delivered	DELIV	Delivery record only (DELIV)	X				X		
81357	Genitourinary tract infection in pregnancy - delivered	DELIV	Delivery record only (DELIV)	X				X		
81086	Breast engorgement in pregnancy, the puerperium or lactation - delivered with postnatal complication	DELIV	Delivery record only (DELIV)	X				X		
80782	Large fetus causing disproportion - delivered	DELIV	Delivery record only (DELIV)	X				X		
80778	Genitourinary tract infection in pregnancy - delivered with postnatal complication	DELIV	Delivery record only (DELIV)	X	X	X		X		
80479	Failure of lactation - delivered with postnatal complication		Delivery record only (DELIV)	X				X		
80474	Shoulder dystocia - delivered	DELIV	Delivery record only (DELIV)	X	X	X		X		
79897	Obstetric non-purulent mastitis - delivered with postnatal complication	DELIV	Delivery record only (DELIV)	X	X	X		X		
79889	Unstable lie - delivered	DELIV	Delivery record only (DELIV)	X				X		
78494	Obstetric breast abscess - delivered with postnatal complication		Delivery record only (DELIV)	X	X	X		X		
77624	Breast engorgement in pregnancy, the puerperium or lactation - delivered	DELIV	Delivery record only (DELIV)	X				X		
77615	High head at term - delivered	DELIV	Delivery record only (DELIV)	X				X		
77347	Suppressed lactation - delivered		Delivery record only (DELIV)	X				X		
77056	Failure of lactation - delivered		Delivery record only (DELIV)	X				X		
76761	Asymptomatic bacteriuria in pregnancy - delivered	DELIV	Delivery record only (DELIV)	X				X		
75882	Deep transverse arrest - delivered	DELIV	Delivery record only (DELIV)	X				X		
74717	Galactorrhea in pregnancy and the puerperium - delivered with postnatal complication	POST	Delivery record only (DELIV)	X				X		
74440	Galactorrhea in pregnancy and the puerperium - delivered	DELIV	Delivery record only (DELIV)	X				X		
74433	Obstetric breast abscess - delivered	DELIV	Delivery record only (DELIV)	X				X		
73538	Hydrocephalic disproportion - delivered	DELIV	Delivery record only (DELIV)	X				X		
73526	Asymptomatic bacteriuria in pregnancy - delivered with postnatal complication		Delivery record only (DELIV)	X				X		
72970	Mixed feto-pelvic disproportion - delivered	DELIV	Delivery record only (DELIV)	X				X		
72697	Fetus with chromosomal abnormality - delivered	DELIV	Delivery record only (DELIV)	X				X		
4194693	Cervical pregnancy		Ectopic pregnancy (ECT)	X				X		
4227267	Cornual pregnancy		Ectopic pregnancy (ECT)	X				X		
2110299	Laparoscopic treatment of ectopic pregnancy; without salpingectomy and/or oophorectomy	ECT_SURG1	Ectopic pregnancy (ECT)	X	X	X		X		
200153	Ovarian pregnancy	ECT	Ectopic pregnancy (ECT)	X	X	X		X		
2004788	Removal of extratubal ectopic pregnancy	ECT_SURG1	Ectopic pregnancy (ECT)	X				X		
4113680	Ruptured tubal pregnancy		Ectopic pregnancy (ECT)	X				X		
2110295	Surgical treatment of ectopic pregnancy; abdominal pregnancy		Ectopic pregnancy (ECT)	X				X		
2110298	Surgical treatment of ectopic pregnancy; cervical, with evacuation		Ectopic pregnancy (ECT)	X				X		
2110296	Surgical treatment of ectopic pregnancy; interstitial, uterine pregnancy requiring total hysterectomy		Ectopic pregnancy (ECT)	X				X		
2110297	Surgical treatment of ectopic pregnancy; interstitial, uterine pregnancy with partial resection of uterus		Ectopic pregnancy (ECT)	X				X		
2110293	Surgical treatment of ectopic pregnancy; tubal or ovarian, requiring salpingectomy and/or oophorectomy, abdominal or vaginal approach	ECT_SURG1	Ectopic pregnancy (ECT)	X	X	X		X		
2110294	Surgical treatment of ectopic pregnancy; tubal or ovarian, without salpingectomy and/or oophorectomy	ECT_SURG1	Ectopic pregnancy (ECT)	X				X		
192379	Legal termination of pregnancy complicated by damage to pelvic organ and/or tissues	SA	Induced abortion (AB)	X				X		
197333	Legal termination of pregnancy complicated by shock	SA	Induced abortion (AB)	X				X		
435868	Incomplete legal termination of pregnancy	SA	Induced abortion (AB)	X				X		
436228	Fetal death due to termination of pregnancy	AB	Induced abortion (AB)	X	X	X		X		
438196	Complete legal termination of pregnancy with delayed or excessive hemorrhage	SA	Induced abortion (AB)	X				X		
439330	Incomplete legal termination of pregnancy with shock		Induced abortion (AB)	X				X		
439883	Legal termination of pregnancy complicated by genital-pelvic infection	SA	Induced abortion (AB)	X				X		
440453	Incomplete legal termination of pregnancy with delayed or excessive hemorrhage	SA	Induced abortion (AB)	X				X		
441073	Complete legal termination of pregnancy	SA	Induced abortion (AB)	X	X	X		X		
441349	Legal termination of pregnancy with complication	SA	Induced abortion (AB)	X				X		
441915	Illegal termination of pregnancy without complication		Induced abortion (AB)	X				X		
442346	Legal termination of pregnancy complicated by embolism		Induced abortion (AB)	X				X		
444077	Legal termination of pregnancy	AB	Induced abortion (AB)	X	X	X		X		
2110331	Induced abortion, by 1 or more intra-amniotic injections (amniocentesis-injections), including hospital admission and visits, delivery of fetus and secundines	AB	Induced abortion (AB)	X				X		
2110332	Induced abortion, by 1 or more intra-amniotic injections (amniocentesis-injections), including hospital admission and visits, delivery of fetus and secundines; with dilation and curettage and/or evacuation	AB	Induced abortion (AB)	X				X		
2110334	Induced abortion, by 1 or more vaginal suppositories (eg, prostaglandin) with or without cervical dilation (eg, laminaria), including hospital admission and visits, delivery of fetus and secundines	AB	Induced abortion (AB)	X	X	X		X		
2110335	Induced abortion, by 1 or more vaginal suppositories (eg, prostaglandin) with or without cervical dilation (eg, laminaria), including hospital admission and visits, delivery of fetus and secundines; with dilation and curettage and/or evacuation	AB	Induced abortion (AB)	X	X	X		X		
2110336	Induced abortion, by 1 or more vaginal suppositories (eg, prostaglandin) with or without cervical dilation (eg, laminaria), including hospital admission and visits, delivery of fetus and secundines; with hysterotomy (failed medical evacuation)		Induced abortion (AB)	X				X		
2721146	Induced abortion, 17 to 24 weeks	AB	Induced abortion (AB)	X				X		
4168396	Dilation of cervix uteri and curettage for termination of pregnancy		Induced abortion (AB)	X				X		

4212941	Aspiration curettage of uterus for termination of pregnancy	AB	Induced abortion (AB)	X				X		
4240605	Termination of pregnancy	AB	Induced abortion (AB)	X				X		
4297250	Termination of pregnancy	AB	Induced abortion (AB)	X	X	X		X		
4299609	Therapeutic termination of pregnancy procedure	SA	Induced abortion (AB)	X				X		
4014455	Twins - one still and one live born	LB	Livebirth (LB)	X	X	X		X		
4014456	Triplets - all live born		Livebirth (LB)	X	X	X		X		
4092289	Livebirth	LB	Livebirth (LB)	X	X	X		X		
4094046	Triplet birth		Livebirth (LB)	X				X		
4101844	Twin birth	DELIV	Livebirth (LB)	X	X	X		X		
4163851	Multiple birth	LB	Livebirth (LB)	X	X	X		X		
434413	Incomplete miscarriage with complication	SA	Spontaneous abortion (SA)	X	X	X	X	X		
434689	Incomplete abortion	SA	Spontaneous abortion (SA)	X						
435000	Complete miscarriage with metabolic disorder		Spontaneous abortion (SA)	X				X		
435318	Complete miscarriage with genital tract or pelvic infection	SA	Spontaneous abortion (SA)	X				X		
436176	Miscarriage without complication	SA	Spontaneous abortion (SA)	X	X	X	X	X		
436742	Complete miscarriage with renal failure		Spontaneous abortion (SA)	X				X		
436748	Incomplete inevitable miscarriage complicated by embolism		Spontaneous abortion (SA)	X				X		
437045	Incomplete miscarriage with embolism		Spontaneous abortion (SA)	X				X		
437046	Complete abortion	SA	Spontaneous abortion (SA)	X						
437618	Incomplete inevitable miscarriage complicated by genital tract and pelvic infection	SA	Spontaneous abortion (SA)	X				X		
437920	Miscarriage complicated by genital-pelvic infection	SA	Spontaneous abortion (SA)	X	X	X		X		
437933	Incomplete miscarriage with damage to pelvic organs or tissues	SA	Spontaneous abortion (SA)	X				X		
438211	Incomplete inevitable miscarriage complicated by delayed or excessive hemorrhage	SA	Spontaneous abortion (SA)	X				X		
438805	Complete miscarriage with shock		Spontaneous abortion (SA)	X				X		
438812	Abortion complicated by genital-pelvic infection	SA	Spontaneous abortion (SA)	X						
439085	Complete inevitable miscarriage complicated by delayed or excessive hemorrhage	SA	Spontaneous abortion (SA)	X				X		
439328	Incomplete legal abortion with no mention of complication	SA	Spontaneous abortion (SA)	X						
439688	Incomplete miscarriage with renal failure		Spontaneous abortion (SA)	X				X		
440454	Abortion without complication	SA	Spontaneous abortion (SA)	X						
440460	Incomplete miscarriage with delayed or excessive hemorrhage	SA	Spontaneous abortion (SA)	X	X	X	X	X		
440776	Abortion with complication	SA	Spontaneous abortion (SA)	X						
441348	Miscarriage complicated by metabolic disorder	SA	Spontaneous abortion (SA)	X				X		
441629	Complete inevitable miscarriage complicated by embolism		Spontaneous abortion (SA)	X				X		
441631	Incomplete miscarriage with shock		Spontaneous abortion (SA)	X				X		
4015162	Twins - both stillborn	SB	Stillbirth (SB)	X	X	X		X		
4063309	Intrauterine death with antenatal problem	SA	Stillbirth (SB)	X				X		
4223536	Classical cesarean section		Cesarean section	X						
4192676	Born by cesarean section	DELIV	Cesarean section	X	X	X				
4167089	Emergency cesarean section	DELIV	Cesarean section	X						
4075182	Elective cesarean section	DELIV	Cesarean section	X						
4066112	Delivery by emergency cesarean section	DELIV	Cesarean section	X						
4066111	Cesarean section - pregnancy at term	FT	Cesarean section	X						
4065739	Cesarean section following previous cesarean section		Cesarean section	X						
4063163	Multiple delivery, all by cesarean section	DELIV	Cesarean section	X						
4061457	Delivery by elective cesarean section	DELIV	Cesarean section	X						
2110324	Cesarean delivery only, following attempted vaginal delivery after previous cesarean delivery; including postpartum care	POST	Cesarean section	X	X	X				
2004802	Other cesarean section of unspecified type	DELIV	Cesarean section	X						
2004789	Cesarean section of other specified type	DELIV	Cesarean section	X						
4167089	Emergency cesarean section	DELIV	Emergency cesarean section	X						
4066112	Delivery by emergency cesarean section	DELIV	Emergency cesarean section	X						
440794	Elderly primigravida - delivered	DELIV	Gravidity	X	X	X				
4061424	Antenatal care: primigravida	PCONF	Gravidity	X						
441647	Continuing pregnancy after intrauterine death of twin fetus	PCOMP	Multiple pregnancy	X						
438213	Delayed delivery of second twin, triplet etc		Multiple pregnancy	X						
4064560	Delayed delivery second twin with antenatal problem		Multiple pregnancy	X						
4196733	Heterozygous twin		Multiple pregnancy	X						
444386	Interlocked twins		Multiple pregnancy	X						
2110337	Multifetal pregnancy reduction(s) (MPR)	PCOMP	Multiple pregnancy	X	X					
4163851	Multiple birth	LB	Multiple pregnancy	X	X	X				
4063163	Multiple delivery, all by caesarean section	DELIV	Multiple pregnancy	X						
75015	Multiple gestation with one OR more fetal malpresentations	PCOMP	Multiple pregnancy	X						
4060542	Multiple pregnancy with malpresentation		Multiple pregnancy	X						
442082	Multiple pregnancy with malpresentation - delivered	DELIV	Multiple pregnancy	X						
4063175	Multiple pregnancy with malpresentation with antenatal problem	PCOMP	Multiple pregnancy	X						
4101844	Twin birth	DELIV	Multiple pregnancy	X	X	X				
435018	Twin pregnancy - delivered	DELIV	Multiple pregnancy	X	X	X				
4015162	Twins - both stillborn	SB	Multiple pregnancy	X	X	X				
4014455	Twins - one still and one live born	LB	Multiple pregnancy	X	X	X				
4094046	Triplet birth		Multiple pregnancy	X						
432375	Triplet pregnancy	PCONF	Multiple pregnancy	X	X					
440462	Triplet pregnancy - delivered	DELIV	Multiple pregnancy	X						
437931	Triplet pregnancy with antenatal problem	PCOMP	Multiple pregnancy	X						
4014456	Triplets - all live born		Multiple pregnancy	X	X	X				
4063158	Complications specific to multiple gestation		Multiple pregnancy	X	X					
4118411	Fetal or neonatal effect of multiple pregnancy		Multiple pregnancy	X	X					
4157006	Ultrasound scan - multiple fetus		Multiple pregnancy	X						
40483581	Combined tubal and intrauterine pregnancy	ECT	Multiple pregnancy	X	X					
442594	Quadruplet pregnancy	PCONF	Multiple pregnancy	X						
440466	Quadruplet pregnancy with antenatal problem	PCONF	Multiple pregnancy	X						
434110	Grand multiparity - delivered	DELIV	Parity	X						
4060243	A/N care: elderly primiparous	AGP	Parity	X	X					
4060101	Antenatal care: primiparous, under 17 years	AGP	Parity	X	X					
439656	Antenatal care: grand multiparity	AGP	Parity	X	X					
442441	Grand multiparity with antenatal problem	PCOMP	Parity	X	X					
439894	Post-term pregnancy - delivered	POSTT	Post-term	X						
434695	Post-term pregnancy - not delivered		Post-term	X	X					

439128	Extreme prematurity of infant	PREM	Preterm pregnancy	X	X					
4149449	Baby premature 24-26 weeks		Preterm pregnancy	X	X					
2514570	Subsequent intensive care, per day, for the evaluation and management of the recovering very low birth weight infant (present body weight less than 1500 grams)	PREM	Preterm pregnancy	X	X					
4058403	Premature separation of placenta with coagulation defect	PREM	Preterm pregnancy	X	X					
4171115	Low birth weight infant	PREM	Preterm pregnancy	X	X					
4195545	Disorder relating to short gestation AND/OR low birthweight	PREM	Preterm pregnancy	X						
432452	Anemia of prematurity	PREM	Preterm pregnancy	X	X					
4151169	Baby birth weight less than 751gm	PREM	Preterm pregnancy	X	X					
72726	Light-for-dates with signs of fetal malnutrition	PREM	Preterm pregnancy	X	X					
440847	Neonatal jaundice associated with preterm delivery	PREM	Preterm pregnancy	X	X					
2514571	Subsequent intensive care, per day, for the evaluation and management of the recovering low birth weight infant (present body weight of 1500-2500 grams)	PREM	Preterm pregnancy	X	X					
4149451	Baby premature 36 weeks		Preterm pregnancy	X	X					
4149457	Baby premature 26-28 weeks		Preterm pregnancy	X	X					
2514572	Subsequent intensive care, per day, for the evaluation and management of the recovering infant (present body weight of 2501-5000 grams)	PREM	Preterm pregnancy	X	X					
4118056	Fetal or neonatal effect of maternal premature rupture of membrane		Preterm pregnancy	X	X					
4147874	Premature infant 28-37 weeks	PREM	Preterm pregnancy	X						
4148097	Fetal or neonatal effect of abruptio placentae		Preterm pregnancy	X						
439402	Abnormal glucose tolerance test during pregnancy - baby delivered	DELIV	Delivery record only (DELIV)	X						
432968	Anemia during pregnancy - baby delivered	DELIV	Delivery record only (DELIV)	X	X					
136755	Thyroid dysfunction during pregnancy - baby delivered	DELIV	Delivery record only (DELIV)	X						
442920	Mental disorder during pregnancy - baby delivered	DELIV	Delivery record only (DELIV)	X	X					
196181	Tumour of uterine body - baby delivered	DELIV	Delivery record only (DELIV)	X	X					
443012	Diabetes mellitus during pregnancy - baby delivered	DELIV	Delivery record only (DELIV)	X	X					
442922	Anaemia in the puerperium - baby delivered during previous episode of care	DELIV	Delivery record only (DELIV)	X						
441361	Delayed delivery after artificial rupture of membranes	DELIV	Delivery record only (DELIV)	X	X					
442919	Mental disorder in the puerperium - baby delivered	DELIV	Delivery record only (DELIV)	X	X					
443246	Vulval abnormality - baby delivered	DELIV	Delivery record only (DELIV)	X						
442915	Drug dependence during pregnancy - baby delivered	DELIV	Delivery record only (DELIV)	X						
193544	Vaginal abnormality - baby delivered	DELIV	Delivery record only (DELIV)	X						
2110318	Subtotal or total hysterectomy after cesarean delivery (List separately in addition to code for primary procedure)	DELIV	Delivery record only (DELIV)	X	X					
435323	Hemorrhage in early pregnancy, delivered	DELIV	Delivery record only (DELIV)	X						
197049	Tumor of uterine body - baby delivered with postpartum complication	DELIV	Delivery record only (DELIV)	X						
4062685	Diabetes mellitus in the puerperium - baby delivered during current episode of care	DELIV	Delivery record only (DELIV)	X						
435606	Maternal syphilis during pregnancy - baby delivered	DELIV	Delivery record only (DELIV)	X						
438206	Maternal rubella during pregnancy - baby delivered	DELIV	Delivery record only (DELIV)	X						
194707	Congenital abnormality of uterus - baby delivered with postpartum complication	DELIV	Delivery record only (DELIV)	X						
4145318	Outcome of delivery	DELIV	Delivery record only (DELIV)	X						
4311671	Episiotomy	DELIV	Delivery record only (DELIV)	X	X					
4078138	Baby normal at birth	DELIV	Delivery record only (DELIV)	X	X					
4264738	Separation of symphysis pubis during delivery	DELIV	Delivery record only (DELIV)	X						
4309425	Dilation and curettage of uterus after delivery	DELIV	Delivery record only (DELIV)	X						
440464	Complication following molar AND/OR ectopic pregnancy	ECT	Ectopic pregnancy (ECT)	X	X					
4212260	Baby full term maturity	FT	Full term	X	X					
4149455	Baby birth weight 2.0-2.5kg	GEST	Gestation period, X weeks (GW)	X	X					
2721012	Med abortion inc all ex drug	AB	Induced abortion (AB)	X						
3038294	Alpha-1-Fetoprotein interpretation [interpretation] in Serum or Plasma	AFP	Other - Matcho et al	X	X					
3040768	Alpha-1-Fetoprotein interpretation [interpretation] in Amniotic fluid	AFP	Other - Matcho et al	X	X					
3051868	Trisomy 21 risk based on maternal age+Alpha-1-Fetoprotein+Choriogonadotropin+Estriol.unconjugated in Fetus	AFP	Other - Matcho et al	X						
4042557	AFP - Alpha-fetoprotein raised	AFP	Other - Matcho et al	X	X					
4041177	Alpha-fetoprotein low	AFP	Other - Matcho et al	X						
4084693	Referral to obstetrics service	AGP	Other - Matcho et al	X	X					
4047845	Antenatal education	AGP	Other - Matcho et al	X						
4038943	O/E - fetal movemnt.diminished	AGP	Other - Matcho et al	X						
4038420	O/E - breech presentation	AGP	Other - Matcho et al	X						
4015304	Fetus size equals dates	AGP	Other - Matcho et al	X						
4061791	A/N care: late booker	AGP	Other - Matcho et al	X	X					
4038937	O/E - transverse lie	AGP	Other - Matcho et al	X						
4060241	A/N care: H/O stillbirth	AGP	Other - Matcho et al	X						
4059989	A/N care: H/O trophoblastic disease	AGP	Other - Matcho et al	X						
2213267	Chromosome analysis, amniotic fluid or chorionic villus, count 15 cells, 1 karyotype, with banding	AMNIO	Other - Matcho et al	X	X					
433880	Amniotic fluid examination abnormal	AMNIO	Other - Matcho et al	X	X					
4140734	Amniocentesis for possible chromosomal abnormality	AMNIO	Other - Matcho et al	X	X					
3030662	Karyotype [Identifier] in Amniotic fluid Nominal	AMNIO	Other - Matcho et al	X	X					
2110280	Amniocentesis; therapeutic amniotic fluid reduction (includes ultrasound guidance)	AMNIO	Other - Matcho et al	X	X					
40760436	Chromosome 13+18+21+X+Y aneuploidy in Amniotic fluid by Fluorescent in situ hybridization (FISH) Nominal	AMNIO	Other - Matcho et al	X	X					
3034139	Acetylcholinesterase [Enzymatic activity/volume] in Amniotic fluid	AMNIO	Other - Matcho et al	X						
2100998	Anesthesia for intraperitoneal procedures in lower abdomen including laparoscopy; amniocentesis	AMNIO	Other - Matcho et al	X						
2212210	Amniotic fluid scan (spectrophotometric)	AMNIO	Other - Matcho et al	X	X					
4061154	Antenatal amniocentesis	AMNIO	Other - Matcho et al	X						
4143646	Diagnostic amniocentesis	AMNIO	Other - Matcho et al	X						
4057149	Amniotic fluid examination	AMNIO	Other - Matcho et al	X						
1300978	Megestrol	CONTRA	Other - Matcho et al	X						
3004501	Glucose [Mass/volume] in Serum or Plasma	DIAB	Other - Matcho et al	X	X					
3009261	Glucose [Presence] in Urine by Test strip	DIAB	Other - Matcho et al	X	X					

3021737	Glucose [Mass/volume] in Serum or Plasma --2 hours post meal	DIAB	Other - Matcho et al	X	X					
3013604	Glucose [Mass/volume] in Serum or Plasma --30 minutes post 75 g glucose PO	DIAB	Other - Matcho et al	X	X					
3039896	Glucose [Mass/volume] in Urine by Automated test strip	DIAB	Other - Matcho et al	X	X					
3037524	Glucose-6-Phosphate dehydrogenase [Enzymatic activity/volume] in Red Blood Cells	DIAB	Other - Matcho et al	X	X					
3035352	Glucose [Mass/volume] in Serum or Plasma --2nd specimen post XXX challenge	DIAB	Other - Matcho et al	X						
3035858	Glucose [Mass/volume] in Serum or Plasma --3rd specimen post XXX challenge	DIAB	Other - Matcho et al	X						
3035125	Glucose [Mass/volume] in Serum or Plasma --1st specimen post XXX challenge	DIAB	Other - Matcho et al	X	X					
3008804	Glucose [Mass/volume] in Serum or Plasma --1.5 hours post 75 g glucose PO	DIAB	Other - Matcho et al	X	X					
3003541	Glucose [Mass/volume] in Serum or Plasma --4 hour specimen	DIAB	Other - Matcho et al	X						
3022548	Glucose [Mass/volume] in Cerebral spinal fluid	DIAB	Other - Matcho et al	X	X					
3021860	Glucose [Mass/volume] in Serum or Plasma --5 hours post 75 g glucose PO	DIAB	Other - Matcho et al	X	X					
3009582	Glucose [Mass/volume] in Serum or Plasma --3 hours post 75 g glucose PO	DIAB	Other - Matcho et al	X	X					
3019210	Glucose [Mass/volume] in Body fluid	DIAB	Other - Matcho et al	X	X					
3021033	Glucose [Presence] in Urine by Test strip --1 hour post 75 g glucose PO	DIAB	Other - Matcho et al	X						
3028247	Glucose [Mass/volume] in Serum or Plasma --30 minutes post dose glucose	DIAB	Other - Matcho et al	X	X					
3003994	Glucose-6-Phosphate dehydrogenase [Enzymatic activity/mass] in Red Blood Cells	DIAB	Other - Matcho et al	X	X					
3006887	Glucose [Mass/volume] in Serum or Plasma --pre dose lactose PO	DIAB	Other - Matcho et al	X	X					
3008358	Insulin [Units/volume] in Serum or Plasma --1.5 hours post 75 g glucose PO	DIAB	Other - Matcho et al	X						
3021635	Glucose [Presence] in Urine by Test strip --30 minutes post 75 g glucose PO	DIAB	Other - Matcho et al	X						
3011424	Glucose [Mass/volume] in Blood by Test strip auto	DIAB	Other - Matcho et al	X	X					
3003412	Glucose [Mass/volume] in Serum or Plasma --30 minutes post dose lactose PO	DIAB	Other - Matcho et al	X						
3002310	Glucose [Mass/volume] in Serum or Plasma --1 hour post dose lactose PO	DIAB	Other - Matcho et al	X						
3025113	Glucose [Mass/volume] in Serum or Plasma --15 minutes post dose lactose PO	DIAB	Other - Matcho et al	X						
3001975	Glucose [Mass/volume] in Serum or Plasma --1.5 hours post dose lactose PO	DIAB	Other - Matcho et al	X						
3009397	Glucose [Mass/volume] in Serum or Plasma --4th specimen post XXX challenge	DIAB	Other - Matcho et al	X						
3019876	Glucose [Mass/volume] in Serum or Plasma --2 hours post dose lactose PO	DIAB	Other - Matcho et al	X						
3026145	Insulin [Units/volume] in Serum or Plasma --2 hours post 75 g glucose PO	DIAB	Other - Matcho et al	X						
3022285	Glucose [Mass/volume] in Serum or Plasma --6 hours post 75 g glucose PO	DIAB	Other - Matcho et al	X	X					
3025866	Glucose [Mass/volume] in Serum or Plasma --post 50 g glucose	DIAB	Other - Matcho et al	X	X					
3033462	Insulin [Units/volume] in Serum or Plasma --1 hour post 75 g glucose PO	DIAB	Other - Matcho et al	X						
3020399	Glucose [Mass/volume] in Urine	DIAB	Other - Matcho et al	X	X					
3040820	Glucose [Mass/volume] in Serum or Plasma --pre-meal	DIAB	Other - Matcho et al	X	X					
4311629	Impaired glucose tolerance	DIAB	Other - Matcho et al	X	X					
4019096	TAH BSO - Total abdominal hysterectomy and bilateral salpingo-oophorectomy	ECT_SURG1	Other - Matcho et al	X	X					
4107080	BSO - Bilateral salpingo-oophorectomy	ECT_SURG1	Other - Matcho et al	X						
4021363	Tubo-oophorectomy	ECT_SURG1	Other - Matcho et al	X	X					
4074424	Right salpingectomy	ECT_SURG1	Other - Matcho et al	X						
4074877	Left salpingectomy	ECT_SURG1	Other - Matcho et al	X						
4072837	Right salpingo-oophorectomy	ECT_SURG1	Other - Matcho et al	X						
4074876	LSO - Left salpingo-oophorectomy	ECT_SURG1	Other - Matcho et al	X						
4200678	Abdominal hysterectomy and left salpingo-oophorectomy	ECT_SURG1	Other - Matcho et al	X						
4093429	Bilateral salpingectomy	ECT_SURG1	Other - Matcho et al	X						
4263669	Salpingostomy	ECT_SURG1	Other - Matcho et al	X						
4001541	Fallopian tube excision	ECT_SURG1	Other - Matcho et al	X						
4074426	Excision of ectopic ovarian pregnancy	ECT_SURG1	Other - Matcho et al	X						
2110253	Salpingostomy (salpingoneostomy)	ECT_SURG1	Other - Matcho et al	X						
2110245	Laparoscopy, surgical; with salpingostomy (salpingoneostomy)	ECT_SURG1	Other - Matcho et al	X						
2110240	Laparoscopy, surgical; with removal of adnexal structures (partial or total oophorectomy and/or salpingectomy)	ECT_SURG1	Other - Matcho et al	X	X					
2004348	Other partial salpingectomy	ECT_SURG1	Other - Matcho et al	X						
2004345	Excision or destruction of lesion of fallopian tube	ECT_SURG1	Other - Matcho et al	X						
2004343	Removal of remaining fallopian tube	ECT_SURG1	Other - Matcho et al	X						
2004342	Removal of both fallopian tubes at same operative episode	ECT_SURG1	Other - Matcho et al	X	X					
2004330	Total unilateral salpingectomy	ECT_SURG1	Other - Matcho et al	X						
4251314	Laparoscopy	ECT_SURG2	Other - Matcho et al	X	X					
4100257	Fimbriectomy of fallopian tube	ECT_SURG2	Other - Matcho et al	X						
4319321	Laparoscopic adhesiolysis	ECT_SURG2	Other - Matcho et al	X	X					
4142956	Diagnostic laparoscopy of female pelvis	ECT_SURG2	Other - Matcho et al	X	X					
4214980	Lysis of adhesions	ECT_SURG2	Other - Matcho et al	X						
2212632	Gonadotropin, chorionic (hCG); qualitative	HCG	Other - Matcho et al	X	X					
3011996	Choriogonadotropin.beta subunit [Moles/volume] in Serum or Plasma	HCG	Other - Matcho et al	X	X					
3011149	Choriogonadotropin.beta subunit (pregnancy test) [Presence] in Serum or Plasma	HCG	Other - Matcho et al	X	X					
3018954	Choriogonadotropin (pregnancy test) [Presence] in Urine	HCG	Other - Matcho et al	X	X					
3009417	Choriogonadotropin.beta subunit (pregnancy test) [Presence] in Urine	HCG	Other - Matcho et al	X	X					
3002091	Choriogonadotropin [Moles/volume] in Serum or Plasma	HCG	Other - Matcho et al	X	X					
3003191	Choriogonadotropin (pregnancy test) [Presence] in Serum or Plasma	HCG	Other - Matcho et al	X	X					

3036988	Choriogonadotropin.intact [Units/volume] in Serum or Plasma	HCG	Other - Matcho et al	X	X					
4042751	Serum total HCG measurement	HCG	Other - Matcho et al	X						
4041161	Serum pregnancy test (B-HCG)	HCG	Other - Matcho et al	X						
4055426	Urine HCG titre	HCG	Other - Matcho et al	X						
192972	Prolonged rupture of membranes	LDEL	Other - Matcho et al	X	X					
434105	Maternal pyrexia during labour	LDEL	Other - Matcho et al	X						
195075	Passage of meconium	LDEL	Other - Matcho et al	X	X					
73267	Breech malpresentation successfully converted to cephalic presentation	LDEL	Other - Matcho et al	X						
2004747	Other surgical induction of labor	LDEL	Other - Matcho et al	X	X					
432976	Obstetrical complication of anaesthesia AND/OR sedation	LDEL	Other - Matcho et al	X	X					
2110287	Fetal monitoring during labor by consulting physician (ie, non-attending physician) with written report; interpretation only	LDEL	Other - Matcho et al	X	X					
312733	Abnormal foetal heart beat, not clear if noted before OR after onset of labour in liveborn infant	LDEL	Other - Matcho et al	X						
193827	Obstructed labor	LDEL	Other - Matcho et al	X	X					
442777	Generalized infection during labor	LDEL	Other - Matcho et al	X	X					
434435	Prolonged first stage with antenatal problem	LDEL	Other - Matcho et al	X						
3002574	Fasting glucose [Presence] in Urine by Test strip	LDEL	Other - Matcho et al	X						
435024	Precipitate labour with antenatal problem	LDEL	Other - Matcho et al	X						
2110281	Cordocentesis (intrauterine), any method	LDEL	Other - Matcho et al	X	X					
197051	Obstructed labour caused by bony pelvis	LDEL	Other - Matcho et al	X						
2004751	Failed forceps	LDEL	Other - Matcho et al	X						
2101809	Anesthesia for cesarean hysterectomy without any labor analgesia/anesthesia care	LDEL	Other - Matcho et al	X	X					
442440	Obstructed labor caused by pelvic soft tissues with antenatal problem	LDEL	Other - Matcho et al	X						
192694	Obstructed labour caused by pelvic soft tissues	LDEL	Other - Matcho et al	X						
194106	Rupture of uterus during AND/OR after labor	LDEL	Other - Matcho et al	X						
4309364	Spontaneous onset of labour	LDEL	Other - Matcho et al	X	X					
4032756	Labour Induction	LDEL	Other - Matcho et al	X	X					
4128030	Birthing procedure	LDEL	Other - Matcho et al	X	X					
4075159	Labour operations	LDEL	Other - Matcho et al	X						
2004811	Other fetal monitoring	OTEST	Other - Matcho et al	X	X					
2004822	Other diagnostic procedures on fetus and amnion	OTEST	Other - Matcho et al	X	X					
3003694	ABO and Rh group [Type] in Blood	OTEST	Other - Matcho et al	X	X					
2212144	Chorionic gonadotropin stimulation panel; testosterone response This panel must include the following: Testosterone (84403 x 2 on three pooled blood samples)	OTEST	Other - Matcho et al	X						
3023524	Rh immune globulin screen [interpretation]	OTEST	Other - Matcho et al	X						
3044819	Karyotype [Identifier] in Chorionic villus sample Nominal	OTEST	Other - Matcho et al	X						
2110285	Fetal scalp blood sampling	OTEST	Other - Matcho et al	X						
2212391	Hemoglobin; F (fetal), qualitative	OTEST	Other - Matcho et al	X	X					
2212439	Fetal lung maturity assessment; lamellar body density	OTEST	Other - Matcho et al	X						
2101804	Anesthesia for external cephalic version procedure	OTEST	Other - Matcho et al	X	X					
4070303	Estriol measurement, serum	OTEST	Other - Matcho et al	X						
4239302	Artificial insemination	OVUL	Other - Matcho et al	X	X					
2213360	Insemination of oocytes	OVUL	Other - Matcho et al	X	X					
4072862	Oocyte recovery	OVUL	Other - Matcho et al	X						
2213348	Culture and fertilization of oocyte(s); with co-culture of embryos	OVUL	Other - Matcho et al	X						
2110196	Artificial insemination; intra-cervical	OVUL	Other - Matcho et al	X						
2213365	Biopsy, oocyte polar body or embryo blastomere, microtechnique (for pre-implantation genetic diagnosis); greater than 5 embryos	OVUL	Other - Matcho et al	X	X					
2110276	Gamete, zygote, or embryo intrafallopian transfer, any method	OVUL	Other - Matcho et al	X						
4199599	Test tube ovum fertilization	OVUL	Other - Matcho et al	X						
4138741	Intrauterine artificial insemination	OVUL	Other - Matcho et al	X						
4145664	IVF - In vitro fertilisation using donor sperm	OVUL	Other - Matcho et al	X						
4144415	Laparoscopic oocyte recovery	OVUL	Other - Matcho et al	X						
4327048	Embryo transfer	Not categorized	Other - Matcho et al	X						
437041	Mother not delivered	PCOMP	Other - Matcho et al	X	X					
440216	Abnormal glucose tolerance test	PCOMP	Other - Matcho et al	X	X					
76756	Breech presentation with antenatal problem	PCOMP	Other - Matcho et al	X	X					
136760	Transient hypertension of pregnancy - not delivered	PCOMP	Other - Matcho et al	X	X					
314090	PET - Mild pre-eclamptic toxaemia	PCOMP	Other - Matcho et al	X	X					
313590	Perinatal respiratory failure	PCOMP	Other - Matcho et al	X	X					
435023	Mild hyperemesis-not delivered	PCOMP	Other - Matcho et al	X						
80205	Perinatal condition	PCOMP	Other - Matcho et al	X	X					
4162865	Abnormal glucose tolerance test during pregnancy, childbirth and the puerperium	PCOMP	Other - Matcho et al	X						
4064172	Fetus with hereditary disease with antenatal problem	PCOMP	Other - Matcho et al	X	X					
192691	Diabetes mellitus during pregnancy - baby not yet delivered	PCOMP	Other - Matcho et al	X	X					
73268	Fetal distress affecting management of mother	PCOMP	Other - Matcho et al	X	X					
138479	Thyroid dysfunction during pregnancy - baby not yet delivered	PCOMP	Other - Matcho et al	X	X					
192385	Tumour of uterine body complicating antenatal care, baby not yet delivered	PCOMP	Other - Matcho et al	X	X					
77338	Malposition and malpresentation of fetus	PCOMP	Other - Matcho et al	X	X					
201083	Fetal AND/OR placental disorder affecting management of mother	PCOMP	Other - Matcho et al	X	X					
200468	Premature rupture of membranes with antenatal problem	PCOMP	Other - Matcho et al	X	X					
4062565	Habitual aborter - not delivered	PCOMP	Other - Matcho et al	X	X					
4027371	Bone AND/OR joint disorder in mother complicating pregnancy, childbirth AND/OR puerperium	PCOMP	Other - Matcho et al	X						
201958	Perinatal dermatological disorders	PCOMP	Other - Matcho et al	X	X					
442565	Gestational edema without hypertension	PCOMP	Other - Matcho et al	X	X					
442918	Mental disorder during pregnancy - baby not yet delivered	PCOMP	Other - Matcho et al	X	X					
440788	Hyperemesis gravidarum with metabolic disturbance - not delivered	PCOMP	Other - Matcho et al	X	X					
316478	Heart disease in mother complicating pregnancy, childbirth AND/OR puerperium	PCOMP	Other - Matcho et al	X	X					
437090	Hemolytic disease of fetus OR newborn due to ABO immunization	PCOMP	Other - Matcho et al	X	X					
438478	Hemorrhage in early pregnancy	PCOMP	Other - Matcho et al	X	X					

78818	Large fetus causing disproportion with antenatal problem	PCOMP	Other - Matcho et al	X						
4064178	Fetus with drug damage with antenatal problem	PCOMP	Other - Matcho et al	X						
4277110	Pre-existing hypertension complicating AND/OR reason for care during pregnancy	PCOMP	Other - Matcho et al	X						
135370	Perinatal cutaneous hemorrhage	PCOMP	Other - Matcho et al	X	X					
81358	Transverse OR oblique presentation of fetus	PCOMP	Other - Matcho et al	X	X					
436219	Fetus or neonate affected by maternal infection	PCOMP	Other - Matcho et al	X	X					
441682	Fetus OR newborn affected by compression of umbilical cord	PCOMP	Other - Matcho et al	X	X					
80156	Asymptomatic bacteriuria in pregnancy	PCOMP	Other - Matcho et al	X	X					
433266	Infective/parasitic disease in preg/childbirth/puerperium	PCOMP	Other - Matcho et al	X						
258564	Perinatal interstitial emphysema	PCOMP	Other - Matcho et al	X	X					
72374	Unstable lie with antenatal problem	PCOMP	Other - Matcho et al	X						
201350	Placental abruption - not delivered	PCOMP	Other - Matcho et al	X	X					
442772	Isoimmunization from non-ABO, non-Rh blood-group incompatibility affecting pregnancy	PCOMP	Other - Matcho et al	X	X					
193825	Congenital abnormality of uterus complicating antenatal care, baby not yet delivered	PCOMP	Other - Matcho et al	X	X					
197339	Congenital abnormality of uterus, affecting pregnancy	PCOMP	Other - Matcho et al	X	X					
433027	Foetus affected by maternal toxaemia	PCOMP	Other - Matcho et al	X	X					
136743	Pre-eclampsia or eclampsia with pre-existing hypertension - not delivered	PCOMP	Other - Matcho et al	X						
195019	Pelvic soft tissue abnormality in pregnancy, childbirth and the puerperium with antenatal problem	PCOMP	Other - Matcho et al	X	X					
79890	Disorder of pelvic size and disproportion	PCOMP	Other - Matcho et al	X	X					
442913	Drug dependence during pregnancy - baby not yet delivered	PCOMP	Other - Matcho et al	X						
433603	Hemolytic disease - Rh	PCOMP	Other - Matcho et al	X	X					
314426	Congenital cardiovascular disorder during pregnancy - baby not yet delivered	PCOMP	Other - Matcho et al	X	X					
435887	Antepartum deep phlebothrombosis	PCOMP	Other - Matcho et al	X	X					
4063183	Mixed feto-pelvic disproportion	PCOMP	Other - Matcho et al	X						
443292	Liver disorder in pregnancy - not delivered	PCOMP	Other - Matcho et al	X						
133284	Impetigo herpetiformis	PCOMP	Other - Matcho et al	X						
435880	Amniotic cavity infection with antenatal problem	PCOMP	Other - Matcho et al	X	X					
4064175	Fetus with viral damage via mother with antenatal problem	PCOMP	Other - Matcho et al	X						
72696	High head at term with antenatal problem	PCOMP	Other - Matcho et al	X						
78210	Face OR brow presentation of fetus	PCOMP	Other - Matcho et al	X						
442088	High head at term	PCOMP	Other - Matcho et al	X	X					
76491	Obstetric non-purulent mastitis with antenatal complication	PCOMP	Other - Matcho et al	X	X					
4064169	Fetus with hereditary disease	PCOMP	Other - Matcho et al	X	X					
434712	Varicose veins of legs in pregnancy and the puerperium with antenatal complication	PCOMP	Other - Matcho et al	X	X					
442290	Varicose veins of legs complicating pregnancy AND/OR puerperium	PCOMP	Other - Matcho et al	X						
196486	Retroverted incarcerated gravid uterus with antenatal problem	PCOMP	Other - Matcho et al	X						
43530888	Suspected fetal damage from radiation	PCOMP	Other - Matcho et al	X						
192684	Renal hypertension complicating pregnancy, childbirth and the puerperium - not delivered	PCOMP	Other - Matcho et al	X						
137613	Eclampsia in pregnancy	PCOMP	Other - Matcho et al	X	X					
4061971	Umbilical cord tight around neck with antenatal problem	PCOMP	Other - Matcho et al	X						
194598	Neoplasm of uncertain behaviour of placenta	PCOMP	Other - Matcho et al	X	X					
435331	Superficial thrombophlebitis in pregnancy and the puerperium with antenatal complication	PCOMP	Other - Matcho et al	X						
197608	Peripheral neuritis in pregnancy	PCOMP	Other - Matcho et al	X	X					
43022052	Fetal anemia	PCOMP	Other - Matcho et al	X						
317941	Phlebitis AND/OR thrombosis complicating pregnancy AND/OR puerperium	PCOMP	Other - Matcho et al	X						
79146	Foetal blood loss	PCOMP	Other - Matcho et al	X						
196757	Fetal-maternal haemorrhage with antenatal problem	PCOMP	Other - Matcho et al	X						
443700	Eclampsia	PCOMP	Other - Matcho et al	X	X					
79091	Unstable lie	PCOMP	Other - Matcho et al	X	X					
443323	Papyraceous fetus - not delivered	PCOMP	Other - Matcho et al	X						
73282	Breast engorgement in pregnancy, the puerperium or lactation with antenatal complication	PCOMP	Other - Matcho et al	X						
441929	Varicose veins of perineum and vulva in pregnancy and the puerperium with antenatal complication	PCOMP	Other - Matcho et al	X						
437341	Failed mechanical induction with antenatal problem	PCOMP	Other - Matcho et al	X						
433545	Obstetric blood-clot pulmonary embolism with antenatal complication	PCOMP	Other - Matcho et al	X						
435021	Vasa praevia with antenatal problem	PCOMP	Other - Matcho et al	X						
81360	Fetus papyraceous	PCOMP	Other - Matcho et al	X						
75639	Fetal malnutrition	PCOMP	Other - Matcho et al	X	X					
313272	Puerperal cerebrovascular disorder with antenatal complication	PCOMP	Other - Matcho et al	X						
76772	Galactorrhea in pregnancy and the puerperium with antenatal complication	PCOMP	Other - Matcho et al	X						
2110340	Unlisted fetal invasive procedure, including ultrasound guidance	PCOMP	Other - Matcho et al	X	X					
444374	Persistent occipitoposterior position	PCOMP	Other - Matcho et al	X						
81923	Hydrocephalic disproportion with antenatal problem	PCOMP	Other - Matcho et al	X						
78218	Obstetric breast abscess with antenatal complication	PCOMP	Other - Matcho et al	X	X					
435883	Prolapse of cord with antenatal problem	PCOMP	Other - Matcho et al	X						
201091	Disproportion - major pelvic abnormality	PCOMP	Other - Matcho et al	X						
201366	Fetal-maternal hemorrhage	PCOMP	Other - Matcho et al	X	X					
2211782	Ultrasonic guidance for intrauterine fetal transfusion or cordocentesis, imaging supervision and interpretation	PCOMP	Other - Matcho et al	X	X					
4321386	Cervical cerclage suture present	PCOMP	Other - Matcho et al	X	X					
2110288	Transabdominal amnioinfusion, including ultrasound guidance	PCOMP	Other - Matcho et al	X	X					
438489	Antepartum hemorrhage associated with coagulation defect	PCOMP	Other - Matcho et al	X	X					
77662	Immune hydrops fetalis	PCOMP	Other - Matcho et al	X						
436768	Amniotic fluid pulmonary embolism with antenatal complication	PCOMP	Other - Matcho et al	X						
75608	Shoulder dystocia with antenatal problem	PCOMP	Other - Matcho et al	X						
315884	Vascular lesions of cord with antenatal problem	PCOMP	Other - Matcho et al	X						
4060672	Fetus with drug damage	PCOMP	Other - Matcho et al	X						

2110305	Cerclage of cervix, during pregnancy; abdominal	PCOMP	Other - Matcho et al	X						
435616	Amniotic fluid embolism	PCOMP	Other - Matcho et al	X	X					
441926	Obstetric nipple infection with antenatal complication	PCOMP	Other - Matcho et al	X						
4199931	Morning sickness	PCOMP	Other - Matcho et al	X	X					
4058246	Iron deficiency anemia of pregnancy	PCOMP	Other - Matcho et al	X	X					
4082504	Pruritus gravidarum	PCOMP	Other - Matcho et al	X	X					
4284028	Placenta previa	PCOMP	Other - Matcho et al	X	X					
4034094	Moderate proteinuric hypertension of pregnancy	PCOMP	Other - Matcho et al	X						
4062574	Pregnancy-related glycosuria	PCOMP	Other - Matcho et al	X						
4154997	Amniotic fluid leaking	PCOMP	Other - Matcho et al	X	X					
4170118	Cystitis of pregnancy	PCOMP	Other - Matcho et al	X						
4071437	Face presentation	PCOMP	Other - Matcho et al	X						
4311256	Pemphigoid gestationis	PCOMP	Other - Matcho et al	X	X					
4016059	Maternal alcohol abuse	PCOMP	Other - Matcho et al	X						
4023420	Intra-amniotic infection of fetus	PCOMP	Other - Matcho et al	X						
197031	Intrauterine pregnancy	PCONF	Other - Matcho et al	X	X					
196484	Retroverted incarcerated gravid uterus	PCONF	Other - Matcho et al	X						
192380	Failed attempted abortion without complication	PCONF	Other - Matcho et al	X	X					
4030415	Illegitimate pregnancy, life event	PCONF	Other - Matcho et al	X						
40760833	Birth plurality of Pregnancy	PCONF	Other - Matcho et al	X	X					
4139126	Braxton Hicks' contraction	PCONF	Other - Matcho et al	X						
4059985	Pregnancy unplanned ? wanted	PCONF	Other - Matcho et al	X						
4059982	Pregnant - blood test confirms	PCONF	Other - Matcho et al	X						
4061686	Pregnant - on history	PCONF	Other - Matcho et al	X						
4059984	Pregnant - planned	PCONF	Other - Matcho et al	X						
4060237	Pregnant - unplanned - wanted	PCONF	Other - Matcho et al	X						
4059981	Pregnant - urine test confirms	PCONF	Other - Matcho et al	X						
4061785	Pregnant -unplanned-not wanted	PCONF	Other - Matcho et al	X						
4061672	Pregnant, IUD failure	PCONF	Other - Matcho et al	X						
4042412	Serum pregnancy test positive	PCONF	Other - Matcho et al	X	X					
4129023	Teenage pregnancy	PCONF	Other - Matcho et al	X	X					
4041878	Urine pregnancy test positive	PCONF	Other - Matcho et al	X	X					
435656	Neonatal jaundice	POST	Other - Matcho et al	X	X					
433315	Feeding difficulties in newborn	POST	Other - Matcho et al	X	X					
2514555	Initial hospital or birthing center care, per day, for evaluation and management of normal newborn infant	POST	Other - Matcho et al	X	X					
42739013	Subsequent hospital care, for the evaluation and management of a normal newborn, per day	POST	Other - Matcho et al	X						
2514557	Subsequent hospital care, per day, for evaluation and management of normal newborn	POST	Other - Matcho et al	X	X					
2721181	NEWBORN METABOLIC SCREENING PANEL, INCLUDES TEST KIT, POSTAGE AND THE LABORATORY TESTS SPECIFIED BY THE STATE FOR INCLUSION IN THIS PANEL (E.G. GALACTOSE; HEMOGLOBIN, ELECTROPHORESIS; HYDROXYPROGESTERONE, 17-D; PHENYLANINE (PKU); AND THYROXINE, TOTAL)	POST	Other - Matcho et al	X	X					
4259636	Well child visit, newborn	POST	Other - Matcho et al	X						
2514559	Attendance at delivery (when requested by the delivering physician) and initial stabilization of newborn	POST	Other - Matcho et al	X	X					
42739562	Attendance at delivery (when requested by delivering physician) and initial stabilization of newborn	POST	Other - Matcho et al	X						
258866	Respiratory distress syndrome in the newborn	POST	Other - Matcho et al	X	X					
4132649	Well child visit	POST	Other - Matcho et al	X	X					
201136	Infection of navel cord	POST	Other - Matcho et al	X	X					
314479	Primary apnea in the newborn	POST	Other - Matcho et al	X	X					
321683	TTN - Transient tachypnoea of newborn	POST	Other - Matcho et al	X	X					
2514575	Home visit for postnatal assessment and follow-up care	POST	Other - Matcho et al	X	X					
42739014	History and examination of the normal newborn infant, including the preparation of medical records. (This code should only be used for newborns assessed and discharged from the hospital or birthing room on the same date.)	POST	Other - Matcho et al	X						
2514569	Initial hospital care, per day, for the evaluation and management of the neonate, 28 days of age or less, who requires intensive observation, frequent interventions, and other intensive care services	POST	Other - Matcho et al	X	X					
23034	Neonatal hypoglycemia	POST	Other - Matcho et al	X	X					
2514558	Initial hospital or birthing center care, per day, for evaluation and management of normal newborn infant admitted and discharged on the same date	POST	Other - Matcho et al	X	X					
2514564	Subsequent inpatient neonatal critical care, per day, for the evaluation and management of a critically ill neonate, 28 days of age or less	POST	Other - Matcho et al	X	X					
2514576	Home visit for newborn care and assessment	POST	Other - Matcho et al	X	X					
440840	Neonatal candidiasis	POST	Other - Matcho et al	X	X					
2514563	Initial inpatient neonatal critical care, per day, for the evaluation and management of a critically ill neonate, 28 days of age or less	POST	Other - Matcho et al	X	X					
74469	Light-for-dates without mention of fetal malnutrition	POST	Other - Matcho et al	X	X					
437947	Complication of obstetrical surgical wound	POST	Other - Matcho et al	X	X					
439129	Fetus OR newborn affected by breech delivery AND extraction	POST	Other - Matcho et al	X	X					
4048293	Delayed conjugation causing neonatal jaundice associated with another disorder	POST	Other - Matcho et al	X	X					
439137	Neonatal jaundice due to delayed conjugation	POST	Other - Matcho et al	X						
437985	Disturbance of temperature regulation of newborn	POST	Other - Matcho et al	X	X					
313023	Neonatal chronic respiratory disease	POST	Other - Matcho et al	X	X					
73546	Disorder of breast associated with childbirth	POST	Other - Matcho et al	X	X					
318247	Apnea in the newborn	POST	Other - Matcho et al	X	X					
436234	Transitory neonatal electrolyte disturbance	POST	Other - Matcho et al	X	X					
439156	Observation of neonate	POST	Other - Matcho et al	X	X					
443522	Neonatal bradycardia	POST	Other - Matcho et al	X	X					
434744	Fetus or neonate affected by maternal medical problem	POST	Other - Matcho et al	X	X					
74716	Galactorrhea associated with childbirth	POST	Other - Matcho et al	X						
314478	Abnormal foetal heart beat first noted during labour AND/OR delivery in liveborn infant	POST	Other - Matcho et al	X						
141370	Toxic erythema	POST	Other - Matcho et al	X	X					
134760	Cyanotic attacks of newborn	POST	Other - Matcho et al	X	X					
435076	Transient neonatal thrombocytopenia	POST	Other - Matcho et al	X	X					

200524	Perinatal digestive system disorders	POST	Other - Matcho et al	X	X					
260212	Perinatal atelectasis	POST	Other - Matcho et al	X	X					
76521	Fetus OR newborn affected by malpresentation, malposition AND/OR disproportion during labor AND/OR delivery	POST	Other - Matcho et al	X	X					
4065753	Puerperal septicemia with postnatal complication	POST	Other - Matcho et al	X						
437374	Neonatal aspiration of meconium	POST	Other - Matcho et al	X	X					
439139	Infections specific to perinatal period	POST	Other - Matcho et al	X	X					
440218	Haemolytic disease of foetus OR newborn due to isoimmunisation	POST	Other - Matcho et al	X	X					
436519	Perinatal intraventricular hemorrhage	POST	Other - Matcho et al	X	X					
436534	Hypothermia of newborn	POST	Other - Matcho et al	X	X					
432396	Cracked nipple in pregnancy, the puerperium or lactation with postnatal complication	POST	Other - Matcho et al	X	X					
434474	Newborn late metabolic acidosis	POST	Other - Matcho et al	X	X					
4162866	Abnormal glucose tolerance test in the puerperium - baby delivered during previous episode of care	POST	Other - Matcho et al	X						
373870	Neonatal dacryocystitis and conjunctivitis	POST	Other - Matcho et al	X	X					
439136	Asphyxia, in liveborn infant	POST	Other - Matcho et al	X	X					
435369	Fetal intrauterine distress, not clear if noted before OR after onset of labour in liveborn infant	POST	Other - Matcho et al	X						
443618	Hypoxaemia in newborn	POST	Other - Matcho et al	X	X					
4047937	Neonatal urinary tract infection	POST	Other - Matcho et al	X	X					
258554	Primary atelectasis	POST	Other - Matcho et al	X	X					
4207827	Newborn regurgitation of feed	POST	Other - Matcho et al	X	X					
76770	Suppressed lactation with postnatal complication	POST	Other - Matcho et al	X						
380533	Neonatal seizures	POST	Other - Matcho et al	X	X					
2514556	Initial care, per day, for evaluation and management of normal newborn infant seen in other than hospital or birthing center	POST	Other - Matcho et al	X	X					
194158	Perinatal gastrointestinal hemorrhage	POST	Other - Matcho et al	X	X					
2110322	Routine obstetric care including antepartum care, cesarean delivery, and postpartum care, following attempted vaginal delivery after previous cesarean delivery	POST	Other - Matcho et al	X	X					
2110339	Removal of cerclage suture under anesthesia (other than local)	POST	Other - Matcho et al	X	X					
433029	Perinatal jaundice from excessive hemolysis	POST	Other - Matcho et al	X	X					
319138	Respiratory condition of fetus OR newborn	POST	Other - Matcho et al	X	X					
201957	Necrotizing enterocolitis in fetus OR newborn	POST	Other - Matcho et al	X						
440829	Fetus and newborn affected by noxious influences transmitted via placenta or breast milk	POST	Other - Matcho et al	X	X					
81685	Fracture of clavicle due to birth trauma	POST	Other - Matcho et al	X	X					
434154	Neonatal aspiration syndromes	POST	Other - Matcho et al	X	X					
443523	Neonatal tachycardia	POST	Other - Matcho et al	X	X					
381952	Injury to brachial plexus as birth trauma	POST	Other - Matcho et al	X	X					
443293	Obstetric breast abscess with postnatal complication	POST	Other - Matcho et al	X						
4062811	Benign essential hypertension complicating pregnancy, childbirth and the puerperium with postnatal complication	POST	Other - Matcho et al	X						
138484	Thyroid dysfunction in the puerperium - baby delivered during previous episode of care	POST	Other - Matcho et al	X	X					
433309	Fetus OR newborn affected by alcohol transmitted via placenta AND/OR breast milk	POST	Other - Matcho et al	X	X					
432389	Puerperal pyrexia of unknown origin with postnatal complication	POST	Other - Matcho et al	X	X					
4057976	Severe pre-eclampsia with postnatal complication	POST	Other - Matcho et al	X	X					
78224	Failure of lactation with postnatal complication	POST	Other - Matcho et al	X						
193174	Cystic fibrosis with meconium ileus	POST	Other - Matcho et al	X	X					
312383	Postpartum cardiomyopathy	POST	Other - Matcho et al	X	X					
377980	Cerebral irritability in newborn	POST	Other - Matcho et al	X	X					
201681	Fetus OR newborn affected by placental separation AND/OR hemorrhage	POST	Other - Matcho et al	X	X					
74434	Infection of the breast AND/OR nipple associated with childbirth	POST	Other - Matcho et al	X						
197337	Second degree perineal tear during delivery with postnatal problem	POST	Other - Matcho et al	X						
374748	Perinatal anoxic-ischemic brain injury	POST	Other - Matcho et al	X	X					
443897	Disruption of perineal wound in the puerperium	POST	Other - Matcho et al	X						
440211	Fetus OR newborn affected by morphologic abnormality of placenta	POST	Other - Matcho et al	X	X					
439140	Neonatal polycythemia	POST	Other - Matcho et al	X	X					
439149	Transient neonatal neutropenia	POST	Other - Matcho et al	X	X					
432734	Fetal OR neonatal hemorrhage	POST	Other - Matcho et al	X						
4062686	Diabetes mellitus in the pueperium - baby delivered during previous episode of care	POST	Other - Matcho et al	X						
438541	Fetus OR newborn affected by abnormality of chorion	POST	Other - Matcho et al	X						
438869	Perinatal jaundice from hereditary hemolytic anemia	POST	Other - Matcho et al	X						
442631	Abnormal cerebral signs in the newborn	POST	Other - Matcho et al	X	X					
196177	Genital tract AND/OR pelvic infection following molar AND/OR ectopic pregnancy	POST	Other - Matcho et al	X	X					
435917	Fetus OR newborn affected by incompetent cervix	POST	Other - Matcho et al	X						
193591	Meconium ileus	POST	Other - Matcho et al	X	X					
4061463	Puerperal endometritis with postnatal complication	POST	Other - Matcho et al	X						
443330	Breast engorgement in pregnancy, the puerperium or lactation	POST	Other - Matcho et al	X	X					
438259	Umbilical hemorrhage after birth	POST	Other - Matcho et al	X						
133594	Bacterial sepsis of newborn	POST	Other - Matcho et al	X	X					
81088	Failed lactation	POST	Other - Matcho et al	X	X					
137974	Abnormal fetal heart beat noted before labor in liveborn infant	POST	Other - Matcho et al	X	X					
73019	Breast engorgement in newborn	POST	Other - Matcho et al	X	X					
441964	Conditions involving the integument AND/OR temperature regulation of fetus OR newborn	POST	Other - Matcho et al	X	X					
434155	Intraventricular (nontraumatic) haemorrhage, grade 3, of foetus and newborn	POST	Other - Matcho et al	X	X					
433311	Fetal intrauterine distress noted before labor in liveborn infant	POST	Other - Matcho et al	X						
441412	Edema of newborn	POST	Other - Matcho et al	X	X					
440473	Major puerperal infection	POST	Other - Matcho et al	X						
434482	Diethylstilbestrol poisoning	POST	Other - Matcho et al	X	X					
79098	Suppressed lactation	POST	Other - Matcho et al	X	X					
195064	Perinatal jaundice due to hepatocellular damage	POST	Other - Matcho et al	X	X					

443263	Obstetric breast abscess	POST	Other - Matcho et al	X							
443328	Galactorrhoea in pregnancy and the puerperium with postnatal complication	POST	Other - Matcho et al	X							
437976	Fetus OR newborn affected by maternal anesthesia AND/OR analgesia	POST	Other - Matcho et al	X	X						
2108149	Catheterization of umbilical vein for diagnosis or therapy, newborn	POST	Other - Matcho et al	X	X						
433837	Obstetric nipple infection with postnatal complication	POST	Other - Matcho et al	X	X						
76533	Non-immune hydrops fetalis	POST	Other - Matcho et al	X	X						
4062264	Postnatal deep vein thrombosis with postnatal complication	POST	Other - Matcho et al	X							
436532	Congenital cytomegalovirus infection	POST	Other - Matcho et al	X	X						
198870	Transitory ileus of newborn	POST	Other - Matcho et al	X	X						
257375	Neonatal pulmonary hemorrhage	POST	Other - Matcho et al	X	X						
432443	Disseminated intravascular coagulation in newborn	POST	Other - Matcho et al	X	X						
199925	Perinatal intestinal perforation	POST	Other - Matcho et al	X	X						
436517	Fetus OR newborn affected by narcotic transmitted via placenta AND/OR breast milk	POST	Other - Matcho et al	X							
435026	Puerperal pulmonary embolism	POST	Other - Matcho et al	X							
260841	Perinatal subarachnoid hemorrhage	POST	Other - Matcho et al	X	X						
199097	Third degree perineal tear during delivery with postnatal problem	POST	Other - Matcho et al	X	X						
201129	Newborn swallowing maternal blood causing hematemesis or melena	POST	Other - Matcho et al	X	X						
195014	Renal failure following molar AND/OR ectopic pregnancy	POST	Other - Matcho et al	X							
438820	Postpartum deep phlebothrombosis	POST	Other - Matcho et al	X	X						
434759	Moderate birth asphyxia	POST	Other - Matcho et al	X							
76763	Obstetric trauma damaging pelvic joints and ligaments	POST	Other - Matcho et al	X							
438823	Retracted nipple in pregnancy, the puerperium or lactation with postnatal complication	POST	Other - Matcho et al	X							
435359	Drug reaction AND/OR intoxication specific to newborn	POST	Other - Matcho et al	X							
4116344	Eclampsia in puerperium	POST	Other - Matcho et al	X	X						
432427	Severe birth asphyxia	POST	Other - Matcho et al	X							
4169915	Vomiting in newborn	POST	Other - Matcho et al	X	X						
437060	Obstetric blood-clot pulmonary embolism with postnatal complication	POST	Other - Matcho et al	X							
2004846	Obstetric tamponade of uterus or vagina	POST	Other - Matcho et al	X							
433018	Fetus OR newborn affected by prolapsed cord	POST	Other - Matcho et al	X	X						
198496	Fourth degree perineal tear during delivery with postnatal problem	POST	Other - Matcho et al	X							
443249	Congenital cardiovascular disorders during pregnancy, childbirth and the puerperium	POST	Other - Matcho et al	X	X						
72377	Shoulder girdle dystocia	POST	Other - Matcho et al	X	X						
193323	Neonatal diabetes mellitus	POST	Other - Matcho et al	X	X						
196475	Damage to pelvic organs AND/OR tissues following molar AND/OR ectopic pregnancy	POST	Other - Matcho et al	X							
2004862	Other obstetric operations	POST	Other - Matcho et al	X							
4061341	Postpartum coagulation defects with postnatal problem	POST	Other - Matcho et al	X							
2106488	Laryngoscopy direct, with or without tracheoscopy; diagnostic, newborn	POST	Other - Matcho et al	X	X						
435358	HDN - Haemorrhagic disease of newborn	POST	Other - Matcho et al	X							
2101808	Anesthesia for urgent hysterectomy following delivery	POST	Other - Matcho et al	X							
442148	Fetus OR newborn affected by toxic substance transmitted via placenta AND/OR breast milk	POST	Other - Matcho et al	X	X						
443243	Cracked nipple in pregnancy, the puerperium or lactation	POST	Other - Matcho et al	X							
439934	Meconium aspiration syndrome	POST	Other - Matcho et al	X							
4297611	Puerperal pelvic sepsis	POST	Other - Matcho et al	X							
76221	Neonatal infective mastitis	POST	Other - Matcho et al	X							
318856	Neonatal respiratory arrest	POST	Other - Matcho et al	X	X						
436529	Hemorrhage of newborn	POST	Other - Matcho et al	X	X						
2110084	Incision and drainage of vaginal hematoma; obstetrical/postpartum	POST	Other - Matcho et al	X							
433604	Transient neonatal disorder of coagulation	POST	Other - Matcho et al	X	X						
433832	Obstetric blood-clot pulmonary embolism	POST	Other - Matcho et al	X							
437061	Retracted nipple in pregnancy, the puerperium or lactation	POST	Other - Matcho et al	X							
4143293	Superficial thrombophlebitis in the puerperium	POST	Other - Matcho et al	X	X						
200472	Obstetric high vaginal laceration with postnatal problem	POST	Other - Matcho et al	X							
137099	Neonatal thyrotoxicosis	POST	Other - Matcho et al	X							
436171	Syphilis in mother complicating pregnancy, childbirth AND/OR puerperium	POST	Other - Matcho et al	X							
197930	Renal hypertension complicating pregnancy, childbirth and the puerperium	POST	Other - Matcho et al	X							
432371	Metabolic disorder following molar AND/OR ectopic pregnancy	POST	Other - Matcho et al	X							
133024	Tetanus neonatorum	POST	Other - Matcho et al	X							
252442	Neonatal aspiration of blood	POST	Other - Matcho et al	X							
434167	Newborn cold injury syndrome	POST	Other - Matcho et al	X							
433869	Congenital rubella syndrome	POST	Other - Matcho et al	X	X						
313833	Puerperal cerebrovascular disorder with postnatal complication	POST	Other - Matcho et al	X							
4143292	Varicose veins of legs in the puerperium	POST	Other - Matcho et al	X	X						
440166	Cervical incompetence with postnatal complication	POST	Other - Matcho et al	X							
433589	Neonatal aspiration of amniotic fluid	POST	Other - Matcho et al	X							
443242	Varicose veins of perineum and vulva in pregnancy and the puerperium	POST	Other - Matcho et al	X							
442083	Obstetric laceration of cervix with postnatal problem	POST	Other - Matcho et al	X							
437681	Kernicterus due to isoimmunization	POST	Other - Matcho et al	X							
442079	Obstetric damage to pelvic joints and ligaments with postnatal problem	POST	Other - Matcho et al	X							
442300	Thyroid disease in mother complicating pregnancy, childbirth AND/OR puerperium	POST	Other - Matcho et al	X	X						
443054	Maternal gonorrhoea during pregnancy, childbirth and the puerperium	POST	Other - Matcho et al	X	X						
444244	Infection of nipple, associated with childbirth	POST	Other - Matcho et al	X							
442271	Shock following molar AND/OR ectopic pregnancy	POST	Other - Matcho et al	X							
442294	Obstetrical pulmonary complication of anesthesia AND/OR sedation	POST	Other - Matcho et al	X							
314107	Obstetrical cardiac complication of anesthesia AND/OR sedation	POST	Other - Matcho et al	X							

42742390	Car seat/bed testing for airway integrity, neonate, with continual nursing observation and continuous recording of pulse oximetry, heart rate and respiratory rate, with interpretation and report; 60 minutes	POST	Other - Matcho et al	X	X					
4328870	Puerperal endometritis	POST	Other - Matcho et al	X	X					
80478	Engorgement of breasts associated with childbirth	POST	Other - Matcho et al	X						
440777	Embolism following molar AND/OR ectopic pregnancy	POST	Other - Matcho et al	X						
40766616	Breast feeding PhenX	POST	Other - Matcho et al	X	X					
4015149	Postnatal visit	POST	Other - Matcho et al	X						
40759177	Body weight - Reported --at birth	POST	Other - Matcho et al	X	X					
4187520	Weight - baby	POST	Other - Matcho et al	X						
4061471	Haemorrhoids in pregnancy and the puerperium	POST	Other - Matcho et al	X						
4066260	Galactorrhea in pregnancy and the puerperium	POST	Other - Matcho et al	X						
4152967	Laceration of perineum	POST	Other - Matcho et al	X						
4071485	Foetus or neonate affected by caesarean section	POST	Other - Matcho et al	X	X					
4063159	Subluxation of symphysis pubis in pregnancy, childbirth and the puerperium	POST	Other - Matcho et al	X	X					
4048607	Transient vaginal bleeding in newborn	POST	Other - Matcho et al	X						
4345685	Toxic erythema of newborn	POST	Other - Matcho et al	X	X					
4116804	Observation of head circumference	POST	Other - Matcho et al	X	X					
4345684	Breastfeeding problem in the newborn	POST	Other - Matcho et al	X	X					
4140071	Floppy infant syndrome	POST	Other - Matcho et al	X	X					
4003030	Retained placenta	POST	Other - Matcho et al	X						
4028781	Infection - perineal wound	POST	Other - Matcho et al	X						
4102318	Puerperal septicemia	POST	Other - Matcho et al	X	X					
4079843	Neonatal death	POST	Other - Matcho et al	X						
4340944	RICP	POST	Other - Matcho et al	X	X					
4047718	Fetus or neonate affected by forceps delivery	POST	Other - Matcho et al	X	X					
4221399	Breast-feeding inhibitors causing neonatal jaundice	POST	Other - Matcho et al	X	X					
4034153	Vulval obstetric varicose veins	POST	Other - Matcho et al	X						
4047719	Fetus or neonate affected by vacuum extraction delivery	POST	Other - Matcho et al	X	X					
4063697	Obstetric breast infections	POST	Other - Matcho et al	X						
4096535	Vaginal tear resulting from childbirth	POST	Other - Matcho et al	X						
441644	Varicose veins of legs in pregnancy and the puerperium	POST	Other - Matcho et al	X						
4091790	Labial tear	POST	Other - Matcho et al	X						
4034650	Omphalitis	POST	Other - Matcho et al	X						
4198705	Jittery newborn	POST	Other - Matcho et al	X						
4047852	Cephalhaematoma due to birth trauma	POST	Other - Matcho et al	X	X					
4296470	Perineal laceration involving labia	POST	Other - Matcho et al	X						
4015431	Apgar at 1 minute = 9	POST	Other - Matcho et al	X						
4079860	Umbilical polyp of newborn	POST	Other - Matcho et al	X	X					
4199437	Postpartum thyroiditis	POST	Other - Matcho et al	X	X					
197349	Perineal haematoma	POST	Other - Matcho et al	X						
4049041	Overfeeding in newborn	POST	Other - Matcho et al	X	X					
4066258	Breastfeeding painful	POST	Other - Matcho et al	X						
4173180	Transient neonatal hyperbilirubinaemia	POST	Other - Matcho et al	X	X					
4307303	Early neonatal death	POST	Other - Matcho et al	X						
4004785	Fetal alcohol syndrome	POST	Other - Matcho et al	X	X					
4283942	Bronchopulmonary dysplasia of newborn	POST	Other - Matcho et al	X	X					
4262580	Primary sleep apnea of newborn	POST	Other - Matcho et al	X	X					
4150001	Retained placenta or membranes with no haemorrhage	POST	Other - Matcho et al	X						
4153452	Severe birth asphyxia - apgar score less than 4 at 1 minute	POST	Other - Matcho et al	X						
4071063	Sepsis of the newborn	POST	Other - Matcho et al	X	X					
4016052	Apgar at 1 minute = 8	POST	Other - Matcho et al	X						
439083	Hydatidiform mole, benign	TRO	Other - Matcho et al	X	X					
2110338	Uterine evacuation and curettage for hydatidiform mole	TRO	Other - Matcho et al	X	X					
4214186	Gestational trophoblastic disease	TRO	Other - Matcho et al	X	X					
4152445	Antenatal ultrasound confirms intrauterine pregnancy	ULS	Other - Matcho et al	X						
4060622	US obstetric scan normal	ULS	Other - Matcho et al	X						
4061130	US scan - fetal cephalometry	ULS	Other - Matcho et al	X	X					
4060623	US obstetric scan abnormal	ULS	Other - Matcho et al	X	X					
2212173	Urine pregnancy test, by visual color comparison methods	UP	Other - Matcho et al	X	X					
4055285	Urine pregnancy test	UP	Other - Matcho et al	X	X					
437369	Postmature infancy	POSTT	Post-term	X	X					
440833	Postmaturity of fetus	POSTT	Post-term	X						
441128	Post-term infant, not heavy-for-dates	POSTT	Post-term	X						
440525	Prematurity of fetus	PREM	Preterm pregnancy	X	X					
440519	Premature - weight 1000g-2499g or gestation of 28-37weeks	PREM	Preterm pregnancy	X						
435925	Very premature - less than 1000g or less than 28 weeks	PREM	Preterm pregnancy	X						
136530	Sclerema neonatorum	PREM	Preterm pregnancy	X	X					
4064709	L281400 Premature rupture of membranes, labour delayed by therapy		Preterm pregnancy	X						
4150406	635A.00 Baby premature 37 weeks		Preterm pregnancy	X						
441632	Complete miscarriage with embolism		Spontaneous abortion (SA)	X				X		
441910	Complete miscarriage with damage to pelvic organs or tissues	SA	Spontaneous abortion (SA)	X				X		
441921	Complete inevitable miscarriage complicated by genital tract and pelvic infection		Spontaneous abortion (SA)	X				X		
444365	Abortion	SA	Spontaneous abortion (SA)	X						
2004526	Aspiration curettage following delivery or abortion	SA	Spontaneous abortion (SA)	X	X					
2101811	Anesthesia for incomplete or missed abortion procedures	SA	Spontaneous abortion (SA)	X	X	X	X	X		
2110292	Hysterotomy, abdominal (eg, for hydatidiform mole, abortion)	SA	Spontaneous abortion (SA)	X						
2110325	Treatment of incomplete abortion, any trimester, completed surgically	SA	Spontaneous abortion (SA)	X	X	X	X	X		
443213	SB - Stillbirth	SB	Stillbirth (SB)	X	X					
4079844	Foetal death	SB	Stillbirth (SB)	X	X					
4129846	Antepartum foetal death	SB	Stillbirth (SB)	X						
4062636	Bacterial disease screening	Not categorized	Other - Matcho et al	X	X					
4061811	Rubella screening	Not categorized	Other - Matcho et al	X						
2004490	Dilation and curettage following delivery or abortion	Not categorized	Other - Matcho et al	X	X					
2110306	Hysterorrhaphy of ruptured uterus	Not categorized	Other - Matcho et al	X						
4327048	Embryo transfer	Not categorized	Other - Matcho et al	X						
3039211	Alpha-1-fetoprotein.tumor marker [Moles/volume] in Serum or Plasma	Not categorized	Other - Matcho et al	X						
81400	Neonatal myasthenia gravis	Not categorized	Other - Matcho et al	X						

2110290	Fetal fluid drainage (eg, vesicocentesis, thoracocentesis, paracentesis), including ultrasound guidance	Not categorized	Other - Matcho et al	X						
4164218	Rubella in pregnancy	Not categorized	Other - Matcho et al	X						
442171	Vasa previa	Not categorized	Other - Matcho et al	X	X					
444269	Shock during AND/OR following labor AND/OR delivery	Not categorized	Other - Matcho et al	X	X					
435886	Maternal distress	Not categorized	Other - Matcho et al	X						
198260	Perinatal adrenal haemorrhage	Not categorized	Other - Matcho et al	X						
374430	Obstetrical central nervous system complication of anesthesia AND/OR sedation	Not categorized	Other - Matcho et al	X						
316494	Cerebrovascular disorder in the puerperium	Not categorized	Other - Matcho et al	X						
2108121	Exchange transfusion, blood; newborn	Not categorized	Other - Matcho et al	X						
438562	Fetus or neonate affected by placental or breast transfer of anticonvulsant	Not categorized	Other - Matcho et al	X						
441640	Maternal gonorrhoea during pregnancy - baby delivered	Not categorized	Other - Matcho et al	X						
4235812	Septic thrombophlebitis	Not categorized	Other - Matcho et al	X						
2004810	Fetal blood sampling and biopsy	Not categorized	Other - Matcho et al	X						
3004209	Glucose [Mass/volume] in Serum or Plasma --1 hour post 50 g lactose PO	Not categorized	Other - Matcho et al	X						
442085	Rupture of uterus before labor with antenatal problem	Not categorized	Other - Matcho et al	X						
437946	Short cord with antenatal problem	Not categorized	Other - Matcho et al	X						
436820	Late anemia due to isoimmunization	Not categorized	Other - Matcho et al	X						
441365	Retracted nipple in pregnancy, the puerperium or lactation with antenatal complication	Not categorized	Other - Matcho et al	X						
195328	Rupture of uterus before onset of labor	Not categorized	Other - Matcho et al	X	X					
3036671	Fasting glucose [Mass/volume] in Capillary blood	Not categorized	Other - Matcho et al	X	X					
436749	Gonorrhoea in mother complicating pregnancy, childbirth AND/OR puerperium	Not categorized	Other - Matcho et al	X						
3031469	Cytomegalovirus DNA [Presence] in Amniotic fluid by Probe and target amplification method	Not categorized	Other - Matcho et al	X						
442255	Intestinal obstruction by inspissated milk in newborn	Not categorized	Other - Matcho et al	X						
3018663	Hemoglobin F/Hemoglobin.total in Amniotic fluid	Not categorized	Other - Matcho et al	X	X					
192980	Placental polyp	Not categorized	Other - Matcho et al	X						
3005478	Glucose [Mass/time] in 24 hour Urine	Not categorized	Other - Matcho et al	X	X					
3025335	Insulin [Units/volume] in Serum or Plasma --30 minutes post 75 g glucose PO	Not categorized	Other - Matcho et al	X						
436218	Fetus OR newborn affected by hallucinogenic agent transmitted via placenta AND/OR breast milk	Not categorized	Other - Matcho et al	X						
443245	Vulval and/or perineal haematoma during delivery	Not categorized	Other - Matcho et al	X						
438226	Short cord	Not categorized	Other - Matcho et al	X						
3007034	Glucose [Mass/volume] in 24 hour Urine	Not categorized	Other - Matcho et al	X	X					
2721174	FETOSCOPIC LASER THERAPY FOR TREATMENT OF TWIN-TO-TWIN TRANSFUSION SYNDROME	Not categorized	Other - Matcho et al	X						
2004825	Fetal pulse oximetry	Not categorized	Other - Matcho et al	X	X					
200455	Failed attempted abortion complicated by delayed AND/OR excessive hemorrhage	Not categorized	Other - Matcho et al	X	X					
74422	Outlet contraction of pelvis	Not categorized	Other - Matcho et al	X						
4064579	Obstetric inversion of uterus with postnatal problem	Not categorized	Other - Matcho et al	X						
193259	Failed attempted abortion complicated by damage to pelvic organs AND/OR tissues	Not categorized	Other - Matcho et al	X						
434416	Tuberculosis in mother complicating pregnancy, childbirth AND/OR puerperium	Not categorized	Other - Matcho et al	X						
74706	Failed attempted abortion complicated by genital-pelvic infection	Not categorized	Other - Matcho et al	X						
3024209	Toxoplasma gondii DNA [Presence] in Amniotic fluid by Probe and target amplification method	Not categorized	Other - Matcho et al	X						
2110341	Unlisted laparoscopy procedure, maternity care and delivery	Not categorized	Other - Matcho et al	X						
3013219	Glucose [Mass/volume] in Serum or Plasma --5th specimen post XXX challenge	Not categorized	Other - Matcho et al	X						
3037292	Glucose-6-Phosphate dehydrogenase [Presence] in Red Blood Cells	Not categorized	Other - Matcho et al	X	X					
441127	Fetus OR newborn affected by medicinal agents transmitted via placenta AND/OR breast milk	Not categorized	Other - Matcho et al	X	X					
4062567	Asymptomatic bacteriuria in pregnancy with postnatal complication	Not categorized	Other - Matcho et al	X						
3011161	Glucose [Mass/volume] in Serum or Plasma --4 hours post dose glucose	Not categorized	Other - Matcho et al	X						
196765	Uterine inversion	Not categorized	Other - Matcho et al	X	X					
442914	Drug dependence in the puerperium - baby delivered	Not categorized	Other - Matcho et al	X						
437062	Cracked nipple in pregnancy, the puerperium or lactation with antenatal complication	Not categorized	Other - Matcho et al	X						
4063692	Placental polyp with postnatal complication	Not categorized	Other - Matcho et al	X						
3027590	Insulin [Units/volume] in Serum or Plasma --3 hours post 75 g glucose PO	Not categorized	Other - Matcho et al	X						
3042443	Glucose [Mass/volume] in Serum or Plasma --pre XXX challenge	Not categorized	Other - Matcho et al	X	X					
3019561	Hemoglobin F [Presence] in Amniotic fluid	Not categorized	Other - Matcho et al	X						
78217	Inlet contraction of pelvis	Not categorized	Other - Matcho et al	X						
2110289	Fetal umbilical cord occlusion, including ultrasound guidance	Not categorized	Other - Matcho et al	X						
3026522	Creatinine [Mass/volume] in Amniotic fluid	Not categorized	Other - Matcho et al	X						
3009414	Glucose [Mass/volume] in Serum or Plasma --5 hours post dose glucose	Not categorized	Other - Matcho et al	X						
3001978	Glucose [Mass/volume] in Synovial fluid	Not categorized	Other - Matcho et al	X						
3023306	Glucose [Mass/volume] in Serum or Plasma --1.5 hours post dose glucose	Not categorized	Other - Matcho et al	X	X					
2101815	Cesarean hysterectomy following neuraxial labor analgesia/anesthesia (List separately in addition to code for primary procedure)	Not categorized	Other - Matcho et al	X						
3000940	Glucose [Mass/volume] in Serum or Plasma --4 hours post 75 g glucose PO	Not categorized	Other - Matcho et al	X	X					
40766808	Cells analyzed [#] in Amniotic fluid by Molecular genetics method	Not categorized	Other - Matcho et al	X						
40766811	Cells counted [#] in Amniotic fluid by Molecular genetics method	Not categorized	Other - Matcho et al	X						
40766809	ISCN band level [#] in Amniotic fluid Qualitative by Molecular genetics method	Not categorized	Other - Matcho et al	X						
40766810	Karyotype [Identifier] in Amniotic fluid Narrative	Not categorized	Other - Matcho et al	X						
40766812	Colonies counted [#] in Amniotic fluid by Molecular genetics method	Not categorized	Other - Matcho et al	X	X					
40766807	Cells karyotyped.total [#] in Amniotic fluid	Not categorized	Other - Matcho et al	X						

3025070	Glucose [Mass/volume] in Serum or Plasma --2 hours post 50 g lactose PO	Not categorized	Other - Matcho et al	X						
442096	Prolapsed arm presentation	Not categorized	Other - Matcho et al	X						
442051	Obstetric air pulmonary embolism with antenatal complication	Not categorized	Other - Matcho et al	X						
3050726	Alpha-1-Fetoprotein panel in Serum or Plasma	Not categorized	Other - Matcho et al	X						
3027936	Glucose [Mass/volume] in Serum or Plasma --30 minutes post 50 g lactose PO	Not categorized	Other - Matcho et al	X						
77059	Failure of lactation with antenatal complication	Not categorized	Other - Matcho et al	X						
435324	Maternal malaria during pregnancy - baby not yet delivered	Not categorized	Other - Matcho et al	X						
442916	Congenital cardiovascular disorder in the puerperium - baby delivered during current episode of care	Not categorized	Other - Matcho et al	X						
2004767	Operations on fetus to facilitate delivery	Not categorized	Other - Matcho et al	X						
3023511	Choriogonadotropin [Units/volume] in Urine	Not categorized	Other - Matcho et al	X	X					
4087113	Slow fetal growth AND/OR fetal malnutrition	Not categorized	Other - Matcho et al	X						
2110291	Fetal shunt placement, including ultrasound guidance	Not categorized	Other - Matcho et al	X						
442353	Failed attempted abortion complicated by metabolic disorder	Not categorized	Other - Matcho et al	X						
3000483	Glucose [Mass/volume] in Blood	Not categorized	Other - Matcho et al	X	X					
2004771	Incision of cervix to assist delivery	Not categorized	Other - Matcho et al	X						
40758321	Chromosome analysis.interphase [interpretation] in Amniotic fluid by Fluorescent in situ hybridization (FISH) Narrative	Not categorized	Other - Matcho et al	X						
40766801	Cells karyotyped.total [#] in Chorionic villus sample	Not categorized	Other - Matcho et al	X						
3016941	Insulin [Mass/volume] in Serum or Plasma --6 hours post 75 g glucose PO	Not categorized	Other - Matcho et al	X						
40766803	ISCN band level [#] in Chorionic villus sample Qualitative by Molecular genetics method	Not categorized	Other - Matcho et al	X						
3024047	Glucose [Mass/volume] in Serum or Plasma --3 hours post 50 g lactose PO	Not categorized	Other - Matcho et al	X						
40766804	Karyotype [Identifier] in Chorionic villus sample Narrative	Not categorized	Other - Matcho et al	X						
40766802	Cells analyzed [#] in Chorionic villus sample by Molecular genetics method	Not categorized	Other - Matcho et al	X						
40766805	Cells counted [#] in Chorionic villus sample by Molecular genetics method	Not categorized	Other - Matcho et al	X						
197047	Failed attempted abortion complicated by renal failure	Not categorized	Other - Matcho et al	X						
439380	Obstetric pyaemic and septic pulmonary embolism with antenatal complication	Not categorized	Other - Matcho et al	X						
3014625	Acetylcholinesterase [Presence] in Amniotic fluid	Not categorized	Other - Matcho et al	X	X					
3038624	Choriogonadotropin.tumor marker [Units/volume] in Serum or Plasma	Not categorized	Other - Matcho et al	X	X					
3017786	Bilirubin.total [Mass/volume] in Amniotic fluid by Spectrophotometry direct	Not categorized	Other - Matcho et al	X						
3012805	Glucose [Mass/volume] in Serum or Plasma --30 minutes post 100 g glucose PO	Not categorized	Other - Matcho et al	X						
3015916	Alpha-1-Fetoprotein [Units/volume] in Serum or Plasma	Not categorized	Other - Matcho et al	X	X					
3046146	Glucose phosphate isomerase [Enzymatic activity/mass] in Red Blood Cells	Not categorized	Other - Matcho et al	X	X					
3000788	Lamellar bodies [#/volume] in Amniotic fluid	Not categorized	Other - Matcho et al	X						
3030260	Glucose [Presence] in Urine by Automated test strip	Not categorized	Other - Matcho et al	X	X					
2721171	REPAIR, MYELOMENINGOCELE IN THE FETUS, PROCEDURE PERFORMED IN UTERO	Not categorized	Other - Matcho et al	X						
443868	Failed attempted abortion complicated by embolism	Not categorized	Other - Matcho et al	X						
3028428	Insulin [Units/volume] in Serum or Plasma --4 hours post 75 g glucose PO	Not categorized	Other - Matcho et al	X						
443019	Maternal syphilis in the puerperium - baby delivered during current episode of care	Not categorized	Other - Matcho et al	X						
3005834	Glucose [Moles/volume] in Serum or Plasma --2 hours post XXX challenge	Not categorized	Other - Matcho et al	X						
3002217	Glucose phosphate isomerase [Enzymatic activity/volume] in Red Blood Cells	Not categorized	Other - Matcho et al	X						
3035502	Maternal cell contamination [Identifier] in Amniotic fluid Nominal	Not categorized	Other - Matcho et al	X	X					
3022268	Glucose [Mass/volume] in Serum or Plasma --4 hours post 100 g glucose PO	Not categorized	Other - Matcho et al	X						
3010794	Glucose [Mass/volume] in Serum or Plasma --5 hours post 100 g glucose PO	Not categorized	Other - Matcho et al	X						
201637	Failed attempted abortion complicated by shock	Not categorized	Other - Matcho et al	X						
3000404	Glucose [Mass/volume] in Serum or Plasma --1.5 hours post 50 g lactose PO	Not categorized	Other - Matcho et al	X						
2213206	Necropsy (autopsy), gross and microscopic; stillborn or newborn with brain	Not categorized	Other - Matcho et al	X						
3034165	Alpha-1-Fetoprotein [Mass/volume] in Body fluid	Not categorized	Other - Matcho et al	X						
3020317	Glucose [Mass/volume] in Serum or Plasma --6th specimen post XXX challenge	Not categorized	Other - Matcho et al	X						
3030753	Glucose tolerance 3 hours gestational panel in Serum or Plasma	Not categorized	Other - Matcho et al	X	X					
4344631	Bottle feeding problem in the newborn	Not categorized	Other - Matcho et al	X						
4225278	Facial palsy as birth trauma	Not categorized	Other - Matcho et al	X						
4064977	Retained portion of placenta or membranes with no haemorrhage	Not categorized	Other - Matcho et al	X	X					
4309151	Complications of attempted introduction of embryo in embryo transfer	Not categorized	Other - Matcho et al	X						
4187920	Perineal laceration involving fourchette	Not categorized	Other - Matcho et al	X						
4149999	Fetal distress-affecting care	Not categorized	Other - Matcho et al	X						
43530979	Miscarriage of tubal ectopic pregnancy	Not categorized	Other - Matcho et al	X						
4129180	Laceration of vulva	Not categorized	Other - Matcho et al	X						
4129186	Obstetric disorders of breast and lactation	Not categorized	Other - Matcho et al	X	X					
4145947	Subnormal birth weight	Not categorized	Other - Matcho et al	X	X					
4212326	Neonatal Abstinence Syndrome	Not categorized	Other - Matcho et al	X	X					
4297249	Obstetric operation	Not categorized	Other - Matcho et al	X						
4150406	Baby premature 37 weeks	Not categorized	Other - Matcho et al	X						
4165077	Total hysterectomy after caesarean delivery	Not categorized	Other - Matcho et al	X						
4141511	Fetal biometry using ultrasound	Not categorized	Other - Matcho et al	X	X					
4087102	Spontaneous abortion at 8 to 28 weeks	Not categorized	Other - Matcho et al	X						
4048144	Foetal distress in labour - liveborn	Not categorized	Other - Matcho et al	X						
4060242	A/N care: H/O perinatal death	Not categorized	Other - Matcho et al	X						
4060547	Cephalopelvic disproportion	Not categorized	Other - Matcho et al	X						
4014581	Maternal drug abuse	Not categorized	Other - Matcho et al	X						
4049044	Congenital hypertonia	Not categorized	Other - Matcho et al	X	X					
4014314	Bonding problems	Not categorized	Other - Matcho et al	X						
4302541	Obstetric procedure	Not categorized	Other - Matcho et al	X	X					

4015292	Apgar at 1 minute = 7	Not categorized	Other - Matcho et al	X						
4071202	Neonatal hypertension	Not categorized	Other - Matcho et al	X	X					
4170459	Vaginal muscle tear	Not categorized	Other - Matcho et al	X						
4218938	Transverse lie	Not categorized	Other - Matcho et al	X	X					
4123182	Removal of cerclage material from cervix	Not categorized	Other - Matcho et al	X						
4143745	Neonatal conjunctivitis	Not categorized	Other - Matcho et al	X						
4016061	High risk infant	Not categorized	Other - Matcho et al	X						
4070527	Brachial plexus palsy due to birth trauma	Not categorized	Other - Matcho et al	X						
4150536	Pregnancy prolonged - 41 weeks	Not categorized	Other - Matcho et al	X						
4062112	Retained products with no hemorrhage with postnatal problem	Not categorized	Other - Matcho et al	X						
4063042	Pre-existing diabetes mellitus, insulin-dependent	Not categorized	Other - Matcho et al	X	X					
4034088	Incomplete hydatidiform mole	Not categorized	Other - Matcho et al	X	X					
4143203	Fetus with chromosomal abnormality	Not categorized	Other - Matcho et al	X						
4014718	Placental abnormality	Not categorized	Other - Matcho et al	X	X					
4060249	No antenatal care	Not categorized	Other - Matcho et al	X						
4220985	Caput succedaneum	Not categorized	Other - Matcho et al	X	X					
4062124	Obstetric spinal and epidural anesthesia-induced headache	Not categorized	Other - Matcho et al	X						
4129689	Vulval abnormality in pregnancy, childbirth and the puerperium	Not categorized	Other - Matcho et al	X						
4129710	CHM - Complete hydatidiform mole	Not categorized	Other - Matcho et al	X	X					
4015425	Baby BW = < 3% (under 2500g)	Not categorized	Other - Matcho et al	X						
4244873	Congenital viral hepatitis B infection	Not categorized	Other - Matcho et al	X						
43530882	Suspected fetal anencephaly	Not categorized	Other - Matcho et al	X						
4070024	Biophysical profile of fetus	Not categorized	Other - Matcho et al	X	X					
4064287	Large-for-dates fetus	Not categorized	Other - Matcho et al	X	X					
4034152	Varicose veins - obstetric	Not categorized	Other - Matcho et al	X						
4048751	Neonatal skin infection	Not categorized	Other - Matcho et al	X	X					
4302027	Neonatal dehydration	Not categorized	Other - Matcho et al	X	X					
4024302	[M]Teratoma, malignant, NOS	Not categorized	Other - Matcho et al	X						
4145940	Tumour of uterine body in pregnancy, childbirth and the puerperium	Not categorized	Other - Matcho et al	X	X					
4251491	Congenital toxoplasmosis	Not categorized	Other - Matcho et al	X	X					
4071869	Congenital cardiac failure	Not categorized	Other - Matcho et al	X						
443415	Gestation greater than 24 weeks	Not categorized	Other - Matcho et al	X						
4030259	Puerperal uterine subinvolution	Not categorized	Other - Matcho et al	X	X					
73534	Disproportion between fetus and pelvis	Not categorized	Other - Matcho et al	X						
4153574	Neonatal diarrhoea	Not categorized	Other - Matcho et al	X						
4060245	A/N care: poor A/N attender	Not categorized	Other - Matcho et al	X						
4145946	Fetus with central nervous system malformation	Not categorized	Other - Matcho et al	X						
4129553	Suspect fetal spina bifida	Not categorized	Other - Matcho et al	X						
4014306	Apgar at 1 minute = 6	Not categorized	Other - Matcho et al	X						
4129703	Uterine fibroid affecting obstetric care	Not categorized	Other - Matcho et al	X						
4319897	Repair of current obstetric laceration of uterus	Not categorized	Other - Matcho et al	X						
4048755	Neonatal withdrawal symptoms from maternal use of drugs of addiction	Not categorized	Other - Matcho et al	X	X					
4171358	Slow feeding in newborn	Not categorized	Other - Matcho et al	X	X					
4070410	Foetus or neonate affected by placenta praevia	Not categorized	Other - Matcho et al	X	X					
4024541	PND - Perinatal death	Not categorized	Other - Matcho et al	X						
4344624	ECV - External cephalic version	Not categorized	Other - Matcho et al	X						
4164128	Dysfunctional labor	Not categorized	Other - Matcho et al	X	X					
4210143	Anhydramnios	Not categorized	Other - Matcho et al	X						
4014469	Apgar at 1 minute = 5	Not categorized	Other - Matcho et al	X						
4345688	Intracerebral haemorrhage in foetus or newborn	Not categorized	Other - Matcho et al	X	X					
4014473	Apgar at 10 minutes = 0	Not categorized	Other - Matcho et al	X						
4064965	Obstetric trauma causing pelvic hematoma	Not categorized	Other - Matcho et al	X						
4070424	Fetus or neonate affected by precipitate delivery	Not categorized	Other - Matcho et al	X	X					
4338674	Obstetric galactocele	Not categorized	Other - Matcho et al	X						
4274334	Oblique lie	Not categorized	Other - Matcho et al	X						
4015303	Fetal movements seen	Not categorized	Other - Matcho et al	X						
4213449	Brow presentation	Not categorized	Other - Matcho et al	X						
4061784	Pregnant - on abdom. palpation	Not categorized	Other - Matcho et al	X						
4150930	Neonatal hypocalcaemia	Not categorized	Other - Matcho et al	X	X					
4071867	Neonatal cerebral leucomalacia	Not categorized	Other - Matcho et al	X	X					
4062671	Pregnancy-induced edema and proteinuria without hypertension	Not categorized	Other - Matcho et al	X						
4016471	Apgar at 10 minutes = 10	Not categorized	Other - Matcho et al	X						
442084	Ruptured uterus before labour	Not categorized	Other - Matcho et al	X						
4057979	Pre-existing secondary hypertension complicating pregnancy, childbirth and puerperium	Not categorized	Other - Matcho et al	X						
4129034	Vaginal varices in pregnancy	Not categorized	Other - Matcho et al	X						
4048747	Meconium plug	Not categorized	Other - Matcho et al	X	X					
4109462	Forceps delivery failed	Not categorized	Other - Matcho et al	X	X					
4065627	Spinal and epidural anaesthesia-induced headache during labour and delivery	Not categorized	Other - Matcho et al	X	X					
4102760	Low maternal weight gain	Not categorized	Other - Matcho et al	X	X					
4245799	Atony of uterus	Not categorized	Other - Matcho et al	X						
4028633	Rectocele - baby delivered	Not categorized	Other - Matcho et al	X						
4233425	Acquired periventricular cysts of newborn	Not categorized	Other - Matcho et al	X	X					
4239065	Congenital herpes simplex infection	Not categorized	Other - Matcho et al	X	X					
4171359	Failed attempted abortion	Not categorized	Other - Matcho et al	X						
443291	Maternal syphilis during pregnancy, childbirth and the puerperium	Not categorized	Other - Matcho et al	X	X					
4220675	Congenital infection	Not categorized	Other - Matcho et al	X						
4049032	Idiopathic hydrops fetalis	Not categorized	Other - Matcho et al	X						
4223016	BRA - Bilateral renal agenesis	Not categorized	Other - Matcho et al	X						
4129701	Rectocele affecting obstetric care	Not categorized	Other - Matcho et al	X						
4174308	Congenital viral pneumonia	Not categorized	Other - Matcho et al	X						
4071591	Vacuum extraction chignon	Not categorized	Other - Matcho et al	X						
4173178	Aspiration of mucus in newborn	Not categorized	Other - Matcho et al	X						
4118054	Fetus or neonate affected by maternal complication of pregnancy	Not categorized	Other - Matcho et al	X	X					
4005289	Single umbilical artery	Not categorized	Other - Matcho et al	X	X					
4129042	VBAC - Vaginal birth after caesarean section	Not categorized	Other - Matcho et al	X	X					
4194229	Congenital hepatic fibrosis	Not categorized	Other - Matcho et al	X						
4171691	Perinatal pneumothorax	Not categorized	Other - Matcho et al	X	X					
4062551	Decreased lactation	Not categorized	Other - Matcho et al	X	X					
4153450	Fracture of radius and/or ulna due to birth trauma	Not categorized	Other - Matcho et al	X						
4305304	Fetal distress	Not categorized	Other - Matcho et al	X						

4147341	Prolonged spontaneous rupture of membranes	Not categorized	Other - Matcho et al	X						
4071069	Perinatal epistaxis	Not categorized	Other - Matcho et al	X						
4014305	Apgar at 1 minute = 3	Not categorized	Other - Matcho et al	X						
4014470	Apgar at 1 minute = 10	Not categorized	Other - Matcho et al	X						
443250	Drug dependence during pregnancy, childbirth and the puerperium	Not categorized	Other - Matcho et al	X	X					
4129015	Uterine fibroid complicating antenatal care, baby not yet delivered	Not categorized	Other - Matcho et al	X						
4063458	Alopecia of pregnancy	Not categorized	Other - Matcho et al	X						
317100	Fetal tachycardia	Not categorized	Other - Matcho et al	X						
4006961	Chloasma of pregnancy	Not categorized	Other - Matcho et al	X						
438258	Baby birth weight above 2.5kg	Not categorized	Other - Matcho et al	X						
4134722	Disruption of episiotomy wound in the puerperium	Not categorized	Other - Matcho et al	X						
4149398	Congenital renal failure	Not categorized	Other - Matcho et al	X	X					
4061458	Delivery by caesarean hysterectomy	Not categorized	Other - Matcho et al	X						
4071365	Fetus or neonate affected by maternal injury	Not categorized	Other - Matcho et al	X						
4034151	Hymen tear	Not categorized	Other - Matcho et al	X						
4146460	Thrombophlebitis of legs in pregnancy	Not categorized	Other - Matcho et al	X	X					
4061532	A/N U/S scan for slow growth	Not categorized	Other - Matcho et al	X	X					
4236182	Interstitial pulmonary fibrosis of prematurity	Not categorized	Other - Matcho et al	X						
4188630	Mendelson's syndrome resulting from a procedure	Not categorized	Other - Matcho et al	X						
4113510	Failed medical abortion, without complication	Not categorized	Other - Matcho et al	X						
4113846	Delivery of viable fetus in abdominal pregnancy	Not categorized	Other - Matcho et al	X						
4063037	Viral hepatitis complicating pregnancy, childbirth and the puerperium	Not categorized	Other - Matcho et al	X	X					
4080880	Congenital falciparum malaria	Not categorized	Other - Matcho et al	X						
4034079	Uterine fibroid - baby delivered	Not categorized	Other - Matcho et al	X						
4147395	Vaginal varices in the puerperium	Not categorized	Other - Matcho et al	X						
442081	Pelvic soft tissue abnormality in pregnancy, childbirth and the puerperium	Not categorized	Other - Matcho et al	X	X					
4071866	Withdrawal symptoms from therapeutic use of drugs in newborn	Not categorized	Other - Matcho et al	X	X					
4086931	Alpha-fetoprotein blood test status	Not categorized	Other - Matcho et al	X						
4121646	Genital varices in pregnancy	Not categorized	Other - Matcho et al	X	X					
4048603	Neonatal vaginal hemorrhage	Not categorized	Other - Matcho et al	X	X					
4345345	Neonatal alloimmune thrombocytopenia	Not categorized	Other - Matcho et al	X						
4048594	Sepsis of newborn due to Staphylococcus aureus	Not categorized	Other - Matcho et al	X	X					
4270073	Perinatal pneumomediastinum	Not categorized	Other - Matcho et al	X	X					
4071480	Fetus or neonate affected by placental transfusion syndrome	Not categorized	Other - Matcho et al	X	X					
4170448	Intrauterine hypoxia	Not categorized	Other - Matcho et al	X						
4221837	Klumpke-Dejerine brachial plexus injury	Not categorized	Other - Matcho et al	X						
4015430	Apgar at 1 minute = 1	Not categorized	Other - Matcho et al	X						
442417	Obstetric perineal wound disruption with postnatal complication	Not categorized	Other - Matcho et al	X						
4047855	Fracture of tibia and/or fibula due to birth trauma	Not categorized	Other - Matcho et al	X						
4172864	Cardiac failure developing in the perinatal period	Not categorized	Other - Matcho et al	X	X					
4006949	Harlequin ichthyosis	Not categorized	Other - Matcho et al	X						
4014437	Fetal size does not accord with dates	Not categorized	Other - Matcho et al	X	X					
444292	Newborn cerebral depression	Not categorized	Other - Matcho et al	X	X					
4060312	Infections of urethra in pregnancy	Not categorized	Other - Matcho et al	X	X					
4172867	Neonatal hypotension	Not categorized	Other - Matcho et al	X						
4071727	Sepsis of newborn due to Escherichia coli	Not categorized	Other - Matcho et al	X	X					
4059983	Pregnant - V.E. confirms	Not categorized	Other - Matcho et al	X						
4034096	Intrapartum eclampsia	Not categorized	Other - Matcho et al	X	X					
4014468	Apgar at 1 minute = 0	Not categorized	Other - Matcho et al	X						
4173577	Kernicterus of newborn	Not categorized	Other - Matcho et al	X						
4062906	Transient hypertension of pregnancy with postnatal complication	Not categorized	Other - Matcho et al	X						
4071070	Neonatal hematemesis	Not categorized	Other - Matcho et al	X	X					
4071073	Late anaemia of newborn due to isoimmunisation	Not categorized	Other - Matcho et al	X						
4048926	Transitory neonatal hyponatraemia	Not categorized	Other - Matcho et al	X	X					
314749	Fetal bradycardia	Not categorized	Other - Matcho et al	X						
4048457	Aspiration of vomit in newborn	Not categorized	Other - Matcho et al	X						
4048923	Perinatal endocrine and metabolic disorders	Not categorized	Other - Matcho et al	X	X					
4170972	Perinatal cardiovascular disorders	Not categorized	Other - Matcho et al	X	X					
4296735	Chloramphenicol toxicity in newborn	Not categorized	Other - Matcho et al	X						
4047853	Scalp bruising due to birth trauma	Not categorized	Other - Matcho et al	X	X					
4071732	Intracranial nontraumatic haemorrhage of foetus and newborn	Not categorized	Other - Matcho et al	X	X					
4113187	Failed medical abortion, complicated by genital tract and pelvic infection	Not categorized	Other - Matcho et al	X						
4170747	Abnormal amniotic fluid	Not categorized	Other - Matcho et al	X						
4090723	Meconium stained amniotic fluid	Not categorized	Other - Matcho et al	X						
80467	Hydrocephalic disproportion	Not categorized	Other - Matcho et al	X						
4317960	Neonatal respiratory failure	Not categorized	Other - Matcho et al	X	X					
4131053	Pelvic hematoma	Not categorized	Other - Matcho et al	X	X					
4251487	Perinatal jaundice due to inspissated bile syndrome	Not categorized	Other - Matcho et al	X						
4048278	Intraventricular (nontraumatic) haemorrhage, grade 2, of foetus and newborn	Not categorized	Other - Matcho et al	X	X					
4071863	Underfeeding in newborn	Not categorized	Other - Matcho et al	X	X					
4153443	Fetus or neonate affected by abnormal uterine contractions	Not categorized	Other - Matcho et al	X	X					
76487	DTA - Deep transverse arrest	Not categorized	Other - Matcho et al	X						
4071611	Congenital group B hemolytic streptococcal pneumonia	Not categorized	Other - Matcho et al	X						
4060691	Premature rupture of membranes with onset of labour after 24 hours of the rupture	Not categorized	Other - Matcho et al	X						
4148451	Environmentally-induced pyrexia in newborn	Not categorized	Other - Matcho et al	X						
4152170	Fetus or neonate affected by maternal polyhydramnios	Not categorized	Other - Matcho et al	X	X					
4049043	Neonatal cerebral ischemia	Not categorized	Other - Matcho et al	X	X					
43530884	Suspected fetal damage from maternal alcohol	Not categorized	Other - Matcho et al	X						
4173170	Neonatal dysrhythmia	Not categorized	Other - Matcho et al	X						
4047992	Foetus or neonate affected by breech presentation before labour	Not categorized	Other - Matcho et al	X	X					
4048150	Neonatal aspiration of milk and regurgitated food	Not categorized	Other - Matcho et al	X	X					
4071484	Foetus or neonate affected by cephalopelvic disproportion during labour and delivery	Not categorized	Other - Matcho et al	X						
444293	Contracted pelvis	Not categorized	Other - Matcho et al	X	X					
4118058	Fetus or neonate affected by ectopic pregnancy	Not categorized	Other - Matcho et al	X						
4147717	Baby birth weight = 1.5-2.0kg	Not categorized	Other - Matcho et al	X	X					
4064286	Small for gestational age baby	Not categorized	Other - Matcho et al	X	X					

4064709	Premature rupture of membranes, labor delayed by therapy	Not categorized	Other - Matcho et al	X						
4150397	Baby birth weight = 1.0-1.5kg	Not categorized	Other - Matcho et al	X	X					
4048592	Congenital viral hepatitis	Not categorized	Other - Matcho et al	X						
4142511	Diagnostic percutaneous examination of fetus	Not categorized	Other - Matcho et al	X	X					
4034097	Symptomatic disorders in pregnancy	Not categorized	Other - Matcho et al	X	X					
4145534	Obstetric umbilical artery Doppler	Not categorized	Other - Matcho et al	X	X					
4048010	Foetus or neonate affected by transverse lie during labour and delivery	Not categorized	Other - Matcho et al	X						
4034091	Antepartum hemorrhage with hypofibrinogenemia	Not categorized	Other - Matcho et al	X						
4113508	Failed medical abortion, complicated by delayed or excessive haemorrhage	Not categorized	Other - Matcho et al	X						
4048460	Tracheobronchial hemorrhage originating in the perinatal period	Not categorized	Other - Matcho et al	X						
4066234	Cerebral venous thrombosis in the puerperium	Not categorized	Other - Matcho et al	X						
4172865	Transient myocardial ischaemia of newborn	Not categorized	Other - Matcho et al	X						
4047854	Fracture of humerus due to birth trauma	Not categorized	Other - Matcho et al	X						
4071721	Neonatal acrocyanosis	Not categorized	Other - Matcho et al	X						
4059900	Labour and delivery complicated by foetal heart rate anomaly with meconium in amniotic fluid	Not categorized	Other - Matcho et al	X						
4337102	Neonatal hypomagnesaemia	Not categorized	Other - Matcho et al	X	X					
4048277	Intraventricular (nontraumatic) haemorrhage, grade 1, of foetus and newborn	Not categorized	Other - Matcho et al	X	X					
4265609	Embryonal cyst of vagina	Not categorized	Other - Matcho et al	X						
4064978	Complications of anaesthesia during labour and delivery	Not categorized	Other - Matcho et al	X	X					
4201423	Wilson-Mikity syndrome	Not categorized	Other - Matcho et al	X						
4101126	Acquired neutropenia in newborn	Not categorized	Other - Matcho et al	X						
4065747	Intrapartum hemorrhage with coagulation defect	Not categorized	Other - Matcho et al	X						
4149609	Birth weight 1000-2499 g	Not categorized	Other - Matcho et al	X						
4108901	Embryonic cyst of cervix	Not categorized	Other - Matcho et al	X						
4061850	Obstructed labor due to face presentation	Not categorized	Other - Matcho et al	X						
4118057	Fetus or neonate affected by maternal oligohydramnios	Not categorized	Other - Matcho et al	X	X					
4170869	Dehydration fever in newborn	Not categorized	Other - Matcho et al	X						
4049042	Newborn drug reaction and intoxication	Not categorized	Other - Matcho et al	X						
4149586	Perinatal massive pulmonary hemorrhage	Not categorized	Other - Matcho et al	X						
4065626	Toxic reaction to local anesthetic during labor and delivery	Not categorized	Other - Matcho et al	X						
4058112	Glycosuria during pregnancy - not delivered	Not categorized	Other - Matcho et al	X						
4327033	Transitory neonatal tyrosinaemia	Not categorized	Other - Matcho et al	X						
4048129	Fetus and newborn affected by maternal use of nutritional chemical substances	Not categorized	Other - Matcho et al	X						
4129839	Obstetric pelvic joint damage	Not categorized	Other - Matcho et al	X						
4064725	Malnutrition in pregnancy	Not categorized	Other - Matcho et al	X	X					
4071745	Disturbances of potassium balance of newborn	Not categorized	Other - Matcho et al	X	X					
4064423	Obstructed labour due to pelvic inlet contraction	Not categorized	Other - Matcho et al	X						
4048149	Congenital pseudomonal pneumonia	Not categorized	Other - Matcho et al	X						
43530885	Suspected fetal damage from maternal toxoplasmosis	Not categorized	Other - Matcho et al	X						
4070521	Disorders due to slow fetal growth, low and high birth weight	Not categorized	Other - Matcho et al	X	X					
4005284	Foetus and newborn affected by maternal use of alcohol	Not categorized	Other - Matcho et al	X	X					
4119149	Absent blood vessel in umbilical cord	Not categorized	Other - Matcho et al	X						
4047725	Fetus small-for-dates with signs of malnutrition	Not categorized	Other - Matcho et al	X						
4146725	Foetus or neonate affected by maternal renal or urinary disease	Not categorized	Other - Matcho et al	X	X					
4065094	Central nervous system complications of anaesthesia during labour and delivery	Not categorized	Other - Matcho et al	X						
4320940	Hyperthermia in newborn	Not categorized	Other - Matcho et al	X						
4241991	Embryonal sarcoma	Not categorized	Other - Matcho et al	X						
4174548	Catheterisation of umbilical vein	Not categorized	Other - Matcho et al	X	X					
443326	Mild hyperemesis-delivered	Not categorized	Other - Matcho et al	X						
4060039	Obstructed labor due to generally contracted pelvis	Not categorized	Other - Matcho et al	X						
4058113	Gestational edema with proteinuria	Not categorized	Other - Matcho et al	X	X					
4062926	Herpes gestationis - not delivered	Not categorized	Other - Matcho et al	X						
4065866	Caesarean wound disruption with postnatal complication	Not categorized	Other - Matcho et al	X						
4289756	Fetal hydantoin syndrome	Not categorized	Other - Matcho et al	X						
4048927	Transitory neonatal hypokalaemia	Not categorized	Other - Matcho et al	X	X					
4047587	Fetus or neonate affected by placental or breast transfer of narcotics	Not categorized	Other - Matcho et al	X	X					
4143079	Tumor of uterine body affecting obstetric care	Not categorized	Other - Matcho et al	X						
4048148	Congenital Escherichia coli pneumonia	Not categorized	Other - Matcho et al	X						
4147342	Obstetric anesthesia with pulmonary complications	Not categorized	Other - Matcho et al	X						
4091321	Obstetrical tetanus	Not categorized	Other - Matcho et al	X						
4193784	Expanded rubella syndrome	Not categorized	Other - Matcho et al	X						
200791	Congenital or acquired abnormality of vagina in pregnancy, childbirth and the puerperium	Not categorized	Other - Matcho et al	X						
4290195	Congenital tuberculosis	Not categorized	Other - Matcho et al	X						
199088	First degree perineal tear during delivery with postnatal problem	Not categorized	Other - Matcho et al	X	X					
4322190	Perinatal jaundice due to fetal OR neonatal hepatitis	Not categorized	Other - Matcho et al	X						
4060299	Fatigue during pregnancy - not delivered	Not categorized	Other - Matcho et al	X						
4062269	Cerebral venous thrombosis in pregnancy	Not categorized	Other - Matcho et al	X						
4118055	Fetus or neonate affected by maternal incompetent cervix	Not categorized	Other - Matcho et al	X						
4070540	Congenital chlamydial pneumonia	Not categorized	Other - Matcho et al	X						
4064723	Abnormal radiological finding on antenatal screening of mother	Not categorized	Other - Matcho et al	X	X					
4064981	Obstetric anesthesia with central nervous system complications	Not categorized	Other - Matcho et al	X						
4173186	Iatrogenic neonatal hypoglycemia	Not categorized	Other - Matcho et al	X						
4048738	Disturbances of sodium balance of newborn	Not categorized	Other - Matcho et al	X	X					
4070415	Fetus or neonate affected by chorioamnionitis	Not categorized	Other - Matcho et al	X	X					
4048606	Cerebellar (nontraumatic) and posterior fossa haemorrhage of foetus and newborn	Not categorized	Other - Matcho et al	X	X					
4150396	Baby BW = 751g-1kg	Not categorized	Other - Matcho et al	X	X					
1521369	norethindrone	CONTRA	Other - Specific to initial possibly-pregnant cohort (633K)	X			X			
1500211	medroxyprogesterone	CONTRA	Other - Specific to initial possibly-pregnant cohort (633K)	X			X			

1519936	etonogestrel	CONTRA	Other - Specific to initial possibly-pregnant cohort (633K)	X			X			
1552310	progesterone	OVULDR	Other - Specific to initial possibly-pregnant cohort (633K)	X			X			
1598819	clomiphene	OVULDR	Other - Specific to initial possibly-pregnant cohort (633K)	X			X			
1563600	chorionic gonadotropin	OVULDR	Other - Specific to initial possibly-pregnant cohort (633K)	X			X			
4290245	Trial forceps delivery		Delivery record only (DELIV)	X				X		
4266683	Mid forceps delivery	DELIV	Delivery record only (DELIV)	X				X		
4232028	Delivery by Kielland rotation	DELIV	Delivery record only (DELIV)	X				X		
4213387	Breech extraction	LDEL	Delivery record only (DELIV)	X				X		
4205240	Spontaneous vertex delivery	DELIV	Delivery record only (DELIV)	X				X		
4189205	Delivery by vacuum extraction	DELIV	Delivery record only (DELIV)	X				X		
4173513	Neville-Barnes forceps delivery	LDEL	Delivery record only (DELIV)	X				X		
4170152	Simpson's forceps delivery		Delivery record only (DELIV)	X				X		
4127248	Nonrotational forceps delivery		Delivery record only (DELIV)	X				X		
4075191	Low vacuum delivery		Delivery record only (DELIV)	X				X		
4075189	Midforceps cephalic delivery with rotation		Delivery record only (DELIV)	X				X		
4075188	High forceps cephalic delivery with rotation		Delivery record only (DELIV)	X				X		
4075170	High vacuum delivery		Delivery record only (DELIV)	X				X		
4075169	Barton forceps cephalic delivery with rotation		Delivery record only (DELIV)	X				X		
4073424	Trial of vacuum delivery		Delivery record only (DELIV)	X				X		
4073422	Spontaneous breech delivery	DELIV	Delivery record only (DELIV)	X				X		
4071630	Non-manipulative cephalic vaginal delivery with abnormal presentation of head at delivery without instrument		Delivery record only (DELIV)	X				X		
4063162	Multiple delivery, all by forceps and vacuum extractor		Delivery record only (DELIV)	X				X		
4063160	Normal delivery but ante- or post- natal conditions present	DELIV	Delivery record only (DELIV)	X				X		
4062136	Delivered by mid-cavity forceps with rotation		Delivery record only (DELIV)	X				X		
4034145	Complete breech delivery	DELIV	Delivery record only (DELIV)	X				X		
2004710	Other high forceps operation		Delivery record only (DELIV)	X				X		
2004702	Forceps, vacuum, and breech delivery		Delivery record only (DELIV)	X				X		
438481	Quadruplet pregnancy - delivered	DELIV	Delivery record only (DELIV)	X				X		
4064559	Delayed delivery second twin - delivered		Delivery record only (DELIV)	X				X		
442422	Locked twins - delivered		Delivery record only (DELIV)	X				X		
4211824	Extraperitoneal cesarean section		Delivery record only (DELIV)	X				X		
4075161	Elective lower segment cesarean section	DELIV	Delivery record only (DELIV)	X				X		
4075160	Elective upper segment cesarean section		Delivery record only (DELIV)	X				X		
2101016	Neuraxial analgesia/anesthesia for labor ending in a cesarean delivery (includes any repeat subarachnoid needle placement and drug injection and/or any necessary replacement of an epidural catheter during labor)	DELIV	Delivery record only (DELIV)	X				X		
2101013	Anesthesia for intraperitoneal procedures in lower abdomen including laparoscopy; cesarean section		Delivery record only (DELIV)	X				X		
2004786	Low cervical cesarean section	DELIV	Delivery record only (DELIV)	X				X		
4129700	Cystocele - delivered with postpartum complication		Delivery record only (DELIV)	X				X		
4064426	Persistent occipitoposterior or occipitoanterior position - delivered		Delivery record only (DELIV)	X				X		
4064177	Fetus with drug damage - delivered	DELIV	Delivery record only (DELIV)	X				X		
4064174	Fetus with viral damage via mother - delivered	DELIV	Delivery record only (DELIV)	X				X		
4063305	Fetal distress - delivered		Delivery record only (DELIV)	X				X		
4063296	Fetus with damage due to intrauterine contraceptive device - delivered	DELIV	Delivery record only (DELIV)	X				X		
4063171	Transverse lie - delivered	DELIV	Delivery record only (DELIV)	X				X		
4062268	Hemorrhoids in pregnancy and the puerperium - delivered		Delivery record only (DELIV)	X				X		
4060539	Face presentation - delivered		Delivery record only (DELIV)	X				X		
4028632	Rectocele - delivered with postpartum complication		Delivery record only (DELIV)	X				X		
443324	Papyraceous fetus - delivered		Delivery record only (DELIV)	X				X		
442828	Fetus with central nervous system malformation - delivered		Delivery record only (DELIV)	X				X		
442420	Obstetric pyemic and septic pulmonary embolism - delivered		Delivery record only (DELIV)	X				X		
442052	Obstetric air pulmonary embolism - delivered with postnatal complication		Delivery record only (DELIV)	X				X		
442048	Obstetric pyemic and septic pulmonary embolism - delivered with postnatal complication		Delivery record only (DELIV)	X				X		
441924	Failed mechanical induction - delivered	DELIV	Delivery record only (DELIV)	X				X		
441643	Maternal distress - delivered with postnatal problem		Delivery record only (DELIV)	X				X		
440475	Prolonged first stage - delivered	DELIV	Delivery record only (DELIV)	X				X		
439095	Superficial thrombophlebitis in pregnancy and the puerperium - delivered with postnatal complication	DELIV	Delivery record only (DELIV)	X				X		
435607	Cervical incompetence - delivered with postnatal complication		Delivery record only (DELIV)	X				X		
435330	Varicose veins of legs in pregnancy and the puerperium - delivered with postnatal complication		Delivery record only (DELIV)	X				X		
434112	Obstetric blood-clot pulmonary embolism - delivered with postnatal complication		Delivery record only (DELIV)	X				X		
433276	Retracted nipple in pregnancy, the puerperium or lactation - delivered with postnatal complication		Delivery record only (DELIV)	X				X		
432388	Amniotic fluid pulmonary embolism - delivered with postnatal complication		Delivery record only (DELIV)	X				X		
200157	Renal hypertension complicating pregnancy, childbirth and the puerperium - delivered	DELIV	Delivery record only (DELIV)	X				X		
198486	Disproportion - major pelvic abnormality - delivered	DELIV	Delivery record only (DELIV)	X				X		
197616	Retroverted incarcerated gravid uterus - delivered with postnatal complication		Delivery record only (DELIV)	X				X		
197042	Peripheral neuritis in pregnancy - delivered		Delivery record only (DELIV)	X				X		
195025	Obstetric shock - delivered with postnatal problem	DELIV	Delivery record only (DELIV)	X				X		
194113	Cesarean wound disruption - delivered with postnatal complication	POST	Other - Matcho et al	X				X		
77621	Inlet pelvic contraction - delivered	DELIV	Delivery record only (DELIV)	X				X		
77052	Generally contracted pelvis - delivered	DELIV	Delivery record only (DELIV)	X				X		
76769	Suppressed lactation - delivered with postnatal complication		Delivery record only (DELIV)	X				X		

75326	Obstetric damage to pelvic joints and ligaments - delivered	DELIV	Delivery record only (DELIV)	X				X		
73824	Outlet pelvic contraction - delivered	DELIV	Delivery record only (DELIV)	X				X		
72692	Prolapsed arm - delivered	DELIV	Delivery record only (DELIV)	X				X		
4152444	Antenatal ultrasound confirms ectopic pregnancy	ECT	Ectopic pregnancy (ECT)	X				X		
4300368	Aspiration of ectopic pregnancy from fallopian tube	ECT_SURG1	Ectopic pregnancy (ECT)	X				X		
4306064	Fimbrial extraction of tubal pregnancy		Ectopic pregnancy (ECT)	X				X		
4112952	Membranous pregnancy		Ectopic pregnancy (ECT)	X				X		
193532	Incomplete legal termination of pregnancy with genital tract or pelvic infection		Induced abortion (AB)	X				X		
193821	Illegal termination of pregnancy complicated by embolism		Induced abortion (AB)	X				X		
195595	Legal termination of pregnancy complicated by renal failure		Induced abortion (AB)	X				X		
195600	Illegal termination of pregnancy complicated by renal failure		Induced abortion (AB)	X				X		
195870	Complete illegal termination of pregnancy with genital tract or pelvic infection		Induced abortion (AB)	X				X		
196754	Complete legal termination of pregnancy with genital tract or pelvic infection		Induced abortion (AB)	X				X		
434095	Incomplete legal termination of pregnancy with metabolic disorder		Induced abortion (AB)	X				X		
434096	Complete illegal termination of pregnancy with delayed or excessive hemorrhage		Induced abortion (AB)	X				X		
434705	Incomplete legal termination of pregnancy with damage to pelvic organs or tissues		Induced abortion (AB)	X				X		
435008	Complete legal termination of pregnancy with renal failure		Induced abortion (AB)	X				X		
435017	Complete legal termination of pregnancy with metabolic disorder		Induced abortion (AB)	X				X		
435869	Illegal termination of pregnancy		Induced abortion (AB)	X				X		
435871	Complete illegal termination of pregnancy with renal failure		Induced abortion (AB)	X				X		
436169	Illegal termination of pregnancy with complication		Induced abortion (AB)	X				X		
436474	Legal termination of pregnancy complicated by metabolic disorder		Induced abortion (AB)	X				X		
437929	Incomplete illegal termination of pregnancy with delayed or excessive hemorrhage		Induced abortion (AB)	X				X		
437934	Complete legal termination of pregnancy with shock		Induced abortion (AB)	X				X		
438487	Incomplete illegal termination of pregnancy with embolism		Induced abortion (AB)	X				X		
439310	Illegal termination of pregnancy, complete		Induced abortion (AB)	X				X		
439316	Complete illegal termination of pregnancy with embolism		Induced abortion (AB)	X				X		
439318	Complete illegal termination of pregnancy with metabolic disorder		Induced abortion (AB)	X				X		
439319	Complete illegal termination of pregnancy with damage to pelvic organs or tissues		Induced abortion (AB)	X				X		
439320	Incomplete illegal termination of pregnancy with shock		Induced abortion (AB)	X				X		
439321	Incomplete illegal termination of pregnancy with metabolic disorder		Induced abortion (AB)	X				X		
439322	Incomplete illegal termination of pregnancy with renal failure		Induced abortion (AB)	X				X		
439323	Incomplete illegal termination of pregnancy with damage to pelvic organs or tissues		Induced abortion (AB)	X				X		
439324	Incomplete illegal termination of pregnancy with genital tract or pelvic infection		Induced abortion (AB)	X				X		
439327	Complete legal termination of pregnancy with embolism		Induced abortion (AB)	X				X		
439329	Incomplete legal termination of pregnancy with embolism		Induced abortion (AB)	X				X		
439331	Incomplete legal termination of pregnancy with renal failure		Induced abortion (AB)	X				X		
439400	Illegal termination of pregnancy complicated by genital-pelvic infection		Induced abortion (AB)	X				X		
441911	Complete legal termination of pregnancy with damage to pelvic organs or tissues		Induced abortion (AB)	X				X		
442596	Illegal termination of pregnancy complicated by shock		Induced abortion (AB)	X				X		
443705	Illegal termination of pregnancy complicated by metabolic disorder		Induced abortion (AB)	X				X		
444048	Illegal termination of pregnancy complicated by damage to pelvic organs and/or tissues		Induced abortion (AB)	X				X		
2004489	Dilation and curettage for termination of pregnancy	AB	Induced abortion (AB)	X				X		
2110333	Induced abortion, by 1 or more intra-amniotic injections (amniocentesis-injections), including hospital admission and visits, delivery of fetus and secundines; with hysterotomy (failed intra-amniotic injection)		Induced abortion (AB)	X				X		
4074301	Insertion of prostaglandin abortifacient suppository		Induced abortion (AB)	X				X		
4082412	Hysterotomy and termination of pregnancy	AB	Induced abortion (AB)	X				X		
4112315	Insertion of abortifacient suppository		Induced abortion (AB)	X				X		
4113503	Readmission for retained products of conception, legal termination of pregnancy	SA	Induced abortion (AB)	X				X		
4114271	Readmission for retained products of conception, illegal termination of pregnancy		Induced abortion (AB)	X				X		
4189561	Therapeutic termination of pregnancy by aspiration curettage	SA	Induced abortion (AB)	X				X		
2110326	Treatment of missed abortion, completed surgically; first trimester	SA	Spontaneous abortion (SA)	X	X	X	X	X		
2110327	Treatment of missed abortion, completed surgically; second trimester	SA	Spontaneous abortion (SA)	X	X	X	X	X		
2110328	Treatment of septic abortion, completed surgically	SA	Spontaneous abortion (SA)	X	X					
4001856	Miscarriage with renal tubular necrosis		Spontaneous abortion (SA)					X		
4003276	Miscarriage with amniotic fluid embolism		Spontaneous abortion (SA)					X		
4006806	Miscarriage with uremia		Spontaneous abortion (SA)					X		
4031837	Miscarriage with perforation of broad ligament		Spontaneous abortion (SA)					X		
4032621	Evacuation of retained product of conception	SA	Spontaneous abortion (SA)	X	X					
4211824	Extraperitoneal cesarean section		Cesarean section	X						
4075161	Elective lower segment cesarean section	DELIV	Cesarean section	X						
4075160	Elective upper segment cesarean section		Cesarean section	X						
2101016	Neuraxial analgesia/anesthesia for labor ending in a cesarean delivery (includes any repeat subarachnoid needle placement and drug injection and/or any necessary replacement of an epidural catheter during labor)	DELIV	Cesarean section	X						
2101013	Anesthesia for intraperitoneal procedures in lower abdomen including laparoscopy; cesarean section		Cesarean section	X						

2004786	Low cervical cesarean section	DELIV	Cesarean section	X						
4058439	Last menstrual period -1st day	LMP	Gestational age, other	X						
4128833	Estimated date of delivery from last period	AGP	Gestational age, other	X						
4061786	A/N care categorized by gravida number	AGP	Gravidity	X						
4064559	Delayed delivery second twin - delivered		Multiple pregnancy	X						
4143537	Endoscopic serial drainage of amniotic fluid for twin-twin transfusion syndrome		Multiple pregnancy	X						
4118398	Fetus or neonate affected by triplet pregnancy		Multiple pregnancy	X						
4118413	Fetus or neonate affected by twin pregnancy		Multiple pregnancy	X						
4070411	Fetus or neonate affected by twin-to-twin transplacental transfusion		Multiple pregnancy	X						
4252738	Head-joined twins		Multiple pregnancy	X						
442422	Locked twins - delivered		Multiple pregnancy	X						
72700	Locked twins with antenatal problem		Multiple pregnancy	X						
4063162	Multiple delivery, all by forceps and vacuum extractor		Multiple pregnancy	X						
4059751	Multiple delivery, all spontaneous		Multiple pregnancy	X						
4171599	On examination - triplet presentation		Multiple pregnancy	X						
4169639	On examination - twin presentation		Multiple pregnancy	X						
4014458	One male and one female baby		Multiple pregnancy	X						
4014457	Two female babies		Multiple pregnancy	X						
4146866	On examination - multiple presentation		Multiple pregnancy	X						
4193698	Identical twin		Multiple pregnancy	X						
4015273	1 male + 2 female babies		Multiple pregnancy	X						
4014297	2 male babies		Multiple pregnancy	X						
4015272	3 male babies		Multiple pregnancy	X						
438481	Quadruplet pregnancy - delivered	DELIV	Multiple pregnancy	X						
4060248	A/N care: primiparous, older than 30 years	AGP	Parity	X						
4014299	Baby premature 36-38 weeks	PREM	Preterm pregnancy	X						
4014461	Baby birth weight equal to 50%-74% (3450-3749g)	PREM	Preterm pregnancy	X						
42740403	Subsequent intensive care, per day, for the evaluation and management of the recovering low birth weight infant (present body weight of 1500-2500 g)	PREM	Preterm pregnancy	X						
442923	Anaemia in the puerperium - baby delivered during current episode of care	DELIV	Delivery record only (DELIV)	X						
442078	Retained placenta with no hemorrhage - delivered with postnatal problem	DELIV	Delivery record only (DELIV)	X						
4155282	Abnormal glucose tolerance test in the puerperium - baby delivered during current episode of care	DELIV	Delivery record only (DELIV)	X						
441082	Late pregnancy vomiting - delivered	DELIV	Delivery record only (DELIV)	X						
2004742	Unspecified instrumental delivery	DELIV	Delivery record only (DELIV)	X						
315013	Congenital cardiovascular disorder during pregnancy - baby delivered	DELIV	Delivery record only (DELIV)	X						
441087	Vulval and/or perineal haematoma during delivery - delivered	DELIV	Delivery record only (DELIV)	X						
443321	Edema or excessive weight gain in pregnancy without mention of hypertension, delivered with mention of postpartum complication	DELIV	Delivery record only (DELIV)	X						
138207	Thyroid dysfunction in the puerperium - baby delivered during current episode of care	DELIV	Delivery record only (DELIV)	X						
435879	Congenital or acquired abnormality of vulva - baby delivered with postpartum complication	DELIV	Delivery record only (DELIV)	X						
4014291	Birth detail	DELIV	Delivery record only (DELIV)	X						
40767416	Was your pregnancy a live birth, stillbirth, miscarriage, abortion, or ectopic pregnancy PhenX	SA	Delivery record only (DELIV)	X						
4071507	Normal delivery procedure	DELIV	Delivery record only (DELIV)	X						
40760193	Labor and delivery process	DELIV	Delivery record only (DELIV)	X						
4161944	Low cervical caesarean section	DELIV	Delivery record only (DELIV)	X						
4015270	Birth of child	DELIV	Delivery record only (DELIV)	X						
4014738	Child examination - birth	DELIV	Delivery record only (DELIV)	X						
40394878	[X]Delivery	DELIV	Delivery record only (DELIV)	X						
4015277	Birth head circumference	DELIV	Delivery record only (DELIV)	X						
4014451	Home birth	DELIV	Delivery record only (DELIV)	X						
4014292	Born - place delivered	DELIV	Delivery record only (DELIV)	X						
4248954	Amniotomy at delivery	DELIV	Delivery record only (DELIV)	X						
4152029	Childbirth examination - normal	DELIV	Delivery record only (DELIV)	X						
4014452	GP unit birth	DELIV	Delivery record only (DELIV)	X						
4016064	Birth details not known	DELIV	Delivery record only (DELIV)	X						
4069969	Manual removal of placenta from delivered uterus	DELIV	Delivery record only (DELIV)	X						
4071632	Water birth delivery	DELIV	Delivery record only (DELIV)	X						
4092760	Sex of baby at delivery	DELIV	Delivery record only (DELIV)	X						
4071629	Cephalic vaginal delivery with abnormal presentation of head at delivery without instrument	DELIV	Delivery record only (DELIV)	X						
4038756	O/E -fundus 38 weeks-term size	FT	Full term	X						
4014149	Antenatal 16 week examination	GEST	Gestation period, X weeks (GW)	X						
4015141	Antenatal 24 week examination	GEST	Gestation period, X weeks (GW)	X						
4015298	Antenatal 34 week examination	GEST	Gestation period, X weeks (GW)	X						
4038934	O/E - fundus 36-38 week size	GEST	Gestation period, X weeks (GW)	X						
4015297	Antenatal 32 week examination	GEST	Gestation period, X weeks (GW)	X						
4014152	Antenatal 38 week examination	GEST	Gestation period, X weeks (GW)	X						
4015299	Antenatal 36 week examination	GEST	Gestation period, X weeks (GW)	X						
4038754	O/E - fundus 32-34 week size	GEST	Gestation period, X weeks (GW)	X						
4014151	Antenatal 30 week examination	GEST	Gestation period, X weeks (GW)	X						
4015142	Antenatal 37 week examination	GEST	Gestation period, X weeks (GW)	X						
4015300	Antenatal 39 week examination	GEST	Gestation period, X weeks (GW)	X						
4014150	Antenatal 20 week examination	GEST	Gestation period, X weeks (GW)	X						
4015301	Antenatal 40 week examination	GEST	Gestation period, X weeks (GW)	X						
4014434	Antenatal 35 week examination	GEST	Gestation period, X weeks (GW)	X						
4015296	Antenatal 12 weeks examination	GEST	Gestation period, X weeks (GW)	X						

4015302	Antenatal 41 week examination	GEST	Gestation period, X weeks (GW)	X						
2004525	Aspiration curettage of uterus for termination of pregnancy	AB	Induced abortion (AB)	X						
4086797	Requests pregnancy termination	AB	Induced abortion (AB)	X						
4190767	Referral for termination of pregnancy	AB	Induced abortion (AB)	X						
4113976	Legally induced abortion NOS	AB	Induced abortion (AB)	X						
4106559	Dilatation and evacuation termination of pregnancy	AB	Induced abortion (AB)	X						
4150401	Reason for termination of pregnancy	AB	Induced abortion (AB)	X						
40767416	Was your pregnancy a live birth, stillbirth, miscarriage, abortion, or ectopic pregnancy PhenX	SA	Livebirth (LB)	X						
40767416	Was your pregnancy a live birth, stillbirth, miscarriage, abortion, or ectopic pregnancy PhenX	SA	Livebirth (LB)	X						
3046979	Alpha-1-Fetoprotein [Multiple of the median] adjusted for weight in Serum or Plasma	AFP	Other - Matcho et al	X						
4014768	Alpha-1-fetoprotein measurement, serum	AFP	Other - Matcho et al	X						
4195345	Serum alpha-fetoprotein multiple of median measurement	AFP	Other - Matcho et al	X						
4042555	Alpha-fetoprotein normal	AFP	Other - Matcho et al	X						
4210719	Plasma alpha-fetoprotein multiple of median measurement	AFP	Other - Matcho et al	X						
4078285	AFP test - antenatal	AFP	Other - Matcho et al	X						
4078287	Triple test	AFP	Other - Matcho et al	X						
4310471	MS alpha-fetoprotein level	AFP	Other - Matcho et al	X						
4099476	Alpha-1-Fetoprotein measurement, amniotic fluid	AFP	Other - Matcho et al	X						
4059689	Alpha-fetoprotein radioimmunoassay	AFP	Other - Matcho et al	X						
4061810	AFP - blood sent	AFP	Other - Matcho et al	X						
4147940	Alpha-fetoprotein blood test	AFP	Other - Matcho et al	X						
4061809	AFP blood test not wanted	AFP	Other - Matcho et al	X						
4042936	Blood sent: alpha-fetoprotein	AFP	Other - Matcho et al	X						
4076939	Delivery booking place	AGP	Other - Matcho et al	X						
4038748	O/E - uterus size - obstetric	AGP	Other - Matcho et al	X						
4083415	Seen in antenatal clinic	AGP	Other - Matcho et al	X						
4038573	O/E - VE for pelvic assessment	AGP	Other - Matcho et al	X						
4038747	Obstetric examination	AGP	Other - Matcho et al	X						
4060244	A/N care: social risk	AGP	Other - Matcho et al	X						
4081292	Referral to antenatal clinic	AGP	Other - Matcho et al	X						
4014148	Antenatal booking examination	AGP	Other - Matcho et al	X						
4060252	Consultant unit booking	AGP	Other - Matcho et al	X						
4176966	Prenatal education	AGP	Other - Matcho et al	X						
4038945	O/E - fetal heart heard	AGP	Other - Matcho et al	X						
4016475	Placenta normal O/E	AGP	Other - Matcho et al	X						
4014433	Antenatal 28 week examination	AGP	Other - Matcho et al	X						
4038753	O/E - fundus 28-32 week size	AGP	Other - Matcho et al	X						
4015159	Consultant unit birth	AGP	Other - Matcho et al	X						
4039602	O/E - fundus 24-28 week size	AGP	Other - Matcho et al	X						
4038932	O/E - fundus 20-24 week size	AGP	Other - Matcho et al	X						
4038751	O/E - fundus 16-20 week size	AGP	Other - Matcho et al	X						
4147564	Antenatal care midwifery led	AGP	Other - Matcho et al	X						
4038935	O/E - fundus = term size	AGP	Other - Matcho et al	X						
4038749	O/E - fundus 12-16 week size	AGP	Other - Matcho et al	X						
4061521	A/N - shared care	AGP	Other - Matcho et al	X						
4305717	Midwife unit delivery booking	AGP	Other - Matcho et al	X						
4060255	Feeding intention - breast	AGP	Other - Matcho et al	X						
4039603	O/E - vertex presentation	AGP	Other - Matcho et al	X						
4140250	Obstetric monitoring	AGP	Other - Matcho et al	X						
4147941	Misc. antenatal data	AGP	Other - Matcho et al	X						
4060247	A/N care: medical risk	AGP	Other - Matcho et al	X						
4059987	A/N care: uncertain dates	AGP	Other - Matcho et al	X						
4156955	Delivery place booked	AGP	Other - Matcho et al	X						
4038418	O/E - fundal size = dates	AGP	Other - Matcho et al	X						
4060104	Home delivery booked	AGP	Other - Matcho et al	X						
4084439	Fetal heart monitoring	AGP	Other - Matcho et al	X						
4038571	O/E - fetal heart 120-160	AGP	Other - Matcho et al	X						
4061437	A/N care from consultant	AGP	Other - Matcho et al	X						
4061425	Antenatal care of 2nd pregnancy	AGP	Other - Matcho et al	X						
4171428	On examination - presenting part engaged	AGP	Other - Matcho et al	X						
4084186	Antenatal exercises	AGP	Other - Matcho et al	X						
4038941	O/E - presenting part free-5/5	AGP	Other - Matcho et al	X						
4190427	Antenatal clinic	AGP	Other - Matcho et al	X						
4039609	O/E - no fetal movements	AGP	Other - Matcho et al	X						
4061793	A/N care provider	AGP	Other - Matcho et al	X						
4062361	Antenatal relaxation class	AGP	Other - Matcho et al	X						
4260976	Seen in foetal medicine clinic	AGP	Other - Matcho et al	X						
4084521	Obstetric domiciliary visit done	AGP	Other - Matcho et al	X						
40525253	[V]Unspecified antenatal screening	AGP	Other - Matcho et al	X						
4175225	On examination - presenting part free - not engaged	AGP	Other - Matcho et al	X						
4061787	Antenatal care of 3rd pregnancy	AGP	Other - Matcho et al	X						
40517216	[V]Pregnancy examination and test	AGP	Other - Matcho et al	X						
4060240	A/N care: recurrent aborter	AGP	Other - Matcho et al	X						
4038424	O/E - presentation engaged-1/5	AGP	Other - Matcho et al	X						
4039088	O/E - fetal heart not heard	AGP	Other - Matcho et al	X						
4150421	Delivery place planned	AGP	Other - Matcho et al	X						
4038759	O/E - partial engagement - 3/5	AGP	Other - Matcho et al	X						
4061795	Delivery: no place booked	AGP	Other - Matcho et al	X						
4194143	On examination - viable fetus	AGP	Other - Matcho et al	X						
4060251	A/N care from G.P.	AGP	Other - Matcho et al	X						
4038760	O/E - fetus very active	AGP	Other - Matcho et al	X						
4038939	O/E - presenting part position	AGP	Other - Matcho et al	X						
4066354	Maternal care for intrauterine growth retardation	AGP	Other - Matcho et al	X						
4038938	O/E -fetal presentation unsure	AGP	Other - Matcho et al	X						
4039608	O/E - presenting part free-4/5	AGP	Other - Matcho et al	X						
4039610	O/E - fetal movements seen	AGP	Other - Matcho et al	X						
4151190	Home birth planned	AGP	Other - Matcho et al	X						
4061789	A/N care: precious pregnancy	AGP	Other - Matcho et al	X						
4060098	A/N care: poor home conditions	AGP	Other - Matcho et al	X						
4060105	Private home delivery booking	AGP	Other - Matcho et al	X						
4081196	Antepartum	AGP	Other - Matcho et al	X						

2514574	Home visit for prenatal monitoring and assessment to include fetal heart rate, non-stress test, uterine monitoring, and gestational diabetes monitoring	AGP	Other - Matcho et al	X							
4060259	A/N amniocentesis - abnormal	AMNIO	Other - Matcho et al	X							
2004805	Diagnostic amniocentesis	AMNIO	Other - Matcho et al	X							
3013297	Phosphatidylglycerol [Presence] in Amniotic fluid	AMNIO	Other - Matcho et al	X							
3035904	Lecithin/Sphingomyelin [Ratio] in Amniotic fluid	AMNIO	Other - Matcho et al	X							
3017439	Surfactant/Albumin [Mass ratio] in Amniotic fluid	AMNIO	Other - Matcho et al	X							
4061530	A/N amniocentesis - normal	AMNIO	Other - Matcho et al	X							
969444	Nonoxynol-9	CONTRA	Other - Matcho et al	X							
970277	Octoxynol-9	CONTRA	Other - Matcho et al	X							
1503184	Mestranol	CONTRA	Other - Matcho et al	X							
1521592	Norgestrel	CONTRA	Other - Matcho et al	X							
1589505	Levonorgestrel	CONTRA	Other - Matcho et al	X							
1595461	ethynodiol	CONTRA	Other - Matcho et al	X							
40225722	ulipristal	CONTRA	Other - Matcho et al	X							
43077184	norgestimate	CONTRA	Other - Matcho et al	X							
1515774	norgestimate	CONTRA	Other - Matcho et al	X							
1549786	Ethinyl Estradiol	CONTRA	Other - Matcho et al	X							
1588000	Desogestrel	CONTRA	Other - Matcho et al	X							
19092358	Lynestrenol	CONTRA	Other - Matcho et al	X							
19128258	Norgestrienone	CONTRA	Other - Matcho et al	X							
3003169	Glucose tolerance 2 hours gestational panel in Urine and Serum or Plasma	DIAB	Other - Matcho et al	X							
3033461	Insulin [Mass/volume] in Serum or Plasma --2 hours post 75 g glucose PO	DIAB	Other - Matcho et al	X							
3015988	Insulin [Mass/volume] in Serum or Plasma --3 hours post 75 g glucose PO	DIAB	Other - Matcho et al	X							
3011355	Glucose [Presence] in Urine by Test strip --2 hours post 75 g glucose PO	DIAB	Other - Matcho et al	X							
3005370	Glucose [Presence] in Urine by Test strip --3 hours post 75 g glucose PO	DIAB	Other - Matcho et al	X							
3008406	Insulin [Mass/volume] in Serum or Plasma --4 hours post 75 g glucose PO	DIAB	Other - Matcho et al	X							
3017118	Insulin [Mass/volume] in Serum or Plasma --5 hours post 75 g glucose PO	DIAB	Other - Matcho et al	X							
4012477	OGTT - Oral glucose tolerance test	DIAB	Other - Matcho et al	X							
4055678	Glucose tolerance test normal	DIAB	Other - Matcho et al	X							
4041698	Laboratory blood sugar	DIAB	Other - Matcho et al	X							
4301905	USO - Unilateral salpingo-oophorectomy	ECT_SURG1	Other - Matcho et al	X							
4336229	Unilateral salpingectomy	ECT_SURG1	Other - Matcho et al	X							
4336229	Unilateral salpingectomy	ECT_SURG1	Other - Matcho et al	X							
4190526	Laparoscopic total abdominal hysterectomy and bilateral salpingo-oophorectomy	ECT_SURG1	Other - Matcho et al	X							
4074443	Endoscopic freeing of adhesions of fallopian tube	ECT_SURG1	Other - Matcho et al	X							
40480864	Laparoscopy assisted vaginal hysterectomy with bilateral salpingo-oophorectomy	ECT_SURG1	Other - Matcho et al	X							
4074427	Excision of ruptured ectopic tubal pregnancy	ECT_SURG1	Other - Matcho et al	X							
4267567	Partial salpingectomy	ECT_SURG1	Other - Matcho et al	X							
4073272	Salpingo-oophorectomy of remaining solitary fallopian tube and ovary	ECT_SURG1	Other - Matcho et al	X							
4032000	Salpingectomy of remaining fallopian tube	ECT_SURG1	Other - Matcho et al	X							
2722217	Laparoscopy, surgical; with salpingostomy (salpingoneostomy).	ECT_SURG1	Other - Matcho et al	X							
2722205	Laparoscopy, surgical; with removal of adnexal structures (partial or total oophorectomy and/or salpingectomy).	ECT_SURG1	Other - Matcho et al	X							
2004362	Salpingo-salpingostomy	ECT_SURG1	Other - Matcho et al	X							
2004351	Salpingo-oophorostomy	ECT_SURG1	Other - Matcho et al	X							
2004347	Bilateral partial salpingectomy, not otherwise specified	ECT_SURG1	Other - Matcho et al	X							
2004346	Salpingectomy with removal of tubal pregnancy	ECT_SURG1	Other - Matcho et al	X							
2004331	Total bilateral salpingectomy	ECT_SURG1	Other - Matcho et al	X							
2004308	Salpingostomy	ECT_SURG1	Other - Matcho et al	X							
2004307	Salpingotomy	ECT_SURG1	Other - Matcho et al	X							
2004306	Salpingotomy and salpingostomy	ECT_SURG1	Other - Matcho et al	X							
40757216	Discharge location CARE	ECT_SURG2	Other - Matcho et al	X							
40757177	Discharge Status	ECT_SURG2	Other - Matcho et al	X							
4032749	Laparoscopy of fallopian tube	ECT_SURG2	Other - Matcho et al	X							
40347147	Laparoscopic	ECT_SURG2	Other - Matcho et al	X							
40378260	Laparoscopic approach	ECT_SURG2	Other - Matcho et al	X							
4084217	Admit gynaecological emergency	ECT_SURG2	Other - Matcho et al	X							
4214930	Refer to early pregnancy unit	PCONF	Other - Matcho et al	X							
40258875	Termination of pregnancy NEC	ECT_SURG2	Other - Matcho et al	X							
4196129	Failed minimum access approach	ECT_SURG2	Other - Matcho et al	X							
4085257	Inhalation anesthesia, machine system, closed, circulation of primary agent	ECT_SURG2	Other - Matcho et al	X							
4080059	Admit obstetric emergency	PCOMP	Other - Matcho et al	X							
40259069	Dye test of fallopian tube	ECT_SURG2	Other - Matcho et al	X							
3004303	Choriogonadotropin.intact [Moles/volume] in Serum or Plasma	HCG	Other - Matcho et al	X							
4210722	Serum free beta human chorionic gonadotropin level	HCG	Other - Matcho et al	X							
4195509	Serum free beta human chorionic gonadotropin multiple of median measurement	HCG	Other - Matcho et al	X							
4198892	Serum human chorionic gonadotropin multiple of median measurement	HCG	Other - Matcho et al	X							
4214677	Total beta human chorionic gonadotrophin level	HCG	Other - Matcho et al	X							
4285271	Human chorionic gonadotropin measurement	HCG	Other - Matcho et al	X							
4210718	Plasma human chorionic gonadotropin multiple of median measurement	HCG	Other - Matcho et al	X							
2004762	Medical induction of labor	LDEL	Other - Matcho et al	X							
138813	Early or threatened labour	LDEL	Other - Matcho et al	X							
2004766	Episiotomy	LDEL	Other - Matcho et al	X							
2004745	Induction of labor by artificial rupture of membranes	LDEL	Other - Matcho et al	X							
2004729	Vacuum extraction with episiotomy	LDEL	Other - Matcho et al	X							
444082	False labor without delivery	LDEL	Other - Matcho et al	X							
2004703	Low forceps operation	LDEL	Other - Matcho et al	X							
2004704	Low forceps operation with episiotomy	LDEL	Other - Matcho et al	X							
432382	Complication of labor AND/OR delivery	LDEL	Other - Matcho et al	X							
442613	Failed forceps	LDEL	Other - Matcho et al	X							
197346	Secondary uterine inertia with antenatal problem	LDEL	Other - Matcho et al	X							
2004785	Classical cesarean section	LDEL	Other - Matcho et al	X							
196759	Primary uterine inertia with antenatal problem	LDEL	Other - Matcho et al	X							

2004769	External version to assist delivery	LDEL	Other - Matcho et al	X						
2004711	Forceps rotation of fetal head	LDEL	Other - Matcho et al	X						
193831	Obstructed labour due to foetal malposition with antenatal problem	LDEL	Other - Matcho et al	X						
2004750	Internal and combined version with extraction	LDEL	Other - Matcho et al	X						
437620	Prolonged second stage with antenatal problem	LDEL	Other - Matcho et al	X						
2004706	Mid forceps operation with episiotomy	LDEL	Other - Matcho et al	X						
193273	Obstructed labor caused by bony pelvis with antenatal problem	LDEL	Other - Matcho et al	X						
2004749	Internal and combined version without extraction	LDEL	Other - Matcho et al	X						
4122728	Length of labour	LDEL	Other - Matcho et al	X						
4014719	Labour details	LDEL	Other - Matcho et al	X						
4125778	Premature/false labour	LDEL	Other - Matcho et al	X						
437614	Early onset of delivery	LDEL	Other - Matcho et al	X						
4016476	Placenta incomplete	LDEL	Other - Matcho et al	X						
4014453	2nd stage of labor length	LDEL	Other - Matcho et al	X						
4073432	SIL - Surgical induction of labor	LDEL	Other - Matcho et al	X						
4015420	Duration of first stage of labor	LDEL	Other - Matcho et al	X						
4145316	Vaginal show	LDEL	Other - Matcho et al	X						
4107728	Intravenous induction of labor	LDEL	Other - Matcho et al	X						
4015161	Duration of third stage of labour	LDEL	Other - Matcho et al	X						
4075176	External version of breech	LDEL	Other - Matcho et al	X						
4070222	Physiological delivery of placenta	LDEL	Other - Matcho et al	X						
4055924	Entire placenta	LDEL	Other - Matcho et al	X						
1305058	Methotrexate	MTX	Other - Matcho et al	X						
1305058	Methotrexate	MTX	Other - Matcho et al	X						
43125784	methotrexate	MTX	Other - Matcho et al	X						
1305058	Methotrexate	MTX	Other - Matcho et al	X						
1305058	Methotrexate	MTX	Other - Matcho et al	X						
4186424	Nuchal ultrasound scan	NULS	Other - Matcho et al	X						
4060266	Antenatal RhD antibody screening	OTEST	Other - Matcho et al	X						
2212145	Chorionic gonadotropin stimulation panel; estradiol response This panel must include the following: Estradiol (82670 x 2 on three pooled blood samples)	OTEST	Other - Matcho et al	X						
2212436	Fetal lung maturity assessment; lecithin sphingomyelin (L/S) ratio	OTEST	Other - Matcho et al	X						
2212438	Fetal lung maturity assessment; fluorescence polarization	OTEST	Other - Matcho et al	X						
2721184	PLACENTAL ALPHA MICROGLOBULIN-1 RAPID IMMUNOASSAY FOR DETECTION OF RUPTURE OF FETAL MEMBRANES	OTEST	Other - Matcho et al	X						
4207466	Antenatal blood tests	OTEST	Other - Matcho et al	X						
4191703	Antenatal HIV screening	OTEST	Other - Matcho et al	X						
4014319	Antenatal blood group screening	OTEST	Other - Matcho et al	X						
4149370	Down screening - blood test	OTEST	Other - Matcho et al	X						
4306192	Cardiotochogram	OTEST	Other - Matcho et al	X						
4015294	Antenatal syphilis screening	OTEST	Other - Matcho et al	X						
4210721	Plasma free beta human chorionic gonadotropin level	OTEST	Other - Matcho et al	X						
4059050	Abdominal obstetric X-ray	OTEST	Other - Matcho et al	X						
4015139	Antenatal blood group screening done	OTEST	Other - Matcho et al	X						
4015137	A/N syphilis screen-blood sent	OTEST	Other - Matcho et al	X						
4120817	Sampling of chorionic villus	OTEST	Other - Matcho et al	X						
4262288	Fetal RBC determination	OTEST	Other - Matcho et al	X						
4098631	Unconjugated oestriol measurement	OTEST	Other - Matcho et al	X						
40525252	[V]Other specified antenatal screening	OTEST	Other - Matcho et al	X						
4232703	Measurement of pregnancy associated plasma protein A concentration	OTEST	Other - Matcho et al	X						
4151412	Fetoplacental hormone measurement	OTEST	Other - Matcho et al	X						
4073428	Percutaneous sampling of chorionic villus	OTEST	Other - Matcho et al	X						
4059051	Obstetric X-ray - fetus	OTEST	Other - Matcho et al	X						
4014290	Triple test not wanted	OTEST	Other - Matcho et al	X						
2004542	Artificial insemination	OVUL	Other - Matcho et al	X						
42739500	Assisted oocyte fertilization, microtechnique (any method)	OVUL	Other - Matcho et al	X						
4073406	Transvaginal oocyte recovery	OVUL	Other - Matcho et al	X						
4022096	Direct injection of sperm into cytoplasm of the oocyte	OVUL	Other - Matcho et al	X						
4127104	GIFT - Gamete intrafallopian transfer	OVUL	Other - Matcho et al	X						
4072715	Intracervical artificial insemination	OVUL	Other - Matcho et al	X						
40259107	Endoscopic oocyte recovery NEC	OVUL	Other - Matcho et al	X						
4138633	In vitro fertilisation with intra-cytoplasmic sperm injection	OVUL	Other - Matcho et al	X						
4138627	In vitro fertilization using donor eggs	OVUL	Other - Matcho et al	X						
4145843	Intrauterine insemination with superovulation using partner sperm	OVUL	Other - Matcho et al	X						
4072852	Endoscopic intrafallopian transfer of gamete	OVUL	Other - Matcho et al	X						
4072421	Gamete intrauterine transfer	OVUL	Other - Matcho et al	X						
4141534	In vitro fertilisation using donor and egg intra-cytoplasmic sperm injection	OVUL	Other - Matcho et al	X						
4137390	Intrauterine insemination with superovulation using donor sperm	OVUL	Other - Matcho et al	X						
4075024	Other specified oocyte recovery	OVUL	Other - Matcho et al	X						
4072863	Endoscopic transurethral ultrasound directed oocyte recovery	Not categorized	Other - Matcho et al	X						
4142402	IVF - In vitro fertilisation with pre-implantation genetic diagnosis	OVUL	Other - Matcho et al	X						
4074569	Endoscopic transvesical oocyte recovery	OVUL	Other - Matcho et al	X						
40490447	Transmyometrial transfer of embryo to uterus	OVUL2	Other - Matcho et al	X						
1515417	Urofollitropin	OVULDR	Other - Matcho et al	X						
1542948	Follitropin Alfa	OVULDR	Other - Matcho et al	X						
1543112	Lutropin alfa	OVULDR	Other - Matcho et al	X						
1597235	Follicle Stimulating Hormone, beta Subunit	OVULDR	Other - Matcho et al	X						
433822	Complication related to pregnancy	PCOMP	Other - Matcho et al	X						
4063297	Fetus with damage due to intrauterine contraceptive device with antenatal problem	PCOMP	Other - Matcho et al	X						
443295	Maternal care for diminished foetal movements	PCOMP	Other - Matcho et al	X						
437935	Hemorrhage in early pregnancy, antepartum	PCOMP	Other - Matcho et al	X						
77335	Known OR suspected fetal abnormality affecting management of mother	PCOMP	Other - Matcho et al	X						
4197403	Intervillous thrombosis	PCOMP	Other - Matcho et al	X						
442827	Fetus with central nervous system malformation with antenatal problem	PCOMP	Other - Matcho et al	X						
443325	Late pregnancy vomiting - not delivered	PCOMP	Other - Matcho et al	X						

438209	Pregnancy care of habitual aborter	PCOMP	Other - Matcho et al	X						
441680	Fetus OR newborn affected by multiple pregnancy	PCOMP	Other - Matcho et al	X						
2004824	Amnioinfusion	PCOMP	Other - Matcho et al	X						
4064277	Fetus with damage due to intrauterine contraceptive device	PCOMP	Other - Matcho et al	X						
76750	Generally contracted pelvis with antenatal problem	PCOMP	Other - Matcho et al	X						
78817	Mixed feto-pelvic disproportion with antenatal problem	PCOMP	Other - Matcho et al	X						
2004764	Manual rotation of fetal head	PCOMP	Other - Matcho et al	X						
4146816	Pre-existing hypertension complicating AND/OR reason for care during childbirth	PCOMP	Other - Matcho et al	X						
438204	Edema or excessive weight gain in pregnancy without mention of hypertension	PCOMP	Other - Matcho et al	X						
201920	Congenital OR acquired abnormality of vagina affecting pregnancy	PCOMP	Other - Matcho et al	X						
196175	Congenital OR acquired abnormality of cervix affecting pregnancy	PCOMP	Other - Matcho et al	X						
438203	Antepartum hemorrhage with coagulation defect - not delivered	PCOMP	Other - Matcho et al	X						
432977	Maternal distress with antenatal problem	PCOMP	Other - Matcho et al	X						
73265	Deep transverse arrest with antenatal problem	PCOMP	Other - Matcho et al	X						
201516	Primary malignant neoplasm of placenta	PCOMP	Other - Matcho et al	X						
440162	Congenital or acquired abnormality of vulva complicating antenatal care - baby not yet delivered	PCOMP	Other - Matcho et al	X						
72378	Outlet pelvic contraction with antenatal problem	PCOMP	Other - Matcho et al	X						
439880	Late vomiting of pregnancy	PCOMP	Other - Matcho et al	X						
4059763	Disproportion - major pelvic abnormality with antenatal problem	PCOMP	Other - Matcho et al	X						
442421	Cord tangled with compression with antenatal problem	PCOMP	Other - Matcho et al	X						
79896	Inlet pelvic contraction with antenatal problem	PCOMP	Other - Matcho et al	X						
4143204	Foetus with viral damage via mother	PCOMP	Other - Matcho et al	X						
444245	Hydrocephalic fetus causing disproportion	PCOMP	Other - Matcho et al	X						
2004823	Correction of fetal defect	PCOMP	Other - Matcho et al	X						
4143074	Antepartum hemorrhage, abruptio placentae and placenta previa	PCOMP	Other - Matcho et al	X						
4038755	O/E - fundus 34-36 week size	PCOMP	Other - Matcho et al	X						
439348	Pregnancy complications	PCOMP	Other - Matcho et al	X						
4145315	Fetal movements felt	PCOMP	Other - Matcho et al	X						
4038426	O/E - fetal movements felt	PCOMP	Other - Matcho et al	X						
4080059	Admit obstetric emergency	PCOMP	Other - Matcho et al	X						
4080067	Non-urgent obstetric admission	PCOMP	Other - Matcho et al	X						
4079751	Iron supplement in pregnancy	PCOMP	Other - Matcho et al	X						
4127241	Shirodkar cervical cerclage	PCOMP	Other - Matcho et al	X						
4129039	Anti-D antibodies	PCOMP	Other - Matcho et al	X						
4120986	Controlled cord traction of placenta	PCOMP	Other - Matcho et al	X						
43530880	Known or suspected fetal abnormality	PCOMP	Other - Matcho et al	X						
4064854	Placental localization	PCOMP	Other - Matcho et al	X						
4062674	Pregnancy complication NOS	PCOMP	Other - Matcho et al	X						
4130310	Macdonald cervical cerclage	PCOMP	Other - Matcho et al	X						
4034308	Acromion presentation	PCOMP	Other - Matcho et al	X						
40479820	Exposure to rubella in pregnancy	PCOMP	Other - Matcho et al	X						
40519557	[V]Other unwanted pregnancy	PCONF	Other - Matcho et al	X						
40524802	[V]Pregnant state, incidental	PCONF	Other - Matcho et al	X						
40524805	[V]Unspecified pregnant state	PCONF	Other - Matcho et al	X						
43054893	Delivery location	PCONF	Other - Matcho et al	X						
3006722	Fetal Heart Activity US	PCONF	Other - Matcho et al	X						
40759953	Fetal Movement	PCONF	Other - Matcho et al	X						
3000119	Fetal presentation US	PCONF	Other - Matcho et al	X						
4061522	General practitioner unit delivery booking	PCONF	Other - Matcho et al	X						
4210896	Length of gestation	PCONF	Other - Matcho et al	X						
4038942	O/E - fetal movements	PCONF	Other - Matcho et al	X						
4150948	On examination - fetal presentation	PCONF	Other - Matcho et al	X						
4148890	Pregnancy benefits	PCONF	Other - Matcho et al	X						
4061423	Pregnant - ? planned	PCONF	Other - Matcho et al	X						
4061411	Pregnant, sheath failure	PCONF	Other - Matcho et al	X						
4214930	Refer to early pregnancy unit	PCONF	Other - Matcho et al	X						
3040000	Reliability of last menstrual period observation	PCONF	Other - Matcho et al	X						
4015590	Counselling for termination of pregnancy	PCONF	Other - Matcho et al	X						
42739011	History and examination of the normal newborn infant, initiation of diagnostic and treatment programs and preparation of hospital records. (This code should also be used for birthing room deliveries.)	POST	Other - Matcho et al	X						
4253751	Child examination - 2 weeks	POST	Other - Matcho et al	X						
439135	Fetus OR newborn affected by cesarean delivery	POST	Other - Matcho et al	X						
2004843	Repair of current obstetric laceration of rectum and sphincter ani	POST	Other - Matcho et al	X						
137371	Neonatal septicaemia	POST	Other - Matcho et al	X						
2004809	Fetal EKG (scalp)	POST	Other - Matcho et al	X						
442917	Mental disorder in the puerperium - baby delivered during previous episode of care	POST	Other - Matcho et al	X						
42739633	Initial neonatal intensive care, per day, for the evaluation and management of a critically ill neonate or infant This code is reserved for the date of admission for neonates who are critically ill. Critically ill neonates require cardiac and/or respirator	POST	Other - Matcho et al	X						
42739550	Subsequent neonatal intensive care, per day, for the evaluation and management of a critically ill and unstable neonate or infant A critically ill and unstable neonate will require cardiac and/or respiratory support (including ventilator or nasal CPAP when	POST	Other - Matcho et al	X						
4066255	Breast engorgement in pregnancy/puerperium/lact + p/n comp	POST	Other - Matcho et al	X						
2004826	Manual removal of retained placenta	POST	Other - Matcho et al	X						
437975	Fetus OR newborn affected by delivery by vacuum extractor	POST	Other - Matcho et al	X						
42739015	Newborn resuscitation: provision of positive pressure ventilation and/or chest compressions in the presence of acute inadequate ventilation and/or cardiac output	POST	Other - Matcho et al	X						
2004845	Manual exploration of uterine cavity, postpartum	POST	Other - Matcho et al	X						
434483	Fetus OR newborn affected by abnormal uterine contraction	POST	Other - Matcho et al	X						
42739012	Normal newborn care in other than hospital or birthing room setting, including physical examination of baby and conference(s) with parent(s)	POST	Other - Matcho et al	X						

78563	Fetus OR newborn affected by malpresentation before labor	POST	Other - Matcho et al	X						
2004842	Repair of current obstetric laceration of bladder and urethra	POST	Other - Matcho et al	X						
433319	Fetus OR newborn affected by maternal complication of pregnancy	POST	Other - Matcho et al	X						
436518	Fetus OR newborn affected by chorioamnionitis	POST	Other - Matcho et al	X						
200469	Third-stage postpartum hemorrhage - delivered with postnatal problem	POST	Other - Matcho et al	X						
436804	Fetus OR newborn affected by premature rupture of membranes	POST	Other - Matcho et al	X						
437088	Fetus OR newborn affected by oligohydramnios	POST	Other - Matcho et al	X						
438868	Fetus OR newborn affected by maternal injury	POST	Other - Matcho et al	X						
438814	Delayed AND/OR excessive hemorrhage following molar AND/OR ectopic pregnancy	POST	Other - Matcho et al	X						
197970	Persistent fetal circulation	POST	Other - Matcho et al	X						
2004546	Removal of cerclage material from cervix	POST	Other - Matcho et al	X						
433873	Hypocalcemia AND/OR hypomagnesemia of newborn	POST	Other - Matcho et al	X						
439931	Perinatal hemolytic jaundice	POST	Other - Matcho et al	X						
4060183	Third-stage postpartum haemorrhage with postnatal problem	POST	Other - Matcho et al	X						
435641	Birth trauma	POST	Other - Matcho et al	X						
378544	Subdural and cerebral hemorrhage due to birth trauma	POST	Other - Matcho et al	X						
2004829	Repair of current obstetric laceration of cervix	POST	Other - Matcho et al	X						
435063	Fetus OR newborn affected by ectopic pregnancy	POST	Other - Matcho et al	X						
435918	Foetus OR newborn affected by forceps delivery	POST	Other - Matcho et al	X						
438871	Drug withdrawal syndrome in newborn	POST	Other - Matcho et al	X						
201410	Fetus OR newborn affected by placenta previa	POST	Other - Matcho et al	X						
433263	Complications following abortion and ectopic and molar pregnancies	POST	Other - Matcho et al	X						
442216	Kernicterus not due to isoimmunization	POST	Other - Matcho et al	X						
435062	Fetus OR newborn affected by maternal renal AND/OR urinary tract disease	POST	Other - Matcho et al	X						
439924	Endocrine AND/OR metabolic disorder specific to the fetus OR newborn	POST	Other - Matcho et al	X						
437668	Fetus OR newborn affected by polyhydramnios	POST	Other - Matcho et al	X						
441122	Fetus OR newborn affected by placental transfusion syndrome	POST	Other - Matcho et al	X						
257370	Skeletal injury due to birth trauma	POST	Other - Matcho et al	X						
433310	Fetus OR newborn affected by precipitate delivery	POST	Other - Matcho et al	X						
4151903	Pre-existing hypertension complicating AND/OR reason for care during puerperium	POST	Other - Matcho et al	X						
377680	Facial nerve injury as birth trauma	POST	Other - Matcho et al	X						
439923	Fetal and newborn blood disorders	POST	Other - Matcho et al	X						
77679	Spine or spinal cord injury due to birth trauma	POST	Other - Matcho et al	X						
2004828	Repair of current obstetric laceration of uterus, not otherwise specified	POST	Other - Matcho et al	X						
441684	Fetus OR newborn affected by surgical operation on mother	POST	Other - Matcho et al	X						
442058	Superficial thrombophlebitis in pregnancy and the puerperium	POST	Other - Matcho et al	X						
196490	Postpartum acute renal failure	POST	Other - Matcho et al	X						
133028	Scalp injuries due to birth trauma	POST	Other - Matcho et al	X						
317669	Cardiac arrest in fetus OR newborn	POST	Other - Matcho et al	X						
434745	Fetus OR newborn affected by maternal nutritional disorder	POST	Other - Matcho et al	X						
436220	Fetus OR newborn affected by chronic maternal respiratory disease	POST	Other - Matcho et al	X						
2004830	Repair of current obstetric laceration of corpus uteri	POST	Other - Matcho et al	X						
442529	Cranial nerve injury due to birth trauma	POST	Other - Matcho et al	X						
438214	Vulval and/or perineal haematoma during delivery with postnatal problem	POST	Other - Matcho et al	X						
433548	Cracked nipple associated with childbirth	POST	Other - Matcho et al	X						
4318553	Respiratory tract haemorrhage of the newborn	POST	Other - Matcho et al	X						
432429	Fetus OR newborn affected by anti-infective agent transmitted via placenta AND/OR breast milk	POST	Other - Matcho et al	X						
196764	Post-delivery acute renal failure - delivered with postnatal problem	POST	Other - Matcho et al	X						
433314	Transitory neonatal endocrine AND/OR metabolic disorder	POST	Other - Matcho et al	X						
4028640	Bicornuate uterus in pregnancy, childbirth and the puerperium	POST	Other - Matcho et al	X						
40767416	Was your pregnancy a live birth, stillbirth, miscarriage, abortion, or ectopic pregnancy PhenX	SA	Other - Matcho et al	X						
40767416	Was your pregnancy a live birth, stillbirth, miscarriage, abortion, or ectopic pregnancy PhenX	SA	Other - Matcho et al	X						
4297233	Maternal postnatal 6 week examination	POST	Other - Matcho et al	X						
3003857	Fetal Gender US	POST	Other - Matcho et al	X						
4170305	Antenatal/postnatal care	POST	Other - Matcho et al	X						
3004221	5 minute Apgar Score	POST	Other - Matcho et al	X						
4014439	Infant feeding method	POST	Other - Matcho et al	X						
4015154	Postnatal examination normal	POST	Other - Matcho et al	X						
4150640	Snuffles in newborn	POST	Other - Matcho et al	X						
4016464	Apgar at 5 minutes	POST	Other - Matcho et al	X						
40485049	Maternal postnatal examination done	POST	Other - Matcho et al	X						
4014304	Apgar at 1 minute	POST	Other - Matcho et al	X						
4187201	Perinatal disorder	POST	Other - Matcho et al	X						
40524819	[V]Postnatal care and examination	POST	Other - Matcho et al	X						
4014445	Postnatal examination minor problem found	POST	Other - Matcho et al	X						
4014280	Postnatal - first day visit	POST	Other - Matcho et al	X						
4014281	Postnatal - fifth day visit	POST	Other - Matcho et al	X						
4014442	Postnatal - second day visit	POST	Other - Matcho et al	X						
4014443	Postnatal - fourth day visit	POST	Other - Matcho et al	X						
4015150	Postnatal - third day visit	POST	Other - Matcho et al	X						
4151029	Postnatal visit status	POST	Other - Matcho et al	X						
4089029	Seen in postnatal clinic	POST	Other - Matcho et al	X						
40485007	Maternal postnatal examination offered	POST	Other - Matcho et al	X						
4015410	Postnatal care provider	POST	Other - Matcho et al	X						
4062356	Infant feeding education	POST	Other - Matcho et al	X						
4296686	Full postnatal examination	POST	Other - Matcho et al	X						
4015412	Postnatal - seventh day visit	POST	Other - Matcho et al	X						
4014282	Postnatal - sixth day visit	POST	Other - Matcho et al	X						

4014156	P/N care from G.P.	POST	Other - Matcho et al	X						
40483251	Maternal postnatal examination not attended	POST	Other - Matcho et al	X						
4015152	Postnatal care less than 48 hours after birth	POST	Other - Matcho et al	X						
4014446	Postnatal data	POST	Other - Matcho et al	X						
4167905	Apgar normal	POST	Other - Matcho et al	X						
4015151	Postnatal - tenth day visit	POST	Other - Matcho et al	X						
4015413	Postnatal - eighth day visit	POST	Other - Matcho et al	X						
4015415	Postnatal care greater than 48 hours after birth	POST	Other - Matcho et al	X						
4215564	Postnatal depression counselling	POST	Other - Matcho et al	X						
4071642	Repair of episiotomy	POST	Other - Matcho et al	X						
4015414	Postnatal - ninth day visit	POST	Other - Matcho et al	X						
4062362	Postnatal support group	POST	Other - Matcho et al	X						
4118417	Anomalies of umbilicus	POST	Other - Matcho et al	X						
4014465	Birth length	POST	Other - Matcho et al	X						
40479783	Child examination at birth with explicit context	POST	Other - Matcho et al	X						
4016466	Apgar at 5 minutes = 9	POST	Other - Matcho et al	X						
4069972	Immediate repair of obstetric laceration	POST	Other - Matcho et al	X						
40514773	[X]Infection of caesarean section wound following delivery	POST	Other - Matcho et al	X						
4015144	Breastfeeding started	POST	Other - Matcho et al	X						
4034146	Postnatal vaginal discomfort	POST	Other - Matcho et al	X						
4260749	Breast fed at birth	POST	Other - Matcho et al	X						
4203918	Teach postpartum care	POST	Other - Matcho et al	X						
441925	Obstetric nipple infection	POST	Other - Matcho et al	X						
4069968	Manual removal of products of conception from delivered uterus	POST	Other - Matcho et al	X						
4073719	On maternity leave	POST	Other - Matcho et al	X						
4260748	Bottle fed at birth	POST	Other - Matcho et al	X						
4016060	Difficult to establish feeding	POST	Other - Matcho et al	X						
4084845	Discharged from hospital within 6 hours of delivery	POST	Other - Matcho et al	X						
40388780	[X]Mental and behavioral disorders associated with the puerperium, not elsewhere classified	POST	Other - Matcho et al	X						
40356741	[X]Mild mental and behavioural disorders associated with the puerperium, not elsewhere classified	POST	Other - Matcho et al	X						
40394902	[X]Vaginitis following delivery	POST	Other - Matcho et al	X						
4016467	Apgar at 5 minutes = 10	POST	Other - Matcho et al	X						
4174400	Urticaria neonatorum	POST	Other - Matcho et al	X						
4174302	Neonatal infection of the eye	POST	Other - Matcho et al	X						
4287783	Perinatal necrotising enterocolitis	POST	Other - Matcho et al	X						
40484589	Maternal postnatal examination refused	POST	Other - Matcho et al	X						
4081774	Obstetric self-referral	POST	Other - Matcho et al	X						
4072420	Dilation of cervix uteri and curettage of uterus for removal of mole	TRO	Other - Matcho et al	X						
4143115	Antenatal ultrasound scan for possible abnormality	ULS	Other - Matcho et al	X						
4150653	US scan - fetal abnormality	ULS	Other - Matcho et al	X						
4082672	Ultrasound scan for fetal growth	ULS	Other - Matcho et al	X						
2006934	Diagnostic ultrasound of gravid uterus	ULS	Other - Matcho et al	X						
4061802	Antenatal ultrasound scan status	ULS	Other - Matcho et al	X						
4060625	Dating/booking US scan	ULS	Other - Matcho et al	X						
4061131	Antenatal ultrasound result received	ULS	Other - Matcho et al	X						
4060626	Viability US scan	ULS	Other - Matcho et al	X						
4060265	Antenatal ultrasound scan at 9-16 weeks	ULS	Other - Matcho et al	X						
4061129	US obstetric scan requested	ULS	Other - Matcho et al	X						
4082534	Ultrasound scan of fetus	ULS	Other - Matcho et al	X						
4061534	Antenatal ultrasound scan at 17-22 weeks	ULS	Other - Matcho et al	X						
4141415	Antenatal ultrasound scan at 22-40 weeks	ULS	Other - Matcho et al	X						
4061803	Antenatal ultrasound scan 4-8 weeks	ULS	Other - Matcho et al	X						
4060264	A/N U/S scan normal += dates	ULS	Other - Matcho et al	X						
4060263	A/N U/S scan awaited	ULS	Other - Matcho et al	X						
4152443	Antenatal scan unable to confirm pregnancy	ULS	Other - Matcho et al	X						
4060262	A/N U/S scan wanted	ULS	Other - Matcho et al	X						
4061531	A/N U/S scan offered	ULS	Other - Matcho et al	X						
4060624	Presentation US scan	ULS	Other - Matcho et al	X						
4055287	High sensitivity urine pregnancy test	UP	Other - Matcho et al	X						
4015274	Baby very premature 32-36 weeks	PREM	Preterm pregnancy	X						
4150404	Baby premature 39 weeks	PREM	Preterm pregnancy	X						
4015275	Baby extremely premature 28-32 weeks	PREM	Preterm pregnancy	X						
4150405	Baby premature 38 weeks	PREM	Preterm pregnancy	X						
4033096	Miscarriage with urinary tract infection		Spontaneous abortion (SA)			X		X		
4041728	Miscarriage with laceration of bowel		Spontaneous abortion (SA)					X		
4047775	Miscarriage with cerebral anoxia		Spontaneous abortion (SA)					X		
4049891	Miscarriage with perforation of uterus		Spontaneous abortion (SA)					X		
4051244	Miscarriage due to Leptospira		Spontaneous abortion (SA)					X		
4064536	Miscarriage with afibrinogenemia		Spontaneous abortion (SA)					X		
4067106	Miscarriage	SA	Spontaneous abortion (SA)	X	X	X	X	X		
4071638	Instrumental removal of products of conception from delivered uterus	SA	Spontaneous abortion (SA)	X						
4074297	Dilation of cervix uteri and vacuum aspiration of products of conception from uterus	SA	Spontaneous abortion (SA)	X						
4074867	Dilation of cervix uteri and curettage of uterus for removal of missed miscarriage	SA	Spontaneous abortion (SA)	X				X		
4078393	Miscarriage in first trimester		Spontaneous abortion (SA)					X		
4085627	Miscarriage with septic shock		Spontaneous abortion (SA)					X		
4087135	Surgical treatment of missed miscarriage of first trimester		Spontaneous abortion (SA)					X		
4112954	Unspecified spontaneous abortion without mention of complication	SA	Spontaneous abortion (SA)	X						
4113036	Unspecified legal abortion with no mention of complication	SA	Spontaneous abortion (SA)	X						
4113352	Complete spontaneous abortion NOS	SA	Spontaneous abortion (SA)	X						
4113983	Unspecified abortion incomplete	SA	Spontaneous abortion (SA)	X						
4113986	Unspecified abortion complete	SA	Spontaneous abortion (SA)	X						
4113994	Endometritis following abortive pregnancy	SA	Spontaneous abortion (SA)	X						
4114265	Delayed or excessive haemorrhage following abortive pregnancy	SA	Spontaneous abortion (SA)	X						
4114280	Readmission for retained products of conception, spontaneous abortion	SA	Spontaneous abortion (SA)	X				X		
4130670	Miscarriage with laceration of cervix		Spontaneous abortion (SA)					X		
4138740	Dilation of cervix uteri and curettage of products of conception from uterus	SA	Spontaneous abortion (SA)	X						
4146772	Inevitable miscarriage complete	SA	Spontaneous abortion (SA)	X				X		

4146773	Inevitable miscarriage incomplete	SA	Spontaneous abortion (SA)	X				X		
4146774	Complete inevitable miscarriage without complication		Spontaneous abortion (SA)	X				X		
442338	Foetal death from asphyxia AND/OR anoxia before onset of labour	SB	Stillbirth (SB)	X						
443695	Foetal death from asphyxia AND/OR anoxia during labour	SB	Stillbirth (SB)	X						
40767416	Was your pregnancy a live birth, stillbirth, miscarriage, abortion, or ectopic pregnancy PhenX	SA	Stillbirth (SB)	X						
40767416	Was your pregnancy a live birth, stillbirth, miscarriage, abortion, or ectopic pregnancy PhenX	SA	Stillbirth (SB)	X						
40406337	Stillbirth	SB	Stillbirth (SB)	X						
4071603	Foetal death due to prelabour anoxia	SB	Stillbirth (SB)	X						
2004426	Hysterotomy	Not categorized	Other - Matcho et al	X						
4062357	Pregnancy smoking education	Not categorized	Other - Matcho et al	X						
4061800	Intends to bottle feed	Not categorized	Other - Matcho et al	X						
4052314	Maternity allowance	Not categorized	Other - Matcho et al	X						
4015293	Rubella screening - blood sent	Not categorized	Other - Matcho et al	X						
4063126	Pregnancy diet education	Not categorized	Other - Matcho et al	X						
4063127	Pregnancy alcohol education	Not categorized	Other - Matcho et al	X						
4047842	Pregnancy review	Not categorized	Other - Matcho et al	X						
40517217	[V]Pregnancy not (yet) confirmed	Not categorized	Other - Matcho et al	X						
4064792	Drugs in pregnancy education	Not categorized	Other - Matcho et al	X						
4062360	Maternity milk and vitamins education	Not categorized	Other - Matcho et al	X						
4041877	Urine pregnancy test equivocal	Not categorized	Other - Matcho et al	X						
4061152	Feeding intention - unsure	Not categorized	Other - Matcho et al	X						
4064791	Pregnancy exercise education	Not categorized	Other - Matcho et al	X						
4061538	Rubella screening offered	Not categorized	Other - Matcho et al	X						
4059657	Maternity grant	Not categorized	Other - Matcho et al	X						
442055	Obstetric air pulmonary embolism	Not categorized	Other - Matcho et al	X						
442921	Drug dependence in the puerperium - baby delivered during previous episode of care	Not categorized	Other - Matcho et al	X						
4071609	Other fetal and newborn respiratory conditions	Not categorized	Other - Matcho et al	X						
2004851	Immediate postpartum manual replacement of inverted uterus	Not categorized	Other - Matcho et al	X						
201356	Congenital OR acquired abnormality of vulva affecting pregnancy	Not categorized	Other - Matcho et al	X						
2004783	Other operations to assist delivery	Not categorized	Other - Matcho et al	X						
3003667	Glucose [Presence] in Urine by Test strip --1.5 hours post 75 g glucose PO	Not categorized	Other - Matcho et al	X						
435322	Maternal tuberculosis during pregnancy - baby not yet delivered	Not categorized	Other - Matcho et al	X						
3033544	Somatotropin [Mass/volume] in Serum or Plasma --pre 1 g/kg glucose PO	Not categorized	Other - Matcho et al	X						
2004787	Extraperitoneal cesarean section	Not categorized	Other - Matcho et al	X						
3002934	Glucose [Presence] in Urine by Test strip --4 hours post 75 g glucose PO	Not categorized	Other - Matcho et al	X						
198215	Renal hypertension complicating pregnancy, childbirth and the puerperium with postnatal complication	Not categorized	Other - Matcho et al	X						
2004804	Intra-amniotic injection for abortion	Not categorized	Other - Matcho et al	X						
2004808	Amnioscopy	Not categorized	Other - Matcho et al	X						
3011305	Lecithin/Sphingomyelin [Mass ratio] in Amniotic fluid	Not categorized	Other - Matcho et al	X						
200151	Peripheral neuritis in pregnancy - delivered with postnatal complication	Not categorized	Other - Matcho et al	X						
2004848	Evacuation of obstetrical incisional hematoma of perineum	Not categorized	Other - Matcho et al	X						
3002376	Glucose [Presence] in Urine by Test strip --5 hours post 75 g glucose PO	Not categorized	Other - Matcho et al	X						
442549	Retracted nipple associated with childbirth	Not categorized	Other - Matcho et al	X						
3016427	Glucose [Mass/volume] in Peritoneal dialysis fluid --5th specimen	Not categorized	Other - Matcho et al	X						
3038388	Glucose [Mass/volume] in Serum or Plasma --3rd specimen post dose lactose	Not categorized	Other - Matcho et al	X						
3025658	Glucose [Mass/volume] in Serum or Plasma --6th specimen post dose lactose	Not categorized	Other - Matcho et al	X						
3041186	Glucose [Mass/volume] in Serum or Plasma --2nd specimen post dose lactose	Not categorized	Other - Matcho et al	X						
4060421	Other medical condition during pregnancy, childbirth and the puerperium	Not categorized	Other - Matcho et al	X						
3041884	Glucose [Mass/volume] in Serum or Plasma --1st specimen post dose lactose	Not categorized	Other - Matcho et al	X						
2721183	MATERNAL SERUM QUADRUPLE MARKER SCREEN INCLUDING ALPHA-FETOPROTEIN (AFP), ESTRIOL, HUMAN CHORIONIC GONADOTROPIN (HCG) AND INHIBIN A	Not categorized	Other - Matcho et al	X						
434102	Abortion complicated by metabolic disorder	Not categorized	Other - Matcho et al	X						
3003773	Glucose [Mass/volume] in Urine --6 hours post 100 g glucose PO	Not categorized	Other - Matcho et al	X						
2004727	Forceps application to aftercoming head	Not categorized	Other - Matcho et al	X						
2004806	Intrauterine transfusion	Not categorized	Other - Matcho et al	X						
80159	Prolapsed arm with antenatal problem	Not categorized	Other - Matcho et al	X						
2004709	High forceps operation with episiotomy	Not categorized	Other - Matcho et al	X						
4129551	Fetus with suspected rubella damage via mother	Not categorized	Other - Matcho et al	X						
319090	Congenital cardiovascular disorder in the puerperium - baby delivered during previous episode of care	Not categorized	Other - Matcho et al	X						
2004791	Hysterotomy to terminate pregnancy	Not categorized	Other - Matcho et al	X						
4047714	Foetus or neonate affected by other complication of labour or delivery	Not categorized	Other - Matcho et al	X						
440477	Obstetric pyaemic and septic pulmonary embolism	Not categorized	Other - Matcho et al	X						
199887	Illegal abortion complicated by delayed AND/OR excessive haemorrhage	Not categorized	Other - Matcho et al	X						
444251	Rubella in mother complicating pregnancy, childbirth AND/OR puerperium	Not categorized	Other - Matcho et al	X						
443017	Maternal tuberculosis in the puerperium - baby delivered during previous episode of care	Not categorized	Other - Matcho et al	X						
2004725	Total breech extraction with forceps to aftercoming head	Not categorized	Other - Matcho et al	X						
195001	Abortion complicated by shock	Not categorized	Other - Matcho et al	X						
4113849	Unspecified spontaneous abortion with other specified complication	Not categorized	Other - Matcho et al	X						
443329	Suppressed lactation with antenatal complication	Not categorized	Other - Matcho et al	X						
2004850	Surgical correction of inverted uterus	Not categorized	Other - Matcho et al	X						
3023191	Choriogonadotropin.alpha subunit [Presence] in Serum or Plasma	Not categorized	Other - Matcho et al	X						

2004723	Partial breech extraction with forceps to aftercoming head	Not categorized	Other - Matcho et al	X						
443052	Maternal gonorrhea in the puerperium - baby delivered during previous episode of care	Not categorized	Other - Matcho et al	X						
73582	Fetus OR newborn affected by maternal death	Not categorized	Other - Matcho et al	X						
194098	Vaginal abnormality - baby delivered with postpartum complication	Not categorized	Other - Matcho et al	X						
2004803	Other obstetric operations	Not categorized	Other - Matcho et al	X						
4071584	Fetal malnutrition without mention of light for dates	Not categorized	Other - Matcho et al	X						
4113048	Unspecified abortion with other specified complication	Not categorized	Other - Matcho et al	X						
437948	Amniotic fluid pulmonary embolism with postnatal complication	Not categorized	Other - Matcho et al	X						
432690	Maternal malaria during pregnancy - baby delivered	Not categorized	Other - Matcho et al	X						
2212437	Fetal lung maturity assessment; foam stability test	Not categorized	Other - Matcho et al	X						
2004770	Replacement of prolapsed umbilical cord	Not categorized	Other - Matcho et al	X						
442050	Obstetric air pulmonary embolism with postnatal complication	Not categorized	Other - Matcho et al	X						
3034206	Rh immune globulin given [Volume]	Not categorized	Other - Matcho et al	X						
434424	Malaria in mother complicating pregnancy, childbirth AND/OR puerperium	Not categorized	Other - Matcho et al	X						
193258	Abortion complicated by embolism	Not categorized	Other - Matcho et al	X						
2004763	Manually assisted delivery	Not categorized	Other - Matcho et al	X						
2101598	Neuraxial analgesia/anesthesia for labor ending in a vaginal delivery (includes any repeat subarachnoid needle placement and drug injection and/or any necessary replacement of an epidural catheter during labor)	Not categorized	Other - Matcho et al	X						
3007097	Phosphatidylglycerol/Surfactant.total in Amniotic fluid	Not categorized	Other - Matcho et al	X						
3017841	Estriol (E3)/Creatinine [Mass ratio] in 24 hour Urine	Not categorized	Other - Matcho et al	X						
3005096	Glucose [Mass/volume] in Serum or Plasma --2.5 hours post 75 g glucose PO	Not categorized	Other - Matcho et al	X						
4191809	Other procedures inducing or assisting delivery	Not categorized	Other - Matcho et al	X						
3016521	Glucose [Mass/volume] in Urine --5 hours post 100 g glucose PO	Not categorized	Other - Matcho et al	X						
2721172	REPAIR OF SACROCOCCYGEAL TERATOMA IN THE FETUS, PROCEDURE PERFORMED IN UTERO	Not categorized	Other - Matcho et al	X						
2004705	Mid forceps operation	Not categorized	Other - Matcho et al	X						
4113971	Unspecified legal abortion with other specified complication	Not categorized	Other - Matcho et al	X						
435874	Maternal rubella in the puerperium - baby delivered during current episode of care	Not categorized	Other - Matcho et al	X						
40765155	Glucose/Insulin [Ratio] in Serum or Plasma	Not categorized	Other - Matcho et al	X						
3046803	Herpes simplex virus 1 DNA [Presence] in Amniotic fluid by Probe and target amplification method	Not categorized	Other - Matcho et al	X						
3046199	Herpes simplex virus 2 DNA [Presence] in Amniotic fluid by Probe and target amplification method	Not categorized	Other - Matcho et al	X						
2721182	MATERNAL SERUM TRIPLE MARKER SCREEN INCLUDING ALPHA-FETOPROTEIN (AFP), ESTRIOL, AND HUMAN CHORIONIC GONADOTROPIN (HCG)	Not categorized	Other - Matcho et al	X						
2101593	Anesthesia for vaginal procedures (including biopsy of labia, vagina, cervix or endometrium); vaginal delivery	Not categorized	Other - Matcho et al	X						
4192036	Cesarean section and removal of fetus	Not categorized	Other - Matcho et al	X						
2004831	Repair of other current obstetric laceration	Not categorized	Other - Matcho et al	X						
2006861	Arteriography of placenta	Not categorized	Other - Matcho et al	X						
2004744	Artificial rupture of membranes	Not categorized	Other - Matcho et al	X						
3015283	Glucose [Mass/volume] in Serum or Plasma --3.5 hours post 75 g glucose PO	Not categorized	Other - Matcho et al	X						
40761515	Herpes simplex virus 1+2 DNA [Presence] in Amniotic fluid by Probe and target amplification method	Not categorized	Other - Matcho et al	X						
2004807	Other intrauterine operations on fetus and amnion	Not categorized	Other - Matcho et al	X						
2004768	Other operations assisting delivery	Not categorized	Other - Matcho et al	X						
3023715	Insulin [Mass/volume] in Serum or Plasma --1 hour post 75 g glucose PO	Not categorized	Other - Matcho et al	X						
3000781	Glucose [Mass/volume] in Serum or Plasma --4.5 hours post 75 g glucose PO	Not categorized	Other - Matcho et al	X						
443013	Maternal rubella in the puerperium - baby delivered during previous episode of care	Not categorized	Other - Matcho et al	X						
3028607	Glucose [Mass/volume] in Serum or Plasma --45 minutes post 50 g lactose PO	Not categorized	Other - Matcho et al	X						
3026604	Glucose [Mass/volume] in Serum or Plasma --15 minutes post 50 g lactose PO	Not categorized	Other - Matcho et al	X						
2721148	INDUCED ABORTION, 25 TO 28 WEEKS	Not categorized	Other - Matcho et al	X						
2213201	Necropsy (autopsy), gross examination only; macerated stillborn	Not categorized	Other - Matcho et al	X						
42742501	Selective head hypothermia, per day, in the neonate 28 days of age or younger	Not categorized	Other - Matcho et al	X						
2007012	Transillumination of newborn skull	Not categorized	Other - Matcho et al	X						
2004782	Pubiotomy to assist delivery	Not categorized	Other - Matcho et al	X						
2004722	Breech extraction	Not categorized	Other - Matcho et al	X						
2004728	Vacuum extraction	Not categorized	Other - Matcho et al	X						
2006756	X-ray of gravid uterus	Not categorized	Other - Matcho et al	X						
2004790	Cesarean section of unspecified type	Not categorized	Other - Matcho et al	X						
3030931	Glucose [Mass/volume] in Serum or Plasma --45 minutes post dose lactose PO	Not categorized	Other - Matcho et al	X						
3035729	Glucose [Moles/volume] in Body fluid	Not categorized	Other - Matcho et al	X						
3000017	Insulin [Mass/volume] in Serum or Plasma --30 minutes post 75 g glucose PO	Not categorized	Other - Matcho et al	X						
3037936	Glucose [Mass/volume] in Urine --30 minutes post dose lactose PO	Not categorized	Other - Matcho et al	X						
3020193	Glucose [Mass/volume] in Serum or Plasma --4 PM specimen	Not categorized	Other - Matcho et al	X						
3036054	Phosphatidylcholine/Albumin [Mass ratio] in Amniotic fluid	Not categorized	Other - Matcho et al	X						
3021580	Glucose [Mass/volume] in Serum or Plasma --7th specimen post XXX challenge	Not categorized	Other - Matcho et al	X						
2721745	OBSTETRICAL TREATMENT/SERVICES, PRENATAL OR POSTPARTUM	Not categorized	Other - Matcho et al	X						
2721149	INDUCED ABORTION, 29 TO 31 WEEKS	Not categorized	Other - Matcho et al	X						
2004708	High forceps operation	Not categorized	Other - Matcho et al	X						
2007465	Placental scan	Not categorized	Other - Matcho et al	X						
4057157	Amniotic fluid AFP normal	Not categorized	Other - Matcho et al	X						
40524864	[V]Twins, both live born	Not categorized	Other - Matcho et al	X						

4197294	Ultrasonic doppler for foetal heart sounds	Not categorized	Other - Matcho et al	X						
4015427	Baby BW = 25%-49% (3150-3449g)	Not categorized	Other - Matcho et al	X						
4146848	Neonatal dacryocystitis or conjunctivitis due to chlamydiae	Not categorized	Other - Matcho et al	X						
4129183	Puerperal phlebitis	Not categorized	Other - Matcho et al	X						
40483081	Antenatal screening declined	Not categorized	Other - Matcho et al	X						
4193440	Notification of birth	Not categorized	Other - Matcho et al	X						
4153441	Fetus or neonate affected by breech delivery and extraction	Not categorized	Other - Matcho et al	X						
4048143	Foetal distress before labour - liveborn	Not categorized	Other - Matcho et al	X						
4210037	Manually assisted spontaneous delivery	Not categorized	Other - Matcho et al	X						
4061428	A/N care: gynecological risk	Not categorized	Other - Matcho et al	X						
4021675	Lactation established	Not categorized	Other - Matcho et al	X						
40525257	[V]Neonatal care in SCBU	Not categorized	Other - Matcho et al	X						
40520511	[V]Pregnancy examination or test, pregnancy unconfirmed	Not categorized	Other - Matcho et al	X						
4061388	O/E - VE - gravid uterus	Not categorized	Other - Matcho et al	X						
4078286	Double test	Not categorized	Other - Matcho et al	X						
4055954	Urine HCG 24 hour assay	Not categorized	Other - Matcho et al	X						
4082067	Referral to postnatal clinic	Not categorized	Other - Matcho et al	X						
4079752	Vitamin supplement - pregnancy	Not categorized	Other - Matcho et al	X						
4065885	Maternal care for fetus	Not categorized	Other - Matcho et al	X						
40524861	[V]Stillbirth	Not categorized	Other - Matcho et al	X						
4082072	Private referral to obstetrician	Not categorized	Other - Matcho et al	X						
4061676	Pregnant, diaphragm failure	Not categorized	Other - Matcho et al	X						
4183563	Human placental lactogen	Not categorized	Other - Matcho et al	X						
4039617	O/E - VE - cervical dilatation	Not categorized	Other - Matcho et al	X						
4015160	Born before arrival	Not categorized	Other - Matcho et al	X						
40358373	[V]Level of neonatal care	Not categorized	Other - Matcho et al	X						
4314269	Echography, scan B-mode for placental localization	Not categorized	Other - Matcho et al	X						
4039604	O/E - oblique lie	Not categorized	Other - Matcho et al	X						
4075179	Prostaglandin induction of labor	Not categorized	Other - Matcho et al	X						
4014320	Antenatal sickle cell screening	Not categorized	Other - Matcho et al	X						
4038423	O/E - partial engagement - 2/5	Not categorized	Other - Matcho et al	X						
4149462	Down screening blood test normal	Not categorized	Other - Matcho et al	X						
4014301	Baby BW = 75%-89% (3750-4049g)	Not categorized	Other - Matcho et al	X						
439687	Complete spontaneous abortion with no mention of complication	Not categorized	Other - Matcho et al	X						
40525245	[V]Amniocentesis to screen for chromosomal anomalies	Not categorized	Other - Matcho et al	X						
40484636	Maternal postnatal examination not offered	Not categorized	Other - Matcho et al	X						
4085933	Antenatal syphilis screening categorised by action status	Not categorized	Other - Matcho et al	X						
4152385	Domino delivery	Not categorized	Other - Matcho et al	X						
4190902	Serum alkaline phosphatase placental enzyme level	Not categorized	Other - Matcho et al	X						
4071513	Immediate repair of obstetric laceration of perineum and sphincter of anus	Not categorized	Other - Matcho et al	X						
4344630	Infant death	Not categorized	Other - Matcho et al	X						
4077075	Pregnancy vitamin/iron prophylaxis	Not categorized	Other - Matcho et al	X						
4161203	Haematoma of perineal wound	Not categorized	Other - Matcho et al	X						
4014472	Apgar at 10 minutes	Not categorized	Other - Matcho et al	X						
4016058	Apgar at 10 minutes = 9	Not categorized	Other - Matcho et al	X						
4061797	Delivery booking place changed	Not categorized	Other - Matcho et al	X						
4126401	Amniotic fluid normal	Not categorized	Other - Matcho et al	X						
4169638	On examination - lie of fetus	Not categorized	Other - Matcho et al	X						
4014460	Baby BW = 10%-24% (2850-3149g)	Not categorized	Other - Matcho et al	X						
40524806	[V]High-risk pregnancy supervision	Not categorized	Other - Matcho et al	X						
4086786	Dilatation and curettage planned	Not categorized	Other - Matcho et al	X						
4149393	Disorders relating to long gestation and high birth weight	Not categorized	Other - Matcho et al	X						
4060109	Parent craft class attended	Not categorized	Other - Matcho et al	X						
4096528	Ragged membranes	Not categorized	Other - Matcho et al	X						
4208216	Maternal care for fetal bradycardia during pregnancy	Not categorized	Other - Matcho et al	X						
4326563	Trauma to vulva during delivery	Not categorized	Other - Matcho et al	X						
40524808	[V]Pregnancy with history of vesicular mole	Not categorized	Other - Matcho et al	X						
4015426	Baby BW = 3% - 9% (2500-2849g)	Not categorized	Other - Matcho et al	X						
40525259	[V]Neonatal care on NNU	Not categorized	Other - Matcho et al	X						
4061527	Feeding intention - not known	Not categorized	Other - Matcho et al	X						
40525271	[V]Twin, mate live born	Not categorized	Other - Matcho et al	X						
40524823	[V]Routine postpartum follow-up	Not categorized	Other - Matcho et al	X						
4168056	Stroke in the puerperium	Not categorized	Other - Matcho et al	X						
4039606	O/E - ROP	Not categorized	Other - Matcho et al	X						
4129043	Spasm of uterus or cervix uteri	Not categorized	Other - Matcho et al	X						
4060253	Short stay delivery booking	Not categorized	Other - Matcho et al	X						
4038940	O/E - presenting part LSA	Not categorized	Other - Matcho et al	X						
4310443	Antenatal ultrasound scan normal and possibly inconsistent with estimated date of delivery	Not categorized	Other - Matcho et al	X						
40394492	[X]Other venous complications in pregnancy	Not categorized	Other - Matcho et al	X						
4308413	Fetus	Not categorized	Other - Matcho et al	X						
40525256	[V]Normal level of neonatal care	Not categorized	Other - Matcho et al	X						
4041367	Human placental lactogen level normal	Not categorized	Other - Matcho et al	X						
40353660	[V]Menstrual extraction	Not categorized	Other - Matcho et al	X						
4061708	Maternal care for viable fetus in abdominal pregnancy	Not categorized	Other - Matcho et al	X						
4061429	Antenatal care: under 5ft tall	Not categorized	Other - Matcho et al	X						
4171106	Perinatal neurological disorder	Not categorized	Other - Matcho et al	X						
4038758	O/E - presenting ROA	Not categorized	Other - Matcho et al	X						
4042923	Fetoplacental hormones normal	Not categorized	Other - Matcho et al	X						
4347416	Cerebral injury due to birth trauma	Not categorized	Other - Matcho et al	X						
4014717	Weight of placenta	Not categorized	Other - Matcho et al	X						
4061432	Antenatal care: 10 years plus since last pregnancy	Not categorized	Other - Matcho et al	X						
4324051	Newborn blood spot screening	Not categorized	Other - Matcho et al	X						
40406767	[X]Neonatal jaundice from other specified causes	Not categorized	Other - Matcho et al	X						
40520528	[V]Screening for haemorrhagic fever	Not categorized	Other - Matcho et al	X						
4055971	Albuminuria in pregnancy without hypertension	Not categorized	Other - Matcho et al	X						
4113371	Genital or pelvic infection following abortive pregnancy	Not categorized	Other - Matcho et al	X						
4010041	Neonatal massive aspiration syndrome	Not categorized	Other - Matcho et al	X						
40394875	[X]Intrapartum hemorrhage, unspecified	Not categorized	Other - Matcho et al	X						
4243314	Determination of outcome, satisfactory to patient	Not categorized	Other - Matcho et al	X						
4146393	Pack to control postnatal vaginal bleeding	Not categorized	Other - Matcho et al	X						
4070648	Neonatal candidiasis of perineum	Not categorized	Other - Matcho et al	X						
40391771	[M]Embryonal carcinoma NOS	Not categorized	Other - Matcho et al	X						
4016465	Apgar at 5 minutes = 8	Not categorized	Other - Matcho et al	X						

4060246	A/N care: H/O child abuse	Not categorized	Other - Matcho et al	X						
40525250	[V]Screening for fetal growth retardation using ultrasonics	Not categorized	Other - Matcho et al	X						
4060250	No antenatal care: not known pregnant	Not categorized	Other - Matcho et al	X						
4146340	Syntocinon induction of labor	Not categorized	Other - Matcho et al	X						
4028780	Blood dyscrasia puerperal	Not categorized	Other - Matcho et al	X						
4280517	Malignant teratoma, trophoblastic	Not categorized	Other - Matcho et al	X						
4038757	O/E - LOA	Not categorized	Other - Matcho et al	X						
4058398	Pregnancy with abortive outcome NOS	Not categorized	Other - Matcho et al	X						
4344626	Fetal reduction or destruction procedure	Not categorized	Other - Matcho et al	X						
4062359	Maternity benefit education	Not categorized	Other - Matcho et al	X						
4034150	Bladder injury - obstetric	Not categorized	Other - Matcho et al	X						
4014302	Baby BW = 90%-96% (4050-4399g)	Not categorized	Other - Matcho et al	X						
4161678	Trauma to perineum during delivery	Not categorized	Other - Matcho et al	X						
4208218	Maternal care for fetal tachycardia during pregnancy	Not categorized	Other - Matcho et al	X						
4014435	Antenatal 42 week examination	Not categorized	Other - Matcho et al	X						
40515177	[X]Fresh stillbirth	Not categorized	Other - Matcho et al	X						
4061801	A/N amniocentesis -not offered	Not categorized	Other - Matcho et al	X						
4047868	Subcutaneous fat necrosis due to birth injury	Not categorized	Other - Matcho et al	X						
4302270	Pregnancy termination care	Not categorized	Other - Matcho et al	X						
40524816	[V]Supervision of high-risk pregnancy due to social problems	Not categorized	Other - Matcho et al	X						
4113988	Unspecified complete abortion NOS	Not categorized	Other - Matcho et al	X						
4114120	Sepsis NOS following abortion and ectopic and molar pregnancy	Not categorized	Other - Matcho et al	X						
40406766	[X]Neonatal jaundice from other and unspecified hepatocellular damage	Not categorized	Other - Matcho et al	X						
4028634	Cystocele - baby delivered	Not categorized	Other - Matcho et al	X						
4081963	Delivered uterus	Not categorized	Other - Matcho et al	X						
4015291	Apgar at 1 minute = 4	Not categorized	Other - Matcho et al	X						
4347417	Fracture of nose due to birth trauma	Not categorized	Other - Matcho et al	X						
4114143	Unspecified incomplete abortion NOS	Not categorized	Other - Matcho et al	X						
4038572	O/E - fetal heart 160-180	Not categorized	Other - Matcho et al	X						
4064793	Pregnancy dental education	Not categorized	Other - Matcho et al	X						
40581659	[V]Antenatal screening for chromosomal anomalies	Not categorized	Other - Matcho et al	X						
4084947	Chlamydial ophthalmia neonatorum	Not categorized	Other - Matcho et al	X						
443264	Maternal rubella in pregnancy, childbirth and the puerperium	Not categorized	Other - Matcho et al	X						
4253816	Infantile embryonal carcinoma	Not categorized	Other - Matcho et al	X						
40518649	[V]Problems related to unwanted pregnancy	Not categorized	Other - Matcho et al	X						
4039607	O/E - RSA	Not categorized	Other - Matcho et al	X						
4128987	Umbilical cord stump almost off	Not categorized	Other - Matcho et al	X						
4066018	Congenital malformation due to valproate	Not categorized	Other - Matcho et al	X						
4014444	P/N care started at birth	Not categorized	Other - Matcho et al	X						
4060107	Delivery booking - length of stay	Not categorized	Other - Matcho et al	X						
4066353	Maternal care for hydrops fetalis	Not categorized	Other - Matcho et al	X						
4028636	Cystocele in pregnancy, childbirth and the puerperium	Not categorized	Other - Matcho et al	X						
4119977	Placentacentesis	Not categorized	Other - Matcho et al	X						
4014155	P/N care from consultant	Not categorized	Other - Matcho et al	X						
4075178	Oxytocic induction of labour	Not categorized	Other - Matcho et al	X						
4173183	Infant of mother with gestational diabetes	Not categorized	Other - Matcho et al	X						
40394501	[X]Pre-existing diabetes mellitus, unspecified	Not categorized	Other - Matcho et al	X						
4192653	Postnatal clinic	Not categorized	Other - Matcho et al	X						
4136449	Infant feeding at birth	Not categorized	Other - Matcho et al	X						
4038763	O/E - VE - cervix ripeness	Not categorized	Other - Matcho et al	X						
4147078	Exploration of uterine cavity	Not categorized	Other - Matcho et al	X						
4073433	Hindwater rupture of amniotic membrane	Not categorized	Other - Matcho et al	X						
4110220	Amniotic sac	Not categorized	Other - Matcho et al	X						
4034076	Cystocele complicating postpartum care - baby delivered during previous episode of care	Not categorized	Other - Matcho et al	X						
4047593	Foetus or neonate affected by external version before labour	Not categorized	Other - Matcho et al	X						
4028624	Polyp of cervix in pregnancy, childbirth and the puerperium	Not categorized	Other - Matcho et al	X						
435332	Superficial thrombophlebitis in pregnancy and the puerperium with postnatal complication	Not categorized	Other - Matcho et al	X						
4070223	Immediate repair of obstetric laceration of vagina and floor of pelvis	Not categorized	Other - Matcho et al	X						
4149939	Intrapartum cardiotochogram monitoring	Not categorized	Other - Matcho et al	X						
40394879	[X]Other single spontaneous delivery	Not categorized	Other - Matcho et al	X						
4081274	Listed for Obstetric admission	Not categorized	Other - Matcho et al	X						
4055694	Carcinoembryonic antigen absent	Not categorized	Other - Matcho et al	X						
4061155	A/N U/S scan not offered	Not categorized	Other - Matcho et al	X						
4060258	A/N amniocentesis - awaited	Not categorized	Other - Matcho et al	X						
4307324	Fluid sample AFP level	Not categorized	Other - Matcho et al	X						
4071596	Birth dislocation of the shoulder	Not categorized	Other - Matcho et al	X						
4061352	Failed or difficult intubation during pregnancy	Not categorized	Other - Matcho et al	X						
4086932	Rubella screening status	Not categorized	Other - Matcho et al	X						
4254204	Artificial rupture of membranes	Not categorized	Other - Matcho et al	X						
4048141	Birth injury to face	Not categorized	Other - Matcho et al	X						
4064179	Maternal care for (suspected) damage to fetus from alcohol	Not categorized	Other - Matcho et al	X						
433533	Incomplete spontaneous abortion with no mention of complication	Not categorized	Other - Matcho et al	X						
4061528	A/N amniocentesis - not wanted	Not categorized	Other - Matcho et al	X						
4031038	Embryonal rhabdomyosarcoma, pleomorphic	Not categorized	Other - Matcho et al	X						
4138600	Obstetric uterine artery Doppler	Not categorized	Other - Matcho et al	X						
4072710	Vaginal removal of uterine foreign body	Not categorized	Other - Matcho et al	X						
4085285	Obstetric X-ray - placenta	Not categorized	Other - Matcho et al	X						
4149463	Down screening blood test abnormal	Not categorized	Other - Matcho et al	X						
4129702	Rectocele in pregnancy, childbirth and the puerperium	Not categorized	Other - Matcho et al	X						
4149443	Unborn child at risk from abnorms as to be serious handicap	Not categorized	Other - Matcho et al	X						
4074580	Fetoscopic sampling of fetal blood	Not categorized	Other - Matcho et al	X						
4014303	Baby BW = > 96% (over 4499g)	Not categorized	Other - Matcho et al	X						
4174307	Staphylococcal ophthalmia neonatorum	Not categorized	Other - Matcho et al	X						
4153436	Antenatal blood group screening categorised by action status	Not categorized	Other - Matcho et al	X						
4150949	On examination - presenting part station	Not categorized	Other - Matcho et al	X						
4073419	Forewater rupture of amniotic membrane	Not categorized	Other - Matcho et al	X						
4150651	Gas and air analgesia in labour	Not categorized	Other - Matcho et al	X						

4127549	Cystocele affecting obstetric care	Not categorized	Other - Matcho et al	X						
4028635	Pendulous abdomen in pregnancy,childbirth and the puerperium	Not categorized	Other - Matcho et al	X						
4064697	Placental transfusion syndromes	Not categorized	Other - Matcho et al	X						
4142510	Selective destruction of fetus	Not categorized	Other - Matcho et al	X						
4016487	Birth examination abnormal - referred	Not categorized	Other - Matcho et al	X						
40524797	[V]First normal pregnancy supervision	Not categorized	Other - Matcho et al	X						
4114281	Other specified pregnancy with abortive outcome	Not categorized	Other - Matcho et al	X						
4047866	Torticollis due to birth injury	Not categorized	Other - Matcho et al	X						
4236626	Drainage of fetal hydrocephalus	Not categorized	Other - Matcho et al	X						
4091640	Quickening, function	Not categorized	Other - Matcho et al	X						
40524796	[V]Supervision of normal pregnancy	Not categorized	Other - Matcho et al	X						
4021162	Pre-termination counseling	Not categorized	Other - Matcho et al	X						
4038422	O/E - LOP	Not categorized	Other - Matcho et al	X						
4039090	O/E - fetal heart 100-120	Not categorized	Other - Matcho et al	X						
4024609	Post-termination counseling	Not categorized	Other - Matcho et al	X						
40515175	[X]Cervicitis following delivery	Not categorized	Other - Matcho et al	X						
40524804	[V]Other specified pregnant state	Not categorized	Other - Matcho et al	X						
4173168	Late neonatal death	Not categorized	Other - Matcho et al	X						
4125621	Umbilical stump not separated	Not categorized	Other - Matcho et al	X						
4047732	Cerebral haematoma in foetus or newborn	Not categorized	Other - Matcho et al	X						
4085140	Xiphopagus	Not categorized	Other - Matcho et al	X						
4146935	Perinatal coagulase-negative staphylococcus	Not categorized	Other - Matcho et al	X						
40515178	[X]Macerated stillbirth	Not categorized	Other - Matcho et al	X						
40524824	[V]Other specified postpartum care and examination	Not categorized	Other - Matcho et al	X						
40406728	[X]Other respiratory distress of newborn	Not categorized	Other - Matcho et al	X						
4082319	Perinatal trauma	Not categorized	Other - Matcho et al	X						
4061807	Rhesus screening - cord blood sample	Not categorized	Other - Matcho et al	X						
4269563	Acetylcholinesterase measurement, amniotic fluid	Not categorized	Other - Matcho et al	X						
4146392	Fetus and gravid uterus operations	Not categorized	Other - Matcho et al	X						
4086468	Obstetric co-op card	Not categorized	Other - Matcho et al	X						
4247430	Tetanus omphalitis	Not categorized	Other - Matcho et al	X						
4060108	Full stay delivery booking	Not categorized	Other - Matcho et al	X						
4016056	Apgar at 5 minutes = 7	Not categorized	Other - Matcho et al	X						
4015411	P/N - shared care	Not categorized	Other - Matcho et al	X						
4070522	Very large baby - weight greater than 4500gm	Not categorized	Other - Matcho et al	X						
40521444	[X]Other antenatal screening	Not categorized	Other - Matcho et al	X						
40394497	[X]Other specified pregnancy-related conditions	Not categorized	Other - Matcho et al	X						
4113973	Unspecified legal abortion NOS	Not categorized	Other - Matcho et al	X						
4061525	Parent craft not wanted	Not categorized	Other - Matcho et al	X						
4048593	Congenital hepatitis A infection	Not categorized	Other - Matcho et al	X						
4074585	Application of fetal scalp electrode	Not categorized	Other - Matcho et al	X						
4246846	Fetal cell test	Not categorized	Other - Matcho et al	X						
4071515	Secondary repair of obstetric laceration	Not categorized	Other - Matcho et al	X						
4048283	Perinatal superficial haematoma	Not categorized	Other - Matcho et al	X						
40356742	[X]Severe mental and behavioral disorders associated with the puerperium, not elsewhere classified	Not categorized	Other - Matcho et al	X						
4015289	Apgar at 1 minute = 2	Not categorized	Other - Matcho et al	X						
4129178	Obstetrical trauma to urethra	Not categorized	Other - Matcho et al	X						
4007112	[D]Abnormality of alpha-fetoprotein	Not categorized	Other - Matcho et al	X						
40394844	[X]Maternal care for other specified known or suspected fetal problems	Not categorized	Other - Matcho et al	X						
40406715	[X]Other preterm infants	Not categorized	Other - Matcho et al	X						
4061156	A/N U/S scan not wanted	Not categorized	Other - Matcho et al	X						
4015147	No postnatal care	Not categorized	Other - Matcho et al	X						
4062417	Antepartum hemorrhage with uterine leiomyoma	Not categorized	Other - Matcho et al	X						
4061529	A/N amniocentesis wanted	Not categorized	Other - Matcho et al	X						
4034081	Bicornuate uterus - baby delivered	Not categorized	Other - Matcho et al	X						
4034155	Abscess of nipple - obstetric	Not categorized	Other - Matcho et al	X						
4171250	Viral ophthalmia neonatorum	Not categorized	Other - Matcho et al	X						
4151558	Medical certificate of still-birth	Not categorized	Other - Matcho et al	X						
4153570	Neonatal dacryocystitis or conjunctivitis due to virus	Not categorized	Other - Matcho et al	X						
4173795	Birth fracture of radius	Not categorized	Other - Matcho et al	X						
4014585	Placental diameter	Not categorized	Other - Matcho et al	X						
4014612	Birth examination abnormal - for observation	Not categorized	Other - Matcho et al	X						
4146728	Foetus or neonate affected by malposition or disproportion during labour or delivery	Not categorized	Other - Matcho et al	X						
40524866	[V]Twins, one live born and one stillborn	Not categorized	Other - Matcho et al	X						
4034149	Obstetric pelvic ligament damage	Not categorized	Other - Matcho et al	X						
40524812	[V]Pregnancy with other poor obstetric history	Not categorized	Other - Matcho et al	X						
4113353	Legal abortion unspecified	Not categorized	Other - Matcho et al	X						
4038574	O/E - VE - pelvis adequate	Not categorized	Other - Matcho et al	X						
4070533	Eye damage due to birth trauma	Not categorized	Other - Matcho et al	X						
40524809	[V]Pregnancy with history of abortion	Not categorized	Other - Matcho et al	X						
4057159	Amniotic fluid AFP abnormal	Not categorized	Other - Matcho et al	X						
4014582	Mother has a social worker	Not categorized	Other - Matcho et al	X						
4071861	Perinatal skin disorder NOS	Not categorized	Other - Matcho et al	X						
4113216	Other abnormal product of conception	Not categorized	Other - Matcho et al	X						
4015307	Bottle changed to breast	Not categorized	Other - Matcho et al	X						
4047878	Fetus or neonate affected by maternal problem unrelated to pregnancy	Not categorized	Other - Matcho et al	X						
4071192	Transitory metabolic disturbance in infant of pre-diabetic mother	Not categorized	Other - Matcho et al	X						
4061149	Parent craft - group class	Not categorized	Other - Matcho et al	X						
4061526	Parent craft -individual class	Not categorized	Other - Matcho et al	X						
4071741	Perinatal jaundice due to congenital obstruction of bile duct	Not categorized	Other - Matcho et al	X						
4034082	Bicornuate uterus affecting obstetric care	Not categorized	Other - Matcho et al	X						
4071589	Cerebral hemorrhage due to birth trauma	Not categorized	Other - Matcho et al	X						
4282460	Intrauterine cordocentesis	Not categorized	Other - Matcho et al	X						
4199558	Observation of sex of baby	Not categorized	Other - Matcho et al	X						
40566538	[X]Abnormal finding on antenatal screening of mother	Not categorized	Other - Matcho et al	X						
4014584	Risk of non-accidental injury	Not categorized	Other - Matcho et al	X						
40524800	[V]Other normal pregnancy supervision	Not categorized	Other - Matcho et al	X						
4264629	Petechiae in fetus OR newborn	Not categorized	Other - Matcho et al	X						
4074869	Introduction of abortifacient into uterine cavity	Not categorized	Other - Matcho et al	X						
4071489	Foetus or neonate affected by induction of labour	Not categorized	Other - Matcho et al	X						
4033956	Post-delivery obstetric operation	Not categorized	Other - Matcho et al	X						
4014578	Apgar at 10 minutes = 5	Not categorized	Other - Matcho et al	X						
40524807	[V]Pregnancy with history of infertility	Not categorized	Other - Matcho et al	X						

4191737	Delayed or excessive hemorrhage NOS following abortive pregnancy	Not categorized	Other - Matcho et al	X						
40525248	[V]Other screening based on amniocentesis	Not categorized	Other - Matcho et al	X						
4073411	Fetoscopic blood transfusion of fetus	Not categorized	Other - Matcho et al	X						
4062560	Early onset of delivery NOS	Not categorized	Other - Matcho et al	X						
4087245	Down screening status	Not categorized	Other - Matcho et al	X						
4347418	Kidney injury due to birth trauma	Not categorized	Other - Matcho et al	X						
40406764	[X]Neonatal jaundice due to drugs or toxins transmitted from mother or given to newborn	Not categorized	Other - Matcho et al	X						
40525288	[V]Twin, unspecified, NOS	Not categorized	Other - Matcho et al	X						
4015276	Baby birth weight 4400-4499g	Not categorized	Other - Matcho et al	X						
40406699	[X]Fetus and newborn affected by other medical procedure on mother, not elsewhere classified	Not categorized	Other - Matcho et al	X						
4028620	Septate vagina in pregnancy, childbirth and the puerperium	Not categorized	Other - Matcho et al	X						
4047867	Sternomastoid injury due to birth injury	Not categorized	Other - Matcho et al	X						
4311540	Intrauterine hypoxia AND/OR birth asphyxia	Not categorized	Other - Matcho et al	X						
40524814	[V]Supervision of pregnancy with history of insufficient antenatal care	Not categorized	Other - Matcho et al	X						
4065092	Obstetric toxic reaction to local anaesthesia	Not categorized	Other - Matcho et al	X						
4008827	[D]Placental imaging abnormal	Not categorized	Other - Matcho et al	X						
4081602	Obstetric co-op card updated	Not categorized	Other - Matcho et al	X						
4113679	Other abnormal product of conception NOS	Not categorized	Other - Matcho et al	X						
4014441	Bottle feeding stopped	Not categorized	Other - Matcho et al	X						
40525276	[V]Twin, mate stillborn	Not categorized	Other - Matcho et al	X						
4071599	Vulval haematoma due to birth trauma	Not categorized	Other - Matcho et al	X						
40394845	[X]Maternal care for known or suspected fetal problem, unspecified	Not categorized	Other - Matcho et al	X						
4028628	Rectocele complicating postpartum care - baby delivered during previous episode of care	Not categorized	Other - Matcho et al	X						
4075174	Operation on gravid uterus	Not categorized	Other - Matcho et al	X						
40524867	[V]Twins, both stillborn	Not categorized	Other - Matcho et al	X						
4061473	Obstetric cerebral venous thrombosis	Not categorized	Other - Matcho et al	X						
4158484	Obstetric investigation	Not categorized	Other - Matcho et al	X						
4028782	Perineal obstetric varicose veins	Not categorized	Other - Matcho et al	X						
4059688	Hormone radioassay - sex/placenta	Not categorized	Other - Matcho et al	X						
4129837	Knot in cord	Not categorized	Other - Matcho et al	X						
40521002	[V]Postnatal screening for chromosomal anomalies	Not categorized	Other - Matcho et al	X						
40394893	[X]Other specified puerperal infection	Not categorized	Other - Matcho et al	X						
4016187	Child not examined at birth	Not categorized	Other - Matcho et al	X						
4014310	Apgar at 5 minutes = 6	Not categorized	Other - Matcho et al	X						
40394894	[X]Other venous complications in the puerperium	Not categorized	Other - Matcho et al	X						
4071641	Immediate repair of obstetric laceration of uterus or cervix uteri	Not categorized	Other - Matcho et al	X						
40394861	[X]Labour and delivery complicated by other evidence of foetal distress	Not categorized	Other - Matcho et al	X						
4071514	Immediate repair of minor obstetric laceration	Not categorized	Other - Matcho et al	X						
4164266	Adverse reaction to gonadotrophic hormone	Not categorized	Other - Matcho et al	X						
4070421	Foetus or neonate affected by maternal anaesthetic and analgesic agents during labour and delivery	Not categorized	Other - Matcho et al	X						
4014583	H.V.: mother not managing well	Not categorized	Other - Matcho et al	X						
4089069	Relation of fetal size to dates	Not categorized	Other - Matcho et al	X						
4060256	A/N amniocentesis - offered	Not categorized	Other - Matcho et al	X						
4039089	O/E - fetal heart 40-80	Not categorized	Other - Matcho et al	X						
4047862	Birth injury to phrenic nerve	Not categorized	Other - Matcho et al	X						
4135547	Cerebrospinal fluid total human chorionic gonadotropin measurement	Not categorized	Other - Matcho et al	X						
4062125	Failed or difficult intubation during labor and delivery	Not categorized	Other - Matcho et al	X						
4014279	P/N care refused	Not categorized	Other - Matcho et al	X						
4089917	Phlegmasia alba dolens - obstetric	Not categorized	Other - Matcho et al	X						
4194736	Maternal care for central nervous system malformation in fetus	Not categorized	Other - Matcho et al	X						
4073413	Percutaneous insertion of pleuroamniotic shunt	Not categorized	Other - Matcho et al	X						
4171121	Intrapartum fetal hypoxia	Not categorized	Other - Matcho et al	X						
4058253	Orthopaedic disorders during pregnancy, childbirth and the puerperium	Not categorized	Other - Matcho et al	X						
4170122	Lymphangitis of breast - obstetric	Not categorized	Other - Matcho et al	X						
4061435	Antenatal care: not attended	Not categorized	Other - Matcho et al	X						
4064284	Maternal care for fetal hypoxia	Not categorized	Other - Matcho et al	X						
4151172	Pregnancy of less than 24 weeks involving risk of injury for the physical or mental health of the pregnant woman	Not categorized	Other - Matcho et al	X						
40406751	[X]Sepsis of newborn due to other and unspecified streptococci	Not categorized	Other - Matcho et al	X						
4091780	Placenta gritty	Not categorized	Other - Matcho et al	X						
4048147	Congenital group A haemolytic streptococcal pneumonia	Not categorized	Other - Matcho et al	X						
4122746	Cord adherent	Not categorized	Other - Matcho et al	X						
4309972	Craniopagus occipitalis	Not categorized	Other - Matcho et al	X						
4070423	Foetus or neonate affected by maternal pethidine during labour and delivery	Not categorized	Other - Matcho et al	X						
40406762	[X]Hydrops fetalis due to other and unspecified haemolytic disease	Not categorized	Other - Matcho et al	X						
4060041	Persistent occipitoposterior or occipitoanterior position	Not categorized	Other - Matcho et al	X						
4268171	Contraction ring dystocia	Not categorized	Other - Matcho et al	X						
4169728	Bruising in fetus OR newborn	Not categorized	Other - Matcho et al	X						
40525284	[V]Twin, unspecified	Not categorized	Other - Matcho et al	X						
4048290	Neonatal jaundice with glucose-6-phosphate dehydrogenase deficiency	Not categorized	Other - Matcho et al	X						
4048013	Foetus or neonate affected by maternal general anaesthesia during labour and delivery	Not categorized	Other - Matcho et al	X						
4058373	Orthopaedic disorder during pregnancy - baby not yet delivered	Not categorized	Other - Matcho et al	X						
4015280	Birth HC = 50th-74th centile	Not categorized	Other - Matcho et al	X						
4061339	Secondary postpartum hemorrhage with postnatal problem	Not categorized	Other - Matcho et al	X						
4015279	Birth HC = 25th-49th centile	Not categorized	Other - Matcho et al	X						
4014293	Ambulance birth	Not categorized	Other - Matcho et al	X						
4015283	Birth HC = > 97th centile	Not categorized	Other - Matcho et al	X						
4016473	Cot death liability	Not categorized	Other - Matcho et al	X						
4244279	Maternal death	Not categorized	Other - Matcho et al	X						
4041372	Serum pregnancy test equivocal	Not categorized	Other - Matcho et al	X						
4081577	Bruising of cord	Not categorized	Other - Matcho et al	X						

4113062	Complication NOS following abortion and ectopic and molar pregnancy	Not categorized	Other - Matcho et al	X						
4077055	Amniotic fluid for C/S	Not categorized	Other - Matcho et al	X						
40394890	[X]Other infection of genital tract following delivery	Not categorized	Other - Matcho et al	X						
40394476	[X]Other abortion	Not categorized	Other - Matcho et al	X						
4199047	Plasma free beta human chorionic gonadotropin multiple of median measurement	Not categorized	Other - Matcho et al	X						
4014471	Apgar at 5 minutes = 4	Not categorized	Other - Matcho et al	X						
4316171	Accident due to abandonment of newborn	Not categorized	Other - Matcho et al	X						
40524825	[V]Unspecified postpartum care and examination	Not categorized	Other - Matcho et al	X						
4002620	Cyst of embryonic remnant - female	Not categorized	Other - Matcho et al	X						
4149395	Mild to moderate birth asphyxia - apgar score 4-7 at 1 minute	Not categorized	Other - Matcho et al	X						
40519203	[V]Illegitimate pregnancy	Not categorized	Other - Matcho et al	X						
4071477	Fetus or neonate affected by placental insufficiency	Not categorized	Other - Matcho et al	X						
4082673	Ultrasound scan for amniotic fluid volume	Not categorized	Other - Matcho et al	X						
40391784	[M]Trophoblastic neoplasms	Not categorized	Other - Matcho et al	X						
40493181	Length of gestation at time of procedure	Not categorized	Other - Matcho et al	X						
4061162	AFP blood test wanted	Not categorized	Other - Matcho et al	X						
4113995	Salpingo-oophoritis following abortive pregnancy	Not categorized	Other - Matcho et al	X						
4149467	Pregnancy prolonged - 42 weeks	Not categorized	Other - Matcho et al	X						
4114268	Oliguria following abortive pregnancy	Not categorized	Other - Matcho et al	X						
4041368	HPL - Human placental lactogen abnormal	Not categorized	Other - Matcho et al	X						
40524820	[V]Examination immediately after delivery	Not categorized	Other - Matcho et al	X						
4048602	Perinatal rectal haemorrhage	Not categorized	Other - Matcho et al	X						
4168055	Retained membrane without haemorrhage	Not categorized	Other - Matcho et al	X						
40406740	[X]Cardiovascular disorder originating in the perinatal period, unspecified	Not categorized	Other - Matcho et al	X						
4028622	Septate vagina affecting obstetric care	Not categorized	Other - Matcho et al	X						
4060360	Rubella screening wanted	Not categorized	Other - Matcho et al	X						
4207588	Education about toxoplasmosis precautions during pregnancy	Not categorized	Other - Matcho et al	X						
4345346	Fetal aspiration pneumonitis	Not categorized	Other - Matcho et al	X						
40406713	[X]Other low birth weight	Not categorized	Other - Matcho et al	X						
4071079	Neonatal jaundice with congenital hypothyroidism	Not categorized	Other - Matcho et al	X						
4136097	Pubiotomy	Not categorized	Other - Matcho et al	X						
4114123	Bladder damage following abortive pregnancy	Not categorized	Other - Matcho et al	X						
4069976	Repositioning of umbilical cord	Not categorized	Other - Matcho et al	X						
4345344	Panniculitis in newborn	Not categorized	Other - Matcho et al	X						
4062254	Death from any obstetric cause occurring more than 42 days but less than one year after delivery	Not categorized	Other - Matcho et al	X						
4048014	Foetus or neonate affected by maternal epidural anaesthesia during labour and delivery	Not categorized	Other - Matcho et al	X						
40525246	[V]Amniotic fluid to screen for alpha-fetoprotein levels	Not categorized	Other - Matcho et al	X						
4061343	Retained products with no haemorrhage - delivered with postnatal problem	Not categorized	Other - Matcho et al	X						
40394882	[X]Other single delivery by caesarean section	Not categorized	Other - Matcho et al	X						
40399552	[X]Transitory neonatai disorder of calcium and magnesium metabolism unspecified	Not categorized	Other - Matcho et al	X						
4304009	Operation on foetus	Not categorized	Other - Matcho et al	X						
4014740	Birth examination abnormal - on treatment	Not categorized	Other - Matcho et al	X						
40394475	[X]Other specified abnormal products of conception	Not categorized	Other - Matcho et al	X						
4016055	Apgar at 5 minutes = 3	Not categorized	Other - Matcho et al	X						
4328506	[M]Placental site trophoblastic tumour	Not categorized	Other - Matcho et al	X						
4048002	Fetus or neonate affected by umbilical cord around neck	Not categorized	Other - Matcho et al	X						
434437	Varicose veins of legs in pregnancy and the puerperium with postnatal complication	Not categorized	Other - Matcho et al	X						
4042411	Fetoplacental hormones abnormal	Not categorized	Other - Matcho et al	X						
4048152	Perinatal hemoptysis	Not categorized	Other - Matcho et al	X						
40525313	[V]Unspecified birth, born in hospital	Not categorized	Other - Matcho et al	X						
4047594	Foetus or neonate affected by unstable lie before labour	Not categorized	Other - Matcho et al	X						
40394474	[X]Other ectopic pregnancy	Not categorized	Other - Matcho et al	X						
4074158	Schuchardt non-obstetric episiotomy	Not categorized	Other - Matcho et al	X						
40524871	[V]Other specified outcome of delivery	Not categorized	Other - Matcho et al	X						
4084440	Foetal heart monitoring in labour	Not categorized	Other - Matcho et al	X						
4064450	ECG: no ectopic beats	Not categorized	Other - Matcho et al	X						
4016057	Apgar at 10 minutes = 8	Not categorized	Other - Matcho et al	X						
4014316	Mother less than 20 years old	Not categorized	Other - Matcho et al	X						
4014448	Down's screening - blood sent	Not categorized	Other - Matcho et al	X						
4163524	Intrauterine transfusion	Not categorized	Other - Matcho et al	X						
4071068	Perinatal purpura	Not categorized	Other - Matcho et al	X						
4075037	Percutaneous insertion of vesicoamniotic shunt	Not categorized	Other - Matcho et al	X						
4162720	Other specified complication NOS follow abortive pregnancy	Not categorized	Other - Matcho et al	X						
4038575	O/E - VE - cervical os closed	Not categorized	Other - Matcho et al	X						
4147333	Congenital abnormality of uterus affecting obstetric care	Not categorized	Other - Matcho et al	X						
4041176	Alpha-fetoprotein equivocal	Not categorized	Other - Matcho et al	X						
4071588	Tentorial tear due to birth trauma	Not categorized	Other - Matcho et al	X						
4047590	Fetus or neonate affected by placental or breast transfer of uterine depressant	Not categorized	Other - Matcho et al	X						
40524818	[V]Unspecified high-risk pregnancy	Not categorized	Other - Matcho et al	X						
4012802	Embryonal carcinosarcoma,	Not categorized	Other - Matcho et al	X						
4127418	Suspect fetal hydrocephaly	Not categorized	Other - Matcho et al	X						
4113685	Incomplete spontaneous abortion with complication NOS	Not categorized	Other - Matcho et al	X						
4324135	Amnion nodosum	Not categorized	Other - Matcho et al	X						
4071195	Congenital faecaliths causing obstruction	Not categorized	Other - Matcho et al	X						
4075175	Repositioning of retroverted gravid uterus	Not categorized	Other - Matcho et al	X						
4072424	Intra-amniotic prostaglandin instillation	Not categorized	Other - Matcho et al	X						
197089	Peripheral nerve injury due to birth trauma	Not categorized	Other - Matcho et al	X						
4071860	Other perinatal skin disorders	Not categorized	Other - Matcho et al	X						
4062915	Early onset of delivery unspecified	Not categorized	Other - Matcho et al	X						
4071196	Other perinatal digestive system disorders	Not categorized	Other - Matcho et al	X						
4129699	Polyp of cervix - baby delivered with postpartum complication	Not categorized	Other - Matcho et al	X						
40525285	[V]Twin, unspecified, born in hospital	Not categorized	Other - Matcho et al	X						
4314756	Amniocentesis with complication, without blame	Not categorized	Other - Matcho et al	X						
4344625	Diagnostic endoscopic examination of fetus using fetoscope	Not categorized	Other - Matcho et al	X						
40524817	[V]Other specified high-risk pregnancy	Not categorized	Other - Matcho et al	X						

4047993	Foetus or neonate affected by transverse lie before labour	Not categorized	Other - Matcho et al	X						
4048139	Cerebral edema due to birth injury	Not categorized	Other - Matcho et al	X						
4039612	O/E - fetal heart 180-200	Not categorized	Other - Matcho et al	X						
4014308	Apgar at 5 minutes = 2	Not categorized	Other - Matcho et al	X						
4286483	Amniotic fluid lecithin/sphingomyelin ratio	Not categorized	Other - Matcho et al	X						
4075040	Therapeutic drainage of amniotic fluid	Not categorized	Other - Matcho et al	X						
40406737	[X]Other apnoea of newborn	Not categorized	Other - Matcho et al	X						
4048279	Intraventricular haemorrhage due to birth injury	Not categorized	Other - Matcho et al	X						
4113497	Cervix damage following abortive pregnancy	Not categorized	Other - Matcho et al	X						
4127541	Persistent hymen in pregnancy, childbirth and the puerperium	Not categorized	Other - Matcho et al	X						
40394488	[X]Edema, proteinuria and hypertensive disorders in pregnancy, childbirth and the puerperium	Not categorized	Other - Matcho et al	X						
4014462	Birth HC = < 3rd centile	Not categorized	Other - Matcho et al	X						
4345690	Fetus affected by placental insufficiency	Not categorized	Other - Matcho et al	X						
4209853	Maternal care for chromosomal abnormality in fetus	Not categorized	Other - Matcho et al	X						
4137218	Endoscopic laser ablation of placental arterio-venous anastomosis	Not categorized	Other - Matcho et al	X						
40520078	[V]Level 2 intensive care (high dependency intensive care) administered to neonates	Not categorized	Other - Matcho et al	X						
4213099	Fetus affected by maternal antepartum hemorrhage	Not categorized	Other - Matcho et al	X						
4061434	Antenatal care: not wanted	Not categorized	Other - Matcho et al	X						
4114121	Septicemia NOS following abortive pregnancy	Not categorized	Other - Matcho et al	X						
4069971	Repositioning of inverted uterus	Not categorized	Other - Matcho et al	X						
4065742	Death from sequelae of direct obstetric causes	Not categorized	Other - Matcho et al	X						
4028631	Rectocele complicating antenatal care - baby not yet delivered	Not categorized	Other - Matcho et al	X						
4070405	Fetus or neonate affected by poison transferred via placenta or breast	Not categorized	Other - Matcho et al	X						
4241331	Perinatal cutaneous ecchymoses	Not categorized	Other - Matcho et al	X						
4070420	Foetus or neonate affected by persistent occipito-posterior malposition during labour and delivery	Not categorized	Other - Matcho et al	X						
4071481	Fetus or neonate affected by fetomaternal transplacental transfusion	Not categorized	Other - Matcho et al	X						
4154314	Finding of arrangement of foetus	Not categorized	Other - Matcho et al	X						
4113374	Salpingitis following abortive pregnancy	Not categorized	Other - Matcho et al	X						
4060358	AFP blood test offered	Not categorized	Other - Matcho et al	X						
40404578	Birth trauma, asphyxia and hypoxia	Not categorized	Other - Matcho et al	X						
4048140	Scalpel wound due to birth trauma	Not categorized	Other - Matcho et al	X						
4070225	Obstetric uterine tamponade	Not categorized	Other - Matcho et al	X						
4028626	Polyp of cervix complicating antenatal care - baby not yet delivered	Not categorized	Other - Matcho et al	X						
4129185	Neonatal dacryocystitis	Not categorized	Other - Matcho et al	X						
4070534	Subconjunctival hemorrhage due to birth trauma	Not categorized	Other - Matcho et al	X						
4050255	Puerperal salpingitis	Not categorized	Other - Matcho et al	X						
4113972	Unspecified legal abortion with complication NOS	Not categorized	Other - Matcho et al	X						
443254	Intrapartum anoxia	Not categorized	Other - Matcho et al	X						
4014309	Apgar at 5 minutes = 5	Not categorized	Other - Matcho et al	X						
4073412	Late selective feticide	Not categorized	Other - Matcho et al	X						
40394885	[X]Other specified assisted single delivery	Not categorized	Other - Matcho et al	X						
4143200	Congenital or acquired abnormality of vulva in pregnancy, childbirth and the puerperium	Not categorized	Other - Matcho et al	X						
4339312	Puerperal peritonitis	Not categorized	Other - Matcho et al	X						
40513904	[V]Admission for administration of abortifacient	Not categorized	Other - Matcho et al	X						
4092290	Stillbirth [prevention record]	Not categorized	Other - Matcho et al	X						
4047716	Foetus or neonate affected by shoulder presentation during labour and delivery	Not categorized	Other - Matcho et al	X						
4064794	Pregnancy prescription exemption education	Not categorized	Other - Matcho et al	X						
4141544	Obstetric co-op card status	Not categorized	Other - Matcho et al	X						
40394898	[X]Other specified puerperal complications	Not categorized	Other - Matcho et al	X						
4062807	Antepartum haemorrhage with trauma	Not categorized	Other - Matcho et al	X						
40399561	[X]Intestinal obstruction of newborn, unspecified	Not categorized	Other - Matcho et al	X						
40525264	[V]Healthy liveborn infants according to type of birth	Not categorized	Other - Matcho et al	X						
40399555	[X]Transitory metabolic disturbance of newborn, unspecified	Not categorized	Other - Matcho et al	X						
4048012	Foetus or neonate affected by maternal analgesic agent during labour and delivery	Not categorized	Other - Matcho et al	X						
4071061	Pseudomonas pyocyaneus congenital infection	Not categorized	Other - Matcho et al	X						
4048601	Perinatal haematemesis	Not categorized	Other - Matcho et al	X						
4039611	O/E - fetal heart 80-100	Not categorized	Other - Matcho et al	X						
4064718	Septicemia during labor - delivered	Not categorized	Other - Matcho et al	X						
40525304	[V]Other multiple birth, born before admission to hospital, mates live and stillborn	Not categorized	Other - Matcho et al	X						
4197823	Congenital malaria	Not categorized	Other - Matcho et al	X						
40483097	Measurement of pH in vaginal fluid specimen using nitrazine yellow for detection of rupture of amniotic membrane	Not categorized	Other - Matcho et al	X						
4113970	Unspecified legal abortion with genital tract or pelvic infection	Not categorized	Other - Matcho et al	X						
40406768	[X]Other congenital anemias, not elsewhere classified	Not categorized	Other - Matcho et al	X						
4048613	Neonatal jaundice with Dubin-Johnson syndrome	Not categorized	Other - Matcho et al	X						
4061160	RhD antibody screening, random non-pregnancy sample	Not categorized	Other - Matcho et al	X						
4056114	Amniotic fluid chemistry: NAD	Not categorized	Other - Matcho et al	X						
40524868	[V]Other multiple birth, all live born	Not categorized	Other - Matcho et al	X						
4071592	Scalp abrasions due to birth trauma	Not categorized	Other - Matcho et al	X						
4129552	Suspect cystic fibrosis fetus	Not categorized	Other - Matcho et al	X						
4038764	O/E - VE - os=0-1cm dilated	Not categorized	Other - Matcho et al	X						
4075034	Therapeutic fetoscopic operations on fetus	Not categorized	Other - Matcho et al	X						
4062925	Fatigue during pregnancy with postnatal complication	Not categorized	Other - Matcho et al	X						
4138610	Doppler ultrasound scan of middle cerebral artery of fetus	Not categorized	Other - Matcho et al	X						
4060294	Genitourinary tract infection in pregnancy with postnatal complication	Not categorized	Other - Matcho et al	X						
4057154	Amniotic fluid microscopy	Not categorized	Other - Matcho et al	X						
4056118	Amniotic fetal cell study: NAD	Not categorized	Other - Matcho et al	X						
40394905	[X]Other obstetric conditions, not elsewhere classified	Not categorized	Other - Matcho et al	X						
4002619	Cyst of embryonic remnant - male	Not categorized	Other - Matcho et al	X						
4066134	Haemorrhoids in pregnancy and the puerperium with antenatal complication	Not categorized	Other - Matcho et al	X						
4150548	Ragged placenta	Not categorized	Other - Matcho et al	X						
40394847	[X]Other placental disorders	Not categorized	Other - Matcho et al	X						

4041369	Placental function test	Not categorized	Other - Matcho et al	X						
4153591	On examination - vaginal examination - descent of presenting part	Not categorized	Other - Matcho et al	X						
4071473	Foetus or neonate affected by complications of placenta, cord and membrane	Not categorized	Other - Matcho et al	X						
4071505	Manipulative cephalic vaginal delivery with abnormal presentation of head at delivery without instrument	Not categorized	Other - Matcho et al	X						
4111088	Acute aseptic myocarditis of the newborn	Not categorized	Other - Matcho et al	X						
4124226	Diagnostic percutaneous examination of placenta	Not categorized	Other - Matcho et al	X						
40525317	[V]Unspecified birth NOS	Not categorized	Other - Matcho et al	X						
4038576	O/E - VE - os=1-2cm dilated	Not categorized	Other - Matcho et al	X						
4047864	Testicular hematoma due to birth trauma	Not categorized	Other - Matcho et al	X						
443706	Dyscoordinate labor	Not categorized	Other - Matcho et al	X						
4029251	Congenital sepsis	Not categorized	Other - Matcho et al	X						
4066005	Post-delivery acute renal failure with postnatal problem	Not categorized	Other - Matcho et al	X						
40391787	[M]Trophoblastic neoplasm NOS	Not categorized	Other - Matcho et al	X						
40520077	[V]Special care administered to neonates	Not categorized	Other - Matcho et al	X						
4039619	O/E - VE - os=6-8cm dilated	Not categorized	Other - Matcho et al	X						
40406711	[X]Fetus and newborn affected by other maternal noxious influences	Not categorized	Other - Matcho et al	X						
4060103	Antenatal care: not offered	Not categorized	Other - Matcho et al	X						
4062628	Maternity grant education	Not categorized	Other - Matcho et al	X						
40399560	[X]Other specified perinatal digestive system disorders	Not categorized	Other - Matcho et al	X						
4153569	Neonatal dacryocystitis or conjunctivitis due to Escherichia coli	Not categorized	Other - Matcho et al	X						
40394491	[X]Other vomiting complicating pregnancy	Not categorized	Other - Matcho et al	X						
4195675	Maternal care for suspected chromosomal abnormality in fetus	Not categorized	Other - Matcho et al	X						
4169003	Pack to control postpartum haemorrhage	Not categorized	Other - Matcho et al	X						
4015140	Antenatal sickle cell screening done	Not categorized	Other - Matcho et al	X						
4147415	Genital varices in the puerperium	Not categorized	Other - Matcho et al	X						
4265196	Couvelaire uterus	Not categorized	Other - Matcho et al	X						
40521437	[X]Supervision of other normal pregnancy	Not categorized	Other - Matcho et al	X						
4038762	O/E - VE - pelvis not adequate	Not categorized	Other - Matcho et al	X						
4113369	Unspecified incomplete abortion with no mention of complication	Not categorized	Other - Matcho et al	X						
4007577	Craniopagus parasiticus	Not categorized	Other - Matcho et al	X						
4071604	Liveborn with labor abnormal heart beat	Not categorized	Other - Matcho et al	X						
4071083	Perinatal jaundice due to galactosaemia	Not categorized	Other - Matcho et al	X						
40406738	[X]Other specified respiratory conditions of newborn	Not categorized	Other - Matcho et al	X						
4063295	Fetus with radiation damage	Not categorized	Other - Matcho et al	X						
40406734	[X]Other chronic respiratory diseases originating in the perinatal period	Not categorized	Other - Matcho et al	X						
4106176	Lymphadenopathy due to congenital toxoplasmosis	Not categorized	Other - Matcho et al	X						
4015281	Birth HC = 75th-89th centile	Not categorized	Other - Matcho et al	X						
4147720	Risk to life of pregnant woman greater than if pregnancy terminated	Not categorized	Other - Matcho et al	X						
4148095	Fetus or neonate affected by placental or breast transfer of anti-infective	Not categorized	Other - Matcho et al	X						
4015285	Birth length=3rd-9th centile	Not categorized	Other - Matcho et al	X						
4070426	Fetus or neonate affected by hypertonic uterine dysfunction	Not categorized	Other - Matcho et al	X						
4153444	Foetus or neonate affected by uterine inertia or dysfunction during labour and delivery	Not categorized	Other - Matcho et al	X						
4147417	Venous complication in the puerperium, unspecified	Not categorized	Other - Matcho et al	X						
4047592	Fetus or neonate affected by placental or breast transfer of endocrine agent	Not categorized	Other - Matcho et al	X						
4273628	Baby miscellaneous at-risk factors	Not categorized	Other - Matcho et al	X						
4170447	Birth fracture of ulna	Not categorized	Other - Matcho et al	X						
4281535	Amnioscopy	Not categorized	Other - Matcho et al	X						
40394891	[X]Other genitourinary tract infections following delivery	Not categorized	Other - Matcho et al	X						
4047595	Fetus or neonate affected by placental damage caused by caesarean section	Not categorized	Other - Matcho et al	X						
4127547	Polyp of cervix complicating postnatal care - baby delivered during previous episode of care	Not categorized	Other - Matcho et al	X						
4113375	Defibrination syndrome following abortive pregnancy	Not categorized	Other - Matcho et al	X						
40521441	[X]Other antenatal screening based on amniocentesis	Not categorized	Other - Matcho et al	X						
4056119	Amniotic fetal cell abnormal	Not categorized	Other - Matcho et al	X						
40394880	[X]Other and unspecified forceps delivery	Not categorized	Other - Matcho et al	X						
4210141	Maternal care for fetal decelerations during pregnancy	Not categorized	Other - Matcho et al	X						
4194737	Maternal care for damage to fetus from maternal rubella	Not categorized	Other - Matcho et al	X						
4061812	Rubella screening not wanted	Not categorized	Other - Matcho et al	X						
4129017	Heart disease during pregnancy	Not categorized	Other - Matcho et al	X						
4113373	Pelvic peritonitis following abortive pregnancy	Not categorized	Other - Matcho et al	X						
4048748	Other perinatal digestive system disorder NOS	Not categorized	Other - Matcho et al	X						
40406720	[X]Other birth injuries to scalp	Not categorized	Other - Matcho et al	X						
4048142	Foetal death due to labour anoxia	Not categorized	Other - Matcho et al	X						
4269205	Single artery AND vein of umbilical cord	Not categorized	Other - Matcho et al	X						
4014307	Apgar at 5 minutes = 0	Not categorized	Other - Matcho et al	X						
40480440	Nitrazine yellow test for detection of rupture of amniotic membrane negative	Not categorized	Other - Matcho et al	X						
40394862	[X]Labor and delivery complicated by other cord entanglement	Not categorized	Other - Matcho et al	X						
4128032	Operation to assist delivery	Not categorized	Other - Matcho et al	X						
4047999	Fetus or neonate affected by maternofetal transplacental transfusion	Not categorized	Other - Matcho et al	X						
4074582	Early selective feticide	Not categorized	Other - Matcho et al	X						
40525272	[V]Twin, born in hospital, mate live born	Not categorized	Other - Matcho et al	X						
4034029	Rigid perineum in pregnancy, childbirth and the puerperium	Not categorized	Other - Matcho et al	X						
40406765	[X]Neonatal jaundice due to other specified excessive hemolysis	Not categorized	Other - Matcho et al	X						
40524803	[V]Supervision of other normal pregnancy	Not categorized	Other - Matcho et al	X						
40399553	[X]Neonatal goiter, not elsewhere classified	Not categorized	Other - Matcho et al	X						
4174306	Neonatal dacryocystitis due to staphylococcus	Not categorized	Other - Matcho et al	X						
4139869	Amniocentesis for possible neural tube defect	Not categorized	Other - Matcho et al	X						
40394910	[X] Other specified diseases and conditions complicating pregnancy, childbirth and the puerperium	Not categorized	Other - Matcho et al	X						
40394852	[X]Other failed induction of labour	Not categorized	Other - Matcho et al	X						
4145945	Congenital or acquired abnormality of vulva affecting obstetric care	Not categorized	Other - Matcho et al	X						

4032754	Operation on amniotic cavity	Not categorized	Other - Matcho et al	X						
4043930	Amniotic fluid microscopy -NAD	Not categorized	Other - Matcho et al	X						
4047731	Local subdural haematoma as birth trauma	Not categorized	Other - Matcho et al	X						
4015286	Birth length=25th-49th centile	Not categorized	Other - Matcho et al	X						
4016054	Apgar at 5 minutes = 1	Not categorized	Other - Matcho et al	X						
4299735	Fetal procedure	Not categorized	Other - Matcho et al	X						
4150804	Neonatal dacryocystitis or conjunctivitis due to staphylococcus	Not categorized	Other - Matcho et al	X						
40394884	[X]Other manipulation-assisted delivery	Not categorized	Other - Matcho et al	X						
4034073	Stenosis of vagina affecting obstetric care	Not categorized	Other - Matcho et al	X						
4015155	Triple test offered	Not categorized	Other - Matcho et al	X						
4072863	Endoscopic transurethral ultrasound directed oocyte recovery	Not categorized	Other - Matcho et al	X						
4048133	Extradural haemorrhage in foetus or newborn	Not categorized	Other - Matcho et al	X						
40525266	[V]Singleton, born in hospital	Not categorized	Other - Matcho et al	X						
4135546	Cerebrospinal fluid alpha-fetoprotein measurement	Not categorized	Other - Matcho et al	X						
4014474	Apgar at 10 minutes = 2	Not categorized	Other - Matcho et al	X						
4015417	Triple test wanted	Not categorized	Other - Matcho et al	X						
4071066	Subarachnoid hemorrhage due to birth injury	Not categorized	Other - Matcho et al	X						
4113499	Uremia following abortive pregnancy	Not categorized	Other - Matcho et al	X						
40406744	[X]Other bacterial sepsis of newborn	Not categorized	Other - Matcho et al	X						
4016468	Apgar at 10 minutes = 3	Not categorized	Other - Matcho et al	X						
4113967	Incomplete spontaneous abortion with other specified complication	Not categorized	Other - Matcho et al	X						
4047859	Birth plexus injury - whole plexus	Not categorized	Other - Matcho et al	X						
4173342	Coliform ophthalmia neonatorum	Not categorized	Other - Matcho et al	X						
4057162	Amniotic fetal cell study	Not categorized	Other - Matcho et al	X						
4048009	Fetus or neonate affected by abnormalities of chorion or amnion NOS	Not categorized	Other - Matcho et al	X						
4114128	Complete spontaneous abortion with complication NOS	Not categorized	Other - Matcho et al	X						
4014463	Birth HC = 3rd-9th centile	Not categorized	Other - Matcho et al	X						
4014467	Birth length = > 97th centile	Not categorized	Other - Matcho et al	X						
4185780	Gestation period, 21 weeks		Gestation period, X weeks (GW)			X	X	X	X	X
4277749	Gestation period, 17 weeks		Gestation period, X weeks (GW)			X	X	X	X	X
4049621	Gestation period, 16 weeks		Gestation period, X weeks (GW)			X	X	X	X	X
4097608	Gestation period, 18 weeks		Gestation period, X weeks (GW)			X	X	X	X	X
4336226	Gestation period, 23 weeks		Gestation period, X weeks (GW)			X	X	X	X	X
4197245	Gestation period, 12 weeks		Gestation period, X weeks (GW)			X	X	X	X	X
434484	Gestation period, 30 weeks		Gestation period, X weeks (GW)			X	X	X	X	X
4283690	Gestation period, 15 weeks		Gestation period, X weeks (GW)			X	X	X	X	X
4174506	Gestation period, 11 weeks		Gestation period, X weeks (GW)			X	X	X	X	X
435655	Gestation period, 39 weeks		Gestation period, X weeks (GW)			X	X	X	X	X
438543	Gestation period, 36 weeks		Gestation period, X weeks (GW)			X	X	X	X	X
443871	Gestation period, 38 weeks		Gestation period, X weeks (GW)			X	X	X	X	X
444023	Gestation period, 26 weeks		Gestation period, X weeks (GW)			X	X	X	X	X
442355	Gestation period, 37 weeks		Gestation period, X weeks (GW)			X	X	X	X	X
4248725	Gestation period, 14 weeks		Gestation period, X weeks (GW)			X	X	X	X	X
4266517	Gestation period, 13 weeks		Gestation period, X weeks (GW)			X	X	X	X	X
4242241	Gestation period, 10 weeks		Gestation period, X weeks (GW)			X	X	X	X	X
4181751	Gestation period, 19 weeks		Gestation period, X weeks (GW)			X	X	X	X	X
4274955	Gestation period, 22 weeks		Gestation period, X weeks (GW)			X	X	X	X	X
443874	Gestation period, 34 weeks		Gestation period, X weeks (GW)			X	X	X	X	X
442558	Gestation period, 32 weeks		Gestation period, X weeks (GW)			X	X	X	X	X
444461	Gestation period, 28 weeks		Gestation period, X weeks (GW)			X	X	X	X	X
4051642	Gestation period, 20 weeks		Gestation period, X weeks (GW)			X	X	X	X	X
4132434	Gestation period, 8 weeks		Gestation period, X weeks (GW)			X	X	X	X	X
3016670	Alpha-1-Fetoprotein [Multiple of the median] adjusted in Serum or Plasma		Gestational age, one week to three months (GR3m)				X		X	X
2212323	Estriol		Gestational age, one week to three months (GR3m)				X		X	X
4239938	First trimester pregnancy		Gestational age, one week to three months (GR3m)				X		X	X
4322726	Gestation less than 9 weeks		Gestational age, one week to three months (GR3m)				X		X	X
3016699	Glucose [Mass/volume] in Serum or Plasma --1 hour post 50 g glucose PO		Gestational age, one week to three months (GR3m)				X		X	X
2212361	Glucose; post glucose dose (includes glucose)		Gestational age, one week to three months (GR3m)				X		X	X
2212362	Glucose; tolerance test (GTT), 3 specimens (includes glucose)		Gestational age, one week to three months (GR3m)				X		X	X
2212363	Glucose; tolerance test, each additional beyond 3 specimens (List separately in addition to code for primary procedure)		Gestational age, one week to three months (GR3m)				X		X	X
37016152	Group B streptococcus carrier complicating pregnancy		Gestational age, one week to three months (GR3m)				X		X	X
2213172	Infectious agent detection by nucleic acid (DNA or RNA); Streptococcus, group B, amplified probe technique		Gestational age, one week to three months (GR3m)				X		X	X
3012620	Inhibin [Mass/volume] in Serum or Plasma		Gestational age, one week to three months (GR3m)				X		X	X

2212802	Inhibin A		Gestational age, one week to three months (GR3m)				X		X	X
3035828	Inhibin A [Multiple of the median] in Serum or Plasma		Gestational age, one week to three months (GR3m)				X		X	X
3048541	Neural tube defect risk [Likelihood] in Fetus		Gestational age, one week to three months (GR3m)				X		X	X
2212538	Progesterone		Gestational age, one week to three months (GR3m)				X		X	X
3027144	Progesterone [Mass/volume] in Serum or Plasma		Gestational age, one week to three months (GR3m)				X		X	X
4244438	Second trimester pregnancy		Gestational age, one week to three months (GR3m)				X		X	X
3049229	Second trimester quad maternal screen [Interpretation] in Serum or Plasma Narrative		Gestational age, one week to three months (GR3m)				X		X	X
3036000	Streptococcus agalactiae [Presence] in Specimen by Organism specific culture		Gestational age, one week to three months (GR3m)				X		X	X
43055441	Streptococcus agalactiae [Presence] in Vag+Rectum by Organism specific culture		Gestational age, one week to three months (GR3m)				X		X	X
3048882	Streptococcus agalactiae DNA [Presence] in Specimen by NAA with probe detection		Gestational age, one week to three months (GR3m)				X		X	X
3047352	Trisomy 18 risk [Likelihood] in Fetus		Gestational age, one week to three months (GR3m)				X		X	X
3043238	Trisomy 21 risk [Likelihood] in Fetus		Gestational age, one week to three months (GR3m)				X		X	X
4084768	Uncertain viability of pregnancy		Gestational age, one week to three months (GR3m)				X		X	X
37312440	Lower uterine segment cesarean section		Delivery record only (DELIV)			X	X	X		
2784578	Delivery of Products of Conception, External Approach		Delivery record only (DELIV)			X	X	X		
43018344	Manual Extraction of Products of Conception, Retained, Via Natural or Artificial Opening		Delivery record only (DELIV)			X	X	X		
2784573	Extraction of Products of Conception, Retained, Via Natural or Artificial Opening		Delivery record only (DELIV)			X	X	X		
2784570	Extraction of Products of Conception, Vacuum, Via Natural or Artificial Opening		Delivery record only (DELIV)			X	X	X		
2784567	Extraction of Products of Conception, Low Forceps, Via Natural or Artificial Opening		Delivery record only (DELIV)			X	X	X		
2784565	Extraction of Products of Conception, Low, Open Approach		Delivery record only (DELIV)			X	X	X		
2784564	Extraction of Products of Conception, High, Open Approach		Delivery record only (DELIV)			X	X	X		
4174577	Secondary abdominal pregnancy		Ectopic pregnancy (ECT)			X	X	X		
4245908	Gestation period, 9 weeks		Gestation period, X weeks (GW)			X	X	X		X
442769	Gestation period, 41 weeks		Gestation period, X weeks (GW)			X	X	X		X
3012266	Gestational age		Gestational age, other			X	X	X		X
3002209	Gestational age Estimated		Gestational age, other			X	X	X		X
36713074	Single liveborn born in hospital by vaginal delivery		Livebirth (LB)			X	X	X		
37312440	Lower uterine segment cesarean section		Cesarean section			X	X			
40488298	Ultrasonography in first trimester		First trimester				X			
4239938	First trimester pregnancy		First trimester				X			
3050129	First trimester maternal screen panel - Serum or Plasma		First trimester				X			
3031648	First trimester maternal screen with nuchal translucency [Interpretation] Narrative		First trimester				X			
45772076	Premature rupture of membranes in full term pregnancy with onset of labor more than 24 hours after rupture		Full term				X			
45757123	Premature rupture of membranes in full term pregnancy with onset of labor unknown		Full term				X			
45757122	Premature rupture of membranes in full term pregnancy with onset of labor within 24 hours of rupture		Full term				X			
44784551	Preterm spontaneous labor with term delivery		Full term				X			
4336958	Term pregnancy		Full term				X			
21493000	Date of gestational age estimate		Gestational age, other				X			
3026070	Delivery date Estimated from last menstrual period		Gestational age, other				X			
3024261	Delivery date US composite estimate		Gestational age, other				X			
3039154	Gestational age Estimated from conception date		Gestational age, other				X			
3001105	Gestational age Estimated from last menstrual period		Gestational age, other				X			
1175142	Gestational age Estimated from physical exam		Gestational age, other				X			
3050433	Gestational age in days		Gestational age, other				X			
4060186	Gravida		Gravidity				X			
45765734	Supervision of high risk pregnancy for primigravida age 15 years or younger		Gravidity				X			
45765733	Supervision of high risk pregnancy for multigravida age 15 years or younger		Gravidity				X			
40481872	Multigravida of advanced maternal age		Gravidity				X			
4012560	Multigravida		Gravidity				X			
43021054	Dichorionic diamniotic twin pregnancy		Multiple pregnancy				X			
43021951	Monochorionic diamniotic twin pregnancy		Multiple pregnancy				X			
3030180	Alpha-1-Fetoprotein [Multiple of the median] adjusted for multiple gestations in Serum or Plasma		Multiple pregnancy				X			
40758410	Number of fetuses		Number of fetuses				X			
3002549	Number of fetuses by US		Number of fetuses				X			
3031029	2-Ethylidene-1,5-Dimethyl-3,3-Diphenylpyrrolidine (EDDP) [Mass/volume] in Urine		Other - Specific to initial possibly-pregnant cohort (633K)				X			
4248851	Treponema pallidum		Other - Specific to initial possibly-pregnant cohort (633K)				X			
40225812	50 ML bupivacaine hydrochloride 2.5 MG/ML Prefilled Syringe		Other - Specific to initial possibly-pregnant cohort (633K)				X			
36304260	Rh group Ag [Type] on Red Blood Cells		Other - Specific to initial possibly-pregnant cohort (633K)				X			
1314330	Hepatitis b screening in non-pregnant, high risk individual includes hepatitis b surface antigen (hbsag), antibodies to hbsag (anti-hbs) and antibodies to hepatitis b core antigen (anti-hbc), and is followed by a neutralizing confirmatory test, when pe...		Other - Specific to initial possibly-pregnant cohort (633K)				X			
4027509	Contraception		Other - Specific to initial possibly-pregnant cohort (633K)				X			
40761466	7-Aminoclonazepam [Presence] in Urine by Confirmatory method		Other - Specific to initial possibly-pregnant cohort (633K)				X			

19111620	folic acid		Other - Specific to initial possibly-pregnant cohort (633K)				X			
40175249	nalbuphine hydrochloride 10 MG/ML Injectable Solution		Other - Specific to initial possibly-pregnant cohort (633K)				X			
40224108	1 ML medroxyprogesterone acetate 150 MG/ML Prefilled Syringe		Other - Specific to initial possibly-pregnant cohort (633K)				X			
3025156	Microscopic observation [Identifier] in Cervical or vaginal smear or scraping by Cyto stain		Other - Specific to initial possibly-pregnant cohort (633K)				X			
45766222	Requires diphtheria, tetanus and pertussis vaccination		Other - Specific to initial possibly-pregnant cohort (633K)				X			
46236017	Lead [Mass/volume] in Venous blood		Other - Specific to initial possibly-pregnant cohort (633K)				X			
35605342	ampicillin 1000 MG Injection		Other - Specific to initial possibly-pregnant cohort (633K)				X			
19058045	desogestrel 0.15 MG / ethinyl estradiol 0.03 MG Oral Tablet		Other - Specific to initial possibly-pregnant cohort (633K)				X			
3051893	Chlamydia trachomatis L2 DNA [Presence] in Specimen by NAA with probe detection		Other - Specific to initial possibly-pregnant cohort (633K)				X			
40224805	1 ML medroxyprogesterone acetate 150 MG/ML Injection		Other - Specific to initial possibly-pregnant cohort (633K)				X			
40224109	1 ML medroxyprogesterone acetate 150 MG/ML Prefilled Syringe [Depo-Provera]		Other - Specific to initial possibly-pregnant cohort (633K)				X			
2213109	Infectious agent detection by nucleic acid (DNA or RNA); Candida species, amplified probe technique		Other - Specific to initial possibly-pregnant cohort (633K)				X			
3023895	Varicella zoster virus IgG Ab [Units/volume] in Serum by Immunoassay		Other - Specific to initial possibly-pregnant cohort (633K)				X			
42800473	0.5 ML Bordetella pertussis filamentous hemagglutinin vaccine, inactivated 0.016 MG/ML / Bordetella pertussis pertactin vaccine, inactivated 0.005 UNT/ML / Bordetella pertussis toxoid vaccine, inactivated 0.016 MG/ML / diphtheria toxoid vaccine, inacti...		Other - Specific to initial possibly-pregnant cohort (633K)				X			
19007902	ibuprofen 600 MG Oral Tablet [Arthrofen 600]		Other - Specific to initial possibly-pregnant cohort (633K)				X			
3021461	Reagin Ab [Presence] in Serum by RPR		Other - Specific to initial possibly-pregnant cohort (633K)				X			
2110171	Dilation and curettage, diagnostic and/or therapeutic (nonobstetrical)		Other - Specific to initial possibly-pregnant cohort (633K)				X			
3035214	Glucose [Mass/volume] in Urine collected for unspecified duration		Other - Specific to initial possibly-pregnant cohort (633K)				X			
438066	Chlamydial infection		Other - Specific to initial possibly-pregnant cohort (633K)				X			
195873	Leukorrhea		Other - Specific to initial possibly-pregnant cohort (633K)				X			
3044651	Treponema pallidum IgG+IgM Ab [Presence] in Serum		Other - Specific to initial possibly-pregnant cohort (633K)				X			
42800030	0.5 ML varicella-zoster virus vaccine live (Oka-Merck) strain 2700 UNT/ML Injection		Other - Specific to initial possibly-pregnant cohort (633K)				X			
42707526	hydrocortisone acetate 25 MG/ML / pramoxine hydrochloride 10 MG/ML Topical Cream		Other - Specific to initial possibly-pregnant cohort (633K)				X			
906891	metoclopramide 10 MG Oral Tablet		Other - Specific to initial possibly-pregnant cohort (633K)				X			
2213281	Level II - Surgical pathology, gross and microscopic examination Appendix, incidental Fallopian tube, sterilization Fingers/toes, amputation, traumatic Foreskin, newborn Hernia sac, any location Hydrocele sac Nerve Skin, plastic repair Sympathetic ganglion Testis, castration Vaginal mucosa, incidental Vas deferens, sterilization		Other - Specific to initial possibly-pregnant cohort (633K)				X			
4019953	Donor for medical or surgical procedure		Other - Specific to initial possibly-pregnant cohort (633K)				X			
759681	Infectious disease, bacterial vaginosis, quantitative real-time amplification of RNA markers for Atopobium vaginae, Gardnerella vaginalis, and Lactobacillus species, utilizing vaginal-fluid specimens, algorithm reported as a positive or negative result for bacterial vaginosis		Other - Specific to initial possibly-pregnant cohort (633K)				X			
3012236	Microscopic observation [Identifier] in Specimen by Wet preparation		Other - Specific to initial possibly-pregnant cohort (633K)				X			
2212758	Blood bank physician services; difficult cross match and/or evaluation of irregular antibody(s), interpretation and written report		Other - Specific to initial possibly-pregnant cohort (633K)				X			
4086928	IUCD status		Other - Specific to initial possibly-pregnant cohort (633K)				X			
1519937	etonogestrel 68 MG Drug Implant		Other - Specific to initial possibly-pregnant cohort (633K)				X			
4183468	Group B streptococcus		Other - Specific to initial possibly-pregnant cohort (633K)				X			
3044017	Ethanol [Presence] in Urine by Confirmatory method		Other - Specific to initial possibly-pregnant cohort (633K)				X			

977968	sodium citrate		Other - Specific to initial possibly-pregnant cohort (633K)				X			
35606292	100 ML ropivacaine hydrochloride 2 MG/ML Injection		Other - Specific to initial possibly-pregnant cohort (633K)				X			
1396808	100 ML tranexamic acid 10 MG/ML Injection		Other - Specific to initial possibly-pregnant cohort (633K)				X			
40318194	Primary amenorrhea		Other - Specific to initial possibly-pregnant cohort (633K)				X			
941478	terconazole 8 MG/ML Vaginal Cream		Other - Specific to initial possibly-pregnant cohort (633K)				X			
2110221	Hysteroscopy, diagnostic (separate procedure)		Other - Specific to initial possibly-pregnant cohort (633K)				X			
19132304	levonorgestrel 0.000833 MG/HR Intrauterine System [Mirena]		Other - Specific to initial possibly-pregnant cohort (633K)				X			
2617251	Skilled services of a licensed nurse (lpn or rn) for the observation and assessment of the patient's condition, each 15 minutes (the change in the patient's condition requires skilled nursing personnel to identify and evaluate the patient's need for po...		Other - Specific to initial possibly-pregnant cohort (633K)				X			
532272	measles virus vaccine live, Enders' attenuated Edmonston strain		Other - Specific to initial possibly-pregnant cohort (633K)				X			
40169689	labetalol hydrochloride 200 MG Oral Tablet		Other - Specific to initial possibly-pregnant cohort (633K)				X			
3035962	HIV 1+2 Ab [Presence] in Serum or Plasma by Immunoassay		Other - Specific to initial possibly-pregnant cohort (633K)				X			
3011520	Treponema pallidum Ab [Presence] in Serum by Immunoassay		Other - Specific to initial possibly-pregnant cohort (633K)				X			
2212772	Complement fixation tests, each antigen		Other - Specific to initial possibly-pregnant cohort (633K)				X			
19027222	labetalol hydrochloride 200 MG Oral Tablet [Labrocol]		Other - Specific to initial possibly-pregnant cohort (633K)				X			
35603391	azithromycin 500 MG Injection [Zithromax]		Other - Specific to initial possibly-pregnant cohort (633K)				X			
40761809	Adulterants panel - Urine		Other - Specific to initial possibly-pregnant cohort (633K)				X			
2212939	Antibody identification, RBC antibodies, each panel for each serum technique		Other - Specific to initial possibly-pregnant cohort (633K)				X			
19121383	betamethasone 3 MG/ML / betamethasone acetate 3 MG/ML Injectable Suspension		Other - Specific to initial possibly-pregnant cohort (633K)				X			
4238715	Removal of intrauterine device		Other - Specific to initial possibly-pregnant cohort (633K)				X			
3041012	Bilirubin.total [Moles/volume] in Urine by Test strip		Other - Specific to initial possibly-pregnant cohort (633K)				X			
35603828	30 ML bupivacaine hydrochloride 7.5 MG/ML Injection [Sensorcaine]		Other - Specific to initial possibly-pregnant cohort (633K)				X			
3004655	Varicella zoster virus Ab [Presence] in Serum		Other - Specific to initial possibly-pregnant cohort (633K)				X			
3015015	HIV 2 Ab [Presence] in Serum		Other - Specific to initial possibly-pregnant cohort (633K)				X			
4170573	History of contraceptive usage		Other - Specific to initial possibly-pregnant cohort (633K)				X			
40229196	aluminum hydroxide 40 MG/ML / magnesium hydroxide 40 MG/ML / simethicone 4 MG/ML Oral Suspension [Milantex]		Other - Specific to initial possibly-pregnant cohort (633K)				X			
4243365	Congenital anomaly of female genital system		Other - Specific to initial possibly-pregnant cohort (633K)				X			
3036472	Blood group antibodies identified in Serum or Plasma		Other - Specific to initial possibly-pregnant cohort (633K)				X			
2102138	Removal, non-biodegradable drug delivery implant		Other - Specific to initial possibly-pregnant cohort (633K)				X			
2110194	Insertion of intrauterine device (IUD)		Other - Specific to initial possibly-pregnant cohort (633K)				X			
941473	terconazole 80 MG Vaginal Insert		Other - Specific to initial possibly-pregnant cohort (633K)				X			
44814344	magnesium hydroxide 80 MG/ML Oral Suspension [Magmex]		Other - Specific to initial possibly-pregnant cohort (633K)				X			
19033921	ibuprofen 600 MG Oral Tablet [Ibu]		Other - Specific to initial possibly-pregnant cohort (633K)				X			

2514415	Office consultation for a new or established patient, which requires these 3 key components: A problem focused history; A problem focused examination; and Straightforward medical decision making. Counseling and/or coordination of care with other physicians, other qualified health care professionals, or agencies are provided consistent with the nature of the problem(s) and the patient's and/or family's needs. Usually, the presenting problem(s) are self limited or minor. Typically, 15 minutes are spent face-to-face with the patient and/or family.		Other - Specific to initial possibly-pregnant cohort (633K)				X			
3042761	Neisseria gonorrhoeae DNA [Presence] in Specimen by Probe with signal amplification		Other - Specific to initial possibly-pregnant cohort (633K)				X			
35603389	azithromycin 500 MG Injection		Other - Specific to initial possibly-pregnant cohort (633K)				X			
3045641	Bacterial vaginosis score		Other - Specific to initial possibly-pregnant cohort (633K)				X			
44816279	ethinyl estradiol 0.00146 MG/HR / norelgestromin 0.00625 MG/HR Transdermal System		Other - Specific to initial possibly-pregnant cohort (633K)				X			
1552350	progesterone 200 MG Oral Capsule		Other - Specific to initial possibly-pregnant cohort (633K)				X			
4205771	Ultrasonography of soft tissue		Other - Specific to initial possibly-pregnant cohort (633K)				X			
4191386	Fertility care		Other - Specific to initial possibly-pregnant cohort (633K)				X			
3012923	Secobarbital [Mass/volume] in Urine by Confirmatory method		Other - Specific to initial possibly-pregnant cohort (633K)				X			
3026572	PHENobarbital [Mass/volume] in Urine by Confirmatory method		Other - Specific to initial possibly-pregnant cohort (633K)				X			
3044965	Chlamydia trachomatis DNA [Presence] in Specimen by Probe with signal amplification		Other - Specific to initial possibly-pregnant cohort (633K)				X			
21125875	Multivitamin preparation Oral Tablet		Other - Specific to initial possibly-pregnant cohort (633K)				X			
3026417	Butalbital [Mass/volume] in Urine by Confirmatory method		Other - Specific to initial possibly-pregnant cohort (633K)				X			
194871	Trichomonal vulvovaginitis		Other - Specific to initial possibly-pregnant cohort (633K)				X			
3041870	Hepatitis C virus IgG Ab [Presence] in Serum or Plasma by Immunoassay		Other - Specific to initial possibly-pregnant cohort (633K)				X			
529713	mumps virus vaccine live, Jeryl Lynn strain		Other - Specific to initial possibly-pregnant cohort (633K)				X			
3022575	Varicella zoster virus IgG Ab [Units/volume] in Serum		Other - Specific to initial possibly-pregnant cohort (633K)				X			
45775208	levomefolate		Other - Specific to initial possibly-pregnant cohort (633K)				X			
3030989	Hemoglobin A1/Hemoglobin.total in Blood by Electrophoresis		Other - Specific to initial possibly-pregnant cohort (633K)				X			
3050361	Neisseria gonorrhoeae DNA [Presence] in Genital specimen by NAA with probe detection		Other - Specific to initial possibly-pregnant cohort (633K)				X			
19041103	terconazole 4 MG/ML Vaginal Cream [Terazol 7]		Other - Specific to initial possibly-pregnant cohort (633K)				X			
4181604	Subcutaneous contraceptive implant present		Other - Specific to initial possibly-pregnant cohort (633K)				X			
3046071	Choriogonadotropin.intact+Beta subunit [Units/volume] in Serum or Plasma		Other - Specific to initial possibly-pregnant cohort (633K)				X			
4250598	Contraception care management		Other - Specific to initial possibly-pregnant cohort (633K)				X			
3049147	HIV 1+O+2 Ab [Units/volume] in Serum or Plasma		Other - Specific to initial possibly-pregnant cohort (633K)				X			
2110611	Injection, epidural, of blood or clot patch		Other - Specific to initial possibly-pregnant cohort (633K)				X			
35603815	bupivacaine hydrochloride 7.5 MG/ML Injection		Other - Specific to initial possibly-pregnant cohort (633K)				X			
19121385	betamethasone 3 MG/ML / betamethasone acetate 3 MG/ML Injectable Suspension [Celestone Soluspan]		Other - Specific to initial possibly-pregnant cohort (633K)				X			
40757049	Antibody; Treponema pallidum		Other - Specific to initial possibly-pregnant cohort (633K)				X			
3024421	Chlamydia trachomatis DNA [Presence] in Genital specimen by NAA with probe detection		Other - Specific to initial possibly-pregnant cohort (633K)				X			
44816657	Yeast [Presence] in Specimen by Gram stain		Other - Specific to initial possibly-pregnant cohort (633K)				X			
46235448	HIV 1+2 Ab and HIV1 p24 Ag [Identifier] in Serum, Plasma or Blood by Rapid immunoassay		Other - Specific to initial possibly-pregnant cohort (633K)				X			
3025939	Karyotype [Identifier] in Blood or Tissue Nominal		Other - Specific to initial possibly-pregnant cohort (633K)				X			

3051969	Bacterial vaginosis and vaginitis rRNA panel - Vaginal fluid by Probe		Other - Specific to initial possibly-pregnant cohort (633K)				X			
42800463	0.5 ML Bordetella pertussis filamentous hemagglutinin vaccine, inactivated 0.01 MG/ML / Bordetella pertussis fimbriae 2/3 vaccine, inactivated 0.01 MG/ML / Bordetella pertussis pertactin vaccine, inactivated 0.006 MG/ML / Bordetella pertussis toxoid va...		Other - Specific to initial possibly-pregnant cohort (633K)				X			
79072	Inflammatory disorder of breast		Other - Specific to initial possibly-pregnant cohort (633K)				X			
1314320	Drug test(s), definitive, utilizing (1) drug identification methods able to identify individual drugs and distinguish between structural isomers (but not necessarily stereoisomers), including, but not limited to gc/ms (any type, single or tandem) and l...		Other - Specific to initial possibly-pregnant cohort (633K)				X			
2213124	Infectious agent detection by nucleic acid (DNA or RNA); Gardnerella vaginalis, quantification		Other - Specific to initial possibly-pregnant cohort (633K)				X			
3007921	HIV 1 Ag [Presence] in Serum		Other - Specific to initial possibly-pregnant cohort (633K)				X			
40041368	etonogestrel Drug Implant		Other - Specific to initial possibly-pregnant cohort (633K)				X			
19057346	copper sulfate		Other - Specific to initial possibly-pregnant cohort (633K)				X			
43527963	Unlisted multianalyte assay with algorithmic analysis		Other - Specific to initial possibly-pregnant cohort (633K)				X			
3022251	Phencyclidine [Mass/volume] in Urine by Confirmatory method		Other - Specific to initial possibly-pregnant cohort (633K)				X			
40241844	simethicone 80 MG Chewable Tablet [Bicarsim]		Other - Specific to initial possibly-pregnant cohort (633K)				X			
19062817	riboflavin		Other - Specific to initial possibly-pregnant cohort (633K)				X			
3014204	Oxygen saturation in Venous cord blood		Other - Specific to initial possibly-pregnant cohort (633K)				X			
2212903	Antibody; parvovirus		Other - Specific to initial possibly-pregnant cohort (633K)				X			
40768325	Hepatitis B virus surface Ag [Presence] in Serum or Plasma by Confirmatory method		Other - Specific to initial possibly-pregnant cohort (633K)				X			
3038943	Toxoplasma gondii IgM Ab [Presence] in Serum or Plasma by Immunoassay		Other - Specific to initial possibly-pregnant cohort (633K)				X			
4064925	Hypertension screening		Other - Specific to initial possibly-pregnant cohort (633K)				X			
35604745	medroxyprogesterone Injection		Other - Specific to initial possibly-pregnant cohort (633K)				X			
3014651	traMADol cutoff [Mass/volume] in Urine for Screen method		Other - Specific to initial possibly-pregnant cohort (633K)				X			
3019601	Blood group antibody investigation [Interpretation] in Plasma or RBC		Other - Specific to initial possibly-pregnant cohort (633K)				X			
43054905	Erythrocytes [Presence] in Specimen by Gram stain		Other - Specific to initial possibly-pregnant cohort (633K)				X			
21027865	Multivitamin preparation Oral Capsule		Other - Specific to initial possibly-pregnant cohort (633K)				X			
3020200	Chlamydia trachomatis DNA [Presence] in Cervix by NAA with probe detection		Other - Specific to initial possibly-pregnant cohort (633K)				X			
3047826	Mullerian inhibiting substance [Mass/volume] in Serum or Plasma		Other - Specific to initial possibly-pregnant cohort (633K)				X			
19008339	vitamin A		Other - Specific to initial possibly-pregnant cohort (633K)				X			
2101590	Anesthesia for vaginal procedures (including biopsy of labia, vagina, cervix or endometrium); not otherwise specified		Other - Specific to initial possibly-pregnant cohort (633K)				X			
4261660	Normal body mass index		Other - Specific to initial possibly-pregnant cohort (633K)				X			
523212	rubella virus vaccine live (Wistar RA 27-3 strain)		Other - Specific to initial possibly-pregnant cohort (633K)				X			
3004574	Meperidine [Mass/volume] in Urine		Other - Specific to initial possibly-pregnant cohort (633K)				X			
3050141	Clinical cytogeneticist [Identifier] in Specimen		Other - Specific to initial possibly-pregnant cohort (633K)				X			
3048453	Treponema pallidum IgG+IgM Ab [Presence] in Serum by Immunoassay		Other - Specific to initial possibly-pregnant cohort (633K)				X			
2102134	Removal, implantable contraceptive capsules		Other - Specific to initial possibly-pregnant cohort (633K)				X			
19125066	menthol 3.3 MG Oral Lozenge		Other - Specific to initial possibly-pregnant cohort (633K)				X			
40757116	Culture, typing; identification by nucleic acid (DNA or RNA) probe, amplified probe technique, per culture or isolate, each organism probed		Other - Specific to initial possibly-pregnant cohort (633K)				X			

35604843	morphine sulfate 1 MG/ML Injection		Other - Specific to initial possibly-pregnant cohort (633K)				X			
1400498	iron carbonyl		Other - Specific to initial possibly-pregnant cohort (633K)				X			
968985	calcium chloride 0.001 MEQ/ML / glucose 50 MG/ML / potassium chloride 0.004 MEQ/ML / sodium chloride 0.103 MEQ/ML / sodium lactate 0.028 MEQ/ML Injectable Solution		Other - Specific to initial possibly-pregnant cohort (633K)				X			
1593919	30 ML epinephrine 0.005 MG/ML / lidocaine hydrochloride 15 MG/ML Injection [Xylocaine with Epinephrine]		Other - Specific to initial possibly-pregnant cohort (633K)				X			
3019832	Treponema pallidum Ab [Presence] in Serum		Other - Specific to initial possibly-pregnant cohort (633K)				X			
19071128	copper		Other - Specific to initial possibly-pregnant cohort (633K)				X			
3043533	Chlamydia trachomatis DNA [Presence] in Vaginal fluid by NAA with probe detection		Other - Specific to initial possibly-pregnant cohort (633K)				X			
3026408	clonazePAM [Mass/volume] in Urine		Other - Specific to initial possibly-pregnant cohort (633K)				X			
4275113	Insertion of intrauterine contraceptive device		Other - Specific to initial possibly-pregnant cohort (633K)				X			
3046574	Neisseria gonorrhoeae DNA [Presence] in Vaginal fluid by NAA with probe detection		Other - Specific to initial possibly-pregnant cohort (633K)				X			
40243346	atropine sulfate 0.025 MG / diphenoxylate hydrochloride 2.5 MG Oral Tablet [Vi-Atro]		Other - Specific to initial possibly-pregnant cohort (633K)				X			
3020293	diazePAM [Mass/volume] in Urine		Other - Specific to initial possibly-pregnant cohort (633K)				X			
4042067	Impaired glucose tolerance test		Other - Specific to initial possibly-pregnant cohort (633K)				X			
3014361	Oxazepam cutoff [Mass/volume] in Urine for Screen method		Other - Specific to initial possibly-pregnant cohort (633K)				X			
197606	Female infertility of tubal origin		Other - Specific to initial possibly-pregnant cohort (633K)				X			
941477	terconazole 4 MG/ML Vaginal Cream		Other - Specific to initial possibly-pregnant cohort (633K)				X			
2514533	Preventive medicine counseling and/or risk factor reduction intervention(s) provided to an individual (separate procedure); approximately 60 minutes		Other - Specific to initial possibly-pregnant cohort (633K)				X			
42708658	docusate sodium 100 MG Oral Capsule [Colace]		Other - Specific to initial possibly-pregnant cohort (633K)				X			
3045640	Choriogonadotropin [Units/volume] in Amniotic fluid		Other - Specific to initial possibly-pregnant cohort (633K)				X			
1146731	10 ML ephedrine sulfate 5 MG/ML Injection		Other - Specific to initial possibly-pregnant cohort (633K)				X			
37498150	copper 313 MG Drug Implant		Other - Specific to initial possibly-pregnant cohort (633K)				X			
43533776	Reagin and Treponema pallidum IgG and IgM [Interpretation] in Serum or Plasma		Other - Specific to initial possibly-pregnant cohort (633K)				X			
3019014	Varicella zoster virus Ab [Units/volume] in Serum		Other - Specific to initial possibly-pregnant cohort (633K)				X			
4312876	Lead measurement, quantitative, blood		Other - Specific to initial possibly-pregnant cohort (633K)				X			
42529203	Choriogonadotropin [Units/volume] in Serum or Plasma by Immunoassay		Other - Specific to initial possibly-pregnant cohort (633K)				X			
2102137	Insertion, non-biodegradable drug delivery implant		Other - Specific to initial possibly-pregnant cohort (633K)				X			
40764171	Cyclobenzaprine [Mass/volume] in Urine by Confirmatory method		Other - Specific to initial possibly-pregnant cohort (633K)				X			
42529226	Progesterone [Mass/volume] in Serum or Plasma by Immunoassay		Other - Specific to initial possibly-pregnant cohort (633K)				X			
3032002	Ethnic background Stated		Other - Specific to initial possibly-pregnant cohort (633K)				X			
3022440	Methylphenidate cutoff [Mass/volume] in Urine for Screen method		Other - Specific to initial possibly-pregnant cohort (633K)				X			
4086921	Situation with explicit context		Other - Specific to initial possibly-pregnant cohort (633K)				X			
3045765	ABO and Rh group [Type] in Cord blood		Other - Specific to initial possibly-pregnant cohort (633K)				X			
3030612	Hemoglobin S/Hemoglobin.total in Blood by Electrophoresis		Other - Specific to initial possibly-pregnant cohort (633K)				X			
3006949	Hemoglobin.other/Hemoglobin.total in Blood by Electrophoresis		Other - Specific to initial possibly-pregnant cohort (633K)				X			
40236648	clomiphene citrate 50 MG Oral Tablet		Other - Specific to initial possibly-pregnant cohort (633K)				X			

3013530	Hemoglobin E/Hemoglobin.total in Blood		Other - Specific to initial possibly-pregnant cohort (633K)				X			
19122554	simethicone 80 MG Chewable Tablet [Mi-Acid Gas Relief]		Other - Specific to initial possibly-pregnant cohort (633K)				X			
4094448	Pregnancy test negative		Other - Specific to initial possibly-pregnant cohort (633K)				X			
2110143	Colposcopy of the cervix including upper/adjacent vagina		Other - Specific to initial possibly-pregnant cohort (633K)				X			
4227745	Infertility study		Other - Specific to initial possibly-pregnant cohort (633K)				X			
42708535	hydrocortisone 25 MG/ML / pramoxine hydrochloride 10 MG/ML Rectal Cream		Other - Specific to initial possibly-pregnant cohort (633K)				X			
40766194	Treponema pallidum Ab [Units/volume] in Serum by Immunoassay		Other - Specific to initial possibly-pregnant cohort (633K)				X			
46221626	levonorgestrel 0.000813 MG/HR Intrauterine System [Liletta]		Other - Specific to initial possibly-pregnant cohort (633K)				X			
44506695	hydrocortisone 25 MG/ML Topical Cream [Proctozone HC]		Other - Specific to initial possibly-pregnant cohort (633K)				X			
42529221	Mullerian inhibiting substance [Mass/volume] in Serum or Plasma by Immunoassay		Other - Specific to initial possibly-pregnant cohort (633K)				X			
40226823	20 ML epinephrine 0.005 MG/ML / lidocaine hydrochloride 20 MG/ML Injection		Other - Specific to initial possibly-pregnant cohort (633K)				X			
2721264	Contraceptive pills for birth control		Other - Specific to initial possibly-pregnant cohort (633K)				X			
1717503	ampicillin 250 MG/ML Injectable Solution		Other - Specific to initial possibly-pregnant cohort (633K)				X			
4037495	Dilated cardiomyopathy secondary to peripartum heart disease		Other - Specific to initial possibly-pregnant cohort (633K)				X			
1360067	iron		Other - Specific to initial possibly-pregnant cohort (633K)				X			
3027529	Toxoplasma gondii IgM Ab [Units/volume] in Serum		Other - Specific to initial possibly-pregnant cohort (633K)				X			
2212918	Antibody; varicella-zoster		Other - Specific to initial possibly-pregnant cohort (633K)				X			
1518198	norelgestromin		Other - Specific to initial possibly-pregnant cohort (633K)				X			
45766058	Sterilization procedure		Other - Specific to initial possibly-pregnant cohort (633K)				X			
40240083	etonogestrel 68 MG Drug Implant [Nexplanon]		Other - Specific to initial possibly-pregnant cohort (633K)				X			
40038376	fentanyl Injectable Solution		Other - Specific to initial possibly-pregnant cohort (633K)				X			
1724667	dicloxacillin 250 MG Oral Capsule		Other - Specific to initial possibly-pregnant cohort (633K)				X			
42903067	morphine sulfate 10 MG/ML Prefilled Syringe		Other - Specific to initial possibly-pregnant cohort (633K)				X			
4049461	Congenital duplication of uterus		Other - Specific to initial possibly-pregnant cohort (633K)				X			
2110242	Laparoscopy, surgical; with fulguration of oviducts (with or without transection)		Other - Specific to initial possibly-pregnant cohort (633K)				X			
4251903	Family planning surveillance		Other - Specific to initial possibly-pregnant cohort (633K)				X			
941577	tetrahydrozoline		Other - Specific to initial possibly-pregnant cohort (633K)				X			
72245	Chlamydial infection of lower genitourinary tract		Other - Specific to initial possibly-pregnant cohort (633K)				X			
40169195	1 ML morphine sulfate 10 MG/ML Prefilled Syringe		Other - Specific to initial possibly-pregnant cohort (633K)				X			
967434	simethicone 80 MG Chewable Tablet		Other - Specific to initial possibly-pregnant cohort (633K)				X			
40224806	1 ML medroxyprogesterone acetate 150 MG/ML Injection [Depo-Provera]		Other - Specific to initial possibly-pregnant cohort (633K)				X			
3009678	Varicella zoster virus IgG Ab [Presence] in Serum by Immunoassay		Other - Specific to initial possibly-pregnant cohort (633K)				X			
927970	hydrocortisone acetate 10 MG/ML / pramoxine hydrochloride 10 MG/ML Rectal Foam		Other - Specific to initial possibly-pregnant cohort (633K)				X			
976245	hydrocortisone 25 MG/ML Rectal Cream [Procto-Kit]		Other - Specific to initial possibly-pregnant cohort (633K)				X			
19131658	0.5 ML measles virus vaccine live, Enders' attenuated Edmonston strain 2000 UNT/ML / mumps virus vaccine live, Jeryl Lynn strain 25000 UNT/ML / rubella virus vaccine live (Wistar RA 27-3 strain) 2000 UNT/ML Injection [M-M-R II]		Other - Specific to initial possibly-pregnant cohort (633K)				X			

40243340	atropine sulfate 0.025 MG / diphenoxylate hydrochloride 2.5 MG Oral Tablet		Other - Specific to initial possibly-pregnant cohort (633K)				X			
4135174	Contraception education		Other - Specific to initial possibly-pregnant cohort (633K)				X			
3047982	CT Upper extremity WO and W contrast IV		Other - Specific to initial possibly-pregnant cohort (633K)				X			
19128370	follicle stimulating hormone 75 UNT / luteinizing hormone 75 UNT Injection [Menopur]		Other - Specific to initial possibly-pregnant cohort (633K)				X			
19045303	magnesium hydroxide 80 MG/ML Oral Suspension [Phillips Milk of Magnesia]		Other - Specific to initial possibly-pregnant cohort (633K)				X			
42709176	simethicone 80 MG Chewable Tablet [Ez2go Zero]		Other - Specific to initial possibly-pregnant cohort (633K)				X			
4324621	Education about oral contraception		Other - Specific to initial possibly-pregnant cohort (633K)				X			
3019428	Reagin Ab [Presence] in Serum by VDRL		Other - Specific to initial possibly-pregnant cohort (633K)				X			
4237017	Genetic test		Other - Specific to initial possibly-pregnant cohort (633K)				X			
3022386	Varicella zoster virus IgG Ab [Presence] in Serum		Other - Specific to initial possibly-pregnant cohort (633K)				X			
201909	Female infertility		Other - Specific to initial possibly-pregnant cohort (633K)				X			
46221622	levonorgestrel 0.000813 MG/HR Intrauterine System		Other - Specific to initial possibly-pregnant cohort (633K)				X			
3026536	Glucose [Mass/volume] in Serum or Plasma --post meal		Other - Specific to initial possibly-pregnant cohort (633K)				X			
40761553	Hepatitis B virus surface Ag [Units/volume] in Serum		Other - Specific to initial possibly-pregnant cohort (633K)				X			
19097664	citrulline		Other - Specific to initial possibly-pregnant cohort (633K)				X			
2211766	Saline infusion sonohysterography (SIS), including color flow Doppler, when performed		Other - Specific to initial possibly-pregnant cohort (633K)				X			
3028572	Rubella virus Ab [Titer] in Serum		Other - Specific to initial possibly-pregnant cohort (633K)				X			
3011981	Treponema pallidum IgG Ab [Presence] in Serum		Other - Specific to initial possibly-pregnant cohort (633K)				X			
3044870	Hemoglobin C/Hemoglobin.total in Blood by Electrophoresis		Other - Specific to initial possibly-pregnant cohort (633K)				X			
46275284	1000 ML magnesium sulfate 40 MG/ML Injection		Other - Specific to initial possibly-pregnant cohort (633K)				X			
1387426	folate		Other - Specific to initial possibly-pregnant cohort (633K)				X			
40185204	miconazole nitrate 100 MG Vaginal Insert		Other - Specific to initial possibly-pregnant cohort (633K)				X			
197044	Female infertility associated with anovulation		Other - Specific to initial possibly-pregnant cohort (633K)				X			
2314183	Medical genetics and genetic counseling services, each 30 minutes face-to-face with patient/family		Other - Specific to initial possibly-pregnant cohort (633K)				X			
4228322	Neuraxial nerve block		Other - Specific to initial possibly-pregnant cohort (633K)				X			
4038490	Finding of fertility		Other - Specific to initial possibly-pregnant cohort (633K)				X			
3020457	Parvovirus B19 IgG Ab [Units/volume] in Serum by Immunoassay		Other - Specific to initial possibly-pregnant cohort (633K)				X			
1593933	10 ML epinephrine 0.005 MG/ML / lidocaine hydrochloride 20 MG/ML Injection		Other - Specific to initial possibly-pregnant cohort (633K)				X			
4047258	Dilation and curettage		Other - Specific to initial possibly-pregnant cohort (633K)				X			
40222687	hydroxyzine pamoate 50 MG Oral Capsule [Vistaril]		Other - Specific to initial possibly-pregnant cohort (633K)				X			
1593934	10 ML epinephrine 0.005 MG/ML / lidocaine hydrochloride 20 MG/ML Injection [Xylocaine with Epinephrine]		Other - Specific to initial possibly-pregnant cohort (633K)				X			
941522	terconazole 4 MG/ML Vaginal Cream [Zazole]		Other - Specific to initial possibly-pregnant cohort (633K)				X			
36032175	Neisseria gonorrhoeae DNA [Presence] in Cervix by NAA with probe detection		Other - Specific to initial possibly-pregnant cohort (633K)				X			
40169560	chorionic gonadotropin 10000 UNT/ML Injectable Solution		Other - Specific to initial possibly-pregnant cohort (633K)				X			
1552348	progesterone 200 MG Oral Capsule [Prometrium]		Other - Specific to initial possibly-pregnant cohort (633K)				X			
40758328	Cells karyotyped.total [#] in Blood or Tissue		Other - Specific to initial possibly-pregnant cohort (633K)				X			

19070743	cetrorelix 0.25 MG Injection		Other - Specific to initial possibly-pregnant cohort (633K)				X			
436164	Corpus luteum cyst		Other - Specific to initial possibly-pregnant cohort (633K)				X			
1536743	ganirelix		Other - Specific to initial possibly-pregnant cohort (633K)				X			
40162344	diclofenac sodium 50 MG / misoprostol 0.2 MG Delayed Release Oral Tablet		Other - Specific to initial possibly-pregnant cohort (633K)				X			
21491353	Hemoglobin pattern [Interpretation] in Blood by Capillary electrophoresis (CE)		Other - Specific to initial possibly-pregnant cohort (633K)				X			
4059978	Insertion of subcutaneous contraceptive		Other - Specific to initial possibly-pregnant cohort (633K)				X			
35606033	clonidine hydrochloride 0.1 MG/ML Injection		Other - Specific to initial possibly-pregnant cohort (633K)				X			
3020147	Parvovirus B19 IgM Ab [Units/volume] in Serum by Immunoassay		Other - Specific to initial possibly-pregnant cohort (633K)				X			
3042063	Carbon dioxide, total [Moles/volume] in Venous cord blood		Other - Specific to initial possibly-pregnant cohort (633K)				X			
1543003	follitropin alfa 600 UNT/ML Injectable Solution		Other - Specific to initial possibly-pregnant cohort (633K)				X			
3020428	Hemoglobin A/Hemoglobin.total in Blood		Other - Specific to initial possibly-pregnant cohort (633K)				X			
35605343	ampicillin 2000 MG Injection		Other - Specific to initial possibly-pregnant cohort (633K)				X			
35603814	2 ML bupivacaine hydrochloride 7.5 MG/ML Injection		Other - Specific to initial possibly-pregnant cohort (633K)				X			
42799739	glycerin 144 MG/ML / petrolatum 150 MG/ML / phenylephrine hydrochloride 2.5 MG/ML / pramoxine hydrochloride 10 MG/ML Rectal Cream		Other - Specific to initial possibly-pregnant cohort (633K)				X			
19127921	{28 (norethindrone 0.35 MG Oral Tablet) } Pack		Other - Specific to initial possibly-pregnant cohort (633K)				X			
2785975	Introduction of Serum, Toxoid and Vaccine into Subcutaneous Tissue, Percutaneous Approach		Other - Specific to initial possibly-pregnant cohort (633K)				X			
1353407	pyridoxine hydrochloride 100 MG/ML Injectable Solution		Other - Specific to initial possibly-pregnant cohort (633K)				X			
1724666	dicloxacillin		Other - Specific to initial possibly-pregnant cohort (633K)				X			
4258374	Reproductive care management		Other - Specific to initial possibly-pregnant cohort (633K)				X			
4196362	Genetic counseling		Other - Specific to initial possibly-pregnant cohort (633K)				X			
45777005	{28 (norethindrone 0.35 MG Oral Tablet) } Pack [Sharobel 28 Day]		Other - Specific to initial possibly-pregnant cohort (633K)				X			
19049909	potassium iodide		Other - Specific to initial possibly-pregnant cohort (633K)				X			
3018056	Glucose.PO [Mass] of Dose		Other - Specific to initial possibly-pregnant cohort (633K)				X			
1351622	leuprolide acetate 5 MG/ML Injectable Solution		Other - Specific to initial possibly-pregnant cohort (633K)				X			
4244530	Uterus arcuatus		Other - Specific to initial possibly-pregnant cohort (633K)				X			
19018419	niacinamide		Other - Specific to initial possibly-pregnant cohort (633K)				X			
19005046	pyridoxine		Other - Specific to initial possibly-pregnant cohort (633K)				X			
941472	terconazole		Other - Specific to initial possibly-pregnant cohort (633K)				X			
2110199	Catheterization and introduction of saline or contrast material for saline infusion sonohysterography (SIS) or hysterosalpingography		Other - Specific to initial possibly-pregnant cohort (633K)				X			
3020784	Hemoglobin A2/Hemoglobin.total in Blood		Other - Specific to initial possibly-pregnant cohort (633K)				X			
709817	Syphilis test, non-treponemal antibody, immunoassay, qualitative (RPR)		Other - Specific to initial possibly-pregnant cohort (633K)				X			
35603820	2 ML bupivacaine hydrochloride 7.5 MG/ML Injection [Marcaine]		Other - Specific to initial possibly-pregnant cohort (633K)				X			
3051396	Hemoglobin.other/Hemoglobin.total in Blood		Other - Specific to initial possibly-pregnant cohort (633K)				X			
196162	Inflammatory disease of the uterus		Other - Specific to initial possibly-pregnant cohort (633K)				X			
3033053	Referral lab test method		Other - Specific to initial possibly-pregnant cohort (633K)				X			
45776016	1.5 ML follitropin alfa 600 UNT/ML Pen Injector [Gonal F]		Other - Specific to initial possibly-pregnant cohort (633K)				X			

3015378	Treponema pallidum Ab [Units/volume] in Serum		Other - Specific to initial possibly-pregnant cohort (633K)				X			
42742305	FMR1 (fragile X mental retardation 1) (eg, fragile X mental retardation) gene analysis; evaluation to detect abnormal (eg, expanded) alleles		Other - Specific to initial possibly-pregnant cohort (633K)				X			
4168318	Genetic disorder carrier		Other - Specific to initial possibly-pregnant cohort (633K)				X			
46235159	Fetal Trisomy 13 prior risk [Likelihood] Based on maternal age		Other - Specific to initial possibly-pregnant cohort (633K)				X			
4098887	Fertility problem		Other - Specific to initial possibly-pregnant cohort (633K)				X			
3016999	Rubella virus IgG Ab [Units/volume] in Serum		Other - Specific to initial possibly-pregnant cohort (633K)				X			
42800417	ammonia		Other - Specific to initial possibly-pregnant cohort (633K)				X			
3011172	Rubella virus IgM Ab [Units/volume] in Serum by Immunoassay		Other - Specific to initial possibly-pregnant cohort (633K)				X			
438685	Arbovirus infection		Other - Specific to initial possibly-pregnant cohort (633K)				X			
36878782	Multivitamin preparation		Other - Specific to initial possibly-pregnant cohort (633K)				X			
37498155	copper 313 MG Drug Implant [ParaGard]		Other - Specific to initial possibly-pregnant cohort (633K)				X			
1593571	30 ML mepivacaine hydrochloride 10 MG/ML Injection		Other - Specific to initial possibly-pregnant cohort (633K)				X			
2213358	Sperm isolation; complex prep (eg, Percoll gradient, albumin gradient) for insemination or diagnosis with semen analysis		Other - Specific to initial possibly-pregnant cohort (633K)				X			
3005081	Hemoglobin S/Hemoglobin.total in Blood		Other - Specific to initial possibly-pregnant cohort (633K)				X			
35604841	10 ML morphine sulfate 1 MG/ML Injection		Other - Specific to initial possibly-pregnant cohort (633K)				X			
19112868	citric acid 60 MG/ML / sodium citrate 100 MG/ML Oral Solution		Other - Specific to initial possibly-pregnant cohort (633K)				X			
1595799	ferrous fumarate		Other - Specific to initial possibly-pregnant cohort (633K)				X			
3029071	Hemoglobin F/Hemoglobin.total in Blood by HPLC		Other - Specific to initial possibly-pregnant cohort (633K)				X			
40175253	nalbuphine hydrochloride 20 MG/ML Injectable Solution		Other - Specific to initial possibly-pregnant cohort (633K)				X			
4040738	Reproductive finding		Other - Specific to initial possibly-pregnant cohort (633K)				X			
40162447	0.5 ML ganirelix acetate 0.5 MG/ML Prefilled Syringe		Other - Specific to initial possibly-pregnant cohort (633K)				X			
19127159	0.5 ML choriogonadotropin alfa 0.5 MG/ML Prefilled Syringe [Ovidrel]		Other - Specific to initial possibly-pregnant cohort (633K)				X			
3018738	Hemoglobin F/Hemoglobin.total in Blood		Other - Specific to initial possibly-pregnant cohort (633K)				X			
4150816	Bicornuate uterus		Other - Specific to initial possibly-pregnant cohort (633K)				X			
19043701	simethicone 80 MG Chewable Tablet [Anti-Gas-80]		Other - Specific to initial possibly-pregnant cohort (633K)				X			
19080159	follicle stimulating hormone 75 UNT / luteinizing hormone 75 UNT Injection		Other - Specific to initial possibly-pregnant cohort (633K)				X			
3015697	Hemoglobin pattern [Interpretation] in Blood		Other - Specific to initial possibly-pregnant cohort (633K)				X			
2785755	Introduction of Anesthetic Agent into Spinal Canal, Percutaneous Approach		Other - Specific to initial possibly-pregnant cohort (633K)				X			
3053000	Hemoglobin pattern [Interpretation] in Blood by Electrophoresis		Other - Specific to initial possibly-pregnant cohort (633K)				X			
19003263	Metamucil		Other - Specific to initial possibly-pregnant cohort (633K)				X			
42742371	Molecular pathology procedure, Level 2		Other - Specific to initial possibly-pregnant cohort (633K)				X			
40162395	1.08 ML follitropin beta 833 UNT/ML Cartridge		Other - Specific to initial possibly-pregnant cohort (633K)				X			
19095115	mineral oil 1000 MG/ML Oral Solution		Other - Specific to initial possibly-pregnant cohort (633K)				X			
3011564	Rubella virus IgG Ab [Units/volume] in Serum or Plasma by Immunoassay		Other - Specific to initial possibly-pregnant cohort (633K)				X			
40221409	{28 (norethindrone 0.35 MG Oral Tablet) } Pack [Heather 28 Day]		Other - Specific to initial possibly-pregnant cohort (633K)				X			
709816	Antibody, Treponema pallidum, total and rapid plasma reagin (RPR), immunoassay, qualitative		Other - Specific to initial possibly-pregnant cohort (633K)				X			

2212388	Hemoglobin fractionation and quantitation; chromatography (eg, A2, S, C, and/or F)		Other - Specific to initial possibly-pregnant cohort (633K)				X			
3031412	Hemoglobin.other/Hemoglobin.total in Blood by HPLC		Other - Specific to initial possibly-pregnant cohort (633K)				X			
3037653	Hemoglobin A2/Hemoglobin.total in Blood by HPLC		Other - Specific to initial possibly-pregnant cohort (633K)				X			
197927	Intrauterine synechiae		Other - Specific to initial possibly-pregnant cohort (633K)				X			
2211565	Hysterosalpingography, radiological supervision and interpretation		Other - Specific to initial possibly-pregnant cohort (633K)				X			
3018759	Base deficit in Venous cord blood		Other - Specific to initial possibly-pregnant cohort (633K)				X			
3017572	Hemoglobin A2 [Presence] in Blood		Other - Specific to initial possibly-pregnant cohort (633K)				X			
3000873	Hemoglobin F [Presence] in Blood by Alkali denaturation		Other - Specific to initial possibly-pregnant cohort (633K)				X			
19127922	{28 (norethindrone 0.35 MG Oral Tablet) } Pack [Camila 28 Day]		Other - Specific to initial possibly-pregnant cohort (633K)				X			
4005743	Female sterility		Other - Specific to initial possibly-pregnant cohort (633K)				X			
42708683	docusate sodium 100 MG Oral Capsule [Dulcolax Stool Softener]		Other - Specific to initial possibly-pregnant cohort (633K)				X			
3046853	Race		Other - Specific to initial possibly-pregnant cohort (633K)				X			
19124609	etonogestrel 68 MG Drug Implant [Implanon]		Other - Specific to initial possibly-pregnant cohort (633K)				X			
3040340	Leukocytes [Presence] in Urine by Visual		Other - Specific to initial possibly-pregnant cohort (633K)				X			
42873527	docusate sodium 100 MG Oral Capsule [Doculase]		Other - Specific to initial possibly-pregnant cohort (633K)				X			
42708709	docusate sodium 100 MG Oral Capsule [Phillips Stool Softener]		Other - Specific to initial possibly-pregnant cohort (633K)				X			
19013437	estradiol 2 MG Oral Tablet [Elleste-Solo]		Other - Specific to initial possibly-pregnant cohort (633K)				X			
3045405	Hemoglobin F/Hemoglobin.total in Blood by Electrophoresis		Other - Specific to initial possibly-pregnant cohort (633K)				X			
3049192	Hemoglobin pattern [Interpretation] in Blood by Electrophesis citrate agar		Other - Specific to initial possibly-pregnant cohort (633K)				X			
3031973	Hemoglobin A/Hemoglobin.total in Blood by HPLC		Other - Specific to initial possibly-pregnant cohort (633K)				X			
4025367	Genetic finding		Other - Specific to initial possibly-pregnant cohort (633K)				X			
3001258	Hemoglobin C/Hemoglobin.total in Blood		Other - Specific to initial possibly-pregnant cohort (633K)				X			
1728416	penicillin G		Other - Specific to initial possibly-pregnant cohort (633K)				X			
43021968	History of gestational diabetes mellitus		Other - Specific to initial possibly-pregnant cohort (633K)				X			
4051702	FH: Congenital anomaly		Other - Specific to initial possibly-pregnant cohort (633K)				X			
3010937	Rubella virus Ab [Presence] in Serum		Other - Specific to initial possibly-pregnant cohort (633K)				X			
19083910	Adacel		Other - Specific to initial possibly-pregnant cohort (633K)				X			
4250175	Pregnancy detection examination		Other - Specific to initial possibly-pregnant cohort (633K)				X			
42708386	docusate calcium 240 MG Oral Capsule		Other - Specific to initial possibly-pregnant cohort (633K)				X			
42529190	Alpha-1-Fetoprotein [Mass/volume] in Serum or Plasma by Immunoassay		Other - Specific to initial possibly-pregnant cohort (633K)				X			
1593923	epinephrine 0.005 MG/ML / lidocaine hydrochloride 20 MG/ML Injection		Other - Specific to initial possibly-pregnant cohort (633K)				X			
2211786	Ultrasonic guidance for aspiration of ova, imaging supervision and interpretation		Other - Specific to initial possibly-pregnant cohort (633K)				X			
3015745	Hemoglobin F [Presence] in Blood		Other - Specific to initial possibly-pregnant cohort (633K)				X			
1724668	dicloxacillin 500 MG Oral Capsule		Other - Specific to initial possibly-pregnant cohort (633K)				X			
43055688	Chlamydia trachomatis and Neisseria gonorrhoeae DNA panel - Specimen		Other - Specific to initial possibly-pregnant cohort (633K)				X			
1593601	1 ML nalbuphine hydrochloride 20 MG/ML Injection		Other - Specific to initial possibly-pregnant cohort (633K)				X			

4079757	Treatment given		Other - Specific to initial possibly-pregnant cohort (633K)				X			
43532913	{28 (norethindrone 0.35 MG Oral Tablet) } Pack [Lyza]		Other - Specific to initial possibly-pregnant cohort (633K)				X			
3033811	Base deficit in Arterial cord blood		Other - Specific to initial possibly-pregnant cohort (633K)				X			
3021009	Hemoglobin A2/Hemoglobin.total in Blood by Electrophoresis		Other - Specific to initial possibly-pregnant cohort (633K)				X			
437221	Chlamydia trachomatis infection		Other - Specific to initial possibly-pregnant cohort (633K)				X			
3050252	CFTR gene targeted mutation analysis in Blood or Tissue by Molecular genetics method		Other - Specific to initial possibly-pregnant cohort (633K)				X			
927478	pramoxine		Other - Specific to initial possibly-pregnant cohort (633K)				X			
3000061	Hemoglobin F [Presence] in Blood by Kleihauer-Betke method		Other - Specific to initial possibly-pregnant cohort (633K)				X			
19019339	norethindrone 0.35 MG Oral Tablet		Other - Specific to initial possibly-pregnant cohort (633K)				X			
4171113	Fetal growth abnormality		Other - Specific to initial possibly-pregnant cohort (633K)				X			
19026717	pyridoxine hydrochloride		Other - Specific to initial possibly-pregnant cohort (633K)				X			
3045429	Collection date of Specimen		Other - Specific to initial possibly-pregnant cohort (633K)				X			
40238937	pyridoxine hydrochloride 25 MG Oral Tablet		Other - Specific to initial possibly-pregnant cohort (633K)				X			
3044997	Hemoglobin A [Presence] in Blood by Electrophoresis		Other - Specific to initial possibly-pregnant cohort (633K)				X			
4194396	Hemoglobinopathy screening test		Other - Specific to initial possibly-pregnant cohort (633K)				X			
2213357	Sperm isolation; simple prep (eg, sperm wash and swim-up) for insemination or diagnosis with semen analysis		Other - Specific to initial possibly-pregnant cohort (633K)				X			
2723345	Insertion of Infusion Device into Spinal Canal, Percutaneous Approach		Other - Specific to initial possibly-pregnant cohort (633K)				X			
37021509	Streptococcus agalactiae DNA [Presence] by NAA with probe detection in Positive blood culture		Other - Specific to initial possibly-pregnant cohort (633K)				X			
35604842	10 ML morphine sulfate 1 MG/ML Injection [Astramorph]		Other - Specific to initial possibly-pregnant cohort (633K)				X			
923876	dibucaine 0.01 MG/MG Rectal Ointment		Other - Specific to initial possibly-pregnant cohort (633K)				X			
4143698	Streptococcus agalactiae infection		Other - Specific to initial possibly-pregnant cohort (633K)				X			
40226824	20 ML epinephrine 0.005 MG/ML / lidocaine hydrochloride 20 MG/ML Injection [Xylocaine with Epinephrine]		Other - Specific to initial possibly-pregnant cohort (633K)				X			
3052922	Fibronectin.fetal [Presence] in Specimen		Other - Specific to initial possibly-pregnant cohort (633K)				X			
1359143	cinnamon bark		Other - Specific to initial possibly-pregnant cohort (633K)				X			
2212475	Molecular diagnostics; isolation or extraction of highly purified nucleic acid, each nucleic acid type (ie, DNA or RNA)		Other - Specific to initial possibly-pregnant cohort (633K)				X			
19127158	0.5 ML choriogonadotropin alfa 0.5 MG/ML Prefilled Syringe		Other - Specific to initial possibly-pregnant cohort (633K)				X			
3035686	CFTR gene mutations tested for in Blood or Tissue by Molecular genetics method Nominal		Other - Specific to initial possibly-pregnant cohort (633K)				X			
19019309	nifedipine 10 MG Oral Capsule		Other - Specific to initial possibly-pregnant cohort (633K)				X			
3013853	Body weight Measured --ante partum		Other - Specific to initial possibly-pregnant cohort (633K)				X			
3035792	Gene XXX targeted mutation analysis in Blood or Tissue by Molecular genetics method		Other - Specific to initial possibly-pregnant cohort (633K)				X			
3032260	Hemoglobin pattern [Interpretation] in Blood by HPLC Narrative		Other - Specific to initial possibly-pregnant cohort (633K)				X			
950435	citric acid		Other - Specific to initial possibly-pregnant cohort (633K)				X			
3043953	Rubella virus IgG Ab [Presence] in Serum or Plasma by Immunoassay		Other - Specific to initial possibly-pregnant cohort (633K)				X			
19131656	0.5 ML measles virus vaccine live, Enders' attenuated Edmonston strain 2000 UNT/ML / mumps virus vaccine live, Jeryl Lynn strain 25000 UNT/ML / rubella virus vaccine live (Wistar RA 27-3 strain) 2000 UNT/ML Injection		Other - Specific to initial possibly-pregnant cohort (633K)				X			
36249485	chloroprocaine Injection		Other - Specific to initial possibly-pregnant cohort (633K)				X			

44786642	Hemoglobin.other [Type] in Blood		Other - Specific to initial possibly-pregnant cohort (633K)				X			
3009131	Hemoglobin A/Hemoglobin.total in Blood by Electrophoresis		Other - Specific to initial possibly-pregnant cohort (633K)				X			
2212387	Hemoglobin fractionation and quantitation; electrophoresis (eg, A2, S, C, and/or F)		Other - Specific to initial possibly-pregnant cohort (633K)				X			
3032358	Hemoglobin A2/Hemoglobin.total in Blood by Chromatography column		Other - Specific to initial possibly-pregnant cohort (633K)				X			
3018595	Inhibin A [Mass/volume] in Serum or Plasma		Other - Specific to initial possibly-pregnant cohort (633K)				X			
1552382	progesterone 50 MG/ML Injectable Solution		Other - Specific to initial possibly-pregnant cohort (633K)				X			
43055234	pH of Vaginal fluid by Test strip		Other - Specific to initial possibly-pregnant cohort (633K)				X			
738818	doxylamine		Other - Specific to initial possibly-pregnant cohort (633K)				X			
35603825	2 ML bupivacaine hydrochloride 7.5 MG/ML Injection [Sensorcaine]		Other - Specific to initial possibly-pregnant cohort (633K)				X			
3010483	D Ag [Presence] in Blood		Other - Specific to initial possibly-pregnant cohort (633K)				X			
3021650	Chenodeoxycholate [Moles/volume] in Serum or Plasma		Other - Specific to initial possibly-pregnant cohort (633K)				X			
2212909	Antibody; rubella		Other - Specific to initial possibly-pregnant cohort (633K)				X			
3013750	Rubella virus IgG Ab [Presence] in Serum		Other - Specific to initial possibly-pregnant cohort (633K)				X			
2212492	Molecular diagnostics; interpretation and report		Other - Specific to initial possibly-pregnant cohort (633K)				X			
40765103	Colonies counted [#]		Other - Specific to initial possibly-pregnant cohort (633K)				X			
1353265	vitamin B6 25 MG Oral Tablet		Other - Specific to initial possibly-pregnant cohort (633K)				X			
4266168	Missed period		Other - Specific to initial possibly-pregnant cohort (633K)				X			
19017494	progesterone 0.08 MG/MG Vaginal Gel [Crinone]		Other - Specific to initial possibly-pregnant cohort (633K)				X			
40168604	butorphanol tartrate 2 MG/ML Injectable Solution		Other - Specific to initial possibly-pregnant cohort (633K)				X			
927971	pramoxine hydrochloride 10 MG/ML Rectal Foam		Other - Specific to initial possibly-pregnant cohort (633K)				X			
4159647	Thromboembolic disorder		Other - Specific to initial possibly-pregnant cohort (633K)				X			
2213376	Storage (per year); embryo(s)		Other - Specific to initial possibly-pregnant cohort (633K)				X			
3032190	Trisomy 21 risk cutoff in Fetus		Other - Specific to initial possibly-pregnant cohort (633K)				X			
3004959	Base excess in Arterial cord blood by calculation		Other - Specific to initial possibly-pregnant cohort (633K)				X			
3028557	Deoxycholate [Moles/volume] in Serum or Plasma		Other - Specific to initial possibly-pregnant cohort (633K)				X			
19036443	nifedipine 10 MG Oral Capsule [Procardia]		Other - Specific to initial possibly-pregnant cohort (633K)				X			
3019641	Cholate [Moles/volume] in Serum or Plasma		Other - Specific to initial possibly-pregnant cohort (633K)				X			
3019398	CFTR gene mutations found [Identifier] in Blood or Tissue by Molecular genetics method Nominal		Other - Specific to initial possibly-pregnant cohort (633K)				X			
4080395	Blood group O Rh(D) positive		Other - Specific to initial possibly-pregnant cohort (633K)				X			
3022281	Body weight Measured --pre pregnancy		Other - Specific to initial possibly-pregnant cohort (633K)				X			
3035795	FMR1 gene targeted mutation analysis in Blood or Tissue by Molecular genetics method		Other - Specific to initial possibly-pregnant cohort (633K)				X			
40169885	10 ML morphine sulfate 1 MG/ML Injection [Duramorph]		Other - Specific to initial possibly-pregnant cohort (633K)				X			
40239109	doxylamine succinate 25 MG Oral Tablet		Other - Specific to initial possibly-pregnant cohort (633K)				X			
2213355	Cryopreservation; embryo(s)		Other - Specific to initial possibly-pregnant cohort (633K)				X			
4175555	Blood group B Rh(D) positive		Other - Specific to initial possibly-pregnant cohort (633K)				X			
2110198	Sperm washing for artificial insemination		Other - Specific to initial possibly-pregnant cohort (633K)				X			

3052868	Fetal Trisomy 18 risk [Likelihood] Based on maternal age		Other - Specific to initial possibly-pregnant cohort (633K)				X			
19020365	dibucaine 0.01 MG/MG Topical Ointment		Other - Specific to initial possibly-pregnant cohort (633K)				X			
42901710	alpha-tocopherol acetate 30 UNT / ascorbic acid 100 MG / beta carotene 1000 UNT / calcium carbonate 200 MG / calcium pantothenate 7 MG / cholecalciferol 400 UNT / docusate sodium 25 MG / ferrous fumarate 29 MG / folic acid 1 MG / niacinamide 15 MG / py...		Other - Specific to initial possibly-pregnant cohort (633K)				X			
19114108	citric acid 66.8 MG/ML / sodium citrate 100 MG/ML Oral Solution [Cytra 2]		Other - Specific to initial possibly-pregnant cohort (633K)				X			
1318988	nifedipine 10 MG Oral Capsule [Adalat]		Other - Specific to initial possibly-pregnant cohort (633K)				X			
3022304	Age		Other - Specific to initial possibly-pregnant cohort (633K)				X			
4082948	Blood group A Rh(D) positive		Other - Specific to initial possibly-pregnant cohort (633K)				X			
42870288	I have blamed myself unnecessarily when things went wrong in the past 7 days [EPDS]		Other - Specific to initial possibly-pregnant cohort (633K)				X			
35604834	morphine sulfate 0.5 MG/ML Injection		Other - Specific to initial possibly-pregnant cohort (633K)				X			
19067887	vitamin B6 25 MG Oral Tablet [Vitelle Nestrex]		Other - Specific to initial possibly-pregnant cohort (633K)				X			
45757490	Pregnancy not yet confirmed		Other - Specific to initial possibly-pregnant cohort (633K)				X			
46235341	Citation [Bibliographic Citation] in Referral lab test Narrative		Other - Specific to initial possibly-pregnant cohort (633K)				X			
3014482	Hemoglobin pattern [Interpretation] in Blood by Electrophoresis Narrative		Other - Specific to initial possibly-pregnant cohort (633K)				X			
3022314	Glucose [Mass/volume] in Urine --2 hours post dose glucose		Other - Specific to initial possibly-pregnant cohort (633K)				X			
19127047	progesterone 100 MG Vaginal Insert		Other - Specific to initial possibly-pregnant cohort (633K)				X			
42870293	I have felt sad or miserable in the past 7 days [EPDS]		Other - Specific to initial possibly-pregnant cohort (633K)				X			
19127049	progesterone 100 MG Vaginal Insert [Endometrin]		Other - Specific to initial possibly-pregnant cohort (633K)				X			
3028110	Bile acid [Moles/volume] in Serum or Plasma		Other - Specific to initial possibly-pregnant cohort (633K)				X			
1114122	nalbuphine		Other - Specific to initial possibly-pregnant cohort (633K)				X			
40758289	Ursodeoxycholate [Moles/volume] in Serum or Plasma		Other - Specific to initial possibly-pregnant cohort (633K)				X			
40239110	doxylamine succinate 25 MG Oral Tablet [Unisom]		Other - Specific to initial possibly-pregnant cohort (633K)				X			
40175254	nalbuphine hydrochloride 20 MG/ML Injectable Solution [Nubain]		Other - Specific to initial possibly-pregnant cohort (633K)				X			
42870295	The thought of harming myself has occurred to me in the past 7 days [EPDS]		Other - Specific to initial possibly-pregnant cohort (633K)				X			
19060393	quercetin		Other - Specific to initial possibly-pregnant cohort (633K)				X			
40479565	Carrier of cystic fibrosis gene mutation		Other - Specific to initial possibly-pregnant cohort (633K)				X			
40169879	10 ML morphine sulfate 0.5 MG/ML Injection [Duramorph]		Other - Specific to initial possibly-pregnant cohort (633K)				X			
3032863	Rubella virus IgG Ab [Titer] in Serum		Other - Specific to initial possibly-pregnant cohort (633K)				X			
19073229	cupric oxide		Other - Specific to initial possibly-pregnant cohort (633K)				X			
1593917	epinephrine 0.005 MG/ML / lidocaine hydrochloride 15 MG/ML Injection [Xylocaine with Epinephrine]		Other - Specific to initial possibly-pregnant cohort (633K)				X			
42628031	Obstetric panel (includes HIV testing) This panel must include the following: Blood count, complete (CBC), and automated differential WBC count (85025 or 85027 and 85004) OR Blood count, complete (CBC), automated (85027) and appropriate manual differential WBC count (85007 or 85009) Hepatitis B surface antigen (HBsAg) (87340) HIV-1 antigen(s), with HIV-1 and HIV-2 antibodies, single result (87389) Antibody, rubella (86762) Syphilis test, non-treponemal antibody; qualitative (eg, VDRL, RPR, ART) (86592) Antibody screen, RBC, each serum technique (86850) Blood typing, ABO (86900) AND Blood typing, Rh (D) (86901)		Other - Specific to initial possibly-pregnant cohort (633K)				X			
3004624	Choriogonadotropin.intact [Presence] in Serum or Plasma		Other - Specific to initial possibly-pregnant cohort (633K)				X			
3039361	Rubella virus IgG Ab avidity [Ratio] in Serum by Immunoassay		Other - Specific to initial possibly-pregnant cohort (633K)				X			

2101014	Anesthesia for intraperitoneal procedures in lower abdomen including laparoscopy; tubal ligation/transection		Other - Specific to initial possibly-pregnant cohort (633K)				X			
42707635	hydrocortisone acetate 10 MG/ML / pramoxine hydrochloride 10 MG/ML Rectal Cream		Other - Specific to initial possibly-pregnant cohort (633K)				X			
4239471	Postpartum depression		Other - Specific to initial possibly-pregnant cohort (633K)				X			
4011615	Recurrent miscarriage		Other - Specific to initial possibly-pregnant cohort (633K)				X			
1379525	inositol		Other - Specific to initial possibly-pregnant cohort (633K)				X			
19056802	alpha tocopherol		Other - Specific to initial possibly-pregnant cohort (633K)				X			
1593921	5 ML epinephrine 0.005 MG/ML / lidocaine hydrochloride 15 MG/ML Injection [Xylocaine with Epinephrine]		Other - Specific to initial possibly-pregnant cohort (633K)				X			
3035779	Inhibin A [Multiple of the median] adjusted in Serum or Plasma		Other - Specific to initial possibly-pregnant cohort (633K)				X			
4124081	Under care of pediatrician		Other - Specific to initial possibly-pregnant cohort (633K)				X			
4217899	Carrier detection, molecular genetics		Other - Specific to initial possibly-pregnant cohort (633K)				X			
1552383	progesterone 0.08 MG/MG Vaginal Gel		Other - Specific to initial possibly-pregnant cohort (633K)				X			
4024004	Female reproductive finding		Other - Specific to initial possibly-pregnant cohort (633K)				X			
3042975	Streptococcus sp identified in Isolate by Organism specific culture		Other - Specific to initial possibly-pregnant cohort (633K)				X			
4273323	Rubella		Other - Specific to initial possibly-pregnant cohort (633K)				X			
46235475	Fetal sex in Plasma cell-free DNA by Dosage of chromosome specific cell free (cf) DNA		Other - Specific to initial possibly-pregnant cohort (633K)				X			
3005044	Rubella virus IgG Ab [Interpretation] in Serum		Other - Specific to initial possibly-pregnant cohort (633K)				X			
3049207	Fetal Trisomy 21 risk [Likelihood] Based on maternal age		Other - Specific to initial possibly-pregnant cohort (633K)				X			
40168605	butorphanol tartrate 2 MG/ML Injectable Solution [Stadol]		Other - Specific to initial possibly-pregnant cohort (633K)				X			
37109077	Exposure to Zika virus		Other - Specific to initial possibly-pregnant cohort (633K)				X			
4129550	Unexplained infertility		Other - Specific to initial possibly-pregnant cohort (633K)				X			
46235242	Fetal Trisomy 18 risk [Interpretation] based on Plasma cell-free+WBC DNA by Dosage of chromosome-specific cfDNA Qualitative		Other - Specific to initial possibly-pregnant cohort (633K)				X			
3045991	Gestational age of fetus by Amniocentesis		Other - Specific to initial possibly-pregnant cohort (633K)				X			
432677	History of recurrent miscarriage - not pregnant		Other - Specific to initial possibly-pregnant cohort (633K)				X			
442781	Disorder of uterine cervix		Other - Specific to initial possibly-pregnant cohort (633K)				X			
2778636	Repair Vagina, Via Natural or Artificial Opening		Other - Specific to initial possibly-pregnant cohort (633K)				X			
40178991	20 ML chloroprocaine hydrochloride 30 MG/ML Injection [Nesacaine]		Other - Specific to initial possibly-pregnant cohort (633K)				X			
40168593	1 ML butorphanol tartrate 1 MG/ML Injection		Other - Specific to initial possibly-pregnant cohort (633K)				X			
19045810	dibucaine 0.01 MG/MG Topical Ointment [Rectacaine]		Other - Specific to initial possibly-pregnant cohort (633K)				X			
2213254	Tissue culture for non-neoplastic disorders; skin or other solid tissue biopsy		Other - Specific to initial possibly-pregnant cohort (633K)				X			
535714	Rho(D) immune globulin		Other - Specific to initial possibly-pregnant cohort (633K)				X			
440922	Diabetic on insulin		Other - Specific to initial possibly-pregnant cohort (633K)				X			
3035794	SMN1 gene targeted mutation analysis in Blood or Tissue by Molecular genetics method		Other - Specific to initial possibly-pregnant cohort (633K)				X			
1593599	1 ML nalbuphine hydrochloride 10 MG/ML Injection		Other - Specific to initial possibly-pregnant cohort (633K)				X			
19003319	misoprostol 0.2 MG Oral Tablet [Cytotec]		Other - Specific to initial possibly-pregnant cohort (633K)				X			
40065007	mineral oil Oral Solution		Other - Specific to initial possibly-pregnant cohort (633K)				X			
1593920	5 ML epinephrine 0.005 MG/ML / lidocaine hydrochloride 15 MG/ML Injection		Other - Specific to initial possibly-pregnant cohort (633K)				X			

19114106	citric acid 66.8 MG/ML / sodium citrate 100 MG/ML Oral Solution		Other - Specific to initial possibly-pregnant cohort (633K)				X			
1593916	epinephrine 0.005 MG/ML / lidocaine hydrochloride 15 MG/ML Injection		Other - Specific to initial possibly-pregnant cohort (633K)				X			
40757580	SMN2 gene targeted mutation analysis in Blood or Tissue by Molecular genetics method		Other - Specific to initial possibly-pregnant cohort (633K)				X			
19049410	chloroprocaine		Other - Specific to initial possibly-pregnant cohort (633K)				X			
2780875	Insertion of Contraceptive Device into Uterus, Via Natural or Artificial Opening		Other - Specific to initial possibly-pregnant cohort (633K)				X			
42628693	Injection(s), including indwelling catheter placement, continuous infusion or intermittent bolus, of diagnostic or therapeutic substance(s) (eg, anesthetic, antispasmodic, opioid, steroid, other solution), not including neurolytic substances, interlaminar epidural or subarachnoid, lumbar or sacral (caudal); without imaging guidance		Other - Specific to initial possibly-pregnant cohort (633K)				X			
35604832	10 ML morphine sulfate 0.5 MG/ML Injection		Other - Specific to initial possibly-pregnant cohort (633K)				X			
928002	pramoxine hydrochloride 10 MG/ML Rectal Foam [Proctofoam]		Other - Specific to initial possibly-pregnant cohort (633K)				X			
45774525	witch hazel 200 MG/ML Medicated Pad		Other - Specific to initial possibly-pregnant cohort (633K)				X			
3045859	Age at delivery		Other - Specific to initial possibly-pregnant cohort (633K)				X			
3007191	Age - Reported		Other - Specific to initial possibly-pregnant cohort (633K)				X			
42629302	tranexamic acid Injection		Other - Specific to initial possibly-pregnant cohort (633K)				X			
4316372	HELLP syndrome		Other - Specific to initial possibly-pregnant cohort (633K)				X			
43526401	doxylamine succinate 10 MG / pyridoxine hydrochloride 10 MG Delayed Release Oral Tablet		Other - Specific to initial possibly-pregnant cohort (633K)				X			
2212224	Bile acids; total		Other - Specific to initial possibly-pregnant cohort (633K)				X			
19099840	benzocaine 200 MG/ML Topical Spray		Other - Specific to initial possibly-pregnant cohort (633K)				X			
40175250	nalbuphine hydrochloride 10 MG/ML Injectable Solution [Nubain]		Other - Specific to initial possibly-pregnant cohort (633K)				X			
45757142	Viral hepatitis in mother complicating pregnancy		Other - Specific to initial possibly-pregnant cohort (633K)				X			
4268045	Arterial anomaly of umbilical cord		Other - Specific to initial possibly-pregnant cohort (633K)				X			
764109	History of complication of pregnancy, childbirth and/or puerperium		Other - Specific to initial possibly-pregnant cohort (633K)				X			
19128547	citric acid 66.8 MG/ML / sodium citrate 100 MG/ML Oral Solution [Polycitra]		Other - Specific to initial possibly-pregnant cohort (633K)				X			
40224726	doconexent		Other - Specific to initial possibly-pregnant cohort (633K)				X			
40771968	Mother's body weight --at delivery		Other - Specific to initial possibly-pregnant cohort (633K)				X			
3017885	Carboxy tetrahydrocannabinol [Presence] in Urine by Screen method		Other - Specific to initial possibly-pregnant cohort (633K)				X			
2213381	Thawing of cryopreserved; embryo(s)		Other - Specific to initial possibly-pregnant cohort (633K)				X			
3041037	FMR1 gene mutations found [Identifier] in Blood or Tissue by Molecular genetics method Nominal		Other - Specific to initial possibly-pregnant cohort (633K)				X			
2786230	Introduction of Serum, Toxoid and Vaccine into Peripheral Vein, Percutaneous Approach		Other - Specific to initial possibly-pregnant cohort (633K)				X			
3031330	Alpha-1-Microglobulin.placental [Presence] in Vaginal fluid		Other - Specific to initial possibly-pregnant cohort (633K)				X			
1593914	10 ML epinephrine 0.005 MG/ML / lidocaine hydrochloride 15 MG/ML Injection		Other - Specific to initial possibly-pregnant cohort (633K)				X			
3013826	Glucose [Moles/volume] in Serum or Plasma		Other - Specific to initial possibly-pregnant cohort (633K)				X			
3014295	Oxygen saturation in Arterial cord blood		Other - Specific to initial possibly-pregnant cohort (633K)				X			
43021845	Psychosocial problems related to unwanted pregnancy		Other - Specific to initial possibly-pregnant cohort (633K)				X			
42742292	CFTR (cystic fibrosis transmembrane conductance regulator) (eg, cystic fibrosis) gene analysis; common variants (eg, ACMG/ACOG guidelines)		Other - Specific to initial possibly-pregnant cohort (633K)				X			
959196	witch hazel		Other - Specific to initial possibly-pregnant cohort (633K)				X			

36715476	Human immunodeficiency virus complicating pregnancy childbirth and the puerperium		Other - Specific to initial possibly-pregnant cohort (633K)				X			
40174284	hydrocortisone acetate 10 MG/ML / pramoxine hydrochloride 10 MG/ML Topical Foam		Other - Specific to initial possibly-pregnant cohort (633K)				X			
42800419	ammonia 150 MG/ML Nasal Inhalant		Other - Specific to initial possibly-pregnant cohort (633K)				X			
1593918	30 ML epinephrine 0.005 MG/ML / lidocaine hydrochloride 15 MG/ML Injection		Other - Specific to initial possibly-pregnant cohort (633K)				X			
444094	Finding related to pregnancy		Other - Specific to initial possibly-pregnant cohort (633K)				X			
43526402	doxylamine succinate 10 MG / pyridoxine hydrochloride 10 MG Delayed Release Oral Tablet [Diclegis]		Other - Specific to initial possibly-pregnant cohort (633K)				X			
46271370	Assessment of fetal gestational age		Other - Specific to initial possibly-pregnant cohort (633K)				X			
3034112	Fetal cell screen [Interpretation] in Blood		Other - Specific to initial possibly-pregnant cohort (633K)				X			
19087317	lanolin		Other - Specific to initial possibly-pregnant cohort (633K)				X			
3022499	Rh immune globulin dosage.vials recommended [#]		Other - Specific to initial possibly-pregnant cohort (633K)				X			
1150926	misoprostol 0.2 MG Oral Tablet		Other - Specific to initial possibly-pregnant cohort (633K)				X			
44818159	docosaenoic acid 300 MG / eicosapentaenoic acid 0.75 MG Oral Capsule		Other - Specific to initial possibly-pregnant cohort (633K)				X			
40166455	penicillin G potassium 1000000 UNT/ML Injectable Solution		Other - Specific to initial possibly-pregnant cohort (633K)				X			
4016041	Diabetic on diet only		Other - Specific to initial possibly-pregnant cohort (633K)				X			
4307113	Genetics education, guidance, counseling		Other - Specific to initial possibly-pregnant cohort (633K)				X			
46275283	500 ML magnesium sulfate 40 MG/ML Injection		Other - Specific to initial possibly-pregnant cohort (633K)				X			
36249938	1 ML hydroxyprogesterone caproate (USP) 250 MG/ML Injection		Other - Specific to initial possibly-pregnant cohort (633K)				X			
1593915	10 ML epinephrine 0.005 MG/ML / lidocaine hydrochloride 15 MG/ML Injection [Xylocaine with Epinephrine]		Other - Specific to initial possibly-pregnant cohort (633K)				X			
43531008	Pre-existing type 1 diabetes mellitus in pregnancy		Other - Specific to initial possibly-pregnant cohort (633K)				X			
45757107	Malignant neoplastic disease in pregnancy		Other - Specific to initial possibly-pregnant cohort (633K)				X			
3050396	Erythrocytes.fetal/1000 erythrocytes [Ratio] in Blood by Kleihauer-Betke method		Other - Specific to initial possibly-pregnant cohort (633K)				X			
3032980	Neural tube defect risk [Likelihood] in Population		Other - Specific to initial possibly-pregnant cohort (633K)				X			
40213213	Rho(D) Immune globulin- IV or IM		Other - Specific to initial possibly-pregnant cohort (633K)				X			
964336	1.1 ML hydroxyprogesterone caproate (USP) 250 MG/ML Auto-Injector [Makena]		Other - Specific to initial possibly-pregnant cohort (633K)				X			
40178990	20 ML chloroprocaine hydrochloride 30 MG/ML Injection		Other - Specific to initial possibly-pregnant cohort (633K)				X			
1593598	nalbuphine Injection		Other - Specific to initial possibly-pregnant cohort (633K)				X			
1150871	misoprostol		Other - Specific to initial possibly-pregnant cohort (633K)				X			
40760179	Estimated date of delivery Narrative		Other - Specific to initial possibly-pregnant cohort (633K)				X			
4013995	RhD positive		Other - Specific to initial possibly-pregnant cohort (633K)				X			
3022202	Estriol (E3) [Multiple of the median] in Serum or Plasma		Other - Specific to initial possibly-pregnant cohort (633K)				X			
4188598	High risk pregnancy		Other - Specific to initial possibly-pregnant cohort (633K)				X			
43530950	Complication occurring during pregnancy		Other - Specific to initial possibly-pregnant cohort (633K)				X			
4016045	Diabetic - good control		Other - Specific to initial possibly-pregnant cohort (633K)				X			
1593776	terbutaline Injection		Other - Specific to initial possibly-pregnant cohort (633K)				X			
45765728	Supervision of high risk pregnancy		Other - Specific to initial possibly-pregnant cohort (633K)				X			
40479205	History of premature labor		Other - Specific to initial possibly-pregnant cohort (633K)				X			

42708649	witch hazel 500 MG/ML Medicated Pad [Tucks]		Other - Specific to initial possibly-pregnant cohort (633K)				X			
42534813	Maternal tobacco use in pregnancy		Other - Specific to initial possibly-pregnant cohort (633K)				X			
1391470	ferrous asparto glycinate		Other - Specific to initial possibly-pregnant cohort (633K)				X			
4228043	Pruritic urticarial papules and plaques of pregnancy		Other - Specific to initial possibly-pregnant cohort (633K)				X			
3003515	Hemoglobin F/Hemoglobin.total in Blood by Kleihauer-Betke method		Other - Specific to initial possibly-pregnant cohort (633K)				X			
535842	Rho(D) immune globulin, human 1500 UNT Prefilled Syringe [RhoGAM]		Other - Specific to initial possibly-pregnant cohort (633K)				X			
1728442	penicillin G 600000 UNT Injectable Suspension		Other - Specific to initial possibly-pregnant cohort (633K)				X			
46275941	betamethasone 6 MG/ML Injectable Suspension		Other - Specific to initial possibly-pregnant cohort (633K)				X			
43531010	Pre-existing type 2 diabetes mellitus in pregnancy		Other - Specific to initial possibly-pregnant cohort (633K)				X			
901660	benzocaine 200 MG/ML / menthol 5 MG/ML Topical Spray		Other - Specific to initial possibly-pregnant cohort (633K)				X			
438480	Abnormal glucose tolerance in mother complicating pregnancy, childbirth AND/OR puerperium		Other - Specific to initial possibly-pregnant cohort (633K)				X			
4013540	RhD negative		Other - Specific to initial possibly-pregnant cohort (633K)				X			
45757093	Alcohol dependence in pregnancy		Other - Specific to initial possibly-pregnant cohort (633K)				X			
40758860	Inject Rh immune globulin [Mass]		Other - Specific to initial possibly-pregnant cohort (633K)				X			
4118910	Maternal hypertension		Other - Specific to initial possibly-pregnant cohort (633K)				X			
19006855	kelp preparation		Other - Specific to initial possibly-pregnant cohort (633K)				X			
1329476	dinoprostone 10 MG Drug Implant		Other - Specific to initial possibly-pregnant cohort (633K)				X			
4025198	Hemorrhagic complication of pregnancy		Other - Specific to initial possibly-pregnant cohort (633K)				X			
43530695	Group B Streptococcus carrier		Other - Specific to initial possibly-pregnant cohort (633K)				X			
3029427	Karyotype [Identifier] in Blood or Tissue Narrative		Other - Specific to initial possibly-pregnant cohort (633K)				X			
3017809	Bicarbonate [Moles/volume] in Arterial cord blood		Other - Specific to initial possibly-pregnant cohort (633K)				X			
36713471	Routine postpartum follow-up		Other - Specific to initial possibly-pregnant cohort (633K)				X			
2787738	Introduction of Other Therapeutic Substance into Mouth and Pharynx, Via Natural or Artificial Opening		Other - Specific to initial possibly-pregnant cohort (633K)				X			
4193062	Early stage of pregnancy		Other - Specific to initial possibly-pregnant cohort (633K)				X			
19011034	citrate		Other - Specific to initial possibly-pregnant cohort (633K)				X			
40236088	hydroxyprogesterone caproate (USP) 250 MG/ML Injectable Solution		Other - Specific to initial possibly-pregnant cohort (633K)				X			
19035413	50 ML penicillin G potassium 20000 UNT/ML Injection		Other - Specific to initial possibly-pregnant cohort (633K)				X			
3003932	Carbon dioxide [Partial pressure] in Arterial cord blood		Other - Specific to initial possibly-pregnant cohort (633K)				X			
45757136	Physical abuse complicating pregnancy		Other - Specific to initial possibly-pregnant cohort (633K)				X			
4282746	Prenatal state of fetus		Other - Specific to initial possibly-pregnant cohort (633K)				X			
197344	Cesarean wound disruption		Other - Specific to initial possibly-pregnant cohort (633K)				X			
3032972	SMN1 gene+SMN2 gene targeted mutation analysis in Blood or Tissue by Molecular genetics method		Other - Specific to initial possibly-pregnant cohort (633K)				X			
4126571	Fetal problem		Other - Specific to initial possibly-pregnant cohort (633K)				X			
40222954	penicillin G potassium 1000000 UNT/ML Injectable Solution [Pfizerpen]		Other - Specific to initial possibly-pregnant cohort (633K)				X			
35604819	butorphanol Injection		Other - Specific to initial possibly-pregnant cohort (633K)				X			
19037446	calcium sulfate		Other - Specific to initial possibly-pregnant cohort (633K)				X			

4099889	Anemia of pregnancy		Other - Specific to initial possibly-pregnant cohort (633K)				X			
37017051	Supervision of high risk pregnancy with history of previous intrauterine death		Other - Specific to initial possibly-pregnant cohort (633K)				X			
3007090	Glucose tolerance [Interpretation] in Serum or Plasma		Other - Specific to initial possibly-pregnant cohort (633K)				X			
3006598	pH of Arterial cord blood		Other - Specific to initial possibly-pregnant cohort (633K)				X			
4230179	Reproductive technology management		Other - Specific to initial possibly-pregnant cohort (633K)				X			
37017058	Supervision of high risk pregnancy with poor reproductive history		Other - Specific to initial possibly-pregnant cohort (633K)				X			
36717636	Supervision of pregnancy with history of insufficient antenatal care		Other - Specific to initial possibly-pregnant cohort (633K)				X			
4253306	Suspected neurological disease		Other - Specific to initial possibly-pregnant cohort (633K)				X			
43531007	Pre-existing diabetes mellitus in pregnancy		Other - Specific to initial possibly-pregnant cohort (633K)				X			
1133732	butorphanol		Other - Specific to initial possibly-pregnant cohort (633K)				X			
4247552	Labor and delivery educational tour		Other - Specific to initial possibly-pregnant cohort (633K)				X			
2789516	Introduction of Other Therapeutic Substance into Female Reproductive, Via Natural or Artificial Opening		Other - Specific to initial possibly-pregnant cohort (633K)				X			
917389	benzocaine 0.2 MG/MG Topical Ointment		Other - Specific to initial possibly-pregnant cohort (633K)				X			
3018650	Bicarbonate [Moles/volume] in Venous cord blood		Other - Specific to initial possibly-pregnant cohort (633K)				X			
45757789	Postpartum gestational diabetes mellitus		Other - Specific to initial possibly-pregnant cohort (633K)				X			
4125611	Vaginal delivery		Other - Specific to initial possibly-pregnant cohort (633K)				X			
4041280	Postpartum finding		Other - Specific to initial possibly-pregnant cohort (633K)				X			
978053	citric acid 128 MG/ML / sodium citrate 98 MG/ML Oral Solution [Oracit]		Other - Specific to initial possibly-pregnant cohort (633K)				X			
46235338	Cell-free DNA.fetal/Cell-free DNA.total in Plasma cell-free DNA by Dosage of chromosome-specific cfDNA		Other - Specific to initial possibly-pregnant cohort (633K)				X			
43530951	Venous complication in the puerperium		Other - Specific to initial possibly-pregnant cohort (633K)				X			
19057128	oxytocin 10 UNT/ML Injectable Solution		Other - Specific to initial possibly-pregnant cohort (633K)				X			
46235716	Fetal Chromosome 21 trisomy [Presence] based on Plasma cell-free DNA by Dosage of chromosome-specific cfDNA		Other - Specific to initial possibly-pregnant cohort (633K)				X			
4326434	Gestational diabetes mellitus, class A>1<		Other - Specific to initial possibly-pregnant cohort (633K)				X			
3039437	Donated egg [Presence]		Other - Specific to initial possibly-pregnant cohort (633K)				X			
1508440	mifepristone 200 MG Oral Tablet [Mifeprex]		Other - Specific to initial possibly-pregnant cohort (633K)				X			
4200256	Insufficient weight gain of pregnancy		Other - Specific to initial possibly-pregnant cohort (633K)				X			
3017942	Specimen drawn [Date and time] of Serum or Plasma		Other - Specific to initial possibly-pregnant cohort (633K)				X			
42620786	Bupivacaine / Fentanyl Injectable Solution		Other - Specific to initial possibly-pregnant cohort (633K)				X			
19134227	witch hazel 500 MG/ML Medicated Pad		Other - Specific to initial possibly-pregnant cohort (633K)				X			
43533847	Fetal Trisomy 13 risk [Interpretation] based on Plasma cell-free DNA by Dosage of chromosome-specific cfDNA Qualitative		Other - Specific to initial possibly-pregnant cohort (633K)				X			
923840	dibucaine		Other - Specific to initial possibly-pregnant cohort (633K)				X			
1236744	terbutaline		Other - Specific to initial possibly-pregnant cohort (633K)				X			
36712695	Suspected fetal abnormality affecting management of mother		Other - Specific to initial possibly-pregnant cohort (633K)				X			
3046451	Rubella virus Ab [Presence] in Serum by Immunoassay		Other - Specific to initial possibly-pregnant cohort (633K)				X			
4031364	Healthcare supervision finding		Other - Specific to initial possibly-pregnant cohort (633K)				X			
3022007	Birth date		Other - Specific to initial possibly-pregnant cohort (633K)				X			

4176015	Hypothyroidism in pregnancy		Other - Specific to initial possibly-pregnant cohort (633K)				X			
40661402	Medical home program, comprehensive care coordination and planning, maintenance of plan		Other - Specific to initial possibly-pregnant cohort (633K)				X			
40213212	Rho(D) Immune globulin - IM		Other - Specific to initial possibly-pregnant cohort (633K)				X			
19040768	misoprostol 0.1 MG Oral Tablet [Cytotec]		Other - Specific to initial possibly-pregnant cohort (633K)				X			
4081720	Placenta previa partialis		Other - Specific to initial possibly-pregnant cohort (633K)				X			
3019199	Base deficit in Capillary blood		Other - Specific to initial possibly-pregnant cohort (633K)				X			
43530881	Suspected fetal disorder		Other - Specific to initial possibly-pregnant cohort (633K)				X			
3030608	Diabetes status [Identifier]		Other - Specific to initial possibly-pregnant cohort (633K)				X			
4058565	H/O: cesarean section		Other - Specific to initial possibly-pregnant cohort (633K)				X			
19077143	17-alpha-hydroxyprogesterone		Other - Specific to initial possibly-pregnant cohort (633K)				X			
45763657	Suspected hereditary disease		Other - Specific to initial possibly-pregnant cohort (633K)				X			
40482406	Low lying placenta		Other - Specific to initial possibly-pregnant cohort (633K)				X			
4129027	Viable fetus in abdominal pregnancy		Other - Specific to initial possibly-pregnant cohort (633K)				X			
3021706	Oxygen [Partial pressure] in Arterial cord blood		Other - Specific to initial possibly-pregnant cohort (633K)				X			
3004341	History of Hereditary disorders		Other - Specific to initial possibly-pregnant cohort (633K)				X			
46235498	Fetal Chromosome 13+18+21+X+Y aneuploidy [Presence] based on Plasma cell-free DNA by Dosage of chromosome-specific cfDNA		Other - Specific to initial possibly-pregnant cohort (633K)				X			
2787754	Introduction of Other Therapeutic Substance into Mouth and Pharynx, External Approach		Other - Specific to initial possibly-pregnant cohort (633K)				X			
535811	Rho(D) immune globulin, human 1500 UNT Prefilled Syringe		Other - Specific to initial possibly-pregnant cohort (633K)				X			
2779060	Control Bleeding in Genitourinary Tract, Via Natural or Artificial Opening		Other - Specific to initial possibly-pregnant cohort (633K)				X			
40229419	benzocaine 200 MG/ML / menthol 5 MG/ML Topical Spray [Little Ouchies]		Other - Specific to initial possibly-pregnant cohort (633K)				X			
3046418	Insulin dependent diabetes mellitus [Presence]		Other - Specific to initial possibly-pregnant cohort (633K)				X			
45765732	Supervision of high risk pregnancy for social problem		Other - Specific to initial possibly-pregnant cohort (633K)				X			
4060188	H/O: miscarriage		Other - Specific to initial possibly-pregnant cohort (633K)				X			
19003290	ranitidine 25 MG/ML Injectable Solution [Zantac]		Other - Specific to initial possibly-pregnant cohort (633K)				X			
35604820	1 ML butorphanol tartrate 2 MG/ML Injection		Other - Specific to initial possibly-pregnant cohort (633K)				X			
43531419	Short cervical length in pregnancy		Other - Specific to initial possibly-pregnant cohort (633K)				X			
42628981	1 ML oxytocin 10 UNT/ML Injection [Pitocin]		Other - Specific to initial possibly-pregnant cohort (633K)				X			
1150924	misoprostol 0.1 MG Oral Tablet		Other - Specific to initial possibly-pregnant cohort (633K)				X			
4080397	Blood group A Rh(D) negative		Other - Specific to initial possibly-pregnant cohort (633K)				X			
37208366	Breast feeding		Other - Specific to initial possibly-pregnant cohort (633K)				X			
3014737	Glucose [Mass/volume] in Serum or Plasma --3 hours post XXX challenge		Other - Specific to initial possibly-pregnant cohort (633K)				X			
927146	SMN1 (survival of motor neuron 1, telomeric) (eg, spinal muscular atrophy) gene analysis; dosage/deletion analysis (eg, carrier testing), includes SMN2 (survival of motor neuron 2, centromeric) analysis, if performed		Other - Specific to initial possibly-pregnant cohort (633K)				X			
36713460	Supervision of pregnancy with history of infertility		Other - Specific to initial possibly-pregnant cohort (633K)				X			
4082947	Blood group O Rh(D) negative		Other - Specific to initial possibly-pregnant cohort (633K)				X			
40222882	methylergonovine maleate 0.2 MG Oral Tablet [Methergine]		Other - Specific to initial possibly-pregnant cohort (633K)				X			

40171399	Rho(D) immune globulin, human 1500 UNT Prefilled Syringe [HyperRHO]		Other - Specific to initial possibly-pregnant cohort (633K)				X			
1326115	oxytocin		Other - Specific to initial possibly-pregnant cohort (633K)				X			
37018878	High risk pregnancy due to recurrent pregnancy loss		Other - Specific to initial possibly-pregnant cohort (633K)				X			
4246974	Suspected chromosome abnormality		Other - Specific to initial possibly-pregnant cohort (633K)				X			
3022828	Mother's race		Other - Specific to initial possibly-pregnant cohort (633K)				X			
3022670	pH of Venous cord blood		Other - Specific to initial possibly-pregnant cohort (633K)				X			
3026677	Oxygen [Partial pressure] in Venous cord blood		Other - Specific to initial possibly-pregnant cohort (633K)				X			
40659816	Medical home program, comprehensive care coordination and planning, initial plan		Other - Specific to initial possibly-pregnant cohort (633K)				X			
4091642	Fetal heart finding		Other - Specific to initial possibly-pregnant cohort (633K)				X			
3007696	Carbon dioxide [Partial pressure] in Venous cord blood		Other - Specific to initial possibly-pregnant cohort (633K)				X			
3020058	Glucose [Mass/volume] in Serum or Plasma --pre 100 g glucose PO		Other - Specific to initial possibly-pregnant cohort (633K)				X			
3002638	Streptococcus agalactiae [Presence] in Genital specimen by Organism specific culture		Other - Specific to initial possibly-pregnant cohort (633K)				X			
4060559	Uterine scar from previous surgery in pregnancy, childbirth and the puerperium with antenatal problem		Other - Specific to initial possibly-pregnant cohort (633K)				X			
36712703	Preterm labor without delivery		Other - Specific to initial possibly-pregnant cohort (633K)				X			
4059484	H/O: ectopic pregnancy		Other - Specific to initial possibly-pregnant cohort (633K)				X			
40480278	High risk pregnancy due to history of preterm labor		Other - Specific to initial possibly-pregnant cohort (633K)				X			
44818288	citric acid 1000 MG / potassium bicarbonate 344 MG / sodium bicarbonate 1050 MG Effervescent Oral Tablet		Other - Specific to initial possibly-pregnant cohort (633K)				X			
37110290	Disease of circulatory system complicating pregnancy childbirth and puerperium		Other - Specific to initial possibly-pregnant cohort (633K)				X			
3036848	Fibronectin.fetal [Presence] in Vaginal fluid		Other - Specific to initial possibly-pregnant cohort (633K)				X			
3048599	History of Neural tube defect Narrative		Other - Specific to initial possibly-pregnant cohort (633K)				X			
19071941	M-M-R II		Other - Specific to initial possibly-pregnant cohort (633K)				X			
40222881	methylergonovine maleate 0.2 MG Oral Tablet		Other - Specific to initial possibly-pregnant cohort (633K)				X			
42627901	Magnetic resonance (eg, proton) imaging, fetal, including placental and maternal pelvic imaging when performed; single or first gestation		Other - Specific to initial possibly-pregnant cohort (633K)				X			
21493324	Genomic structural variant copy number		Other - Specific to initial possibly-pregnant cohort (633K)				X			
4080398	Blood group B Rh(D) negative		Other - Specific to initial possibly-pregnant cohort (633K)				X			
37016105	History of pre-eclampsia		Other - Specific to initial possibly-pregnant cohort (633K)				X			
4024560	Hypertension AND/OR vomiting complicating pregnancy childbirth AND/OR puerperium		Other - Specific to initial possibly-pregnant cohort (633K)				X			
4143187	Anomaly of placenta		Other - Specific to initial possibly-pregnant cohort (633K)				X			
40241529	2 ML Rho(D) immune globulin, human 750 UNT/ML Prefilled Syringe		Other - Specific to initial possibly-pregnant cohort (633K)				X			
4060195	H/O: premature delivery		Other - Specific to initial possibly-pregnant cohort (633K)				X			
1329415	dinoprostone		Other - Specific to initial possibly-pregnant cohort (633K)				X			
42708650	witch hazel 500 MG/ML Medicated Pad [A.E.R. Witch Hazel]		Other - Specific to initial possibly-pregnant cohort (633K)				X			
4291933	Chronic hypertension complicating AND/OR reason for care during pregnancy		Other - Specific to initial possibly-pregnant cohort (633K)				X			
3029887	History of Trisomy 21 Narrative		Other - Specific to initial possibly-pregnant cohort (633K)				X			
1508439	mifepristone		Other - Specific to initial possibly-pregnant cohort (633K)				X			
19078927	mifepristone 200 MG Oral Tablet		Other - Specific to initial possibly-pregnant cohort (633K)				X			

2721177	Performance measurement, evaluation of patient self assessment, depression		Other - Specific to initial possibly-pregnant cohort (633K)				X			
3050414	Sonographer name		Other - Specific to initial possibly-pregnant cohort (633K)				X			
40239099	docosahexaenoic acid 250 MG Oral Capsule		Other - Specific to initial possibly-pregnant cohort (633K)				X			
37017056	Supervision of high risk pregnancy with poor obstetric history		Other - Specific to initial possibly-pregnant cohort (633K)				X			
2780119	Excision of Bilateral Fallopian Tubes, Open Approach		Other - Specific to initial possibly-pregnant cohort (633K)				X			
45757113	False labor before 37 completed weeks of gestation		Other - Specific to initial possibly-pregnant cohort (633K)				X			
3038313	History of Trisomy 21 Qualitative		Other - Specific to initial possibly-pregnant cohort (633K)				X			
1329477	dinoprostone 10 MG Drug Implant [Cervidil]		Other - Specific to initial possibly-pregnant cohort (633K)				X			
37020820	Fetal Chorion [Type]		Other - Specific to initial possibly-pregnant cohort (633K)				X			
45757788	Postpartum pregnancy-induced hypertension		Other - Specific to initial possibly-pregnant cohort (633K)				X			
4157351	Finding of lactation		Other - Specific to initial possibly-pregnant cohort (633K)				X			
45757149	Preterm premature rupture of membranes with onset of labor unknown		Other - Specific to initial possibly-pregnant cohort (633K)				X			
46234957	Fetal Chromosome X and Y aneuploidy [interpretation] based on dosage of chromosome-specific cell-free DNA in Maternal plasma		Other - Specific to initial possibly-pregnant cohort (633K)				X			
2779072	Division of Female Perineum, External Approach		Other - Specific to initial possibly-pregnant cohort (633K)				X			
4154711	Varicella non-immune		Other - Specific to initial possibly-pregnant cohort (633K)				X			
2213179	Infectious agent antigen detection by immunoassay with direct optical observation; Streptococcus, group B		Other - Specific to initial possibly-pregnant cohort (633K)				X			
46235714	Fetal Chromosome X and Y aneuploidy [Presence] based on Plasma cell-free DNA by Dosage of chromosome-specific cfDNA		Other - Specific to initial possibly-pregnant cohort (633K)				X			
40241530	2 ML Rho(D) immune globulin, human 750 UNT/ML Prefilled Syringe [Rhophylac]		Other - Specific to initial possibly-pregnant cohort (633K)				X			
40222885	1 ML methylergonovine maleate 0.2 MG/ML Injection		Other - Specific to initial possibly-pregnant cohort (633K)				X			
2778638	Repair Vagina, External Approach		Other - Specific to initial possibly-pregnant cohort (633K)				X			
46235339	Cell-free DNA.fetal/Cell-free DNA.total in Plasma cell-free DNA by Dosage of chromosome-specific cfDNA Narrative		Other - Specific to initial possibly-pregnant cohort (633K)				X			
46235499	Fetal Chromosome 13 trisomy [Presence] based on Plasma cell-free DNA by Dosage of chromosome-specific cfDNA		Other - Specific to initial possibly-pregnant cohort (633K)				X			
3048559	Fetal Nuchal fold [Multiple of the median] Thickness US		Other - Specific to initial possibly-pregnant cohort (633K)				X			
46235500	Fetal Chromosome 18 trisomy [Presence] based on Plasma cell-free DNA by Dosage of chromosome-specific cfDNA		Other - Specific to initial possibly-pregnant cohort (633K)				X			
35625971	Vomiting during third trimester of pregnancy		Other - Specific to initial possibly-pregnant cohort (633K)				X			
45757079	Pre-existing diabetes mellitus in mother complicating childbirth		Other - Specific to initial possibly-pregnant cohort (633K)				X			
4232026	Ultrasound study follow-up		Other - Specific to initial possibly-pregnant cohort (633K)				X			
4324607	Transvaginal obstetric ultrasonography		Other - Specific to initial possibly-pregnant cohort (633K)				X			
41107651	Oxytocin 10 UNT		Other - Specific to initial possibly-pregnant cohort (633K)				X			
4143214	Maternal pyrexia in labor		Other - Specific to initial possibly-pregnant cohort (633K)				X			
40539858	Pregnancy with abortive outcome		Other - Specific to initial possibly-pregnant cohort (633K)				X			
4172863	Fetal dysrhythmia		Other - Specific to initial possibly-pregnant cohort (633K)				X			
40067433	oxytocin Injectable Solution		Other - Specific to initial possibly-pregnant cohort (633K)				X			
3020320	Base excess in Cord blood by calculation		Other - Specific to initial possibly-pregnant cohort (633K)				X			
45757112	Obesity in mother complicating childbirth		Other - Specific to initial possibly-pregnant cohort (633K)				X			
40065804	misoprostol Oral Tablet		Other - Specific to initial possibly-pregnant cohort (633K)				X			

42629024	oxytocin 20 UNT/ML Injectable Solution [Oxoject]		Other - Specific to initial possibly-pregnant cohort (633K)				X			
3029645	Glucose screen gestational panel - Urine and Serum or Plasma		Other - Specific to initial possibly-pregnant cohort (633K)				X			
45757092	Anemia in mother complicating childbirth		Other - Specific to initial possibly-pregnant cohort (633K)				X			
21030769	Oxytocin 0.06 UNT/ML Injectable Solution		Other - Specific to initial possibly-pregnant cohort (633K)				X			
4115963	Injury of female perineum		Other - Specific to initial possibly-pregnant cohort (633K)				X			
4090719	Baby overdue		Other - Specific to initial possibly-pregnant cohort (633K)				X			
1154222	fentanyl 0.002 MG/ML		Other - Specific to initial possibly-pregnant cohort (633K)				X			
45757177	Tobacco use in mother complicating childbirth		Other - Specific to initial possibly-pregnant cohort (633K)				X			
2110237	Ligation or transection of fallopian tube(s) when done at the time of cesarean delivery or intra-abdominal surgery (not a separate procedure) (List separately in addition to code for primary procedure)		Other - Specific to initial possibly-pregnant cohort (633K)				X			
45757134	Group B streptococcus infection in mother complicating childbirth		Other - Specific to initial possibly-pregnant cohort (633K)				X			
21149534	Bupivacaine 0.625 MG/ML / Fentanyl 0.002 MG/ML Injection		Other - Specific to initial possibly-pregnant cohort (633K)				X			
45757775	Failed attempted vaginal birth after previous cesarean section		Other - Specific to initial possibly-pregnant cohort (633K)				X			
3043569	Date Rh immune globulin given		Other - Specific to initial possibly-pregnant cohort (633K)				X			
4091643	Fetal heart rate		Other - Specific to initial possibly-pregnant cohort (633K)				X			
35605516	methylergonovine maleate 0.2 MG/ML Injection		Other - Specific to initial possibly-pregnant cohort (633K)				X			
3030109	pH of Arterial cord blood adjusted to patient's actual temperature		Other - Specific to initial possibly-pregnant cohort (633K)				X			
3032186	pH of Venous cord blood adjusted to patient's actual temperature		Other - Specific to initial possibly-pregnant cohort (633K)				X			
3029246	Carbon dioxide [Partial pressure] adjusted to patient's actual temperature in Venous cord blood		Other - Specific to initial possibly-pregnant cohort (633K)				X			
3043950	Carbon dioxide, total [Moles/volume] in Arterial cord blood		Other - Specific to initial possibly-pregnant cohort (633K)				X			
3011972	Carbon dioxide [Partial pressure] in Cord blood		Other - Specific to initial possibly-pregnant cohort (633K)				X			
3025864	Chromosome 21 trisomy [Presence] in Blood or Tissue by Cytogenetics		Other - Specific to initial possibly-pregnant cohort (633K)				X			
4127696	Finding of uterine contractions		Other - Specific to initial possibly-pregnant cohort (633K)				X			
3028646	Oxygen [Partial pressure] in Cord blood		Other - Specific to initial possibly-pregnant cohort (633K)				X			
40129954	lanolin Topical Ointment		Other - Specific to initial possibly-pregnant cohort (633K)				X			
3031279	Fetal Narrative [Interpretation] Study observation general, multiple fetuses US		Other - Specific to initial possibly-pregnant cohort (633K)				X			
42709935	Type 3a third degree laceration of perineum		Other - Specific to initial possibly-pregnant cohort (633K)				X			
46235831	Pregnancy associated plasma protein A [Multiple of the median] adjusted in Serum or Plasma		Other - Specific to initial possibly-pregnant cohort (633K)				X			
44784460	Dietary education for impaired glucose tolerance		Other - Specific to initial possibly-pregnant cohort (633K)				X			
4313474	Prenatal visit		Other - Specific to initial possibly-pregnant cohort (633K)				X			
3032759	Carbon dioxide [Partial pressure] adjusted to patient's actual temperature in Arterial cord blood		Other - Specific to initial possibly-pregnant cohort (633K)				X			
3040332	Fetal Nasal bone diaphysis [Length] US		Other - Specific to initial possibly-pregnant cohort (633K)				X			
3004395	History of family member diseases		Other - Specific to initial possibly-pregnant cohort (633K)				X			
46274509	anhydrous lanolin		Other - Specific to initial possibly-pregnant cohort (633K)				X			
4301257	Contraction		Other - Specific to initial possibly-pregnant cohort (633K)				X			
37498112	lanolin 1000 MG/ML Topical Cream		Other - Specific to initial possibly-pregnant cohort (633K)				X			

45757141	Viral hepatitis in mother complicating childbirth		Other - Specific to initial possibly-pregnant cohort (633K)				X			
42708422	benzocaine 200 MG/ML / menthol 5 MG/ML Topical Spray [Clinistat]		Other - Specific to initial possibly-pregnant cohort (633K)				X			
45772074	Obstructed labor due to incomplete rotation of fetal head		Other - Specific to initial possibly-pregnant cohort (633K)				X			
40222880	methylergonovine maleate 0.2 MG		Other - Specific to initial possibly-pregnant cohort (633K)				X			
4164838	Edinburgh postnatal depression scale		Other - Specific to initial possibly-pregnant cohort (633K)				X			
4180145	Disorders of fetal movement		Other - Specific to initial possibly-pregnant cohort (633K)				X			
40760207	Fetal Movement - Reported		Other - Specific to initial possibly-pregnant cohort (633K)				X			
42709936	Type 3b third degree laceration of perineum		Other - Specific to initial possibly-pregnant cohort (633K)				X			
2775777	Dilation of Cervix, Via Natural or Artificial Opening		Other - Specific to initial possibly-pregnant cohort (633K)				X			
3037749	Bicarbonate [Moles/volume] in Cord blood		Other - Specific to initial possibly-pregnant cohort (633K)				X			
19035204	oxytocin 10 UNT/ML Injectable Solution [Pitocin]		Other - Specific to initial possibly-pregnant cohort (633K)				X			
2618151	Prenatal care, at-risk enhanced service; antepartum management		Other - Specific to initial possibly-pregnant cohort (633K)				X			
45757688	Infectious disease in mother complicating childbirth		Other - Specific to initial possibly-pregnant cohort (633K)				X			
2784582	Insertion of Monitoring Electrode into Products of Conception, Via Natural or Artificial Opening		Other - Specific to initial possibly-pregnant cohort (633K)				X			
19123306	lanolin 1 MG/MG Topical Ointment		Other - Specific to initial possibly-pregnant cohort (633K)				X			
43527959	Fetal congenital abnormalities, biochemical assays of two proteins (PAPP-A, hCG [any form]), utilizing maternal serum, algorithm reported as a risk score		Other - Specific to initial possibly-pregnant cohort (633K)				X			
40770920	Previous fetus defect		Other - Specific to initial possibly-pregnant cohort (633K)				X			
40770418	Trisomy 18 + Trisomy 13 risk [Likelihood] in Fetus		Other - Specific to initial possibly-pregnant cohort (633K)				X			
3000394	Fetal presentation palpation		Other - Specific to initial possibly-pregnant cohort (633K)				X			
3030441	Oxygen [Partial pressure] adjusted to patient's actual temperature in Venous cord blood		Other - Specific to initial possibly-pregnant cohort (633K)				X			
4266767	Positions of breech presentation - finding		Other - Specific to initial possibly-pregnant cohort (633K)				X			
3025833	Maternal risk factors		Other - Specific to initial possibly-pregnant cohort (633K)				X			
2787780	Monitoring of Products of Conception, Cardiac Rate, Via Natural or Artificial Opening		Other - Specific to initial possibly-pregnant cohort (633K)				X			
40143309	lanolin Topical Cream		Other - Specific to initial possibly-pregnant cohort (633K)				X			
4311447	Prenatal initial visit		Other - Specific to initial possibly-pregnant cohort (633K)				X			
45757124	Gestational diabetes mellitus in childbirth		Other - Specific to initial possibly-pregnant cohort (633K)				X			
2787789	Monitoring of Products of Conception, Cardiac Rhythm, External Approach		Other - Specific to initial possibly-pregnant cohort (633K)				X			
40222605	folic acid / iron carbonyl Oral Tablet		Other - Specific to initial possibly-pregnant cohort (633K)				X			
46235712	Fetal Chromosome 13+18+21 trisomy [Presence] based on Plasma cell-free DNA by Dosage of chromosome-specific cfDNA		Other - Specific to initial possibly-pregnant cohort (633K)				X			
4208215	Preterm premature rupture of membranes		Other - Specific to initial possibly-pregnant cohort (633K)				X			
40761030	Carbon dioxide, total [Moles/volume] in Arterial cord blood by calculation		Other - Specific to initial possibly-pregnant cohort (633K)				X			
4060036	Obstructed labor due to breech presentation		Other - Specific to initial possibly-pregnant cohort (633K)				X			
45757094	Previous bariatric surgery in mother complicating childbirth		Other - Specific to initial possibly-pregnant cohort (633K)				X			
4242238	Intrapartum hemorrhage		Other - Specific to initial possibly-pregnant cohort (633K)				X			
2785338	Reposition Products of Conception, External Approach		Other - Specific to initial possibly-pregnant cohort (633K)				X			
4061852	Obstructed labor due to abnormality of maternal pelvic organs		Other - Specific to initial possibly-pregnant cohort (633K)				X			

42628979	1 ML oxytocin 10 UNT/ML Injection		Other - Specific to initial possibly-pregnant cohort (633K)				X			
4064296	Prem rupture of membranes onset of labor within 24 hours		Other - Specific to initial possibly-pregnant cohort (633K)				X			
2101594	Anesthesia for vaginal procedures (including biopsy of labia, vagina, cervix or endometrium); cervical cerclage		Other - Specific to initial possibly-pregnant cohort (633K)				X			
45757153	Viral disease in mother complicating childbirth		Other - Specific to initial possibly-pregnant cohort (633K)				X			
2110302	Insertion of cervical dilator (eg, laminaria, prostaglandin) (separate procedure)		Other - Specific to initial possibly-pregnant cohort (633K)				X			
40758412	Fetal Heart rate		Other - Specific to initial possibly-pregnant cohort (633K)				X			
3033254	Oxygen [Partial pressure] adjusted to patient's actual temperature in Arterial cord blood		Other - Specific to initial possibly-pregnant cohort (633K)				X			
3035080	pH of Amniotic fluid		Other - Specific to initial possibly-pregnant cohort (633K)				X			
4334638	Fetal heart disorder		Other - Specific to initial possibly-pregnant cohort (633K)				X			
2780619	Extirpation of Matter from Cervix, Via Natural or Artificial Opening		Other - Specific to initial possibly-pregnant cohort (633K)				X			
44790279	Obstetric ultrasound monitoring		Other - Specific to initial possibly-pregnant cohort (633K)				X			
1728548	penicillin G 100000 UNT/ML Injectable Solution		Other - Specific to initial possibly-pregnant cohort (633K)				X			
4030067	Impaired glucose tolerance in pregnancy		Other - Specific to initial possibly-pregnant cohort (633K)				X			
2778650	Repair Vulva, External Approach		Other - Specific to initial possibly-pregnant cohort (633K)				X			
3011988	pH of Cord blood		Other - Specific to initial possibly-pregnant cohort (633K)				X			
35774715	Rho(D) Immune Globulin Injectable Solution [Rhophylac]		Other - Specific to initial possibly-pregnant cohort (633K)				X			
3031159	Ultrasound date		Other - Specific to initial possibly-pregnant cohort (633K)				X			
2780609	Extirpation of Matter from Uterus, Via Natural or Artificial Opening		Other - Specific to initial possibly-pregnant cohort (633K)				X			
42479054	Bupivacaine 1.2 MG/ML / Fentanyl 0.002 MG/ML Injection		Other - Specific to initial possibly-pregnant cohort (633K)				X			
40486958	Insertion of catheter into lumbar epidural space		Other - Specific to initial possibly-pregnant cohort (633K)				X			
2787779	Monitoring of Products of Conception, Cardiac Electrical Activity, Via Natural or Artificial Opening		Other - Specific to initial possibly-pregnant cohort (633K)				X			
43527962	Fetal congenital abnormalities, biochemical assays of four analytes (AFP, uE3, hCG [any form], DIA) utilizing maternal serum, algorithm reported as a risk score (may include additional results from previous biochemical testing)		Other - Specific to initial possibly-pregnant cohort (633K)				X			
4088026	Postnatal care status		Other - Specific to initial possibly-pregnant cohort (633K)				X			
4127201	Hypertonic contractions		Other - Specific to initial possibly-pregnant cohort (633K)				X			
2787975	Introduction of Electrolytic and Water Balance Substance into Products of Conception, Via Natural or Artificial Opening		Other - Specific to initial possibly-pregnant cohort (633K)				X			
3020321	Streptococcus agalactiae [Presence] in Vaginal fluid by Organism specific culture		Other - Specific to initial possibly-pregnant cohort (633K)				X			
2784545	Drainage of Amniotic Fluid, Therapeutic from Products of Conception, Via Natural or Artificial Opening		Other - Specific to initial possibly-pregnant cohort (633K)				X			
19007678	Varivax		Other - Specific to initial possibly-pregnant cohort (633K)				X			
442333	Failed induction of labor		Other - Specific to initial possibly-pregnant cohort (633K)				X			
2787977	Introduction of Other Therapeutic Substance into Products of Conception, Via Natural or Artificial Opening		Other - Specific to initial possibly-pregnant cohort (633K)				X			
4058562	H/O: infertility - female		Other - Specific to initial possibly-pregnant cohort (633K)				X			
21493402	Recommended follow-up [Identifier] Narrative		Other - Specific to initial possibly-pregnant cohort (633K)				X			
19127608	penicillin G potassium 100000 UNT/ML Injectable Solution		Other - Specific to initial possibly-pregnant cohort (633K)				X			
19057129	1 ML carboprost 0.25 MG/ML Injection		Other - Specific to initial possibly-pregnant cohort (633K)				X			
45757118	Spontaneous onset of labor between 37 and 39 weeks gestation with planned cesarean section		Other - Specific to initial possibly-pregnant cohort (633K)				X			

4126257	Finding of measures of pregnancy		Other - Specific to initial possibly-pregnant cohort (633K)				X			
3007435	Base excess in Venous cord blood by calculation		Other - Specific to initial possibly-pregnant cohort (633K)				X			
19122065	oxytocin 20 UNT/ML Injectable Solution		Other - Specific to initial possibly-pregnant cohort (633K)				X			
40079076	Rho(D) immune globulin Injectable Solution		Other - Specific to initial possibly-pregnant cohort (633K)				X			
2787054	Measurement of Products of Conception, Cardiac Rate, External Approach		Other - Specific to initial possibly-pregnant cohort (633K)				X			
43018343	Introduction of Hormone into Female Reproductive, Via Natural or Artificial Opening		Other - Specific to initial possibly-pregnant cohort (633K)				X			
4302765	Endometritis		Other - Specific to initial possibly-pregnant cohort (633K)				X			
43011798	hydrocortisone acetate 10 MG/ML / pramoxine hydrochloride 10 MG/ML Rectal Cream [Analpram HC]		Other - Specific to initial possibly-pregnant cohort (633K)				X			
2784583	Insertion of Other Device into Products of Conception, Via Natural or Artificial Opening		Other - Specific to initial possibly-pregnant cohort (633K)				X			
2212482	Molecular diagnostics; amplification, target, multiplex, first 2 nucleic acid sequences		Other - Specific to initial possibly-pregnant cohort (633K)				X			
2212491	Molecular diagnostics; separation and identification by high resolution technique (eg, capillary electrophoresis), each nucleic acid preparation		Other - Specific to initial possibly-pregnant cohort (633K)				X			
3016214	Estriol (E3).unconjugated [Presence] in Serum or Plasma		Other - Specific to initial possibly-pregnant cohort (633K)				X			
917176	benzocaine 200 MG/ML Topical Spray [Americaine]		Other - Specific to initial possibly-pregnant cohort (633K)				X			
3032496	First and Second trimester integrated maternal screen [Interpretation]		Other - Specific to initial possibly-pregnant cohort (633K)				X			
2786438	Introduction of Other Hormone into Peripheral Vein, Percutaneous Approach		Other - Specific to initial possibly-pregnant cohort (633K)				X			
2212494	Mutation identification by enzymatic ligation or primer extension, single segment, each segment (eg, oligonucleotide ligation assay [OLA], single base chain extension [SBCE], or allele-specific primer extension [ASPE])		Other - Specific to initial possibly-pregnant cohort (633K)				X			
2212483	Molecular diagnostics; amplification, target, multiplex, each additional nucleic acid sequence beyond 2 (List separately in addition to code for primary procedure)		Other - Specific to initial possibly-pregnant cohort (633K)				X			
3007259	Gestational age method		Other - Specific to initial possibly-pregnant cohort (633K)				X			
4147345	Pregnancy with mental disorders		Other - Specific to initial possibly-pregnant cohort (633K)				X			
37498115	lanolin 1000 MG/ML Topical Cream [Lansinoh]		Other - Specific to initial possibly-pregnant cohort (633K)				X			
4170304	Ferning - cervical test		Other - Specific to initial possibly-pregnant cohort (633K)				X			
46234980	Fetal Y chromosome [Interpretation] based on Plasma cell-free DNA by Sequencing		Other - Specific to initial possibly-pregnant cohort (633K)				X			
3657563	First trimester bleeding		Other - Specific to initial possibly-pregnant cohort (633K)				X			
43018095	Insertion of Contraceptive Device into Uterus, Open Approach		Other - Specific to initial possibly-pregnant cohort (633K)				X			
4146627	Non-proteinuric hypertension of pregnancy		Other - Specific to initial possibly-pregnant cohort (633K)				X			
36713030	Bleeding from female genital tract during pregnancy		Other - Specific to initial possibly-pregnant cohort (633K)				X			
4209094	Anemia during the puerperium		Other - Specific to initial possibly-pregnant cohort (633K)				X			
3001951	Fetal Crown Rump length US		Other - Specific to initial possibly-pregnant cohort (633K)				X			
40766945	Current smoker		Other - Specific to initial possibly-pregnant cohort (633K)				X			
2787787	Monitoring of Products of Conception, Cardiac Electrical Activity, External Approach		Other - Specific to initial possibly-pregnant cohort (633K)				X			
2756126	Repair Perineum Muscle, Open Approach		Other - Specific to initial possibly-pregnant cohort (633K)				X			
36249939	17-alpha-hydroxyprogesterone Injection [Makena]		Other - Specific to initial possibly-pregnant cohort (633K)				X			
2756499	Repair Perineum Skin, External Approach		Other - Specific to initial possibly-pregnant cohort (633K)				X			
44783943	Anti-D isoimmunization affecting pregnancy		Other - Specific to initial possibly-pregnant cohort (633K)				X			
2787788	Monitoring of Products of Conception, Cardiac Rate, External Approach		Other - Specific to initial possibly-pregnant cohort (633K)				X			

46234981	Fetal Y chromosome [Presence] based on Plasma cell-free DNA by Sequencing		Other - Specific to initial possibly-pregnant cohort (633K)				X			
443445	Outcome of delivery - finding		Other - Specific to initial possibly-pregnant cohort (633K)				X			
19035414	50 ML penicillin G potassium 60000 UNT/ML Injection		Other - Specific to initial possibly-pregnant cohort (633K)				X			
4088025	Antenatal care status		Other - Specific to initial possibly-pregnant cohort (633K)				X			
46275855	magnesium sulfate Injection		Other - Specific to initial possibly-pregnant cohort (633K)				X			
4111608	Normal fetal growth		Other - Specific to initial possibly-pregnant cohort (633K)				X			
3049700	In vitro fertilization pregnancy		Other - Specific to initial possibly-pregnant cohort (633K)				X			
4088927	Pregnancy, childbirth and puerperium finding		Other - Specific to initial possibly-pregnant cohort (633K)				X			
44507597	lidocaine 5 MG/ML Topical Spray		Other - Specific to initial possibly-pregnant cohort (633K)				X			
1305637	methylergonovine		Other - Specific to initial possibly-pregnant cohort (633K)				X			
3025053	Alpha-1-Fetoprotein [Presence] in Serum or Plasma		Other - Specific to initial possibly-pregnant cohort (633K)				X			
4054949	Breastfeeding education		Other - Specific to initial possibly-pregnant cohort (633K)				X			
4034966	Hyperglycemic disorder in pregnancy		Other - Specific to initial possibly-pregnant cohort (633K)				X			
3019671	Effacement Cervix		Other - Specific to initial possibly-pregnant cohort (633K)				X			
19049150	carboprost		Other - Specific to initial possibly-pregnant cohort (633K)				X			
42536563	Obstructed labor due to shoulder dystocia		Other - Specific to initial possibly-pregnant cohort (633K)				X			
46257465	Fetal chromosomal aneuploidy (eg, trisomy 21, monosomy X) genomic sequence analysis panel, circulating cell-free fetal DNA in maternal blood, must include analysis of chromosomes 13, 18, and 21		Other - Specific to initial possibly-pregnant cohort (633K)				X			
19066475	1 ML carboprost 0.25 MG/ML Injection [Hemabate]		Other - Specific to initial possibly-pregnant cohort (633K)				X			
40163433	1 ML terbutaline sulfate 1 MG/ML Injection		Other - Specific to initial possibly-pregnant cohort (633K)				X			
3039433	History of Neural tube defect Qualitative		Other - Specific to initial possibly-pregnant cohort (633K)				X			
46235057	Radiology Note		Other - Specific to initial possibly-pregnant cohort (633K)				X			
40072606	penicillin G Injectable Solution		Other - Specific to initial possibly-pregnant cohort (633K)				X			
19129581	benzocaine 200 MG/ML / menthol 5 MG/ML Topical Spray [Dermoplast Pain]		Other - Specific to initial possibly-pregnant cohort (633K)				X			
3012213	Cervical canal external os Diameter palpation		Other - Specific to initial possibly-pregnant cohort (633K)				X			
44793477	Antenatal screening status		Other - Specific to initial possibly-pregnant cohort (633K)				X			
3005033	Fetal Nuchal fold Thickness US		Other - Specific to initial possibly-pregnant cohort (633K)				X			
43530891	Suspected fetal damage from maternal drug use		Other - Specific to initial possibly-pregnant cohort (633K)				X			
2752623	Repair Anal Sphincter, Open Approach		Other - Specific to initial possibly-pregnant cohort (633K)				X			
2756823	Insertion of Contraceptive Device into Left Upper Arm Subcutaneous Tissue and Fascia, Percutaneous Approach		Other - Specific to initial possibly-pregnant cohort (633K)				X			
4085900	Placenta circumvallata		Other - Specific to initial possibly-pregnant cohort (633K)				X			
74104	Fetal condition affecting obstetrical care of mother		Other - Specific to initial possibly-pregnant cohort (633K)				X			
45757356	Pre-existing hypertensive chronic kidney disease in mother complicating pregnancy		Other - Specific to initial possibly-pregnant cohort (633K)				X			
4038496	Labor finding		Other - Specific to initial possibly-pregnant cohort (633K)				X			
43020489	Psychosocial problems related to multiparity		Parity				X			
45773507	Post-term pregnancy of 40 to 42 weeks		Post-term				X			
44784550	Preterm spontaneous labor with preterm delivery		Preterm pregnancy				X			
45770893	Preterm premature rupture of membranes with onset of labor within 24 hours of rupture		Preterm pregnancy				X			
45757175	Preterm labor in second trimester with preterm delivery in second trimester		Preterm pregnancy				X			

36712702	Preterm labor with preterm delivery		Preterm pregnancy				X			
45757148	Preterm premature rupture of membranes with onset of labor later than 24 hours after rupture		Preterm pregnancy				X			
45757176	Preterm labor in third trimester with preterm delivery in third trimester		Preterm pregnancy				X			
4244438	Second trimester pregnancy		Second trimester				X			
3049229	Second trimester quad maternal screen [Interpretation] in Serum or Plasma Narrative		Second trimester				X			
3032802	Second trimester triple maternal screen [Interpretation] in Serum or Plasma Narrative		Second trimester				X			
3032628	Second trimester triple maternal screen panel - Serum or Plasma		Second trimester				X			
2793351	Ultrasonography of Third Trimester, Single Fetus		Singleton pregnancy				X			
4336958	Term pregnancy		Third trimester				X			
2793351	Ultrasonography of Third Trimester, Single Fetus		Third trimester				X			
46273629	Delivery by cesarean section for footling breech presentation		Delivery record only (DELIV)					X		
46273305	Delivery by cesarean section for flexed breech presentation		Delivery record only (DELIV)					X		
46273304	Delivery by cesarean section for breech presentation		Delivery record only (DELIV)					X		
45757174	Vacuum assisted vaginal delivery		Delivery record only (DELIV)			X		X		
44513756	Other specified cephalic vaginal delivery with abnormal presentation of head at delivery without instrument		Delivery record only (DELIV)					X		
44513752	Other specified vacuum delivery		Delivery record only (DELIV)					X		
44513747	Other specified forceps cephalic delivery		Delivery record only (DELIV)					X		
44513746	Low forceps cephalic delivery		Delivery record only (DELIV)					X		
44513745	Mid forceps cephalic delivery NEC		Delivery record only (DELIV)					X		
44513743	High forceps cephalic delivery NEC		Delivery record only (DELIV)					X		
44513740	Other specified other breech delivery		Delivery record only (DELIV)					X		
44513736	Other specified breech extraction delivery		Delivery record only (DELIV)					X		
42535817	Mid vacuum delivery		Delivery record only (DELIV)					X		
42535816	Outlet vacuum delivery		Delivery record only (DELIV)					X		
37310404	Born by mid-cavity forceps delivery		Delivery record only (DELIV)					X		
37310393	Born by low forceps delivery		Delivery record only (DELIV)					X		
37310369	Born by high forceps delivery		Delivery record only (DELIV)					X		
4331179	Vaginal delivery, medical personnel present		Delivery record only (DELIV)					X		
4324549	Forceps delivery with rotation of fetal head		Delivery record only (DELIV)					X		
4294827	Pinard maneuver		Delivery record only (DELIV)					X		
4250441	Kristeller maneuver		Delivery record only (DELIV)					X		
4250009	Born by breech delivery		Delivery record only (DELIV)			X		X		
4244672	Frank breech delivery		Delivery record only (DELIV)					X		
4240325	Bracht maneuver		Delivery record only (DELIV)					X		
4234710	Delivery by Malstrom's extraction		Delivery record only (DELIV)					X		
4234421	Partial breech delivery		Delivery record only (DELIV)					X		
4231702	High forceps delivery		Delivery record only (DELIV)					X		
4230533	Partial breech extraction		Delivery record only (DELIV)					X		
4223638	Delivery by Malstrom's extraction with episiotomy		Delivery record only (DELIV)					X		
4217642	Footling breech delivery		Delivery record only (DELIV)					X		
4217586	Born by forceps delivery		Delivery record only (DELIV)					X		
4204679	Total breech delivery with forceps to aftercoming head		Delivery record only (DELIV)					X		
4170626	Abnormal delivery		Delivery record only (DELIV)					X		
4167018	Vaginal delivery with forceps including postpartum care		Delivery record only (DELIV)					X		
4166775	Brow delivery		Delivery record only (DELIV)					X		
4166247	Total breech extraction		Delivery record only (DELIV)					X		
4164221	Face delivery		Delivery record only (DELIV)					X		
4156948	Delivered by low forceps delivery		Delivery record only (DELIV)					X		
4154615	Deliveries by spontaneous breech delivery		Delivery record only (DELIV)			X		X		
4153284	Delivered by mid-cavity forceps delivery		Delivery record only (DELIV)					X		
4147974	Barton's forceps delivery		Delivery record only (DELIV)					X		
4143647	Midforceps delivery without rotation		Delivery record only (DELIV)					X		
4137400	Breech presentation, no version		Delivery record only (DELIV)					X		
4130319	Delivery of the after coming head		Delivery record only (DELIV)					X		
4128031	Instrumental delivery		Delivery record only (DELIV)					X		
4127705	Term pregnancy delivered		Delivery record only (DELIV)			X		X		
4127251	Mauriceau Smellie Veit maneuver		Delivery record only (DELIV)					X		
4127250	Lovset's maneuver		Delivery record only (DELIV)					X		
4127249	Outlet forceps delivery		Delivery record only (DELIV)					X		
4119050	Born after precipitate delivery		Delivery record only (DELIV)					X		
4118904	Abnormal head presentation delivery		Delivery record only (DELIV)					X		
4118903	Normal delivery - occipitoanterior		Delivery record only (DELIV)					X		
4114636	Breech extraction with internal podalic version		Delivery record only (DELIV)					X		
4106406	Delivery by double application of forceps		Delivery record only (DELIV)					X		
4103850	Prague maneuver		Delivery record only (DELIV)					X		
4102595	Wigand-Martin maneuver		Delivery record only (DELIV)					X		
4100410	Delivery by Scanzoni maneuver		Delivery record only (DELIV)					X		
4096145	Delivery by vacuum extraction with episiotomy		Delivery record only (DELIV)					X		
4093774	Mid forceps delivery with episiotomy		Delivery record only (DELIV)					X		
4083524	Face to pubes birth		Delivery record only (DELIV)					X		
4075735	Low forceps delivery with episiotomy		Delivery record only (DELIV)					X		
4075190	DeLee forceps cephalic delivery with rotation		Delivery record only (DELIV)					X		
4075171	Vacuum delivery before full dilation of cervix		Delivery record only (DELIV)					X		
4075165	Breech extraction delivery with version		Delivery record only (DELIV)					X		
4073438	Piper forceps delivery		Delivery record only (DELIV)					X		
4066113	Deliveries by destructive operation		Delivery record only (DELIV)					X		
4064824	Piper forceps delivery by application to aftercoming head		Delivery record only (DELIV)					X		
4035778	High forceps delivery with episiotomy		Delivery record only (DELIV)					X		
4032766	Burns Marshall maneuver		Delivery record only (DELIV)					X		
4032764	Groin traction at breech delivery		Delivery record only (DELIV)					X		
4032761	Forceps delivery, face to pubes		Delivery record only (DELIV)					X		
4023797	Partial breech delivery with forceps to aftercoming head		Delivery record only (DELIV)					X		
4011047	Postmature pregnancy delivered		Delivery record only (DELIV)					X		
3186670	Threatened premature labor - delivered		Delivery record only (DELIV)					X		
440790	Deliveries by vacuum extractor		Delivery record only (DELIV)					X		
46270991	Emergency lower segment cesarean section with inverted T incision		Delivery record only (DELIV)					X		
44513733	Other specified other caesarean delivery		Delivery record only (DELIV)					X		
44513729	Other specified elective caesarean delivery		Delivery record only (DELIV)					X		
42872493	Cesarean section through J shaped incision of uterus		Delivery record only (DELIV)					X		

42872492	Cesarean section through inverted T shaped incision of uterus		Delivery record only (DELIV)					X		
42537021	Emergency lower segment cesarean section with bilateral tubal ligation		Delivery record only (DELIV)					X		
42536960	Elective lower segment cesarean section with bilateral tubal ligation		Delivery record only (DELIV)			X		X		
42536954	Emergency upper segment cesarean section with bilateral tubal ligation		Delivery record only (DELIV)					X		
42536952	Elective upper segment cesarean section with bilateral tubal ligation		Delivery record only (DELIV)					X		
38001486	Cesarean section w/o CC/MCC		Delivery record only (DELIV)					X		
38001485	Cesarean section w CC/MCC		Delivery record only (DELIV)			X		X		
37110284	Preterm delivery following Cesarean section		Delivery record only (DELIV)					X		
4250010	Born by emergency cesarean section		Delivery record only (DELIV)					X		
4228344	Vaginal cesarean section		Delivery record only (DELIV)					X		
4212794	Born by elective cesarean section		Delivery record only (DELIV)					X		
4172142	Placenta previa found before labor AND delivery by cesarean section without hemorrhage		Delivery record only (DELIV)					X		
4171820	Anesthesia for cesarean section		Delivery record only (DELIV)					X		
4130321	Abdominal delivery for shoulder dystocia		Delivery record only (DELIV)					X		
4127252	Emergency lower segment cesarean section		Delivery record only (DELIV)					X		
4119336	Delivered by cesarean delivery following previous cesarean delivery		Delivery record only (DELIV)					X		
4118802	Delivered by cesarean section - pregnancy at term		Delivery record only (DELIV)					X		
4032767	Emergency upper segment cesarean section		Delivery record only (DELIV)					X		
4066133	Hemorrhoids in pregnancy and the puerperium - delivered with postnatal complication		Delivery record only (DELIV)					X		
4066124	Puerperal septicemia - delivered with postnatal complication		Delivery record only (DELIV)					X		
4066122	Puerperal peritonitis - delivered with postnatal complication		Delivery record only (DELIV)					X		
4065622	Obstetric anesthesia with central nervous system complications - delivered		Delivery record only (DELIV)					X		
4065621	Obstetric anesthesia with cardiac complications - delivered		Delivery record only (DELIV)					X		
4064960	Rupture of uterus during and after labor - delivered with postnatal problem		Delivery record only (DELIV)					X		
4064834	Hypertonic uterine inertia - delivered		Delivery record only (DELIV)					X		
4063028	Glycosuria during pregnancy - delivered		Delivery record only (DELIV)					X		
4062572	Fatigue during pregnancy - delivered with postnatal complication		Delivery record only (DELIV)					X		
4062116	Obstetric anesthesia with pulmonary complications - delivered with postnatal problem		Delivery record only (DELIV)					X		
4062115	Obstetric anesthesia with pulmonary complications - delivered		Delivery record only (DELIV)					X		
4061465	Puerperal salpingitis - delivered with postnatal complication		Delivery record only (DELIV)					X		
4061349	Obstetric anesthesia with central nervous system complication - delivered with postnatal problem		Delivery record only (DELIV)					X		
4061346	Obstetric anesthesia with cardiac complications - delivered with postnatal problem		Delivery record only (DELIV)					X		
4060695	Prolonged artificial rupture of membranes - delivered		Delivery record only (DELIV)					X		
4060675	Fetus with radiation damage - delivered		Delivery record only (DELIV)					X		
4060540	Brow presentation - delivered		Delivery record only (DELIV)					X		
4060301	Herpes gestationis - delivered		Delivery record only (DELIV)					X		
4059757	Oblique lie - delivered		Delivery record only (DELIV)					X		
4058526	Antepartum hemorrhage with uterine leiomyoma - delivered		Delivery record only (DELIV)					X		
4058524	Antepartum hemorrhage with trauma - delivered		Delivery record only (DELIV)					X		
4058111	Glycosuria during pregnancy - delivered with postnatal complication		Delivery record only (DELIV)					X		
4058110	Herpes gestationis - delivered with postnatal complication		Delivery record only (DELIV)					X		
442829	Pelvic soft tissue abnormality in pregnancy, childbirth and the puerperium - delivered		Delivery record only (DELIV)					X		
442072	Obstetric shock - delivered		Delivery record only (DELIV)					X		
442070	Maternal hypotension syndrome - delivered		Delivery record only (DELIV)					X		
436183	Maternal distress - delivered		Delivery record only (DELIV)					X		
435612	Retracted nipple in pregnancy, the puerperium or lactation - delivered		Delivery record only (DELIV)					X		
43018345	Manual Extraction of Products of Conception, Retained, Via Natural or Artificial Opening Endoscopic		Delivery record only (DELIV)					X		
2784574	Extraction of Products of Conception, Retained, Via Natural or Artificial Opening Endoscopic		Delivery record only (DELIV)					X		
2784572	Extraction of Products of Conception, Other, Via Natural or Artificial Opening		Delivery record only (DELIV)			X		X		
2784571	Extraction of Products of Conception, Internal Version, Via Natural or Artificial Opening		Delivery record only (DELIV)			X		X		
2784569	Extraction of Products of Conception, High Forceps, Via Natural or Artificial Opening		Delivery record only (DELIV)					X		
2784568	Extraction of Products of Conception, Mid Forceps, Via Natural or Artificial Opening		Delivery record only (DELIV)			X		X		
2784566	Extraction of Products of Conception, Extraperitoneal, Open Approach		Delivery record only (DELIV)					X		
4113682	Angular pregnancy		Ectopic pregnancy (ECT)					X		
45763635	Aspiration of ectopic pregnancy from cornu of fallopian tube		Ectopic pregnancy (ECT)					X		
42537767	Damage to pelvic organs and tissues following ectopic pregnancy		Ectopic pregnancy (ECT)					X		
42537762	Delayed hemorrhage due to and following ectopic pregnancy		Ectopic pregnancy (ECT)					X		
38001494	Ectopic pregnancy		Ectopic pregnancy (ECT)					X		
37209653	Ectopic pregnancy of left ovary		Ectopic pregnancy (ECT)					X		
37209654	Ectopic pregnancy of right ovary		Ectopic pregnancy (ECT)					X		
42539701	Embolism due to and following ectopic pregnancy		Ectopic pregnancy (ECT)					X		
37118254	Entire product of conception of ectopic pregnancy		Ectopic pregnancy (ECT)					X		
42537763	Excessive hemorrhage due to and following ectopic pregnancy		Ectopic pregnancy (ECT)					X		
40493226	Excision of fallopian tube and surgical removal of ectopic pregnancy		Ectopic pregnancy (ECT)					X		
42536561	Genital tract infection due to and following ectopic pregnancy		Ectopic pregnancy (ECT)					X		
4149029	Intraligamentous pregnancy		Ectopic pregnancy (ECT)					X		
37203955	Intramural ectopic pregnancy of myometrium		Ectopic pregnancy (ECT)					X		

4066151	Intraperitoneal pregnancy		Ectopic pregnancy (ECT)					X		
42872571	Laparoscopic excision of ruptured ectopic tubal pregnancy		Ectopic pregnancy (ECT)					X		
4265032	Laparoscopic treatment of ectopic pregnancy with oophorectomy		Ectopic pregnancy (ECT)					X		
4270068	Laparoscopic treatment of ectopic pregnancy with salpingectomy		Ectopic pregnancy (ECT)					X		
3183328	Left ruptured ectopic pregnancy with hemoperitoneum		Ectopic pregnancy (ECT)					X		
4113218	Mesenteric pregnancy		Ectopic pregnancy (ECT)					X		
4243803	Mesometric pregnancy		Ectopic pregnancy (ECT)					X		
4232708	Partial hysterectomy for removal of cornual ectopic pregnancy		Ectopic pregnancy (ECT)					X		
42536560	Pelvic infection due to and following ectopic pregnancy		Ectopic pregnancy (ECT)					X		
37209655	Pregnancy of left fallopian tube		Ectopic pregnancy (ECT)					X		
37209658	Pregnancy of right fallopian tube		Ectopic pregnancy (ECT)					X		
44792613	Products of ectopic pregnancy tissue specimen		Ectopic pregnancy (ECT)					X		
4017620	Removal of ectopic cervical pregnancy by evacuation		Ectopic pregnancy (ECT)					X		
4220384	Removal of ectopic fetus		Ectopic pregnancy (ECT)			X		X		
4308664	Removal of ectopic fetus from abdominal cavity		Ectopic pregnancy (ECT)					X		
4227423	Removal of ectopic fetus from fallopian tube without salpingectomy		Ectopic pregnancy (ECT)					X		
4247895	Removal of ectopic fetus from ovary without oophorectomy		Ectopic pregnancy (ECT)					X		
4339303	Removal of ectopic interstitial uterine pregnancy requiring total hysterectomy		Ectopic pregnancy (ECT)					X		
4299185	Removal of ectopic pregnancy from fallopian tube		Ectopic pregnancy (ECT)					X		
4300391	Removal of extrauterine ectopic fetus		Ectopic pregnancy (ECT)					X		
4217304	Removal of intraligamentous ectopic pregnancy		Ectopic pregnancy (ECT)					X		
45757120	Renal failure after ectopic pregnancy		Ectopic pregnancy (ECT)					X		
2785345	Resection of Products of Conception, Ectopic, Open Approach		Ectopic pregnancy (ECT)			X		X		
2785346	Resection of Products of Conception, Ectopic, Percutaneous Approach		Ectopic pregnancy (ECT)					X		
2785347	Resection of Products of Conception, Ectopic, Percutaneous Endoscopic Approach		Ectopic pregnancy (ECT)			X		X		
2785348	Resection of Products of Conception, Ectopic, Via Natural or Artificial Opening		Ectopic pregnancy (ECT)					X		
2785349	Resection of Products of Conception, Ectopic, Via Natural or Artificial Opening Endoscopic		Ectopic pregnancy (ECT)					X		
4069106	Ruptured ectopic pregnancy		Ectopic pregnancy (ECT)			X		X		
37209657	Ruptured tubal pregnancy of left fallopian tube		Ectopic pregnancy (ECT)					X		
37209656	Ruptured tubal pregnancy of right fallopian tube		Ectopic pregnancy (ECT)					X		
4165566	Salpingectomy for tubal ectopic pregnancy by abdominal approach		Ectopic pregnancy (ECT)					X		
46269806	Salpingitis due to ectopic pregnancy		Ectopic pregnancy (ECT)					X		
46269807	Sepsis due to ectopic pregnancy		Ectopic pregnancy (ECT)					X		
46269808	Shock due to ectopic pregnancy		Ectopic pregnancy (ECT)					X		
46270313	Structure of product of conception of ectopic pregnancy		Ectopic pregnancy (ECT)					X		
4208611	Tissue specimen from ectopic pregnancy		Ectopic pregnancy (ECT)					X		
4129712	Unruptured tubal pregnancy		Ectopic pregnancy (ECT)					X		
46269809	Urinary tract infection due to ectopic pregnancy		Ectopic pregnancy (ECT)					X		
2784576	Extraction of Products of Conception, Ectopic, Via Natural or Artificial Opening Endoscopic		Ectopic pregnancy (ECT)					X		
2784575	Extraction of Products of Conception, Ectopic, Via Natural or Artificial Opening		Ectopic pregnancy (ECT)					X		
4220085	Gestation period, 2 weeks		Gestation period, X weeks (GW)					X		X
444067	Gestation period, 42 weeks		Gestation period, X weeks (GW)			X		X		X
4290009	Gestation period, 5 weeks		Gestation period, X weeks (GW)					X		X
4326232	Gestation period, 3 weeks		Gestation period, X weeks (GW)					X		X
4270513	Gestation period, 7 weeks		Gestation period, X weeks (GW)			X		X		X
4313026	Gestation period, 6 weeks		Gestation period, X weeks (GW)			X		X		X
4195157	Gestation period, 4 weeks		Gestation period, X weeks (GW)					X		X
4337360	Gestation period, 1 week		Gestation period, X weeks (GW)					X		X
439317	Complete illegal termination of pregnancy with shock		Induced abortion (AB)					X		
439325	Illegal termination of pregnancy, incomplete		Induced abortion (AB)					X		
2784555	Abortion of Products of Conception, Open Approach		Induced abortion (AB)					X		
2784556	Abortion of Products of Conception, Percutaneous Approach		Induced abortion (AB)					X		
2784557	Abortion of Products of Conception, Percutaneous Endoscopic Approach		Induced abortion (AB)					X		
2784558	Abortion of Products of Conception, Vacuum, Via Natural or Artificial Opening		Induced abortion (AB)			X		X		
2784559	Abortion of Products of Conception, Laminaria, Via Natural or Artificial Opening		Induced abortion (AB)			X		X		
2784560	Abortion of Products of Conception, Abortifacient, Via Natural or Artificial Opening		Induced abortion (AB)			X		X		
2784561	Abortion of Products of Conception, Via Natural or Artificial Opening		Induced abortion (AB)			X		X		
2784562	Abortion of Products of Conception, Via Natural or Artificial Opening Endoscopic		Induced abortion (AB)					X		
4009642	Legal termination of pregnancy with air embolism		Induced abortion (AB)					X		
4023781	Illegal termination of pregnancy with endometritis		Induced abortion (AB)					X		
4025639	Legal termination of pregnancy with cardiac arrest and/or failure		Induced abortion (AB)					X		
4025828	Illegal termination of pregnancy with laceration of periurethral tissue		Induced abortion (AB)					X		
4030009	Therapeutic termination of pregnancy by insertion of laminaria		Induced abortion (AB)					X		
4034753	Illegal termination of pregnancy with sepsis		Induced abortion (AB)					X		
4045581	Legal termination of pregnancy with laceration of uterus		Induced abortion (AB)					X		
4046510	Legal termination of pregnancy with intravascular hemolysis		Induced abortion (AB)					X		
4049453	Legal termination of pregnancy with parametritis		Induced abortion (AB)					X		
4051784	Illegal termination of pregnancy with perforation of bowel		Induced abortion (AB)					X		

4058932	Legal termination of pregnancy with postoperative shock		Induced abortion (AB)					X		
4066227	Illegal termination of pregnancy with blood-clot embolism		Induced abortion (AB)					X		
4066994	Illegal termination of pregnancy with laceration of bowel		Induced abortion (AB)					X		
4068253	Legal termination of pregnancy with perforation of uterus		Induced abortion (AB)					X		
4070939	Legal termination of pregnancy with renal tubular necrosis		Induced abortion (AB)					X		
4077275	Illegal termination of pregnancy with laceration of cervix		Induced abortion (AB)					X		
4077454	Legal termination of pregnancy with cerebral anoxia		Induced abortion (AB)					X		
4094081	Legal termination of pregnancy with pelvic peritonitis		Induced abortion (AB)					X		
4098441	Legal termination of pregnancy with amniotic fluid embolism		Induced abortion (AB)					X		
4101477	Illegal termination of pregnancy with electrolyte imbalance		Induced abortion (AB)					X		
4103148	Illegal termination of pregnancy with perforation of vagina		Induced abortion (AB)					X		
4105063	Legal termination of pregnancy with septic shock		Induced abortion (AB)					X		
4105762	Illegal termination of pregnancy with laceration of broad ligament		Induced abortion (AB)					X		
4106892	Legal termination of pregnancy with septic embolism		Induced abortion (AB)					X		
4107907	Illegal termination of pregnancy with fat embolism		Induced abortion (AB)					X		
4115153	Medical termination of pregnancy		Induced abortion (AB)			X		X		
4119975	Operative termination of pregnancy		Induced abortion (AB)					X		
4121775	Injection of amnion for termination of pregnancy		Induced abortion (AB)					X		
4138267	Illegal termination of pregnancy with afibrinogenemia		Induced abortion (AB)					X		
4140509	Illegal termination of pregnancy with urinary tract infection		Induced abortion (AB)					X		
4143018	Illegal termination of pregnancy with postoperative shock		Induced abortion (AB)					X		
4143190	Illegal termination of pregnancy with acute renal failure		Induced abortion (AB)					X		
4147533	Legal termination of pregnancy with afibrinogenemia		Induced abortion (AB)					X		
4150823	Self-induced termination of pregnancy		Induced abortion (AB)					X		
4178578	Legal termination of pregnancy with pulmonary embolism		Induced abortion (AB)					X		
4180859	Illegal termination of pregnancy with laceration of bladder		Induced abortion (AB)					X		
4181114	Legal termination of pregnancy with acute renal failure		Induced abortion (AB)					X		
4182549	Illegal termination of pregnancy with cardiac arrest AND/OR failure		Induced abortion (AB)					X		
4183557	Illegal termination of pregnancy with uremia		Induced abortion (AB)					X		
4183680	Legal termination of pregnancy with laceration of bowel		Induced abortion (AB)					X		
4184150	Legal termination of pregnancy with perforation of bowel		Induced abortion (AB)					X		
4185440	Illegal termination of pregnancy with air embolism		Induced abortion (AB)					X		
4188764	Legal termination of pregnancy with sepsis		Induced abortion (AB)					X		
4195243	Illegal termination of pregnancy with perforation of broad ligament		Induced abortion (AB)					X		
4195297	Illegal termination of pregnancy with renal tubular necrosis		Induced abortion (AB)					X		
4197681	Legal termination of pregnancy with salpingo-oophoritis		Induced abortion (AB)					X		
4197900	Illegal termination of pregnancy with perforation of bladder		Induced abortion (AB)					X		
4198404	Legal termination of pregnancy with perforation of broad ligament		Induced abortion (AB)					X		
4200986	Induced termination of pregnancy following intra-amniotic injection with hysterotomy		Induced abortion (AB)					X		
4204206	Legal termination of pregnancy with salpingitis		Induced abortion (AB)					X		
4204231	Legal termination of pregnancy with laceration of vagina		Induced abortion (AB)					X		
4204840	Therapeutic termination of pregnancy		Induced abortion (AB)					X		
4206453	Illegal termination of pregnancy with pulmonary embolism		Induced abortion (AB)					X		
4212346	Legal termination of pregnancy with acute necrosis of liver		Induced abortion (AB)					X		
4214233	Legal termination of pregnancy with electrolyte imbalance		Induced abortion (AB)					X		
4216436	Legal termination of pregnancy with laceration of broad ligament		Induced abortion (AB)					X		
4219608	Illegal termination of pregnancy with septic shock		Induced abortion (AB)					X		
4223627	Illegal termination of pregnancy with cerebral anoxia		Induced abortion (AB)					X		
4224143	Illegal termination of pregnancy with perforation of cervix		Induced abortion (AB)					X		
4225572	Legal termination of pregnancy with uremia		Induced abortion (AB)					X		
4229716	Illegal termination of pregnancy with laceration of uterus		Induced abortion (AB)					X		
4231060	Termination of pregnancy contraindicated		Induced abortion (AB)					X		
4234979	Illegal termination of pregnancy with pelvic peritonitis		Induced abortion (AB)					X		
4236326	Illegal termination of pregnancy with acute necrosis of liver		Induced abortion (AB)					X		
4239192	Illegal termination of pregnancy with perforation of periurethral tissue		Induced abortion (AB)					X		
4239693	Termination of pregnancy on demand		Induced abortion (AB)					X		
4243658	Legal termination of pregnancy with laceration of bladder		Induced abortion (AB)					X		
4245041	Legal termination of pregnancy with urinary tract infection		Induced abortion (AB)					X		
4249728	Illegal termination of pregnancy with laceration of vagina		Induced abortion (AB)					X		
4257043	Extra-amniotic termination of pregnancy		Induced abortion (AB)					X		
4264914	Illegal termination of pregnancy with defibrination syndrome		Induced abortion (AB)					X		
4266243	Illegal termination of pregnancy with oliguria		Induced abortion (AB)					X		
4269450	Legal termination of pregnancy with oliguria		Induced abortion (AB)					X		
4271203	Legal termination of pregnancy with perforation of bladder		Induced abortion (AB)					X		
4280224	Legal termination of pregnancy with defibrination syndrome		Induced abortion (AB)					X		
4292089	Legal termination of pregnancy with blood-clot embolism		Induced abortion (AB)					X		
4294829	Legal termination of pregnancy with endometritis		Induced abortion (AB)					X		

4296459	Legal termination of pregnancy with perforation of periurethral tissue		Induced abortion (AB)					X		
4296872	Illegal termination of pregnancy with perforation of uterus		Induced abortion (AB)					X		
4303980	Illegal termination of pregnancy with amniotic fluid embolism		Induced abortion (AB)					X		
4305348	Illegal termination of pregnancy with parametritis		Induced abortion (AB)					X		
4312358	Legal termination of pregnancy with perforation of cervix		Induced abortion (AB)					X		
4313005	Illegal termination of pregnancy with salpingo-oophoritis		Induced abortion (AB)					X		
4318662	Illegal termination of pregnancy with salpingitis		Induced abortion (AB)					X		
4323434	Illegal termination of pregnancy with intravascular hemolysis		Induced abortion (AB)					X		
4324672	Legal termination of pregnancy with laceration of periurethral tissue		Induced abortion (AB)					X		
4324695	Legal termination of pregnancy with laceration of cervix		Induced abortion (AB)					X		
4324965	Illegal termination of pregnancy with septic embolism		Induced abortion (AB)					X		
4327321	Legal termination of pregnancy with perforation of vagina		Induced abortion (AB)					X		
4328084	Legal termination of pregnancy with fat embolism		Induced abortion (AB)					X		
37018340	Induced termination of pregnancy		Induced abortion (AB)					X		
37206226	Excessive hemorrhage due to and following induced termination of pregnancy		Induced abortion (AB)					X		
37206227	Secondary hemorrhage due to and following induced termination of pregnancy		Induced abortion (AB)					X		
37206228	Secondary hemorrhage due to and following illegally induced termination of pregnancy		Induced abortion (AB)					X		
37206229	Secondary hemorrhage due to and following legally induced termination of pregnancy		Induced abortion (AB)			X		X		
37206230	Excessive hemorrhage due to and following legally induced termination of pregnancy		Induced abortion (AB)					X		
37206231	Excessive hemorrhage due to and following illegally induced termination of pregnancy		Induced abortion (AB)					X		
40487510	Medical termination of pregnancy using prostaglandin		Induced abortion (AB)					X		
43020954	Termination of pregnancy after first trimester		Induced abortion (AB)					X		
43530907	Termination of pregnancy with complication		Induced abortion (AB)			X		X		
43530908	Induced termination of pregnancy complicated by damage to pelvic organs and/or tissues		Induced abortion (AB)					X		
43530910	Induced termination of pregnancy complicated by genital-pelvic infection		Induced abortion (AB)					X		
43530911	Induced termination of pregnancy complicated by metabolic disorder		Induced abortion (AB)					X		
43530912	Induced termination of pregnancy complicated by renal failure		Induced abortion (AB)					X		
43530913	Induced termination of pregnancy complicated by acute necrosis of liver		Induced abortion (AB)					X		
43530914	Induced termination of pregnancy complicated by acute renal failure		Induced abortion (AB)					X		
43530915	Induced termination of pregnancy complicated by afibrinogenemia		Induced abortion (AB)					X		
43530916	Induced termination of pregnancy complicated by air embolism		Induced abortion (AB)					X		
43530917	Induced termination of pregnancy complicated by blood-clot embolism		Induced abortion (AB)					X		
43530918	Induced termination of pregnancy complicated by cardiac arrest and/or failure		Induced abortion (AB)					X		
43530919	Induced termination of pregnancy complicated by cerebral anoxia		Induced abortion (AB)					X		
43530920	Induced termination of pregnancy complicated by defibrination syndrome		Induced abortion (AB)					X		
43530921	Induced termination of pregnancy complicated by endometritis		Induced abortion (AB)					X		
43530922	Induced termination of pregnancy complicated by fat embolism		Induced abortion (AB)					X		
43530923	Induced termination of pregnancy complicated by intravascular hemolysis		Induced abortion (AB)					X		
43530924	Induced termination of pregnancy complicated by laceration of bowel		Induced abortion (AB)					X		
43530925	Induced termination of pregnancy complicated by laceration of broad ligament		Induced abortion (AB)					X		
43530926	Induced termination of pregnancy complicated by laceration of uterus		Induced abortion (AB)					X		
43530927	Induced termination of pregnancy complicated by laceration of vagina		Induced abortion (AB)					X		
43530928	Induced termination of pregnancy complicated by acute renal failure with oliguria		Induced abortion (AB)					X		
43530929	Induced termination of pregnancy complicated by parametritis		Induced abortion (AB)					X		
43530930	Induced termination of pregnancy complicated by perforation of bowel		Induced abortion (AB)					X		
43530931	Induced termination of pregnancy complicated by perforation of cervix		Induced abortion (AB)					X		
43530932	Induced termination of pregnancy complicated by perforation of uterus		Induced abortion (AB)					X		
43530933	Induced termination of pregnancy complicated by perforation of vagina		Induced abortion (AB)					X		
43530934	Induced termination of pregnancy complicated by pulmonary embolism		Induced abortion (AB)					X		
43530935	Induced termination of pregnancy complicated by renal tubular necrosis		Induced abortion (AB)					X		
43530936	Induced termination of pregnancy complicated by salpingitis		Induced abortion (AB)					X		
43530937	Induced termination of pregnancy complicated by salpingo-oophoritis		Induced abortion (AB)					X		
43530939	Induced termination of pregnancy complicated by sepsis		Induced abortion (AB)					X		
43530940	Induced termination of pregnancy complicated by septic embolism		Induced abortion (AB)					X		
43530941	Induced termination of pregnancy complicated by septic shock		Induced abortion (AB)					X		
43530943	Induced termination of pregnancy complicated by soap embolism		Induced abortion (AB)					X		
43530944	Induced termination of pregnancy complicated by uremia		Induced abortion (AB)					X		
43530945	Induced termination of pregnancy complicated by urinary tract infection		Induced abortion (AB)					X		

43530946	Termination of pregnancy without complication		Induced abortion (AB)					X		
43530947	Induced termination of pregnancy complicated by tetanus		Induced abortion (AB)					X		
43530948	Induced termination of pregnancy complicated by pelvic disorder		Induced abortion (AB)					X		
43530952	Induced termination of pregnancy complicated by laceration of bladder		Induced abortion (AB)					X		
43530953	Induced termination of pregnancy complicated by laceration of periurethral tissue		Induced abortion (AB)					X		
43530954	Induced termination of pregnancy complicated by perforation of bladder		Induced abortion (AB)					X		
43530955	Induced termination of pregnancy complicated by perforation of broad ligament		Induced abortion (AB)					X		
43530956	Induced termination of pregnancy complicated by perforation of periurethral tissue		Induced abortion (AB)					X		
43530957	Induced termination of pregnancy complicated by bladder damage		Induced abortion (AB)					X		
43530958	Induced termination of pregnancy complicated by bowel damage		Induced abortion (AB)					X		
43530959	Induced termination of pregnancy complicated by broad ligament damage		Induced abortion (AB)					X		
43530960	Induced termination of pregnancy complicated by cardiac arrest		Induced abortion (AB)					X		
43530961	Induced termination of pregnancy complicated by cardiac failure		Induced abortion (AB)					X		
43530962	Induced termination of pregnancy complicated by cervix damage		Induced abortion (AB)					X		
43530964	Induced termination of pregnancy complicated by infectious disease		Induced abortion (AB)					X		
43530965	Induced termination of pregnancy complicated by periurethral tissue damage		Induced abortion (AB)					X		
43530966	Induced termination of pregnancy complicated by oliguria		Induced abortion (AB)					X		
43530967	Induced termination of pregnancy complicated by uterus damage		Induced abortion (AB)					X		
43530968	Induced termination of pregnancy complicated by vaginal damage		Induced abortion (AB)					X		
43531709	Retained products of conception following induced termination of pregnancy		Induced abortion (AB)					X		
43531710	Induced termination of pregnancy complicated by embolism		Induced abortion (AB)					X		
43531711	Induced termination of pregnancy complicated by shock		Induced abortion (AB)					X		
43531712	Induced termination of pregnancy complicated by amniotic fluid embolism		Induced abortion (AB)					X		
43531713	Induced termination of pregnancy complicated by electrolyte imbalance		Induced abortion (AB)					X		
43531714	Induced termination of pregnancy complicated by laceration of cervix		Induced abortion (AB)					X		
43531715	Induced termination of pregnancy complicated by pelvic peritonitis		Induced abortion (AB)					X		
43531716	Induced termination of pregnancy complicated by postoperative shock		Induced abortion (AB)					X		
44513476	Other specified introduction of abortifacient into uterine cavity		Induced abortion (AB)					X		
44784128	Induced termination of pregnancy under unsafe conditions		Induced abortion (AB)					X		
44805020	Incomplete termination of pregnancy		Induced abortion (AB)					X		
45763590	Induced termination of pregnancy in first trimester		Induced abortion (AB)					X		
45763591	Induced termination of pregnancy in second trimester		Induced abortion (AB)					X		
45768717	Incomplete induced termination of pregnancy		Induced abortion (AB)					X		
45771353	Incomplete induced termination of pregnancy with complication		Induced abortion (AB)					X		
45883489	Induced abortion		Induced abortion (AB)					X		
44784482	Complete legal abortion with complication		Induced abortion (AB)					X		
44784433	Complete legal abortion complicated by excessive hemorrhage		Induced abortion (AB)					X		
44782485	Complete illegal abortion complicated by excessive hemorrhage		Induced abortion (AB)					X		
37110280	Incomplete legal abortion without complication		Induced abortion (AB)			X		X		
3174212	Premature 28 week quadruplet		Livebirth (LB)					X		
4008884	Premature birth of newborn sextuplets		Livebirth (LB)					X		
4015421	Triplets - two live and one stillborn		Livebirth (LB)					X		
4015422	Triplets - one live and two stillborn		Livebirth (LB)					X		
4029786	Premature birth of newborn twins		Livebirth (LB)					X		
4049333	Term birth of identical twins, both living		Livebirth (LB)					X		
4052512	Term birth of newborn male		Livebirth (LB)			X		X		
4054968	Term birth of newborn		Livebirth (LB)			X		X		
4066292	Term birth of multiple newborns		Livebirth (LB)					X		
4069200	Premature birth of multiple newborns		Livebirth (LB)					X		
4082863	Premature birth of newborn quintuplets		Livebirth (LB)					X		
4097427	Premature birth of fraternal twins, both living		Livebirth (LB)					X		
4107401	Premature birth of newborn triplets		Livebirth (LB)					X		
4142021	Premature birth of identical twins, both living		Livebirth (LB)					X		
4145125	Premature birth of newborn quadruplets		Livebirth (LB)					X		
4147043	Premature birth of fraternal twins, one living, one stillborn		Livebirth (LB)					X		
4172135	Premature birth of newborn male		Livebirth (LB)					X		
4178275	Term birth of newborn twins		Livebirth (LB)					X		
4193224	Term birth of newborn quadruplets		Livebirth (LB)					X		
4199146	Term birth of newborn sextuplets		Livebirth (LB)					X		
4227728	Term birth of fraternal twins, both living		Livebirth (LB)					X		
4241228	Premature birth of newborn female		Livebirth (LB)					X		
4243026	Term birth of identical twins, one living, one stillborn		Livebirth (LB)					X		
4246654	Term birth of newborn female		Livebirth (LB)			X		X		
4272248	Premature birth of newborn		Livebirth (LB)			X		X		
4307237	Term birth of newborn triplets		Livebirth (LB)					X		
4310910	Premature birth of identical twins, one living, one stillborn		Livebirth (LB)					X		
4330570	Term birth of fraternal twins, one living, one stillborn		Livebirth (LB)					X		
4336090	Term birth of newborn quintuplets		Livebirth (LB)					X		
42535052	Twin live born in hospital by vaginal delivery		Livebirth (LB)					X		
45757165	Triplets, some live born		Livebirth (LB)					X		
45757166	Quadruplets, all live born		Livebirth (LB)					X		
45757167	Quintuplets, all live born		Livebirth (LB)					X		

45757168	Quintuplets, some live born		Livebirth (LB)					X		
45757169	Sextuplets, some live born		Livebirth (LB)					X		
45765500	Quadruplet birth		Livebirth (LB)					X		
45765501	Quintuplet birth		Livebirth (LB)					X		
45765502	Sextuplet birth		Livebirth (LB)					X		
45772082	Sextuplets, all live born		Livebirth (LB)					X		
45773428	Quadruplets, some live born		Livebirth (LB)					X		
42539267	Multiple liveborn in hospital by vaginal delivery		Livebirth (LB)					X		
42539210	Triplet liveborn in hospital by cesarean section		Livebirth (LB)					X		
40483521	Single liveborn born in hospital by cesarean section		Livebirth (LB)			X		X		
40483126	Liveborn born in hospital by cesarean section		Livebirth (LB)			X		X		
40483101	Twin liveborn born in hospital by cesarean section		Livebirth (LB)			X		X		
40483084	Twin liveborn born in hospital		Livebirth (LB)					X		
40482735	Liveborn born in hospital		Livebirth (LB)			X		X		
36717637	Twin liveborn born outside hospital		Livebirth (LB)					X		
36713469	Multiple liveborn other than twins born outside hospital		Livebirth (LB)					X		
36713468	Multiple liveborn other than twins born in hospital		Livebirth (LB)					X		
36713467	Singleton liveborn unspecified as to place of birth		Livebirth (LB)			X		X		
36713466	Singleton liveborn born outside hospital		Livebirth (LB)					X		
36713465	Singleton liveborn born in hospital		Livebirth (LB)			X		X		
4146775	Incomplete inevitable miscarriage without complication		Spontaneous abortion (SA)	X	X	X	X	X		
4149423	Miscarriage with endometritis		Spontaneous abortion (SA)					X		
4149994	Readmission for abortive pregnancy (NHS codes)	SA	Spontaneous abortion (SA)	X						
4150538	Suction evacuation of retained products of conception	SA	Spontaneous abortion (SA)	X						
4153287	Removal of products of conception from fallopian tube	SA	Spontaneous abortion (SA)	X						
4168445	Miscarriage with postoperative shock		Spontaneous abortion (SA)					X		
4170121	Retained products after miscarriage	SA	Spontaneous abortion (SA)	X	X	X		X		
4170151	Evacuation of uterus	SA	Spontaneous abortion (SA)	X						
4170457	Miscarriage with heavy bleeding		Spontaneous abortion (SA)	X				X		
4175137	Miscarriage with cardiac arrest and/or cardiac failure		Spontaneous abortion (SA)					X		
4179796	Miscarriage with defibrination syndrome		Spontaneous abortion (SA)					X		
4180568	Miscarriage with intravascular hemolysis		Spontaneous abortion (SA)					X		
4189350	Miscarriage with salpingo-oophoritis		Spontaneous abortion (SA)					X		
4194113	Miscarriage with laceration of bladder		Spontaneous abortion (SA)					X		
4194423	Post miscarriage counselling	SA	Spontaneous abortion (SA)	X						
4195406	Miscarriage with salpingitis		Spontaneous abortion (SA)					X		
4200070	Miscarriage with perforation of periurethral tissue		Spontaneous abortion (SA)					X		
4202200	Surgical treatment of missed miscarriage of second trimester		Spontaneous abortion (SA)					X		
4209126	Miscarriage with blood-clot embolism		Spontaneous abortion (SA)					X		
4211863	Miscarriage with acute renal failure		Spontaneous abortion (SA)					X		
4216342	Miscarriage with perforation of bowel		Spontaneous abortion (SA)					X		
4217670	Miscarriage with oliguria		Spontaneous abortion (SA)					X		
4219469	Miscarriage with pulmonary embolism		Spontaneous abortion (SA)					X		
4221853	Miscarriage with parametritis		Spontaneous abortion (SA)					X		
4224597	Miscarriage with acute necrosis of liver		Spontaneous abortion (SA)					X		
4224646	Miscarriage in second trimester		Spontaneous abortion (SA)					X		
4238207	Listeria miscarriage		Spontaneous abortion (SA)					X		
4244261	Miscarriage with septic embolism		Spontaneous abortion (SA)					X		
4244408	Inevitable miscarriage	SA	Spontaneous abortion (SA)	X				X		
4247270	Miscarriage with air embolism		Spontaneous abortion (SA)					X		
4253804	Miscarriage with perforation of cervix		Spontaneous abortion (SA)					X		
4261327	Miscarriage with laceration of periurethral tissue		Spontaneous abortion (SA)					X		
4261479	Carneous mole	SA	Spontaneous abortion (SA)	X						
4262136	Blighted ovum	SA	Spontaneous abortion (SA)	X	X					
4269081	Surgical treatment of miscarriage of any trimester		Spontaneous abortion (SA)					X		
4274315	Miscarriage with electrolyte imbalance		Spontaneous abortion (SA)					X		
4278107	Miscarriage with fat embolism		Spontaneous abortion (SA)					X		
4285746	Miscarriage with sepsis		Spontaneous abortion (SA)	X	X	X		X		
4302162	Miscarriage with laceration of uterus		Spontaneous abortion (SA)					X		
4309780	Miscarriage with laceration of broad ligament		Spontaneous abortion (SA)					X		
4310654	Miscarriage with perforation of bladder		Spontaneous abortion (SA)					X		
4311698	Miscarriage with laceration of vagina		Spontaneous abortion (SA)					X		
4313166	Miscarriage with pelvic peritonitis		Spontaneous abortion (SA)					X		
4323612	Miscarriage due to Brucella abortus		Spontaneous abortion (SA)					X		
4339107	Miscarriage with perforation of vagina		Spontaneous abortion (SA)					X		
40318617	Incomplete miscarriage		Spontaneous abortion (SA)			X		X		
40318618	Complete miscarriage		Spontaneous abortion (SA)			X		X		
40482050	Chemical pregnancy		Spontaneous abortion (SA)					X		
40767416	Was your pregnancy a live birth, stillbirth, miscarriage, abortion, or ectopic pregnancy PhenX	SA	Spontaneous abortion (SA)	X						
42535215	Incomplete spontaneous abortion due to hemorrhage		Spontaneous abortion (SA)					X		
42537759	Delayed hemorrhage due to and following miscarriage		Spontaneous abortion (SA)					X		
42537769	Disorder of vein following miscarriage		Spontaneous abortion (SA)					X		
42739743	Anesthesia for abortion procedures	SA	Spontaneous abortion (SA)	X						
45757198	Sepsis due to incomplete miscarriage		Spontaneous abortion (SA)			X		X		
45876808	Miscarriage		Spontaneous abortion (SA)					X		
46269746	Female genital tract infection due to complete miscarriage		Spontaneous abortion (SA)					X		
46269812	Cardiac arrest due to miscarriage		Spontaneous abortion (SA)					X		
46269813	Urinary tract infection due to incomplete miscarriage		Spontaneous abortion (SA)					X		
46274066	Cardiac arrest due to incomplete miscarriage		Spontaneous abortion (SA)					X		
42539377	Quadruplets with all four stillborn		Stillbirth (SB)					X		
42538806	Sextuplets with all six stillborn		Stillbirth (SB)					X		
42538805	Quintuplets with all five stillborn		Stillbirth (SB)					X		
4306288	Term birth of fraternal twins, both stillborn		Stillbirth (SB)					X		
4242724	Premature birth of identical twins, both stillborn		Stillbirth (SB)					X		
4222915	Premature birth of fraternal twins, both stillborn		Stillbirth (SB)					X		
4203001	Term birth of stillborn twins		Stillbirth (SB)					X		
4192641	Term birth of identical twins, both stillborn		Stillbirth (SB)					X		
4184594	Premature birth of stillborn twins		Stillbirth (SB)					X		
4015163	Triplets - three stillborn		Stillbirth (SB)					X		
45773593	Intrapartum stillbirth		Stillbirth (SB)					X		
37017027	Antepartum stillbirth		Stillbirth (SB)					X		
4321563	Stillbirth of mature female		Stillbirth (SB)					X		
4301269	Stillbirth of mature male		Stillbirth (SB)					X		
4300979	Stillbirth of premature male		Stillbirth (SB)					X		
4287409	Stillbirth of immature female		Stillbirth (SB)					X		
4261819	Stillbirth of immature male		Stillbirth (SB)					X		

4217655	Stillbirth of immature fetus, sex undetermined		Stillbirth (SB)					X		
4103876	Stillbirth of premature female		Stillbirth (SB)					X		
4034159	Fresh stillbirth		Stillbirth (SB)					X		
4028787	Macerated stillbirth		Stillbirth (SB)					X		
443463	Stillbirth - unknown if fetal death intrapartum or prior to labor		Stillbirth (SB)					X		
46273629	Delivery by cesarean section for footling breech presentation		Cesarean section							
46273305	Delivery by cesarean section for flexed breech presentation		Cesarean section							
46273304	Delivery by cesarean section for breech presentation		Cesarean section							
46270991	Emergency lower segment cesarean section with inverted T incision		Cesarean section							
44513733	Other specified other caesarean delivery		Cesarean section							
44513729	Other specified elective caesarean delivery		Cesarean section							
42872493	Cesarean section through J shaped incision of uterus		Cesarean section							
42872492	Cesarean section through inverted T shaped incision of uterus		Cesarean section							
42539210	Triplet liveborn in hospital by cesarean section		Cesarean section							
42537021	Emergency lower segment cesarean section with bilateral tubal ligation		Cesarean section							
42536960	Elective lower segment cesarean section with bilateral tubal ligation		Cesarean section			X				
42536954	Emergency upper segment cesarean section with bilateral tubal ligation		Cesarean section							
42536952	Elective upper segment cesarean section with bilateral tubal ligation		Cesarean section							
40483521	Single liveborn born in hospital by cesarean section		Cesarean section			X				
40483126	Liveborn born in hospital by cesarean section		Cesarean section			X				
40483101	Twin liveborn born in hospital by cesarean section		Cesarean section			X				
38001486	Cesarean section w/o CC/MCC		Cesarean section							
38001485	Cesarean section w CC/MCC		Cesarean section			X				
37110284	Preterm delivery following Cesarean section		Cesarean section							
4250010	Born by emergency cesarean section		Cesarean section							
4228344	Vaginal cesarean section		Cesarean section							
4212794	Born by elective cesarean section		Cesarean section							
4172142	Placenta previa found before labor AND delivery by cesarean section without hemorrhage		Cesarean section							
4171820	Anesthesia for cesarean section		Cesarean section							
4130321	Abdominal delivery for shoulder dystocia		Cesarean section							
4127252	Emergency lower segment cesarean section		Cesarean section							
4119336	Delivered by cesarean delivery following previous cesarean delivery		Cesarean section							
4118802	Delivered by cesarean section - pregnancy at term		Cesarean section							
4032767	Emergency upper segment cesarean section		Cesarean section							
37311061	COVID-19		COVID-19 diagnosis (U07.1)							
4052536	Extracorporeal membrane oxygenation		ECMO							
37206601	Venoarterial extracorporeal membrane oxygenation		ECMO							
46257400	Insertion of left heart vent by thoracic incision (eg, sternotomy, thoracotomy) for ECMO/ECLS		ECMO							
46257438	Extracorporeal membrane oxygenation (ECMO) /extracorporeal life support (ECLS) provided by physician; insertion of peripheral (arterial and/or venous) cannula(e), open, 6 years and older		ECMO							
46257440	Extracorporeal membrane oxygenation (ECMO) /extracorporeal life support (ECLS) provided by physician; reposition central cannula(e) by sternotomy or thoracotomy, 6 years and older (includes fluoroscopic guidance, when performed)		ECMO							
46257466	Extracorporeal membrane oxygenation (ECMO) /extracorporeal life support (ECLS) provided by physician; insertion of peripheral (arterial and/or venous) cannula(e), percutaneous, 6 years and older (includes fluoroscopic guidance, when performed)		ECMO							
46257468	Extracorporeal membrane oxygenation (ECMO) /extracorporeal life support (ECLS) provided by physician; removal of peripheral (arterial and/or venous) cannula(e), percutaneous, 6 years and older		ECMO							
46257510	Extracorporeal membrane oxygenation (ECMO) /extracorporeal life support (ECLS) provided by physician; daily management, each day, veno-venous		ECMO							
46257511	Extracorporeal membrane oxygenation (ECMO) /extracorporeal life support (ECLS) provided by physician; daily management, each day, veno-arterial		ECMO							
46257543	Extracorporeal membrane oxygenation (ECMO) /extracorporeal life support (ECLS) provided by physician; initiation, veno-venous		ECMO							
46257585	Extracorporeal membrane oxygenation (ECMO) /extracorporeal life support (ECLS) provided by physician		ECMO							
46257730	Extracorporeal membrane oxygenation (ECMO) /extracorporeal life support (ECLS) provided by physician; removal of peripheral (arterial and/or venous) cannula(e), percutaneous, birth through 5 years of age		ECMO							
1531631	Extracorporeal Oxygenation, Membrane, Peripheral Veno-arterial		ECMO							
46257680	Extracorporeal membrane oxygenation (ECMO) /extracorporeal life support (ECLS) provided by physician; removal of central cannula(e) by sternotomy or thoracotomy, 6 years and older		ECMO							
46257685	Extracorporeal membrane oxygenation (ECMO) /extracorporeal life support (ECLS) provided by physician; removal of central cannula(e) by sternotomy or thoracotomy, birth through 5 years of age		ECMO							
37206603	Venovenous extracorporeal membrane oxygenation		ECMO							
46257398	Extracorporeal membrane oxygenation (ECMO) /extracorporeal life support (ECLS) provided by physician; insertion of central cannula(e) by sternotomy or thoracotomy, 6 years and older		ECMO							
46257512	Extracorporeal membrane oxygenation (ECMO) /extracorporeal life support (ECLS) provided by physician; insertion of peripheral (arterial and/or venous) cannula(e), percutaneous, birth through 5 years of age (includes fluoroscopic guidance, when performed)		ECMO							

2002247	Extracorporeal membrane oxygenation [ECMO]		ECMO							
2787820	Extracorporeal Supersaturated Oxygenation, Intermittent		ECMO							
4338595	Cardiac support using extracorporeal membrane oxygenation circuitry		ECMO							
44515635	Extracorporeal membrane oxygenation		ECMO							
46257439	Extracorporeal membrane oxygenation (ECMO) /extracorporeal life support (ECLS) provided by physician; reposition of central cannula(e) by sternotomy or thoracotomy, birth through 5 years of age (includes fluoroscopic guidance, when performed)		ECMO							
46257469	Removal of left heart vent by thoracic incision (eg, sternotomy, thoracotomy) for ECMO/ECLS		ECMO							
46257513	Extracorporeal membrane oxygenation (ECMO) /extracorporeal life support (ECLS) provided by physician; reposition peripheral (arterial and/or venous) cannula(e), percutaneous, birth through 5 years of age (includes fluoroscopic guidance, when performed)		ECMO							
46257684	Extracorporeal membrane oxygenation (ECMO) /extracorporeal life support (ECLS) provided by physician; reposition peripheral (arterial and/or venous) cannula(e), open, 6 years and older (includes fluoroscopic guidance, when performed)		ECMO							
1531630	Extracorporeal Oxygenation, Membrane, Peripheral Veno-venous		ECMO							
46257397	Extracorporeal membrane oxygenation (ECMO) /extracorporeal life support (ECLS) provided by physician; insertion of central cannula(e) by sternotomy or thoracotomy, birth through 5 years of age		ECMO							
46257441	Arterial exposure with creation of graft conduit (eg, chimney graft) to facilitate arterial perfusion for ECMO/ECLS (List separately in addition to code for primary procedure)		ECMO							
46257586	Extracorporeal Membrane Oxygenation or Extracorporeal Life Support Services and Procedures		ECMO							
1531632	Extracorporeal Oxygenation, Membrane, Central		ECMO							
2787821	Extracorporeal Hyperbaric Oxygenation, Continuous		ECMO							
37206602	Arteriovenous extracorporeal membrane oxygenation		ECMO							
44811012	Fluoroscopy guided percutaneous insertion of cannula for extracorporeal membrane oxygenation		ECMO							
46257399	Extracorporeal membrane oxygenation (ECMO) /extracorporeal life support (ECLS) provided by physician; removal of peripheral (arterial and/or venous) cannula(e), open, 6 years and older		ECMO							
46257467	Extracorporeal membrane oxygenation (ECMO) /extracorporeal life support (ECLS) provided by physician; reposition peripheral (arterial and/or venous) cannula(e), percutaneous, 6 years and older (includes fluoroscopic guidance, when performed)		ECMO							
46257544	Extracorporeal membrane oxygenation (ECMO) /extracorporeal life support (ECLS) provided by physician; removal of peripheral (arterial and/or venous) cannula(e), open, birth through 5 years of age		ECMO							
46257682	Extracorporeal membrane oxygenation (ECMO) /extracorporeal life support (ECLS) provided by physician; initiation, veno-arterial		ECMO							
46257683	Extracorporeal membrane oxygenation (ECMO) /extracorporeal life support (ECLS) provided by physician; insertion of peripheral (arterial and/or venous) cannula(e), open, birth through 5 years of age		ECMO							
46257729	Extracorporeal membrane oxygenation (ECMO) /extracorporeal life support (ECLS) provided by physician; reposition peripheral (arterial and/or venous) cannula(e), open, birth through 5 years of age (includes fluoroscopic guidance, when performed)		ECMO							
46270991	Emergency lower segment cesarean section with inverted T incision		Emergency cesarean section							
42537021	Emergency lower segment cesarean section with bilateral tubal ligation		Emergency cesarean section							
42536954	Emergency upper segment cesarean section with bilateral tubal ligation		Emergency cesarean section							
4250010	Born by emergency cesarean section		Emergency cesarean section							
4127252	Emergency lower segment cesarean section		Emergency cesarean section							
4032767	Emergency upper segment cesarean section		Emergency cesarean section							
45880554	1st trimester		First trimester							
45876697	Months 1-3 (first trimester)		First trimester							
21494044	US for pregnancy in first trimester		First trimester							
21494042	US transabdominal and transvaginal for pregnancy in first trimester		First trimester							
21493909	US transabdominal and transvaginal for multiple gestation pregnancy in first trimester		First trimester							
21493908	US for multiple gestation pregnancy in first trimester		First trimester							
4113139	First trimester		First trimester							
4034340	Prenatal state of fetus, 1st trimester		First trimester							
3050402	First trimester maternal screen with nuchal translucency panel		First trimester							
3037993	Fetal Narrative [Interpretation] Study observation. general transvaginal 1st trimester US		First trimester							
3034647	Fetal Narrative [Interpretation] Study observation. general 1st trimester, multiple fetuses US		First trimester							
3034062	Fetal Narrative [Interpretation] Study observation. general 1st trimester US		First trimester							
3030256	First trimester maternal screen with nuchal translucency [Interpretation]		First trimester							
2793348	Ultrasonography of First Trimester, Multiple Gestation		First trimester							
2793347	Ultrasonography of First Trimester, Single Fetus		First trimester							
4127705	Term pregnancy delivered		Full term			X				
3002314	Last menstrual period start date		Gestational age, other							
1175623	Delivery date Estimated from physical exam		Gestational age, other							
3024973	Delivery date Estimated from ovulation date		Gestational age, other							
3036322	Gestational age Estimated from selected delivery date		Gestational age, other							
3038318	Delivery date Estimated from conception date		Gestational age, other							
3038608	Delivery date Estimated from prior gestational age assessment		Gestational age, other							
3043737	Conception date		Gestational age, other							
4072438	Date of last menstrual period		Gestational age, other							

4089559	Estimated date of conception		Gestational age, other							
4266763	Fetal gestation at delivery - finding		Gestational age, other							
40485048	Estimated fetal gestational age at delivery		Gestational age, other							
40490322	Transvaginal ultrasonography to determine the estimated date of confinement		Gestational age, other							
40760182	Delivery date Estimated from quickening date		Gestational age, other							
40760183	Delivery date Estimated from date fundal height reaches umb		Gestational age, other							
42537958	Estimated date of delivery from antenatal ultrasound scan		Gestational age, other							
43054890	Gestational age--at birth [RHEA]		Gestational age, other							
44817092	Mother's Last menstrual period start date before delivery		Gestational age, other							
46234792	Gestational age--at birth		Gestational age, other							
45757181	Supervision of high risk pregnancy for primigravida age 15 years or younger done		Gravidity							
36684864	Normal pregnancy in primigravida		Gravidity							
45757180	Supervision of high risk pregnancy for multigravida age 15 years or younger done		Gravidity							
3037265	Ultrasonographer gravidity number		Gravidity							
36713461	Antenatal care of elderly primigravida		Gravidity							
4132100	Gravida 4		Gravidity							
4132565	Gravida more than 10		Gravidity							
45769851	Supervision of high risk pregnancy for multigravida done		Gravidity							
3192256	Antepartum elder primigravida		Gravidity							
36684865	Normal pregnancy in multigravida		Gravidity							
4132099	Gravida 3		Gravidity							
45765731	Supervision of high risk pregnancy for multigravida		Gravidity							
4182766	Gravida - finding		Gravidity							
4133030	Gravida 5		Gravidity							
4132563	Gravida 2		Gravidity							
3173485	Normal first pregnancy		Gravidity							
4133033	Gravida 9		Gravidity							
4133724	Gravida 10		Gravidity							
4204988	Gravida 0		Gravidity							
4132101	Gravida 6		Gravidity							
4132564	Gravida 7		Gravidity							
4133032	Gravida 8		Gravidity							
4049024	Previous pregnancy 1		Gravidity							
2788018	Assistance with Respiratory Ventilation, Less than 24 Consecutive Hours		IMV							
4026054	Changing endotracheal tube		IMV							
4082243	Emergency laryngeal intubation		IMV							
4168966	Endotracheal tube present		IMV							
4339623	Oral intubation awake		IMV							
4353715	Ventilator finding		IMV							
2788037	Respiratory Ventilation, 24-96 Consecutive Hours		IMV							
2788038	Respiratory Ventilation, Greater than 96 Consecutive Hours		IMV							
4056812	Laryngeal intubation for inhalation		IMV							
4108138	Ventilator rate		IMV							
4134538	Unintended endobronchial intubation		IMV							
4259233	Ventilator care assessment		IMV							
44509482	Other specified ventilation support		IMV							
765576	Orotracheal intubation using bougie device		IMV							
2788023	Assistance with Respiratory Ventilation, 24-96 Consecutive Hours		IMV							
2788028	Assistance with Respiratory Ventilation, Greater than 96 Consecutive Hours		IMV							
4174085	Dual pressure spontaneous ventilation support weaning protocol		IMV							
4180641	Complication of respiratory therapy procedure		IMV							
4219858	Problem with patient ventilator		IMV							
4229714	Mechanical ventilation weaning response		IMV							
4235361	Hyperventilation therapy for traumatic brain injury		IMV							
4251737	Ventilator care management		IMV							
4287921	Retrograde intubation		IMV							
4301549	Ventilator care and adjustment		IMV							
4335583	Tracheal intubation using rigid bronchoscope		IMV							
4337045	Blind nasal intubation		IMV							
2106469	Intubation, endotracheal, emergency procedure		IMV							
2108681	Patient receiving care in the intensive care unit (ICU) and receiving mechanical ventilation, 24 hours or less (CRIT)		IMV							
2800859	Extracorporeal or Systemic Assistance and Performance @ Physiological Systems @ Performance @ Respiratory @ 24-96 Consecutive Hours @ Ventilation		IMV							
2867784	Extracorporeal or Systemic Assistance and Performance @ Physiological Systems @ Assistance @ Respiratory @ 24-96 Consecutive Hours @ Ventilation		IMV							
2893766	Extracorporeal or Systemic Assistance and Performance @ Physiological Systems @ Performance @ Respiratory @ Less than 24 Consecutive Hours @ Ventilation		IMV							
3006292	Ventilator wave form		IMV							
4119642	Awake intubation		IMV							
4140765	Digital assisted intubation		IMV							
4179373	Insertion of endotracheal tube using laryngoscope		IMV							
4232891	Mechanical ventilation response		IMV							
4237618	Ventilator care		IMV							
4335481	Orotracheal intubation		IMV							
4335584	Nasal intubation awake		IMV							
4337616	Nasotracheal intubation		IMV							
44791135	Ventilatory support		IMV							
259992	Ventilator associated pneumonia		IMV							
2007912	Other intubation of respiratory tract		IMV							
2788036	Respiratory Ventilation, Less than 24 Consecutive Hours		IMV							
2813710	Extracorporeal or Systemic Assistance and Performance @ Physiological Systems @ Assistance @ Respiratory @ Greater than 96 Consecutive Hours @ Ventilation		IMV							

2834015	Extracorporeal or Systemic Assistance and Performance @ Physiological Systems @ Performance @ Respiratory @ Greater than 96 Consecutive Hours @ Ventilation		IMV							
4283807	Intubation of larynx		IMV							
4325601	Complication of ventilation therapy		IMV							
4335585	Endobronchial intubation		IMV							
40487536	Intubation of respiratory tract		IMV							
42738853	Ventilation assist and management, initiation of pressure or volume preset ventilators for assisted or controlled breathing; subsequent days (Deprecated)		IMV							
44808555	Provision of mechanical ventilator		IMV							
4031379	Artificial ventilation finding		IMV							
4058031	Endotracheal intubation, emergency procedure		IMV							
4072633	Weaning from mechanically assisted ventilation		IMV							
4134853	Weaning from mechanically assisted ventilation continued		IMV							
4337615	Orotracheal fiberoptic intubation		IMV							
37116698	Insertion of double lumen tracheobronchial tube		IMV							
42738852	Ventilation assist and management, initiation of pressure or volume preset ventilators for assisted or controlled breathing; first day (Deprecated)		IMV							
2314001	Ventilation assist and management, initiation of pressure or volume preset ventilators for assisted or controlled breathing; hospital inpatient/observation, each subsequent day		IMV							
2805870	Extracorporeal or Systemic Assistance and Performance @ Physiological Systems @ Assistance @ Respiratory @ Less than 24 Consecutive Hours @ Ventilation		IMV							
4013354	Insertion of endotracheal tube		IMV							
4080957	Endotracheal respiratory assistance		IMV							
4230167	Artificial respiration		IMV							
4287922	Weaning from mechanically assisted ventilation commenced		IMV							
4303945	Tracheal intubation through a laryngeal mask airway		IMV							
4308797	Trial for spontaneous breathing		IMV							
4337617	Nasotracheal fiberoptic intubation		IMV							
37116689	Insertion of endotracheal ventilation catheter		IMV							
40481547	Dependence on ventilator		IMV							
44515633	Other specified intubation of trachea		IMV							
45887795	Ventilation assist and management, initiation of pressure or volume preset ventilators for assisted or controlled breathing		IMV							
45889042	Ventilator Management		IMV							
46273390	Dependence on respirator		IMV							
2314000	Ventilation assist and management, initiation of pressure or volume preset ventilators for assisted or controlled breathing; hospital inpatient/observation, initial day		IMV							
4149878	Transglottic catheterization of trachea		IMV							
9189	Negative		Lab Negative Result							
45884092	Nonreactive		Lab Negative Result							
9190	Not detected		Lab Negative Result							
45880296	Not detected		Lab Negative Result							
45878583	Negative		Lab Negative Result							
45884153	Normal		Lab Negative Result							
36309158	Not detected/negative		Lab Negative Result							
36032716	Presumptive positive		Lab Positive Result							
45877985	Detected		Lab Positive Result							
45884084	Positive		Lab Positive Result							
9191	Positive		Lab Positive Result							
4126681	Detected		Lab Positive Result							
36715206	Presumptive positive		Lab Positive Result							
45878745	Abnormal		Lab Positive Result							
45881802	Reactive		Lab Positive Result							
3185356	Antepartum twin pregnancy with death of one fetus		Multiple pregnancy							
435357	Conjoined twins		Multiple pregnancy							
4129709	Continuing pregnancy after abortion of sibling fetus		Multiple pregnancy							
43020670	Continuing pregnancy after intrauterine death of one twin with intrauterine retention of dead twin		Multiple pregnancy							
45757769	Continuing triplet pregnancy after spontaneous abortion of one or more fetuses		Multiple pregnancy							
37017322	Dizygotic twin pregnancy		Multiple pregnancy							
46271081	Fetoscopic laser photocoagulation of placental vessel for twin to twin transfusion syndrome		Multiple pregnancy							
43021057	Monochorionic diamniotic twin pregnancy with dissimilar amniotic fluid volumes		Multiple pregnancy							
43021056	Monochorionic diamniotic twin pregnancy with similar amniotic fluid volumes		Multiple pregnancy							
43021952	Monochorionic monoamniotic twin pregnancy		Multiple pregnancy							
43021055	Monochorionic twin pregnancy		Multiple pregnancy							
37017323	Monozygotic twin pregnancy		Multiple pregnancy							
42872832	MRI of multiple pregnancy		Multiple pregnancy							
4344627	Multifetal pregnancy reduction		Multiple pregnancy							
4178154	Multiple pregnancy with one fetal loss		Multiple pregnancy							
4097427	Premature birth of fraternal twins, both living		Multiple pregnancy							
4222915	Premature birth of fraternal twins, both stillborn		Multiple pregnancy							
4147043	Premature birth of fraternal twins, one living, one stillborn		Multiple pregnancy							
4142021	Premature birth of identical twins, both living		Multiple pregnancy							
4242724	Premature birth of identical twins, both stillborn		Multiple pregnancy							
4310910	Premature birth of identical twins, one living, one stillborn		Multiple pregnancy							
4184594	Premature birth of stillborn twins		Multiple pregnancy							
4227728	Term birth of fraternal twins, both living		Multiple pregnancy							
4306288	Term birth of fraternal twins, both stillborn		Multiple pregnancy							
4330570	Term birth of fraternal twins, one living, one stillborn		Multiple pregnancy							
4049333	Term birth of identical twins, both living		Multiple pregnancy							
4192641	Term birth of identical twins, both stillborn		Multiple pregnancy							
4243026	Term birth of identical twins, one living, one stillborn		Multiple pregnancy							
4203001	Term birth of stillborn twins		Multiple pregnancy							
42535052	Twin live born in hospital by vaginal delivery		Multiple pregnancy							

4028339	Twin-to-twin blood transfer		Multiple pregnancy							
4015422	Triplets - one live and two stillborn		Multiple pregnancy							
4015163	Triplets - three stillborn		Multiple pregnancy							
4015421	Triplets - two live and one stillborn		Multiple pregnancy							
4129025	Vanishing twin syndrome		Multiple pregnancy							
44807919	Doppler ultrasonography of multiple pregnancy		Multiple pregnancy							
4226978	External fetal monitor surveillance during multiple pregnancy		Multiple pregnancy							
44807918	Ultrasonography of multiple pregnancy		Multiple pregnancy							
40492794	Ultrasonography of multiple pregnancy for fetal anomaly		Multiple pregnancy							
40489798	Ultrasonography of multiple pregnancy for fetal nuchal translucency		Multiple pregnancy							
21493908	US for multiple gestation pregnancy in first trimester		Multiple pregnancy							
21493910	US for multiple gestation pregnancy in second or third trimester		Multiple pregnancy							
21493907	US for multiple gestation pregnancy limited		Multiple pregnancy							
21493911	US for multiple gestation pregnancy with fetal abnormality		Multiple pregnancy							
21493909	US transabdominal and transvaginal for multiple gestation pregnancy in first trimester		Multiple pregnancy							
45757730	Quadruplet pregnancy with loss of one or more fetuses		Multiple pregnancy							
44783183	Combined intrauterine and ovarian pregnancy		Multiple pregnancy							
4304316	Septuplet pregnancy		Multiple pregnancy							
4216512	Quintuplet pregnancy		Multiple pregnancy							
4185557	Sextuplet pregnancy		Multiple pregnancy							
4135209	Combined pregnancy		Multiple pregnancy							
4129021	Undiagnosed multiple pregnancy		Multiple pregnancy							
4028643	Undiagnosed twin		Multiple pregnancy							
45765500	Quadruplet birth		Multiple pregnancy							
42539377	Quadruplets with all four stillborn		Multiple pregnancy							
45757166	Quadruplets, all live born		Multiple pregnancy							
45765501	Quintuplet birth		Multiple pregnancy							
42538805	Quintuplets with all five stillborn		Multiple pregnancy							
45757167	Quintuplets, all live born		Multiple pregnancy							
45765502	Sextuplet birth		Multiple pregnancy							
42538806	Sextuplets with all six stillborn		Multiple pregnancy							
45772082	Sextuplets, all live born		Multiple pregnancy							
4014298	Three female babies		Multiple pregnancy							
4014459	Two male and one female babies		Multiple pregnancy							
4077859	Number of fetuses		Number of fetuses							
443269	Elderly primiparous with labor		Parity							
4088917	Parity of cervical os		Parity							
4182453	Parity of cervical os - finding		Parity							
4012561	Nulliparous		Parity							
4132155	Para 7		Parity							
4167088	Grand multipara in labor		Parity							
4304372	Parity nine		Parity							
4044074	Parity ten or more		Parity							
4041279	Parity finding		Parity							
3016572	[#] Parity		Parity							
4275654	Parity eight		Parity							
4167662	Para 2		Parity							
4264419	Parity		Parity							
4146605	Parity, function		Parity							
4172590	Para 3		Parity							
4173934	Para 4		Parity							
4030380	Para 5		Parity							
4097847	Para 6		Parity							
4009589	Post-term delivery		Post-term							
46271814	Baby premature 32-36 weeks		Preterm pregnancy							
4097427	Premature birth of fraternal twins, both living		Preterm pregnancy							
4310910	Premature birth of identical twins, one living, one stillborn		Preterm pregnancy							
4229581	Preterm infant status: 24-37 weeks gestation		Preterm pregnancy							
4272248	Premature birth of newborn		Preterm pregnancy			X				
37079065	Spontaneous preterm delivery (<37 weeks)		Preterm pregnancy							
4029786	Premature birth of newborn twins		Preterm pregnancy							
4103876	Stillbirth of premature female (1000-2499 gms.)		Preterm pregnancy							
4107401	Premature birth of newborn triplets		Preterm pregnancy							
4145125	Premature birth of newborn quadruplets		Preterm pregnancy							
46271955	Baby premature 28-32 weeks		Preterm pregnancy							
4008884	Premature birth of newborn sextuplets		Preterm pregnancy							
4147043	Premature birth of fraternal twins, one living, one stillborn		Preterm pregnancy							
37110284	Preterm delivery following Cesarean section		Preterm pregnancy							
2721457	Home management of preterm premature rupture of membranes (pprom), including administrative services, professional pharmacy services, care coordination, and all necessary supplies or equipment (drugs and nursing visits coded separately), per diem (do n...		Preterm pregnancy							
3174212	Premature 28 week quadruplet		Preterm pregnancy							
4069200	Premature birth of multiple newborns		Preterm pregnancy							
4142021	Premature birth of identical twins, both living		Preterm pregnancy							
4149523	Very preterm maturity of infant		Preterm pregnancy							
4082863	Premature birth of newborn quintuplets		Preterm pregnancy							
4172135	Premature birth of newborn male		Preterm pregnancy							
4241228	Premature birth of newborn female		Preterm pregnancy							
4300979	Stillbirth of premature male (1000-2499 gms.)		Preterm pregnancy							
36675035	Prematurity of infant		Preterm pregnancy							
37110283	Preterm delivery following induction of labor		Preterm pregnancy							
44793462	Preterm infant status		Preterm pregnancy							
586519	SARS-CoV-2 (COVID-19) S gene [Presence] in Serum or Plasma by NAA with probe detection		SARS-CoV-2 rt-PCR and AG							
706166	SARS-related coronavirus E gene [Cycle Threshold #] in Specimen by NAA with probe detection		SARS-CoV-2 rt-PCR and AG							
706169	SARS-CoV-2 (COVID-19) RNA panel - Specimen by NAA with probe detection		SARS-CoV-2 rt-PCR and AG							
706173	SARS-CoV-2 (COVID-19) RdRp gene [Presence] in Specimen by NAA with probe detection		SARS-CoV-2 rt-PCR and AG							

723465	SARS-CoV-2 (COVID-19) S gene [Presence] in Respiratory specimen by NAA with probe detection		SARS-CoV-2 rt-PCR and AG								
723476	SARS-CoV-2 (COVID-19) RNA [Presence] in Nasopharynx by NAA with non-probe detection		SARS-CoV-2 rt-PCR and AG								
757685	SARS-CoV+SARS-CoV-2 (COVID-19) Ag [Presence] in Respiratory specimen by Rapid immunoassay		SARS-CoV-2 rt-PCR and AG								
36031506	SARS-CoV-2 (COVID-19) ORF1ab region [Presence] in Saliva (oral fluid) by NAA with probe detection		SARS-CoV-2 rt-PCR and AG								
586528	SARS-CoV-2 (COVID-19) RNA [Cycle Threshold #] in Respiratory specimen by NAA with probe detection		SARS-CoV-2 rt-PCR and AG								
706161	SARS-CoV-2 (COVID-19) N gene [Presence] in Respiratory specimen by NAA with probe detection		SARS-CoV-2 rt-PCR and AG								
706167	SARS-CoV-2 (COVID-19) N gene [Cycle Threshold #] in Specimen by NAA with probe detection		SARS-CoV-2 rt-PCR and AG								
723463	SARS-CoV-2 (COVID-19) RNA [Presence] in Serum or Plasma by NAA with probe detection		SARS-CoV-2 rt-PCR and AG								
723468	SARS-CoV-2 (COVID-19) S gene [Cycle Threshold #] in Specimen by NAA with probe detection		SARS-CoV-2 rt-PCR and AG								
723471	SARS-CoV-2 (COVID-19) RdRp gene [Cycle Threshold #] in Respiratory specimen by NAA with probe detection		SARS-CoV-2 rt-PCR and AG								
757677	SARS-CoV-2 (COVID-19) RNA [Presence] in Nose by NAA with probe detection		SARS-CoV-2 rt-PCR and AG								
36031238	SARS-CoV-2 (COVID-19) RNA [Presence] in Respiratory specimen by NAA with non-probe detection		SARS-CoV-2 rt-PCR and AG								
36031944	SARS-CoV-2 (COVID-19) specific TCRB gene rearrangements [Presence] in Blood by Sequencing		SARS-CoV-2 rt-PCR and AG								
586516	SARS-CoV-2 (COVID-19) [Presence] in Specimen by Organism specific culture		SARS-CoV-2 rt-PCR and AG								
706155	SARS-CoV-2 (COVID-19) N gene [Cycle Threshold #] in Specimen by Nucleic acid amplification using CDC primer-probe set N2		SARS-CoV-2 rt-PCR and AG								
706156	SARS-CoV-2 (COVID-19) N gene [Presence] in Specimen by Nucleic acid amplification using CDC primer-probe set N1		SARS-CoV-2 rt-PCR and AG								
706170	SARS-CoV-2 (COVID-19) RNA [Presence] in Specimen by NAA with probe detection		SARS-CoV-2 rt-PCR and AG								
706171	SARS-related coronavirus N gene [Presence] in Specimen by Nucleic acid amplification using CDC primer-probe set N3		SARS-CoV-2 rt-PCR and AG								
706172	SARS-related coronavirus N gene [Cycle Threshold #] in Specimen by Nucleic acid amplification using CDC primer-probe set N3		SARS-CoV-2 rt-PCR and AG								
723466	SARS-CoV-2 (COVID-19) S gene [Presence] in Specimen by NAA with probe detection		SARS-CoV-2 rt-PCR and AG								
36031213	SARS-CoV-2 (COVID-19) S gene [Presence] in Respiratory specimen by Sequencing		SARS-CoV-2 rt-PCR and AG								
36661370	SARS-CoV-2 (COVID-19) N gene [#/volume] (viral load) in Respiratory specimen by NAA with probe detection		SARS-CoV-2 rt-PCR and AG								
706168	SARS-CoV-2 (COVID-19) ORF1ab region [Cycle Threshold #] in Specimen by NAA with probe detection		SARS-CoV-2 rt-PCR and AG								
706174	SARS-related coronavirus E gene [Presence] in Specimen by NAA with probe detection		SARS-CoV-2 rt-PCR and AG								
706175	SARS-CoV-2 (COVID-19) N gene [Presence] in Specimen by NAA with probe detection		SARS-CoV-2 rt-PCR and AG								
715262	SARS-CoV-2 (COVID-19) RNA [Log #/volume] (viral load) in Specimen by NAA with probe detection		SARS-CoV-2 rt-PCR and AG								
723477	SARS-CoV-2 (COVID-19) Ag [Presence] in Respiratory specimen by Rapid immunoassay		SARS-CoV-2 rt-PCR and AG								
36031453	SARS-CoV-2 (COVID-19) RdRp gene [Presence] in Upper respiratory specimen by NAA with probe detection		SARS-CoV-2 rt-PCR and AG								
36032419	SARS-CoV-2 (COVID-19) Ag [Presence] in Upper respiratory specimen by Immunoassay		SARS-CoV-2 rt-PCR and AG								
36661378	SARS-CoV-2 (COVID-19) N gene [Presence] in Saliva (oral fluid) by NAA with probe detection		SARS-CoV-2 rt-PCR and AG								
37310257	Measurement of Severe acute respiratory syndrome coronavirus 2 antigen		SARS-CoV-2 rt-PCR and AG								
586520	SARS-CoV-2 (COVID-19) N gene [Presence] in Serum or Plasma by NAA with probe detection		SARS-CoV-2 rt-PCR and AG								
586523	SARS-related coronavirus E gene [Presence] in Respiratory specimen by NAA with probe detection		SARS-CoV-2 rt-PCR and AG								
586525	SARS-CoV-2 (COVID-19) N gene [Presence] in Respiratory specimen by Nucleic acid amplification using CDC primer-probe set N2		SARS-CoV-2 rt-PCR and AG								
586526	SARS-CoV-2 (COVID-19) RNA [Presence] in Nasopharynx by NAA with probe detection		SARS-CoV-2 rt-PCR and AG								
586529	SARS-CoV-2 (COVID-19) RNA [Cycle Threshold #] in Specimen by NAA with probe detection		SARS-CoV-2 rt-PCR and AG								
706157	SARS-CoV-2 (COVID-19) N gene [Cycle Threshold #] in Specimen by Nucleic acid amplification using CDC primer-probe set N1		SARS-CoV-2 rt-PCR and AG								
706159	SARS-related coronavirus+MERS coronavirus RNA [Presence] in Respiratory specimen by NAA with probe detection		SARS-CoV-2 rt-PCR and AG								
715261	SARS-CoV-2 (COVID-19) RNA [Presence] in Saliva (oral fluid) by Sequencing		SARS-CoV-2 rt-PCR and AG								
715272	SARS-CoV-2 (COVID-19) N gene [Presence] in Nasopharynx by NAA with probe detection		SARS-CoV-2 rt-PCR and AG								
723470	SARS-CoV-2 (COVID-19) RdRp gene [Cycle Threshold #] in Specimen by NAA with probe detection		SARS-CoV-2 rt-PCR and AG								
723472	SARS-related coronavirus RNA [Presence] in Specimen by NAA with probe detection		SARS-CoV-2 rt-PCR and AG								
757678	SARS-CoV-2 (COVID-19) N gene [Presence] in Nose by NAA with probe detection		SARS-CoV-2 rt-PCR and AG								
36032061	SARS-CoV-2 (COVID-19) RNA panel - Saliva (oral fluid) by NAA with probe detection		SARS-CoV-2 rt-PCR and AG								
36032174	SARS-CoV-2 (COVID-19) RdRp gene [Presence] in Saliva (oral fluid) by NAA with probe detection		SARS-CoV-2 rt-PCR and AG								
36032258	SARS-CoV-2 (COVID-19) N gene [Presence] in Saliva (oral fluid) by Nucleic acid amplification using CDC primer-probe set N1		SARS-CoV-2 rt-PCR and AG								
36661371	SARS-CoV-2 (COVID-19) N gene [Log #/volume] (viral load) in Respiratory specimen by NAA with probe detection		SARS-CoV-2 rt-PCR and AG								

706158	SARS-CoV-2 (COVID-19) RNA panel - Respiratory specimen by NAA with probe detection		SARS-CoV-2 rt-PCR and AG							
706160	SARS-CoV-2 (COVID-19) RdRp gene [Presence] in Respiratory specimen by NAA with probe detection		SARS-CoV-2 rt-PCR and AG							
706163	SARS-CoV-2 (COVID-19) RNA [Presence] in Respiratory specimen by NAA with probe detection		SARS-CoV-2 rt-PCR and AG							
706165	SARS-related coronavirus RNA [Presence] in Respiratory specimen by NAA with probe detection		SARS-CoV-2 rt-PCR and AG							
715260	SARS-CoV-2 (COVID-19) RNA [Presence] in Saliva (oral fluid) by NAA with probe detection		SARS-CoV-2 rt-PCR and AG							
723469	SARS-CoV-2 (COVID-19) ORF1ab region [Cycle Threshold #] in Respiratory specimen by NAA with probe detection		SARS-CoV-2 rt-PCR and AG							
36661377	SARS-CoV-2 (COVID-19) RNA [Presence] in Respiratory specimen by Sequencing		SARS-CoV-2 rt-PCR and AG							
586518	SARS-related coronavirus E gene [Presence] in Serum or Plasma by NAA with probe detection		SARS-CoV-2 rt-PCR and AG							
586524	SARS-CoV-2 (COVID-19) N gene [Presence] in Respiratory specimen by Nucleic acid amplification using CDC primer-probe set N1		SARS-CoV-2 rt-PCR and AG							
706154	SARS-CoV-2 (COVID-19) N gene [Presence] in Specimen by Nucleic acid amplification using CDC primer-probe set N2		SARS-CoV-2 rt-PCR and AG							
723464	SARS-CoV-2 (COVID-19) ORF1ab region [Presence] in Specimen by NAA with probe detection		SARS-CoV-2 rt-PCR and AG							
723467	SARS-CoV-2 (COVID-19) S gene [Cycle Threshold #] in Respiratory specimen by NAA with probe detection		SARS-CoV-2 rt-PCR and AG							
723478	SARS-CoV-2 (COVID-19) ORF1ab region [Presence] in Respiratory specimen by NAA with probe detection		SARS-CoV-2 rt-PCR and AG							
36031652	SARS-CoV-2 (COVID-19) RdRp gene [Presence] in Lower respiratory specimen by NAA with probe detection		SARS-CoV-2 rt-PCR and AG							
45879078	2nd trimester		Second trimester							
45876698	Months 4-6 (second trimester)		Second trimester							
40486918	Ultrasonography in second trimester		Second trimester							
40483600	Measurement of alpha fetoprotein in second trimester		Second trimester							
4240362	Prenatal state of fetus, 2nd trimester		Second trimester							
4113140	Second trimester		Second trimester							
3053322	Second trimester quad maternal screen panel - Serum or Plasma		Second trimester							
3049557	Second trimester penta maternal screen [Interpretation] in Serum or Plasma		Second trimester							
3049518	Second trimester penta maternal screen panel - Serum or Plasma		Second trimester							
3037974	Fetal Narrative [Interpretation] Study observation. general 2nd trimester US		Second trimester							
3031374	Fetal Narrative [Interpretation] Study observation. general 2nd trimester, multiple fetuses US		Second trimester							
2793350	Ultrasonography of Second Trimester, Multiple Gestation		Second trimester							
2793349	Ultrasonography of Second Trimester, Single Fetus		Second trimester							
4034087	Single pregnancy		Singleton pregnancy							
40483521	Single liveborn born in hospital by cesarean section		Singleton pregnancy			X				
40483126	Liveborn born in hospital by cesarean section		Singleton pregnancy			X				
2793349	Ultrasonography of Second Trimester, Single Fetus		Singleton pregnancy							
45885074	3rd trimester		Third trimester							
45882929	Months 7-9 (third trimester)		Third trimester							
40487413	Ultrasonography in third trimester		Third trimester							
36713272	Three dimensional obstetric ultrasonography in third trimester		Third trimester							
4180111	Third trimester pregnancy less than 36 weeks		Third trimester							
4112238	Third trimester		Third trimester							
4029320	Prenatal state of fetus, 3rd trimester		Third trimester							
3032525	Fetal Narrative [Interpretation] Study observation. general 3rd trimester US		Third trimester							
3032291	Fetal Narrative [Interpretation] Study observation. general 3rd trimester, multiple fetuses US		Third trimester							
2793352	Ultrasonography of Third Trimester, Multiple Gestation		Third trimester							

Table S2. Definitions of variables related to pregnancy, demographics, and COVID.		
**Variable**	**Data Type**	**Definition**
	**Demographics**	
person_id	string	Unique identifier for patient in N3C.
data_partner_id	integer	Anonymized institution contributing to patient's record in N3C.
year_of_birth	integer	Patient's year of birth.
month_of_birth	integer	Patient's month of birth.
date_of_birth	date	Birth date of patient using year_of_birth, month_of_birth, and the value of 1 as day of birth.
race_ethnicity	string	"Hispanic or Latino" for anyone with this ethnicity, otherwise "White Non-Hispanic", "Asian Non-Hispanic", "Black or African American Non-Hispanic", or other "Other Non-Hispanic". "Unknown" is assigned to all others.
location_id	string	Unique location identifier for obtaining patient's location (city, state, and zip code).
	**Pregnancy-related**	
episode_number	integer	Indicates the nth pregnancy episode that appears in a pregnant person's record.
HIP_flag	integer	Value of 1 indicates if pregnancy episode was identifed by HIP.
PPS_flag	integer	Value of 1 indicates if pregnancy episode was identifed by PPS.
HIP_outcome_category	string	Pregnancy outcome category inferred by HIP. Pregnancy outcome categories are "live birth", "stillbirth", "spontaneous abortion", "ectopic pregnacy", "delivery record only", and "missing outcome".
HIP_end_date	date	Pregnancy episode end date inferred by HIP.
PPS_outcome_category	string	Pregnancy outcome category inferred by PPS. Pregnancy outcome categories are "live birth", "stillbirth", "spontaneous abortion", "ectopic pregnacy", "delivery record only", and "missing outcome".
PPS_end_date	date	Pregnancy episode end date inferred by PPS.
recorded_episode_start	date	First date of the recorded pregnancy episode.
recorded_episode_end	date	Last date of the recorded pregnancy episode.
recorded_episode_length	integer	Episode length in days by taking the difference between recorded_episode_end and recorded_episode_start.
GW_flag	integer	Value of 1 indicates if a GW concept occurs during a pregnancy episode.
GR3m_flag	integer	Value of 1 indicates if a GR3m concept occurs during a pregnancy episode.
inferred_episode_start	date	Inferred pregnancy episode start date using ESD algorithm.
precision_days	integer	Precision in days of inferred pregnancy episode start date using ESD algorithm.
precision_category	string	Precision category using precision_days. The precision categories are "week", "week_poor-support", "two-week", "three-week", "month", "two-month", "three-month", and "non-specific".
outcome_match	integer	Value of 1 if PPS_outcome_category and HIP_outcome_category match AND PPS_end_date and HIP_end_date are within 14 days of each other for pregnancy outcomes only. For "Missing outcome", a value of 1 is given if PPS_outcome_category and HIP_outcome_category match.
final_outcome_category	string	If PPS_outcome_category and HIP_outcome_category do not match, take the outcome category that occurs later as the final outcome category. If PPS_outcome_category is "Missing outcome" and HIP_outcome_category is not "Missing outcome", then take the HIP_outcome_category as the final outcome category. Otherwise, take the matching outcome category as the final outcome category.
inferred_episode_end	date	The inferred pregnancy episode end date corresponding with the final_outcome_category.

gestational_age_days_calculated	integer	Gestational age in days taking the difference between inferred_episode_end and inferred_episode_start.
term_duration_flag	integer	Value of 1 if the final_outcome_category occurred during the term duration expected of that pregnancy outcome using gestational_age_days_calculated. For "Missing outcome", a value of 1 was assigned if the gestational_age_days_calculated was <= 301 days.
outcome_concordance score	integer	A score of 2 has outcome_match of 1 AND term_duration_flag of 1 AND GW_flag of 1. A score of 1 has term_duration_flag of 1 AND GW_flag of 1. A score of 0 does not meet the criteria of either score 1 or 2.
num_fetuses	integer	Number of fetuses obtained from values associated with "Number of fetuses" concepts.
singleton_flag	integer	Value of 1 if pregnancy episode contains a "Singleton pregnancy" concept.
multiple_flag	integer	Value of 1 if pregnancy episode contains a "Multiple pregnancy" concept.
preterm_concept_flag	integer	Value of 1 if pregnancy episodes contains a "Preterm pregnancy" concept within 7 days before or 14 days after inferred_episode_end.
preterm_status_from_calculation	integer	Value of 1 if gestational_age_days_calculated < 259 days.
preterm_flag	integer	Value of 1 if preterm_status_from_calculation is 1 OR preterm_concept_flag is 1.
final_preterm_flag	integer	Value of 1 if preterm_flag is 1 and final_outcome_category is "live birth".
cesarean_flag	integer	Value of 1 if pregnancy episode contains a "Cesarean section" concept within 7 days before or 14 days after inferred_episode_end.
age_at_inferred_start	double	Age at inferred start of pregnancy episode by taking the difference between inferred_episode_start and date_of_birth.
age_groups	string	Age group categories using age_at_inferred_start: "15-17", "18-25", "26-35" , and "35+".
**COVID-related**		
had_screening	integer	Value of 1 if an instance of the measurement "SARS-CoV-2 rt-PCR and AG" was done during a pregnancy episode and up to 7 days after inferred_episode_end.
index_infection	integer	Value of 1 if a positive PCR or AG test is the first occurrence in a patient's records.
reinfection	integer	Value of 1 if a positive PCR or AG test occurred > 60 days after the date of the previous PCR or AG test (either positive or negative result).
had_covid	integer	Value of 1 if index_infection is 1 OR reinfection is 1 during a pregnancy episode. The visit date associated with either infection type must occur during a pregnancy episode and up to 7 days after inferred_episode_end.
COVID_first_poslab_or_diagnosis_date	date	Date representing the first instance of either the measurement "SARS-CoV-2 rt-PCR and AG" with a positive result "Lab Positive Result" or a COVID-19 diagnosis ("COVID-19 diagnosis (U07.1)" concept) having been charted (when available). This date serves as the index date.

Table S3. Concepts found empirically to be gestational timing specific (mean of concept month +/- 1.5 std dev) along with established and recommended month(s) during pregnancy for concept occurrence.								
**Concept Name**	**Concept ID**	**Minimum month concept occurs per clinician**	**Maximum month concept occurs per clinician**	**Timespan between minimum and maximum month (months)**	**Midpoint of timespan (months)**	**Used for PPS and ESD**	**Used for Validation**
Abnormal chromosomal and genetic finding on antenatal screening of mother	4064724						
Alpha-1-Fetoprotein [Mass/volume] in Serum or Plasma	3009306	3.75	5.5	1.75	4.625	X	
Alpha-1-Fetoprotein [Multiple of the median] adjusted in Serum or Plasma	3016670	3.75	5.5	1.75	4.625	X	
Alpha-1-Fetoprotein [Multiple of the median] in Serum or Plasma	3024370	3.75	5.5	1.75	4.625	X	
Alpha-fetoprotein (AFP); serum	2212198	3.75	5.5	1.75	4.625	X	
Amenorrhea	443800						
Amniocentesis; diagnostic	2110279	4	7	3	5.5	X	
Anemia during pregnancy - baby not yet delivered	432967						
Antibody; rubella	2212909	2	7	5	4.5		
Antibody; varicella-zoster	2212918	2	6	4	4		
Breech presentation	74698	4.5	11	6.5	7.75		X
CFTR (cystic fibrosis transmembrane conductance regulator) (eg, cystic fibrosis) gene analysis; common variants (eg, ACMG/ACOG guidelines)	42742292	2	6	4	4		
Choriogonadotropin [Units/volume] in Serum or Plasma	3018171						
Choriogonadotropin.beta subunit [Units/volume] in Serum or Plasma	3038136						
Choriogonadotropin.intact+Beta subunit [Units/volume] in Serum or Plasma	3046071						
Complication occurring during labor and delivery	440795	5	11	6	8		X
Diabetic on diet only	4016041						
Doppler echocardiography, fetal, pulsed wave and/or continuous wave with spectral display; complete	2211763	4	7	3	5.5	X	
Doppler velocimetry, fetal; umbilical artery	2211760	6	10	4	8		X
Early stage of pregnancy	4193062						
Echocardiography, fetal, cardiovascular system, real time with image documentation (2D), with or without M-mode recording	2722250	4	7	3	5.5	X	
Estriol	2212323	3.75	5.5	1.75	4.625	X	
Estriol (E3).unconjugated [Mass/volume] in Serum or Plasma	3025455	3.75	5.5	1.75	4.625	X	
Excessive fetal growth affecting management of mother	81636	7	11	4	9		X
False labor	4062557	5	11	6	8		X
False labor at or after 37 completed weeks of gestation	4062558	9	11	2	10	X	
False labor before 37 completed weeks of gestation	45757113	5	9.25	4.25	7.125		X
Fetal biophysical profile; with non-stress testing	2211758	6	10	4	8		X
Fetal biophysical profile; without non-stress testing	2211759	6	11	5	8.5		X
Fetal non-stress test	2110284	6	11	5	8.5		X
Fetal problem	4126571						
First trimester pregnancy	4239938	1	4	3	2.5	X	
Gestation less than 24 weeks	438542	1	6	5	3.5		X
Gestation less than 9 weeks	4322726	1	3	2	2	X	
Gestation period, 10 weeks	4242241	2.5	2.5	0	2.5	X	
Gestation period, 11 weeks	4174506	2.75	2.75	0	2.75	X	
Gestation period, 12 weeks	4197245	3	3	0	3	X	
Gestation period, 13 weeks	4266517	3.25	3.25	0	3.25	X	
Gestation period, 14 weeks	4248725	3.5	3.5	0	3.5	X	
Gestation period, 15 weeks	4283690	3.75	3.75	0	3.75	X	
Gestation period, 16 weeks	4049621	4	4	0	4	X	

Gestation period, 17 weeks	4277749	4.25	4.25	0	4.25	X	
Gestation period, 18 weeks	4097608	4.5	4.5	0	4.5	X	
Gestation period, 19 weeks	4181751	4.75	4.75	0	4.75	X	
Gestation period, 20 weeks	4051642	5	5	0	5	X	
Gestation period, 21 weeks	4185780	5.25	5.25	0	5.25	X	
Gestation period, 22 weeks	4274955	5.5	5.5	0	5.5	X	
Gestation period, 23 weeks	4336226	5.75	5.75	0	5.75	X	
Gestation period, 25 weeks	435640	6.25	6.25	0	6.25	X	
Gestation period, 26 weeks	444023	6.5	6.5	0	6.5	X	
Gestation period, 27 weeks	432430	6.75	6.75	0	6.75	X	
Gestation period, 28 weeks	444461	7	7	0	7	X	
Gestation period, 29 weeks	444417	7.25	7.25	0	7.25	X	
Gestation period, 30 weeks	434484	7.5	7.5	0	7.5	X	
Gestation period, 31 weeks	433864	7.75	7.75	0	7.75	X	
Gestation period, 32 weeks	442558	8	8	0	8	X	
Gestation period, 33 weeks	441678	8.25	8.25	0	8.25	X	
Gestation period, 34 weeks	443874	8.5	8.5	0	8.5	X	
Gestation period, 35 weeks	444267	8.75	8.75	0	8.75	X	
Gestation period, 36 weeks	438543	9	9	0	9	X	
Gestation period, 37 weeks	442355	9.25	9.25	0	9.25	X	
Gestation period, 38 weeks	443871	9.5	9.5	0	9.5	X	
Gestation period, 39 weeks	435655	9.75	9.75	0	9.75	X	
Gestation period, 40 weeks	444098	10	10	0	10	X	
Gestation period, 8 weeks	4132434	2	2	0	2	X	
Gestational age	3012266						
Gestational age method	3007259						
Gestational diabetes mellitus	4024659	6	10	4	8		X
Glucose [Mass/volume] in Serum or Plasma --1 hour post 100 g glucose PO	3014716	6	8	2	7	X	
Glucose [Mass/volume] in Serum or Plasma --1 hour post 50 g glucose PO	3016699	6	8	2	7	X	
Glucose [Mass/volume] in Serum or Plasma --1 hour post dose glucose	3010300	6	9	3	7.5	X	
Glucose [Mass/volume] in Serum or Plasma --2 hours post 100 g glucose PO	3006717	6	8	2	7	X	
Glucose [Mass/volume] in Serum or Plasma --2 hours post dose glucose	3026300						
Glucose [Mass/volume] in Serum or Plasma --3 hours post 100 g glucose PO	3027457	6	8	2	7	X	
Glucose [Mass/volume] in Serum or Plasma --3 hours post dose glucose	3027198	6	8	2	7	X	
Glucose; post glucose dose (includes glucose)	2212361	6	8	2	7	X	
Glucose; tolerance test (GTT), 3 specimens (includes glucose)	2212362	6	8	2	7	X	
Glucose; tolerance test, each additional beyond 3 specimens (List separately in addition to code for primary procedure)	2212363	6	8	2	7	X	
Gonadotropin, chorionic (hCG); quantitative	2212631						
Group B streptococcus carrier complicating pregnancy	37016152	8	11	3	9.5	X	
Hemoglobin A/Hemoglobin.total in Blood by Electrophoresis	3009131	2	10	8	6		
Hemoglobin A2/Hemoglobin.total in Blood	3020784	2	11	9	6.5		
Hemoglobin A2/Hemoglobin.total in Blood by Electrophoresis	3021009	2	10	8	6		
Hemoglobin F/Hemoglobin.total in Blood	3018738	2	10	8	6		
Hemoglobin fractionation and quantitation; chromatography (eg, A2, S, C, and/or F)	2212388	2	10	8	6		
Hemoglobin fractionation and quantitation; electrophoresis (eg, A2, S, C, and/or F)	2212387	2	10	8	6		
Hemoglobin pattern [Interpretation] in Blood by Electrophoresis Narrative	3014482	2	10	8	6		
Hemoglobin S/Hemoglobin.total in Blood	3005081	2	10	8	6		
Hemorrhagic complication of pregnancy	4025198						
High risk pregnancy due to history of preterm labor	40480278						
Infectious agent detection by nucleic acid (DNA or RNA); Streptococcus, group B, amplified probe technique	2213172	8	11	3	9.5	X	
Inhibin [Mass/volume] in Serum or Plasma	3012620	3.75	5.5	1.75	4.625	X	

Inhibin A	2212802	3.75	5.5	1.75	4.625	X	
Inhibin A [Multiple of the median] in Serum or Plasma	3035828	3.75	5.5	1.75	4.625	X	
Initial prenatal care visit (report at first prenatal encounter with health care professional providing obstetrical care. Report also date of visit and, in a separate field, the date of the last menstrual period [LMP]) (Prenatal)	2101829	1	11	10	6		X
Insulin dependent diabetes mellitus [Presence]	3046418						
Low lying placenta	40482406	4.5	11	6.5	7.75		X
Maternal hypertension	4118910						
Medical genetics and genetic counseling services, each 30 minutes face-to-face with patient/family	2314183	2	7	5	4.5		X
Monitoring of Products of Conception, Cardiac Rate, External Approach	2787788						
Multiple pregnancy	3045823						
Neural tube defect risk [Likelihood] in Fetus	3048541	3.75	5.5	1.75	4.625	X	
Number of fetuses by US	3002549						
Polyhydramnios	437623	6	11	5	8.5		X
Poor fetal growth affecting management	72693	6	11	5	8.5		X
Post-term pregnancy	432695	9	11	2	10	X	
Pre-existing hypertension complicating pregnancy, childbirth and puerperium	321074						
Pre-existing hypertension in obstetric context	4311246						
Pregnancy test positive	4094910	1	4	3	2.5	X	
Pregnancy-induced hypertension	4167493	5	11	6	8		X
Prenatal flow sheet documented in medical record by first prenatal visit (documentation includes at minimum blood pressure, weight, urine protein, uterine size, fetal heart tones, and estimated date of delivery). Report also: date of visit and, in a separate field, the date of the last menstrual period [LMP] (Note: If reporting 0501F Prenatal flow sheet, it is not necessary to report 0500F Initial prenatal care visit) (Prenatal)	2101830	1	11	10	6		X
Preterm labor without delivery	36712703	5	11	6	8		X
Progesterone	2212538	1	4	3	2.5	X	
Progesterone [Mass/volume] in Serum or Plasma	3027144	1	4	3	2.5	X	
Reduced fetal movement	77619	5.5	11	5.5	8.25		X
Requires diphtheria, tetanus and pertussis vaccination	45766222	6.75	11	4.25	8.875		X
Rubella virus IgG Ab [Presence] in Serum	3013750	2	6	4	4		
Rubella virus IgG Ab [Presence] in Serum or Plasma by Immunoassay	3043953	2	6	4	4		
Rubella virus IgG Ab [Units/volume] in Serum	3016999	2	6	4	4		
Rubella virus IgG Ab [Units/volume] in Serum or Plasma by Immunoassay	3011564	2	6	4	4		
Second trimester pregnancy	4244438	3.5	6.25	2.75	4.875	X	
Second trimester quad maternal screen [Interpretation] in Serum or Plasma Narrative	3049229	3.75	5.5	1.75	4.625	X	
Streptococcus agalactiae [Presence] in Specimen by Organism specific culture	3036000	8	11	3	9.5	X	
Streptococcus agalactiae [Presence] in Vag+Rectum by Organism specific culture	43055441	8	11	3	9.5	X	
Streptococcus agalactiae DNA [Presence] in Specimen by NAA with probe detection	3048882	8	11	3	9.5	X	
Subsequent prenatal care visit (Prenatal) [Excludes: patients who are seen for a condition unrelated to pregnancy or prenatal care (eg, an upper respiratory infection; patients seen for consultation only, not for continuing care)]	2101831						
Supervision of pregnancy with history of insufficient antenatal care	36717636						
Suspected chromosome abnormality	4246974						
Suspected fetal abnormality affecting management of mother	36712695	3	11	8	7		X
Suspected fetal disorder	43530881						
Suspected neurological disease	4253306						
Third trimester pregnancy	4218813	7	11	4	9		X

Trisomy 18 risk [Likelihood] in Fetus	3047352	2.75	5.5	2.75	4.125	X	
Trisomy 21 risk [Likelihood] in Fetus	3043238	2.75	5.5	2.75	4.125	X	
Ultrasonic guidance for amniocentesis, imaging supervision and interpretation	2211785	4	7	3	5.5	X	
Ultrasound, pregnant uterus, real time with image documentation, fetal and maternal evaluation plus detailed fetal anatomic examination, transabdominal approach; single or first gestation	2211751	4	6	2	5	X	
Ultrasound, pregnant uterus, real time with image documentation, fetal and maternal evaluation, after first trimester (> or = 14 weeks 0 days), transabdominal approach; single or first gestation	2211749	4	11	7	7.5		X
Ultrasound, pregnant uterus, real time with image documentation, fetal and maternal evaluation, first trimester (< 14 weeks 0 days), transabdominal approach; single or first gestation	2211747	2	4	2	3	X	
Ultrasound, pregnant uterus, real time with image documentation, first trimester fetal nuchal translucency measurement, transabdominal or transvaginal approach; single or first gestation	2211753	2.75	3.5	0.75	3.125	X	
Ultrasound, pregnant uterus, real time with image documentation, transvaginal	2211757	1	6	5	3.5		X
Uncertain viability of pregnancy	4084768	1	3	2	2	X	
Varicella zoster virus IgG Ab [Presence] in Serum	3022386	2	6	4	4		
Varicella zoster virus IgG Ab [Presence] in Serum by Immunoassay	3009678	2	6	4	4		
Varicella zoster virus IgG Ab [Units/volume] in Serum by Immunoassay	3023895	2	6	4	4		
Viral disease in mother complicating pregnancy, childbirth AND/OR puerperium	435604						

Note: Column "Used for PPS and ESD" denotes the final concepts used for both PPS and ESD. Column "Used for Validation" denotes the 25 concepts external to HIPPS used for validation of HIPPS to detect pregnancy episodes within N3C and infer the start and end of pregnancy episodes.

Table S4. Number of episodes by precision category for baseline vs. Estimated Start Date (ESD) Algorithm.

Precision Categories	Baseline Number of Episodes	ESD Number of Episodes		
week	305,761	475,433		
week_poor-support	115,857	90,743		
two-week	85,248	79,251		
three-week	47,692	23,131		
month	31,779	10,979		
two-month	58,129	19,124		
three-month	22,219	20,569		
non-specific	149,786	97,241		

Table S5. Number of episodes by precision category and outcome category for baseline vs. Estimated Start Date (ESD) Algorithm.

Precision Categories	Outcome Category	Baseline Number of Episodes	ESD Number of Episodes
week	Delivery record only	17,772	30,802
week_poor-support	Delivery record only	8,014	4,704
two-week	Delivery record only	4,726	5,172
three-week	Delivery record only	2,792	1,576
month	Delivery record only	2,221	1,153
two-month	Delivery record only	4,976	1,644
three-month	Delivery record only	2,103	7,179
non-specific	Delivery record only	39,597	29,971
week	Ectopic pregnancy	1,517	2,500
week_poor-support	Ectopic pregnancy	2,145	1,242
two-week	Ectopic pregnancy	354	340
three-week	Ectopic pregnancy	169	156
month	Ectopic pregnancy	65	68
two-month	Ectopic pregnancy	52	8,101
three-month	Ectopic pregnancy	21	1,627
non-specific	Ectopic pregnancy	11,691	1,980
week	Live birth	198,104	307,059
week_poor-support	Live birth	35,432	33,501
two-week	Live birth	60,418	54,074
three-week	Live birth	32,308	12,471
month	Live birth	20,942	4,730
two-month	Live birth	39,361	4,356
three-month	Live birth	14,196	2,375
non-specific	Live birth	26,091	8,286
week	Missing outcome	74,943	114,444
week_poor-support	Missing outcome	56,605	42,839
two-week	Missing outcome	15,941	15,814
three-week	Missing outcome	9,923	6,762
month	Missing outcome	6,990	3,646
two-month	Missing outcome	11,811	3,785
three-month	Missing outcome	5,454	1,072
non-specific	Missing outcome	8,686	1,991
week	Abortion	12,006	18,471
week_poor-support	Abortion	13,206	8,130
two-week	Abortion	3,427	3,464
three-week	Abortion	2,272	2,048
month	Abortion	1,419	1,318
two-month	Abortion	1,662	1,180
three-month	Abortion	321	8,219
non-specific	Abortion	63,193	54,676
week	Stillbirth	1,419	2,157
week_poor-support	Stillbirth	455	327
two-week	Stillbirth	382	387
three-week	Stillbirth	228	118
month	Stillbirth	142	64
two-month	Stillbirth	267	58
three-month	Stillbirth	124	97
non-specific	Stillbirth	528	337

Table S6. Percentage of occurrences overlapping with HIPPS-identified episodes using 25 pregnancy-specific concepts not used in HIPPS.

**Concept Name**	**Number of Occurrences**	**Number of Occurrences Overlapping with Episodes**	**Percent Overlap Occurrences**
Third trimester pregnancy	1,154,879	906,187	78.5
Fetal non-stress test	329,304	305,771	92.9
Gestational diabetes mellitus	221,672	179,606	81.0
Ultrasound, pregnant uterus, real time with image documentation, transvaginal	213,852	184,152	86.1
Complication occurring during labor and delivery	173,246	167,585	96.7
Suspected fetal abnormality affecting management of mother	167,156	148,040	88.6
Fetal biophysical profile; without non-stress testing	152,107	147,867	97.2
Ultrasound, pregnant uterus, real time with image documentation, fetal and maternal evaluation, after first trimester (> or = 14 weeks 0 days), transabdominal approach; single or first gestation	123,706	110,507	89.3
Poor fetal growth affecting management	105,903	91,232	86.1
Pregnancy-induced hypertension	76,607	71,298	93.1
Doppler velocimetry, fetal; umbilical artery	58,934	47,152	80.0
Breech presentation	58,725	55,896	95.2
Gestation less than 24 weeks	57,443	52,522	91.4
Initial prenatal care visit (report at first prenatal encounter with health care professional provid…	52,559	51,583	98.1
Reduced fetal movement	45,563	43,218	94.9
Polyhydramnios	35,622	32,106	90.1
Low lying placenta	32,763	30,842	94.1
Excessive fetal growth affecting management of mother	31,372	26,063	83.1
False labor before 37 completed weeks of gestation	29,105	27,214	93.5
Fetal biophysical profile; with non-stress testing	27,204	24,447	89.9
Requires diphtheria, tetanus and pertussis vaccination	22,240	19,554	87.9
Medical genetics and genetic counseling services, each 30 minutes face-to-face with patient/family	21,786	18,933	86.9
Preterm labor without delivery	20,493	19,371	94.5
Prenatal flow sheet documented in medical record by first prenatal visit (documentation includes at minimum blood pressure, weight, urine protein, uterine size, fetal heart tones, and estimated date of delivery). Report also: date of visit and, in a separate field, the date of the last menstrual period [LMP] (Note: If reporting 0501F Prenatal flow sheet, it is not necessary to report 0500F Initial prenatal care visit) (Prenatal)	16,322	15,240	93.4
False labor	15,799	14,694	93.0

Note: We used 25 concepts that were validated to be pregnancy-specific by clinicians and were not used for PPS or ESD to evaluate HIPPS' ability to detect all possible pregnancy episodes in N3C.

Table S7. Demographics and outcomes of pregnant persons before and during COVID-19 pandemic stratified by different cohort inclusion criteria.								
	All Episodes		Pre-March 2020		Post-March 2020		Post-March 2020	
**Panel A. All Episodes (Cohort 1)**								
	N	%	N	%	N	%	N	%
**Age**								
15-17	12,587	1.5	4,894	1.7	6,627	1.4	1,066	1.7
18-25	175,960	21.6	63,331	22.6	97,224	20.5	15,405	24.6
26-35	459,405	56.3	156,408	55.9	267,830	56.5	35,167	56.2
35+	168,519	20.6	55,270	19.7	102,347	21.6	10,902	17.4
**Race/Ethnicity**								
Asian American Non-Hispanic	35,173	4.3	10,673	3.8	22,500	4.7	2,000	3.2
Black/African American Non-Hispanic	155,976	19.1	55,384	19.8	88,903	18.8	11,689	18.7
Hispanic or Latino Any Race	144,149	17.7	44,512	15.9	86,292	18.2	13,345	21.3
Native Hawaiian or Other Pacific Islander Non-Hispanic	1,458	0.2	444	0.2	882	0.2	132	0.2
Other Non-Hispanic	9,365	1.1	2,863	1.0	5,582	1.2	920	1.5
Unknown	50,408	6.2	15,052	5.4	31,393	6.6	3,963	6.3
White Non-Hispanic	419,942	51.4	150,975	53.9	238,476	50.3	30,491	48.8
**Outcome Type**								
Live birth	426,852	52.3	135,049	48.2	260,292	54.9	31,511	50.4
Stillbirth	3,545	0.4	1,242	0.4	2,032	0.4	271	0.4
Abortion	97,506	11.9	44,715	16.0	49,367	10.4	3,424	5.5
Ectopic pregnancy	16,014	2.0	6,485	2.3	8,876	1.9	653	1
Delivery record only	82,201	10.1	29,908	10.7	46,409	9.8	5,884	9.4
Missing outcome	190,353	23.3	62,504	22.3	107,052	22.6	20,797	33.3
**COVID-19 Screening Status**								
No COVID screening	465,463	57	N/A	N/A	173,871	36.7	11,690	18.7
COVID screening	351,008	43	N/A	N/A	300,157	63.3	50,850	81.3
**Preterm Status**								
Yes	61,516	7.5	18,270	6.5	38,199	8.1	5,047	8.1
**Total**	816,471		279,903		474,028		62,540	
**Panel B. Month-Level Resolution for Pregnancy Start Date of Outcomes Only (Cohort 2)**								
	N	%	N	%	N	%	N	%
**Age**								
15-17	7,626	1.5	2,738	1.7	4,229	1.4	659	1.9
18-25	105,915	21.5	36,122	22.7	61,125	20.5	8,668	24.7
26-35	285,506	58.0	93,058	58.4	172,515	57.9	19,933	56.7
35+	93,244	18.9	27,353	17.2	60,014	20.1	5,877	16.7
**Race/Ethnicity**								
Asian American Non-Hispanic	22,902	4.7	6,329	4.0	15,358	5.2	1,215	3.5
Black/African American Non-Hispanic	87,550	17.8	28,654	18.0	52,439	17.6	6,457	18.4
Hispanic or Latino Any Race	85,930	17.5	23,907	15.0	54,097	18.2	7,926	22.6
Native Hawaiian or Other Pacific Islander Non-Hispanic	905	0.2	258	0.2	573	0.2	74	0.2
Other Non-Hispanic	5,343	1.1	15,29	1.0	3,370	1.1	444	1.3
Unknown	29,047	5.9	7,483	4.7	19,325	6.5	2,239	6.4
White Non-Hispanic	260,614	52.9	91,111	57.2	152,721	51.3	16,782	47.8
**Outcome Type**								
Live birth	411,319	83.6	129,414	81.3	251,552	84.4	30,353	86.4
Stillbirth	2,987	0.6	1,006	0.6	1,747	0.6	234	0.7
Abortion	32,667	6.6	14,498	9.1	17,149	5.8	1,020	2.9
Ectopic pregnancy	4,089	0.8	1,466	0.9	2,464	0.8	159	0.5
Delivery record only	41,229	8.4	12,887	8.1	24,971	8.4	3,371	9.6
**COVID-19 Screening Status**								
No COVID screening	228,921	46.5	N/A	N/A	65,019	21.8	4,632	13.2
COVID screening	263,370	53.5	N/A	N/A	232,864	78.2	30,505	86.8
**Preterm Status**								
Yes	58,280	11.8	17,275	10.8	36,233	12.2	4,772	13.6
**Total**	492,291		159,271		297,883		35,137	
**Panel C. Week-Level Resolution for Pregnancy Start Date of Outcomes Only with Known Status (Cohort 3)**								
	N	%	N	%	N	%	N	%
**Age**								
15-17	5,012	1.6	1,743	1.8	2,851	1.5	418	1.9
18-25	68,288	22.1	22,774	23	40,021	21.2	5,493	25
26-35	181,059	58.5	58,607	59.3	109,918	58.3	12,534	57,1
35+	54,973	17.8	15,789	16	35,695	18.9	3,489	15.9
**Race/Ethnicity**								

Asian American Non-Hispanic	13,877	4.5	3,775	3.8	9,330	4.9	772	3.5
Black/African American Non-Hispanic	54,468	17.6	17,286	17.5	33,172	17.6	4,010	18.3
Hispanic or Latino Any Race	52,271	16.9	14,523	14.7	32,996	17.5	4,752	21.7
Native Hawaiian or Other Pacific Islander Non-Hispanic	592	0.2	166	0.2	377	0.2	49	0.2
Other Non-Hispanic	3,303	1.1	881	0.9	2,158	1.1	264	1.2
Unknown	18,132	5.9	4,625	4.7	12,110	6.4	1,397	6.4
White Non-Hispanic	166,689	53.9	57,657	58.3	98,342	52.2	10,690	48.7
**Outcome Type**								
Live birth	290,813	94	91,438	92.4	178,200	94.5	21,175	96.5
Stillbirth	1,917	0.6	646	0.7	1,141	0.6	130	0.6
Abortion	14,631	4.7	6,149	6.2	7,948	4.2	534	2.4
Ectopic pregnancy	1,971	0.6	680	0.7	1,196	0.6	95	0.4
**COVID-19 Screening Status**								
No COVID screening	135,258	43.7	N/A	N/A	33,553	17.8	2,793	12,7
COVID screening	174,074	56.3	N/A	N/A	154,932	82.2	19,141	87.3
**Preterm Status**								
Yes	37,686	12.2	11,178	11.3	23,503	12.5	3,005	13.7
**Total**	309,332		98,913		188,485		21,934	

Table S8. Comparison of outcomes by HIP and PPS.

A) Outcome match (same outcome and dates within 14 days) by outcome category

Outcome Category	Number of episodes with concordant outcomes	Number of episodes with discordant outcomes	Total number of episodes	Percent of episodes with discordant outcomes
Ectopic pregnancy	6,367	9,647	16,014	60.2
Abortion	44,134	53,372	97,506	54.7
Delivery record only	55,194	27,007	82,201	32.9
Stillbirth	2,947	598	3,545	16.9
Missing outcome	174,033	16,320	190,353	8.6
Live birth	397,721	29,131	426,852	6.8
Total	680,396	136,075	816,471	

B) Algorithm used for discordant outcomes (N=119,755)

Outcome Category	Number of episodes using HIP outcome	Number of episodes using PPS outcomes	Total number of episodes with discordant outcomes
Abortion	51,613	1,759	53,372
Live birth	24,491	4,640	29,131
Delivery record only	23,256	3,751	27,007
Ectopic pregnancy	9,317	330	9,647
Stillbirth	564	34	598
Total	109,241	10,514	119,755

Table S9. Comparison of maternal characteristics: HIPPS-inferred episodes vs. national live births.

	re-March 2020 N3C	2019 National	pre-Pandemic difference	2020-2022 N3C	2020-2021 National	Pandemic difference
**Age**						
15-24	0.24	0.26	-0.02	0.22	0.24	-0.02
25-34	0.56	0.58	-0.02	0.56	0.59	-0.03
35+	0.20	0.16	0.04	0.21	0.17	0.04
**Race**						
White Non-Hispanic	0.54	0.62	-0.08	0.50	0.61	-0.11
Hispanic/Latina	0.16	0.16	0.00	0.19	0.17	0.02
Black Non-Hispanic	0.20	0.16	0.04	0.19	0.15	0.04
Asian/Pacific Islander	0.04	0.02	0.02	0.05	0.02	0.03
Other or not specified	0.06	0.04	0.02	0.08	0.05	0.03
**Total**	**279,903**	**2,894,714**		**536,568**	**5,689,576**	

Note: N3C data reflect full episodes (Cohort 1 Table S7. Panel A). National data come from Bailey et al. 2022.

Table S10. Comparison of Maternal Characteristics: HIPPS inferred Episodes vs CDC Pregnancy COVID Surveillance.

	N3C			CDC		
	**COVID+**	**COVID -**	**diff between covid+ and covid-**	**COVID+**	**COVID -**	**diff between covid+ and covid-**
**Age**						
15-24	26.3	21.9	4.4	26.3	21.8	4.5
25-34	56.2	56.5	-0.3	57.4	57.5	-0.1
35+	17.4	21.6	-4.2	16.2	20.7	-4.5
**Race**						
White Non-Hispanic	48.8	50.3	-1.5	49.1	49.5	-0.4
Hispanic/Latina	21.3	18.2	3.1	27.3	26.7	0.6
Black Non-Hispanic	18.7	18.8	-0.1	15.5	11.9	3.6
Asian/Pacific Islander	3.2	4.7	-1.5	3.6	7.7	-4.1
Other or not specified	7.8	7.8	0.0	4.6	4.2	0.4
**Total**	**62,540**	**474,028**		**92,775**	**1,823,631**	

Note: N3C data reflect full episodes (Cohort 1 Table S7. Panel A). CDC data come from Osterman et al. 2022 and reflect 14 states.

Supplementary Figures

1 Get expected time ranges for each gestational-timing concept

Record	Concept date (A)	Concept	Min month (E)	Max month (E)
R1	2019-12-01	Inhibin A [Multiple of the median] in Serum or Plasma	3.75	5.5
R2	2020-03-29	Glucose; post glucose dose (includes glucose)	6	8
R3	2020-06-02	Gestation period, 11 weeks	2.75	2.75
R4	2020-07-01	Gestation period, 39 weeks	9.75	9.75
R5	2022-01-13	Progesterone [Mass/volume] in Serum or Plasma	1	4
R6	2022-02-24	Neural tube defect risk [Likelihood] in Fetus	3.75	5.5

Discordant record (R3)

2 Compare actual (*A*) vs. expected (*E*) time differences across records

Check 1: Compare the current record (*i*) to all its previous (*i - x*) records

$$\left(\min E_i - \max E_{i-x}\right) - 2 \le \left(A_i - A_{i-x}\right)/30 \le \left(\max E_i - \min E_{i-x}\right) + 2$$

i	*x*	*i - x*	Evaluated Equation	Result
R2	1	R1	-1.50 ≤ 3.97 ≤ 6.25	True
R3	1	R2	-7.25 ≤ 2.17 ≤ -1.25	False
R3	2	R1	-4.75 ≤ 6.13 ≤ 1.00	False
R4	1	R3	5.00 ≤ 0.97 ≤ 9.00	False
R4	2	R2	-0.25 ≤ 3.13 ≤ 5.75	True
R4	3	R1	2.25 ≤ 7.10 ≤ 8.00	True
R5	1	R4	-10.75 ≤ 18.70 ≤ -3.75	False

Check 2: Compare all successive pairs of records surrounding and outwards from the current record (*i*)

$$\left(\min E_{i+y} - \max E_{i-y}\right) - 2 \le \left(A_{i+y} - A_{i-y}\right)/30 \le \left(\max E_{i+y} - \min E_{i-y}\right) + 2$$

i	*y*	*i + y*	*i - y*	Evaluated Equation	Result
R2	1	R3	R1	-4.75 ≤ 6.13 ≤ 1.00	False
R3	1	R4	R2	-0.25 ≤ 3.13 ≤ 5.75	True
R3	2	R5	R1	-6.50 ≤ 25.80 ≤ 2.25	False
R4	1	R5	R3	-3.75 ≤ 19.67 ≤ 3.25	False
R4	2	R6	R2	-6.25 ≤ 23.23 ≤ 1.50	False
R5	1	R6	R4	-8.00 ≤ 20.10 ≤ -2.25	False

Start of new episode (R5)

3 Assign start of new episode if all comparisons from both checks are false

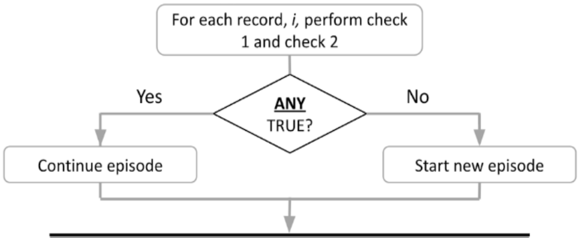

Episode Number	Start Date	End Date
1	2019-11-01	2020-07-01
2	2022-01-13	2022-02-24

Figure S1. Detailed steps of Pregnancy Progression Signature (PPS) Algorithm using a fictional example patient. 1) The list of 74 concepts (Table S3) was used to search for signatures of progressing pregnancy concepts across each patient record using an adaptation of longest increasing consecutive subsequence analysis. Outlined in red is a record that is discordant with the other records. 2) Time intervals across these concepts in the patient data were compared to their expected intervals in terms of gestational month assigned by clinicians (Table S3) in order to build plausible pregnancy episodes, where i is the current gestational timing

record in the patient data, A is the actual (observed) patient record date, E is the expected concept gestational timing in months, x is the iteration across records for check 1 and y is the iteration across records for check 2. Two additional months were added to the expected ranges in order to allow for a margin of error in when the concepts were recorded in the data. Outlined in purple is the record that indicates the start of a new episode. 3) Upon checking each record, i, if any records comparison from check 1 or check 2 evaluate to a true result, the episode is continued and it is assumed the patient still is progressing with the same pregnancy, i.e. the actual difference in dates matches the expected difference in dates based on the concepts gestational time ranges. If none of the checks evaluate to true and there are > 2 months (minimum retry period for any pregnancy outcome) between consecutive record dates, a new episode begins. The large number of comparisons performed per date in the patient records was due to historical records in the patient data intervening with the true progressing sequence of pregnancy-specific gestational timing concepts, meaning that concept comparisons often had to be skipped and instead surrounding concepts were assessed to determine whether to continue an episode or start a new one.

A)

B)

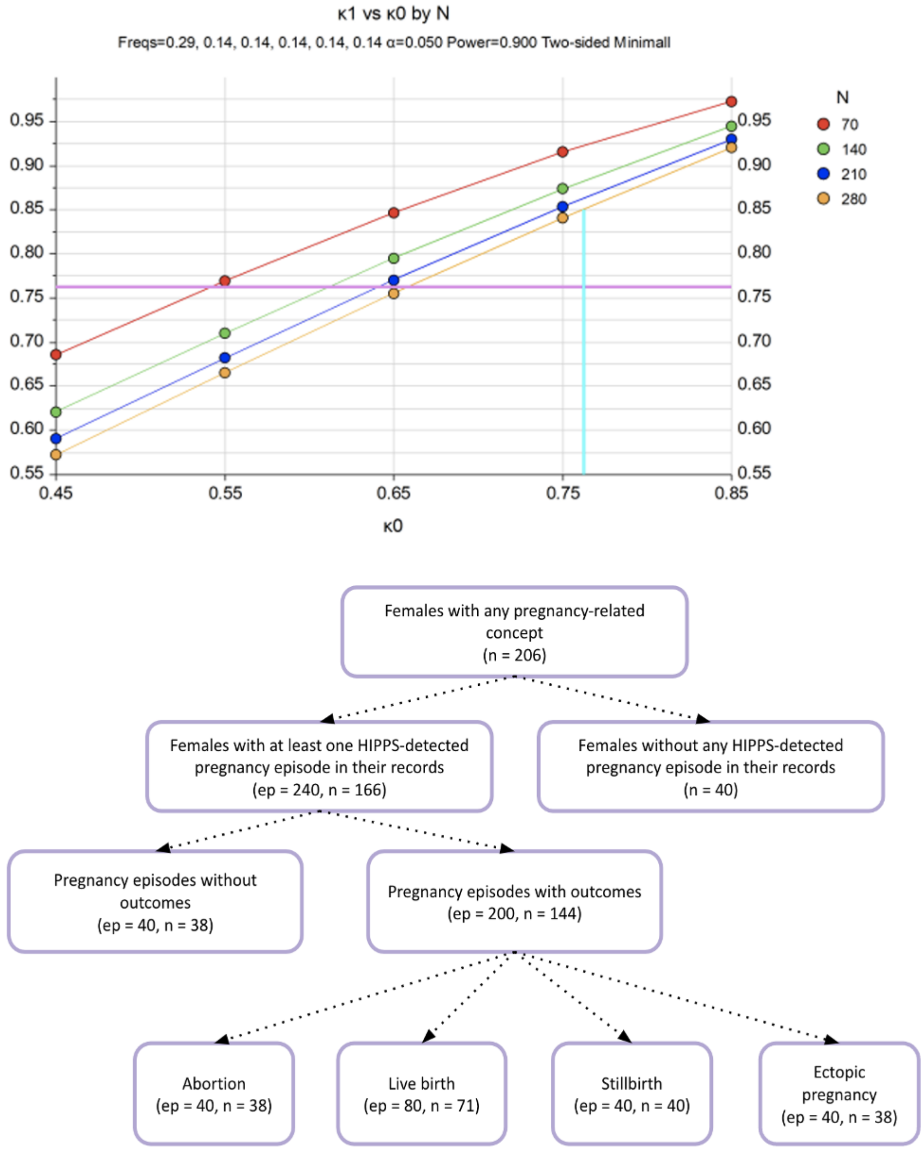

Figure S2. Sample size determination and selection of patients and episodes for independent clinician validation. A) Plot of minimally detectable measure of agreement (Cohen's $\boldsymbol{\kappa}$; 'κ1' vertical axis) across six pregnancy episode classification categories (allowing 2:1 ratio for live birth outcomes to non-live-birth outcomes) demonstrating ranges for both total sample size N=70*f* {*f*=1,...,4} and referent null value $\boldsymbol{\kappa}_0$ 'κ0' (horizontal axis), when targeting 90% power at the 5% significance level. Note that decreases in minimally detectable values are greater for twofold sample size (*f*=2) relative to the internal pilot sample size of 70 (60 episodes and 10 non-pregnant persons) than it is when increasing *f* from three- to fourfold increases in total sample size. B) Flowchart of episode and patient selection where ep = number of pregnancy episodes and n = number of patients (noting that individual patients could contribute to more than one pregnancy episode and, thus, be randomly sampled again for a separate pregnancy episode). As these cases were randomly selected for clinician validation they do not reveal information about the N3C subpopulation and are not subject to the same N3C policy regarding masking small numbers of patients.

Concept category	Count and proportion of sites (out of 72) with at least one person matching the concept		Count and proportion of persons (out of 633,914 across all sites) matching the concept		Count and proportion of unique persons who have the concept recorded but who are missing corresponding values	
Gestation period, X weeks	70	97.2%	499,655	78.8%	0	0.0%
Gestational age	22	30.6%	20,184	3.2%	11,189	55.4%
Last menstrual period start date	21	29.2%	22,158	3.5%	16,532	74.6%
Delivery date Estimated	19	26.4%	17,280	2.7%	10,217	59.1%
Gestational age in weeks	19	26.4%	10,761	1.7%	2,818	26.2%
Gestational age in days	19	26.4%	6,014	0.9%	495	8.2%
Gestational age Estimated	18	25.0%	10,352	1.6%	381	3.7%
Gestational age Estimated from conception date	15	20.8%	4,980	0.8%	4,980	100.0%
Gestational age US composite estimate	10	13.9%	5,839	0.9%	5,687	97.4%
Delivery date Estimated from last menstrual period	9	12.5%	3,790	0.6%	3,229	85.2%
Delivery date US composite estimate	9	12.5%	4,010	0.6%	3,108	77.5%
Gestational age Estimated from last menstrual period	8	11.1%	4,187	0.7%	3,775	90.2%
Estimated date of delivery	5	6.9%	8,776	1.4%	8,776	100.0%
Date of gestational age estimate	2	2.8%	3,022	0.5%	3,021	100.0%
Gestational age Estimated from physical exam	1	1.4%	3,050	0.5%	3,050	100.0%
Date of last menstrual period	1	1.4%	365	0.1%	363	99.5%
Length of gestation at birth	1	1.4%	11,317	1.8%	34	0.3%

Figure S3. Count and proportion of concepts by persons and sites.

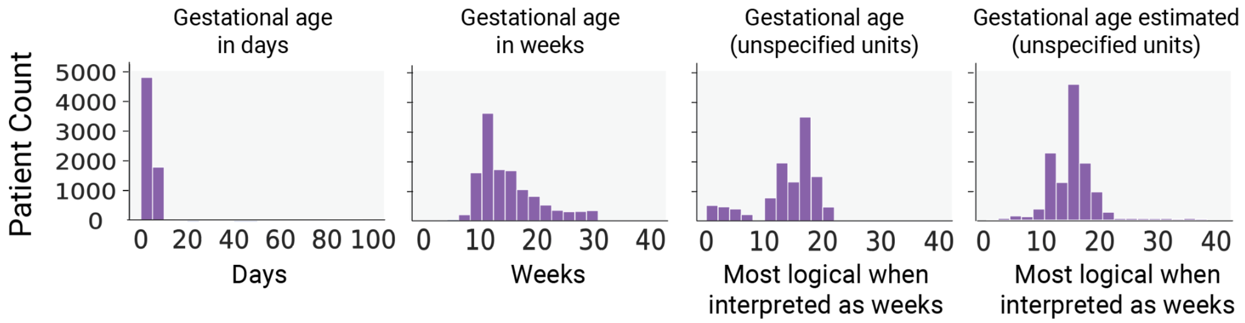

Figure S4. Distribution of values of gestational age concepts in the initial reference cohort.

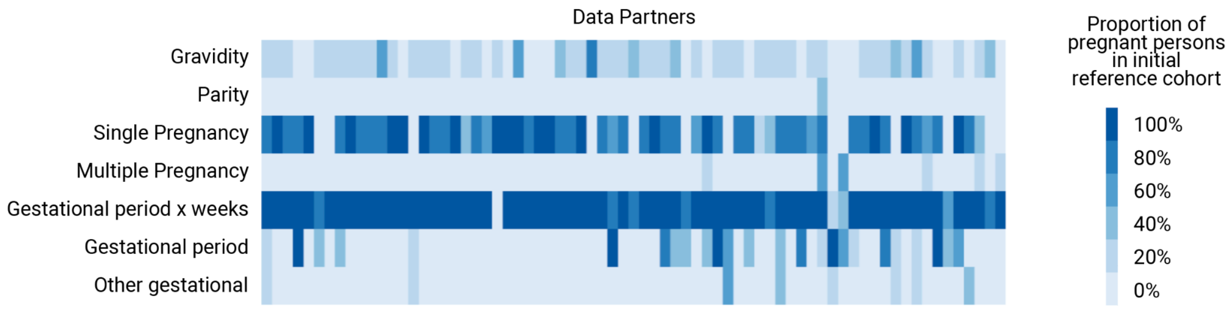

Figure S5. Heatmap of proportion of pregnant persons in initial reference cohort by site with concept for Gravidity, Parity, Single Pregnancy, Multiple Pregnancy, Gestation Period, X weeks, Other Gestational, and Number of Fetuses.

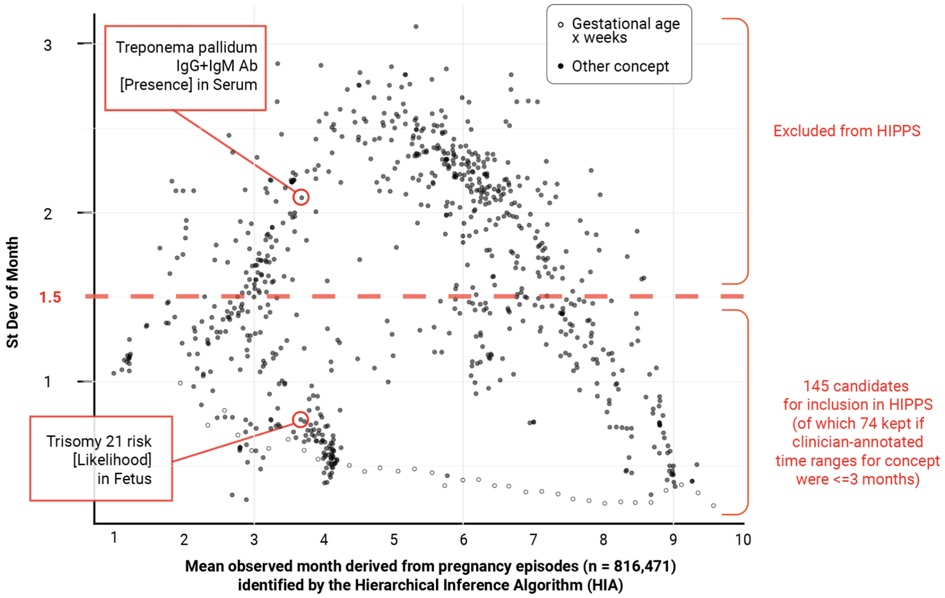

Figure S6. Empirical estimation of threshold for gestational timing specificity for concepts. The standard deviation of mean month the concept occurs during pregnancy (for any outcome category) is used as an indicator of gestational timing specificity. Two concepts are called out as examples that were excluded and included in the HIPPS approach.

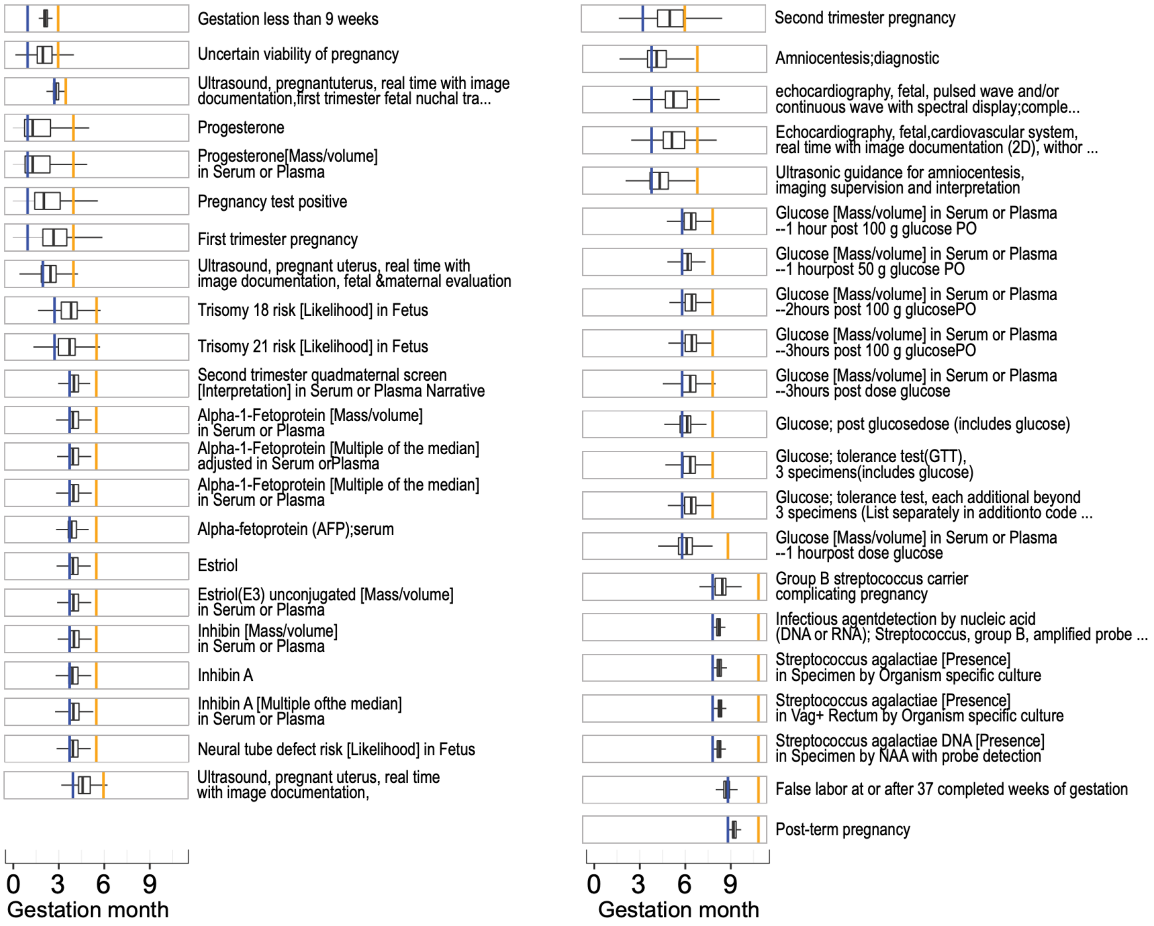

Figure S7. Gestational timing precision for clinician-curated concepts of > 1 week and ≤ 3 months (GR3m). Agreement between clinician-assigned gestational ranges (blue bars = min, orange bar = max) and IQR of concepts in the data (box plots) for the concepts selected as input for algorithm 2 with clinician gestational time ranges > 1 week and ≤ 3 months. For simplicity, we have not shown the "Gestation period, X weeks" (GW) concepts as these are all narrow by definition.

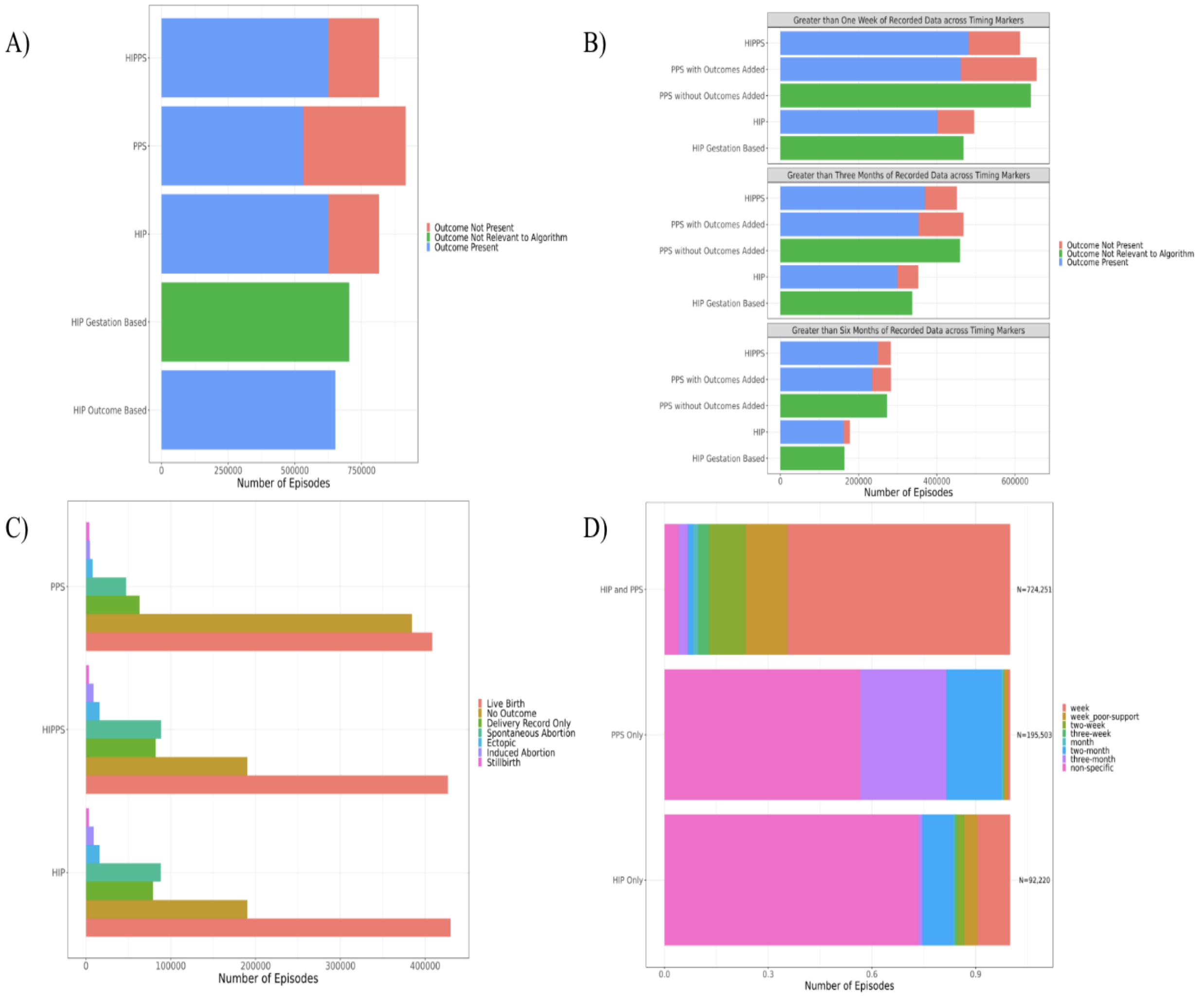

Figure S8. Comparison of component algorithms. A) Number of episodes inferred and number of episodes with outcomes inferred for each component algorithm compared to baseline (HIP Outcome Based); HIP contains two components (outcome-based and gestation-based). B) Number of episodes of each component algorithm with timespan of recorded data across gestational timing markers stratified by 1 week, 3 months, and 6 months (baseline not included because it does not infer episode start). C) Number of episodes by outcome type for PPS, HIP, and HIPPS. D) Proportion of episodes by start date precision level for PPS-only (not included in final HIPPS episodes), HIP-only (included in final HIPPS episodes), and PPS/HIP overlap (included in full HIPPS episodes). Precision for full HIPPS (816K) shown in Figure 5F.

References

1 Matcho A, Ryan P, Fife D, *et al.* Inferring pregnancy episodes and outcomes within a network of observational databases. *PLoS One* 2018;**13**:e0192033.

2 Odysseus Data Services, Inc. Athena 2015-2022 Version 1.12.2.8.210316.0857n OMOP Vocabulary version: v5.0 22-JUN-22. Athena. 22 June 2022.athena.ohdsi.org (accessed 14 July 2022).

3 Observational Health Data Sciences and Informatics (OHDSI) ATLAS. ATLAS. https://atlas.ohdsi.org/ (accessed 14 July 2022).

4 CDC. What is stillbirth? Centers for Disease Control and Prevention. 2020.https://www.cdc.gov/ncbddd/stillbirth/facts.html (accessed 22 Jul 2022).

5 Altman MR, Colorafi K, Daratha KB. The Reliability of Electronic Health Record Data Used for Obstetrical Research. *Appl Clin Inform* 2018;**9**:156–62.

6 Schmaltz S, Vaughn J, Elliott T. Comparison of electronic versus manual abstraction for 2 standardized perinatal care measures. *J Am Med Inform Assoc* 2022;**29**:789–97.

7 Moll K, Wong HL, Fingar K, *et al.* Validating Claims-Based Algorithms Determining Pregnancy Outcomes and Gestational Age Using a Linked Claims-Electronic Medical Record Database. *Drug Saf* 2021;**44**:1151–64.

8 Berry KJ, Johnston JE, Mielke PW Jr. *A Chronicle of Permutation Statistical Methods: 1920–2000, and Beyond*. Springer Science & Business Media 2014.

9 Pepe MS. *The Statistical Evaluation of Medical Tests for Classification and Prediction*. Oxford University Press on Demand 2004.

10 Gwet KL. Computing inter-rater reliability and its variance in the presence of high agreement. *Br J Math Stat Psychol* 2008;**61**:29–48.

11 Wongpakaran N, Wongpakaran T, Wedding D, *et al.* A comparison of Cohen’s Kappa and Gwet’s AC1 when calculating inter-rater reliability coefficients: a study conducted with personality disorder samples. *BMC Med Res Methodol* 2013;**13**:1–7.

12 irrCAC: Computing Chance-Corrected Agreement Coefficients (CAC). Comprehensive R Archive Network (CRAN). https://CRAN.R-project.org/package=irrCAC (accessed 2 Mar 2023).

13 Mehta CR. StatXact: A Statistical Package for Exact Nonparametric Inference. The American Statistician. 1991;**45**:74. doi:10.2307/2685246

14 Bailey MJ, Currie J, Schwandt H. The Covid-19 Baby Bump: The Unexpected Increase in U.S. Fertility Rates in Response to the Pandemic. 2022. doi:10.3386/w30569

15 Maternal and infant characteristics among women with confirmed or presumed cases of Coronavirus disease (COVID-19) during pregnancy. 2022. https://www.cdc.gov/nchs/covid19/technical-linkage.htm (accessed 14 Nov 2022).
